# Supplementary material for: Assessing the utility of COVID-19 case reports as a leading indicator for hospitalization forecasting in the United States
Source: medRxiv. 2023 Mar 10:2023.03.08.23286582. Preprint. [Version 1] doi: 10.1101/2023.03.08.23286582 (PMC10029058; doi:10.1101/2023.03.08.23286582)

California case data as of: 2021−04−26

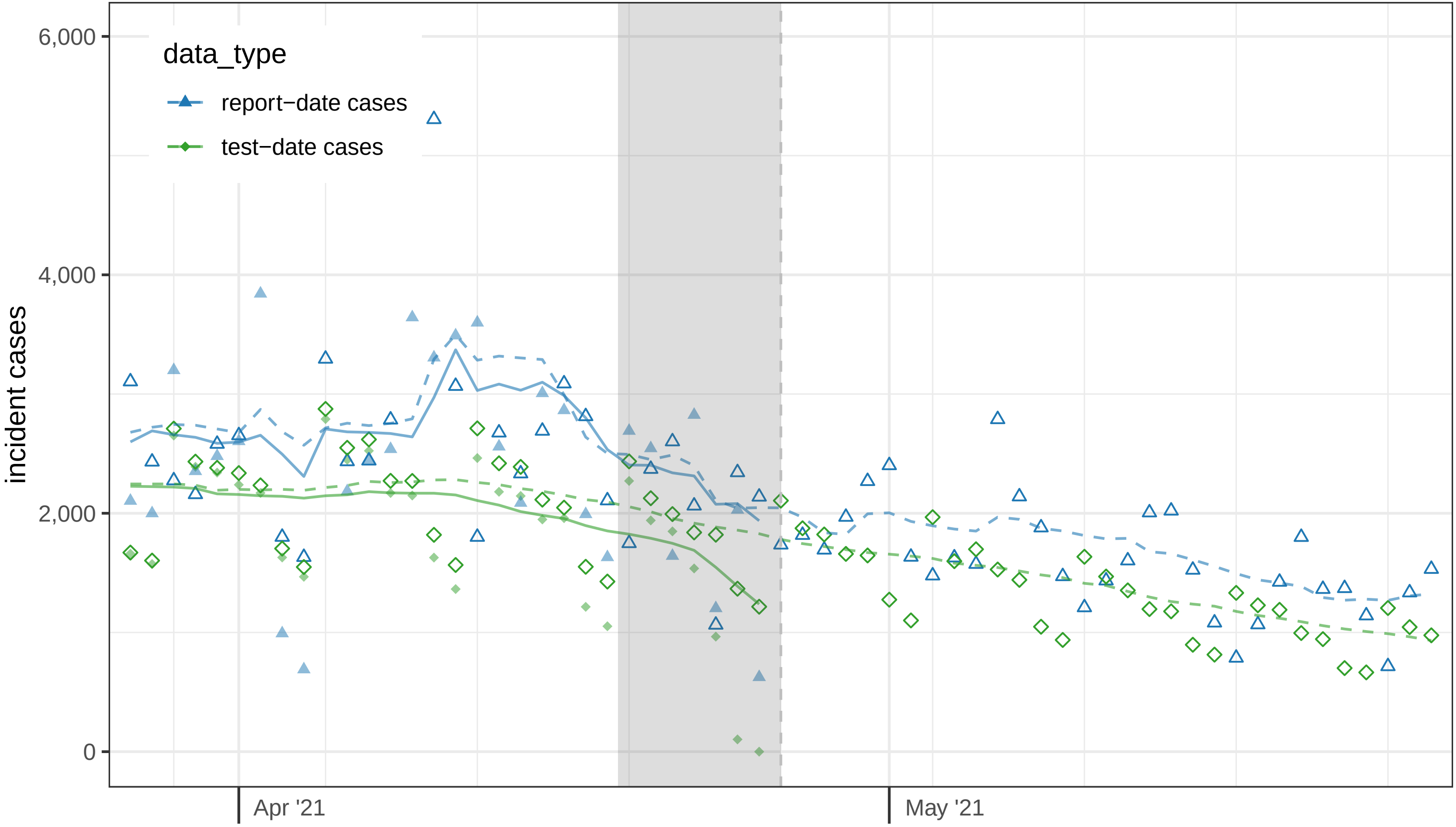

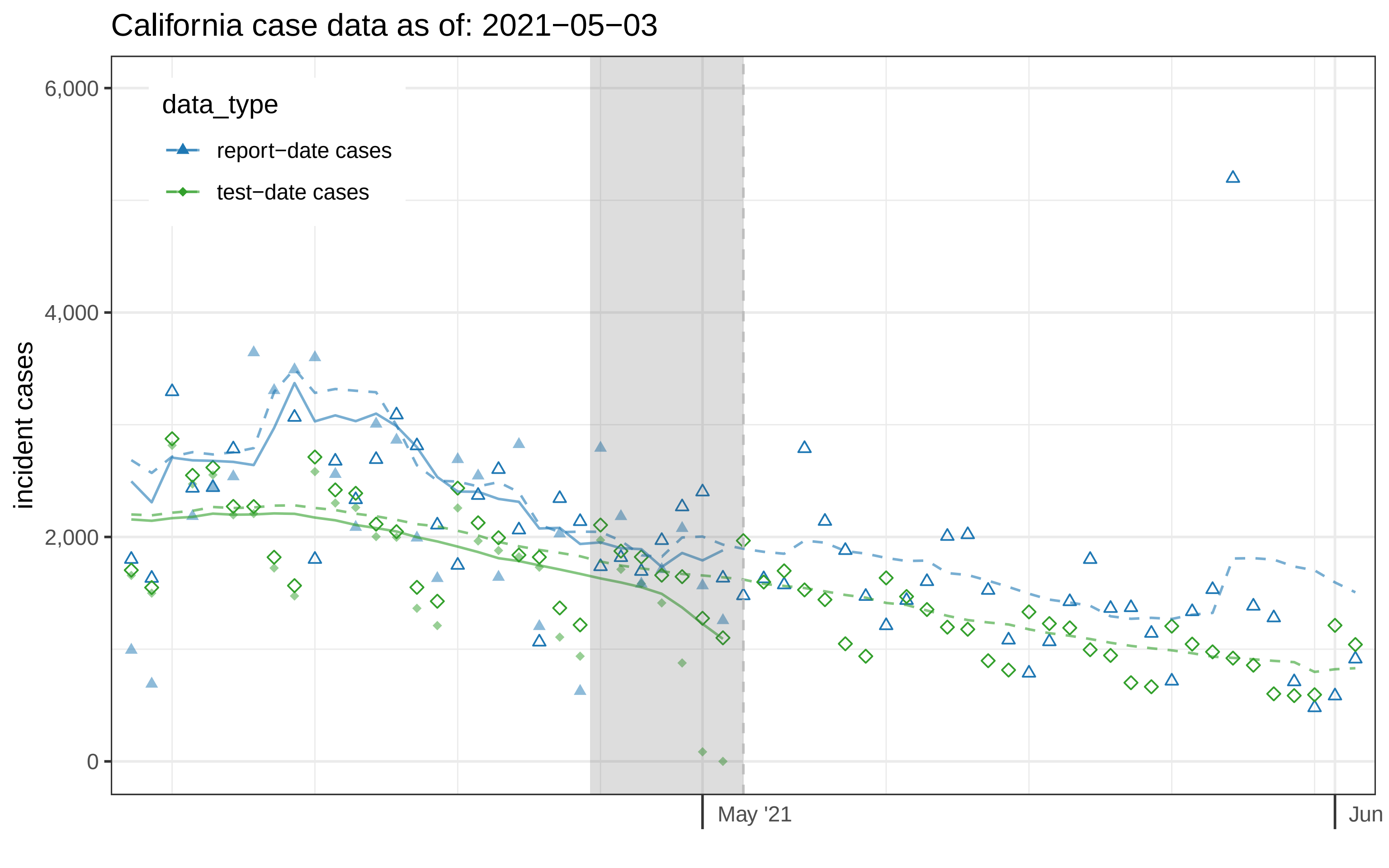
California case data as of: 2021−05−03
data_type
report−date cases
test−date cases
incident cases
6,000
4,000
2,000
0
May '21
Jun

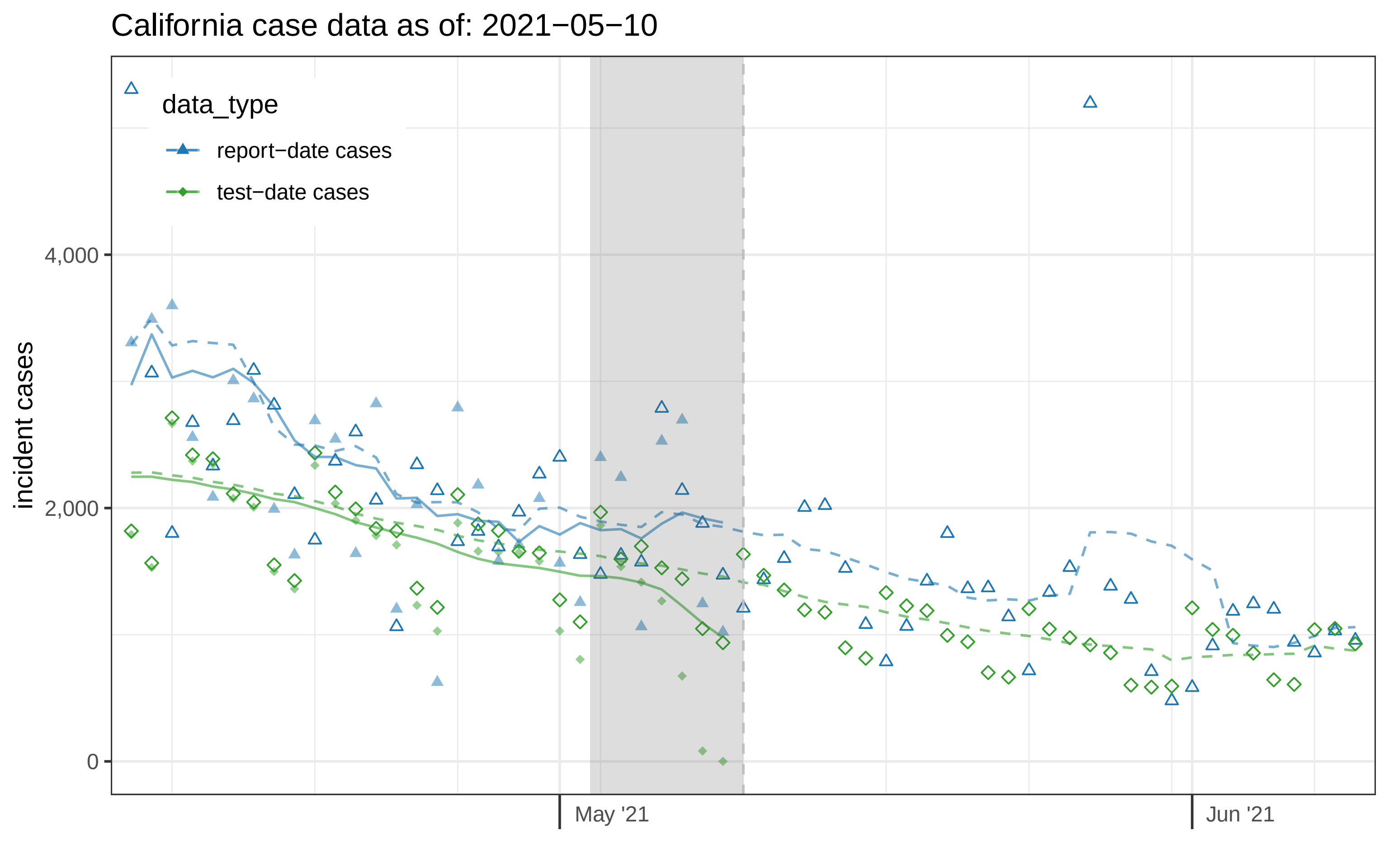
California case data as of: 2021−05−10
data_type
report−date cases
test−date cases
incident cases
4,000
2,000
0
May '21
Jun '21

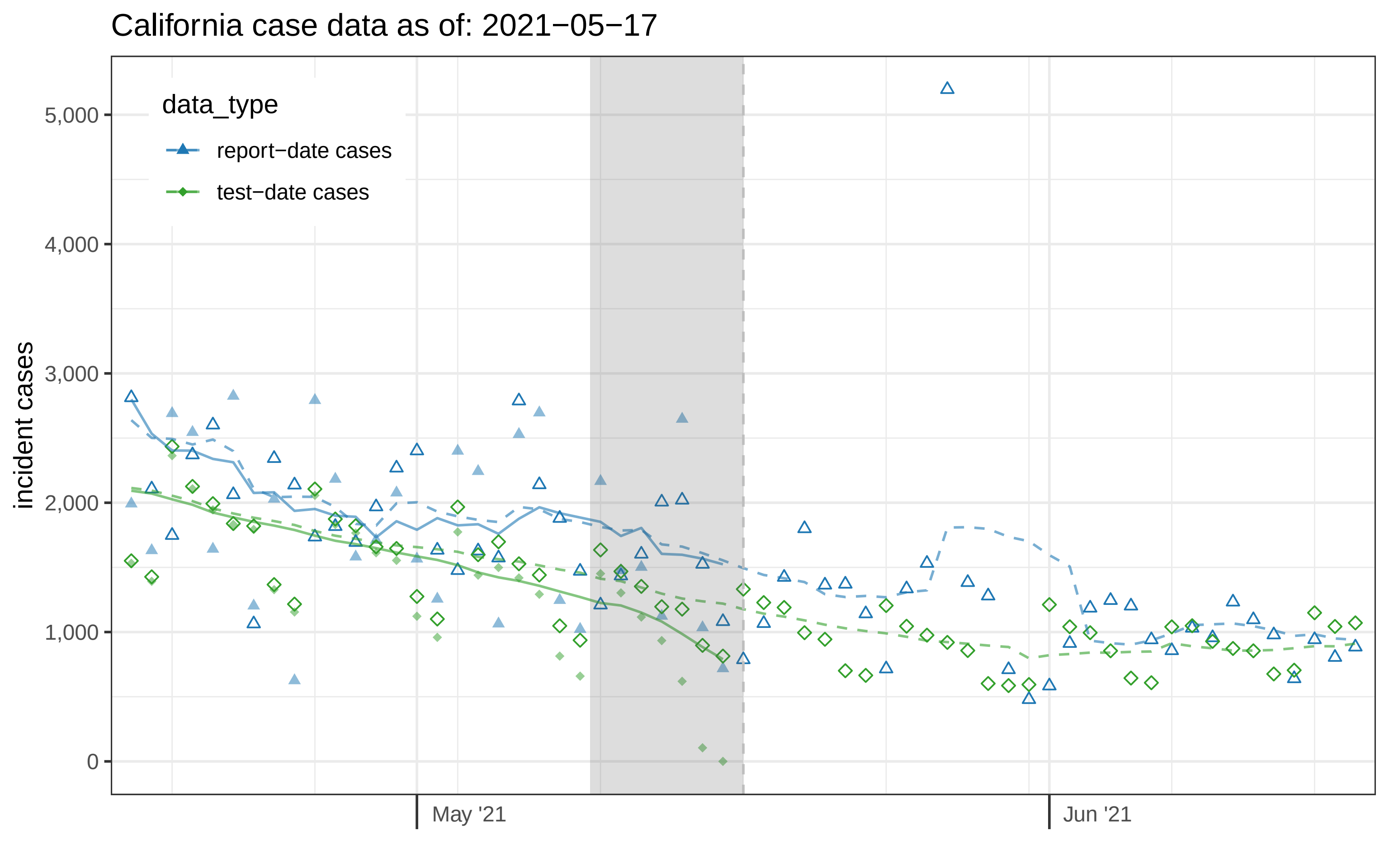

California case data as of: 2021−05−17
data_type
report−date cases
test−date cases
incident cases
5,000
4,000
3,000
2,000
1,000
0
May '21
Jun '21

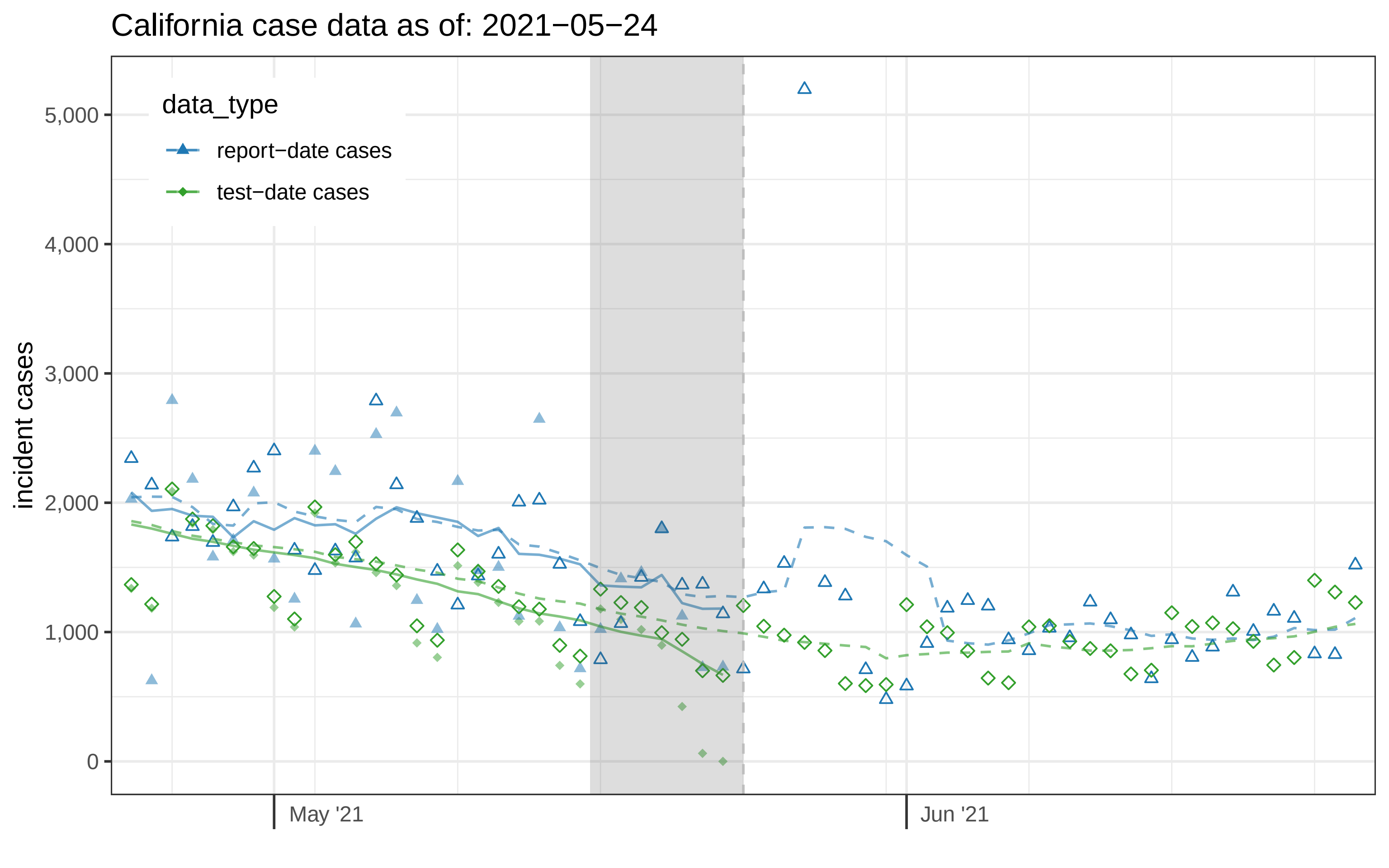
California case data as of: 2021−05−24
data_type
report−date cases
test−date cases
incident cases
5,000
4,000
3,000
2,000
1,000
0
May '21
Jun '21

California case data as of: 2021−05−31

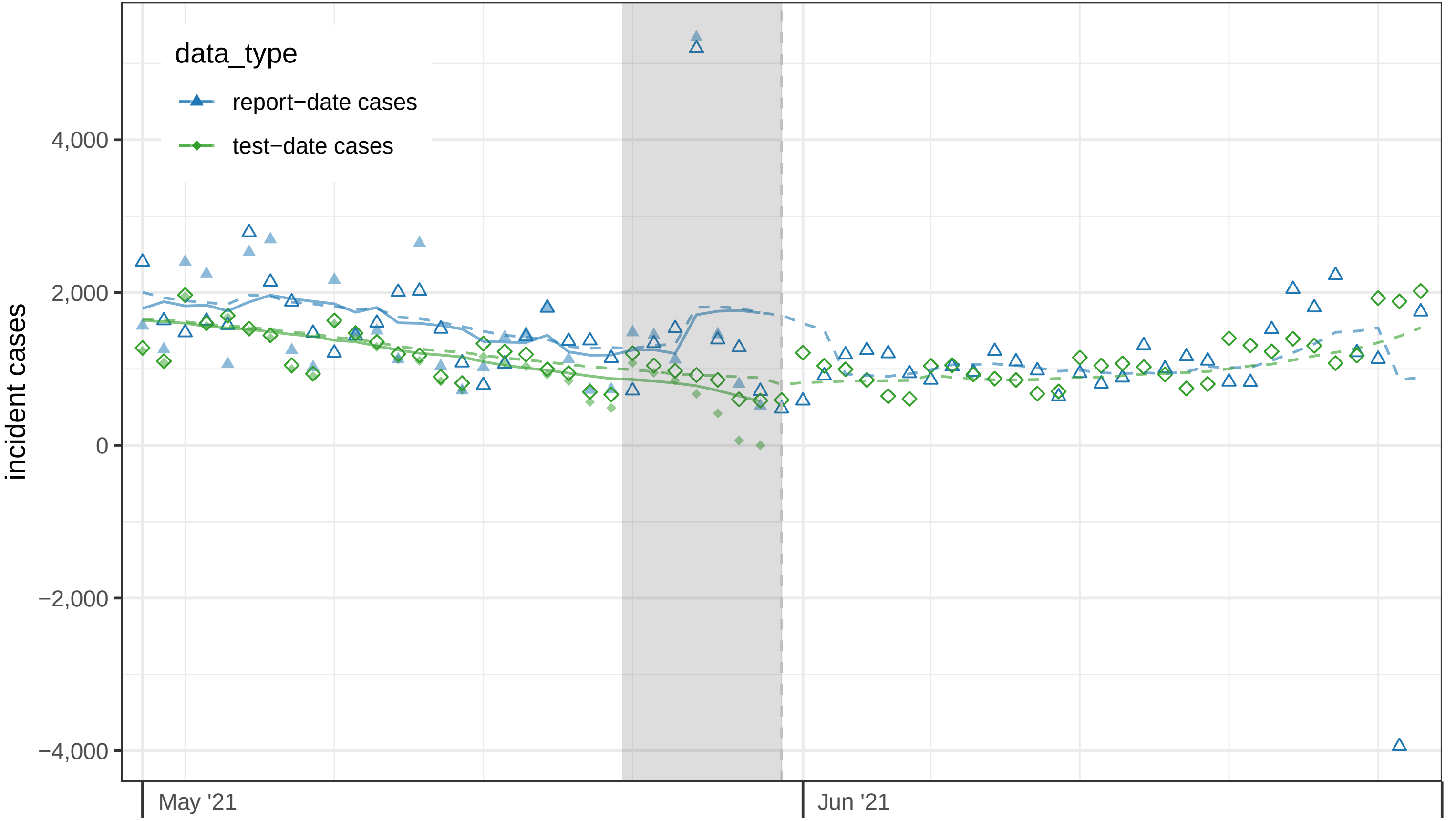

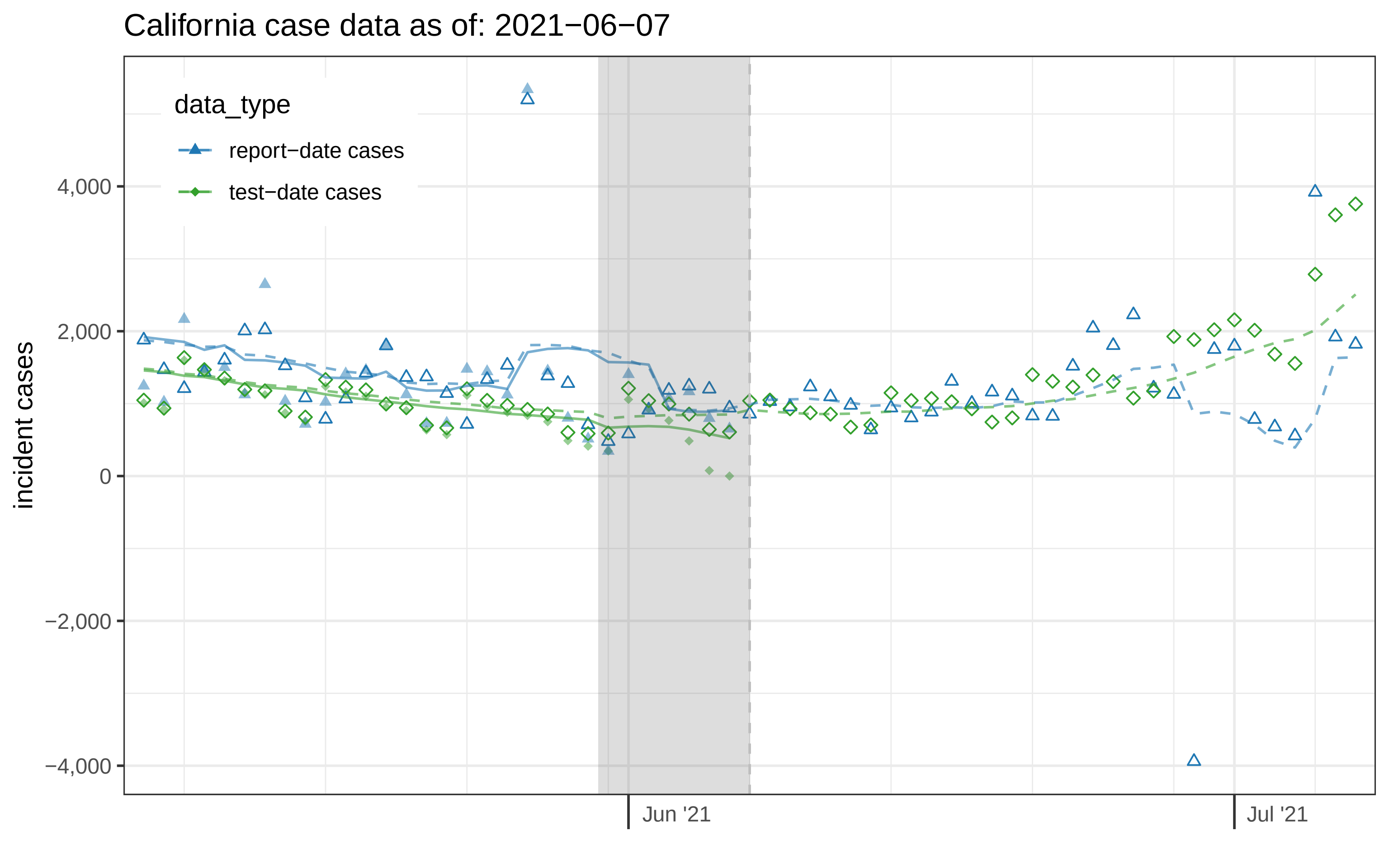

California case data as of: 2021−06−07
data_type
report−date cases
test−date cases
incident cases
4,000
2,000
0
−2,000
−4,000
Jun '21
Jul '21

California case data as of: 2021−06−14

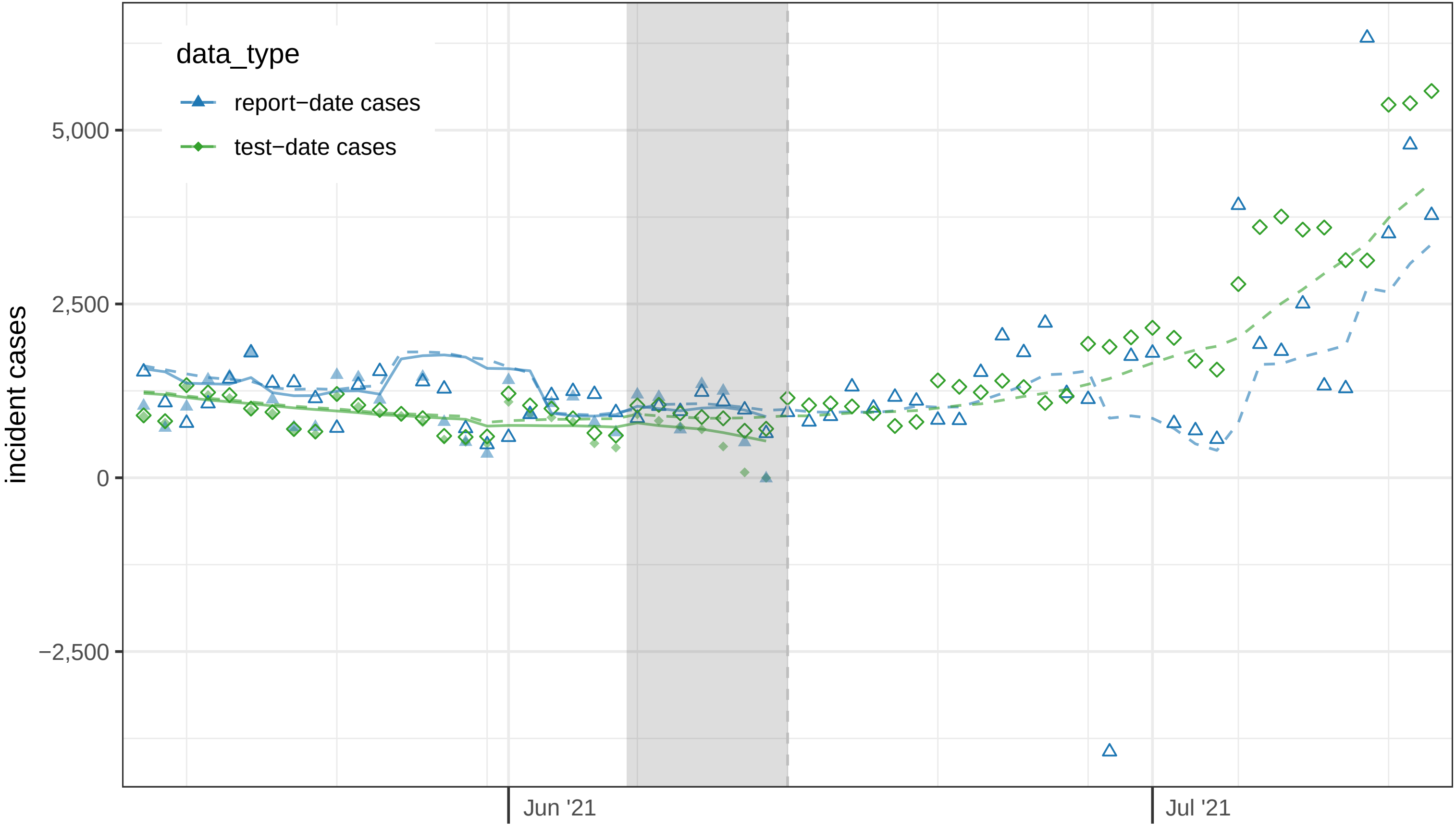

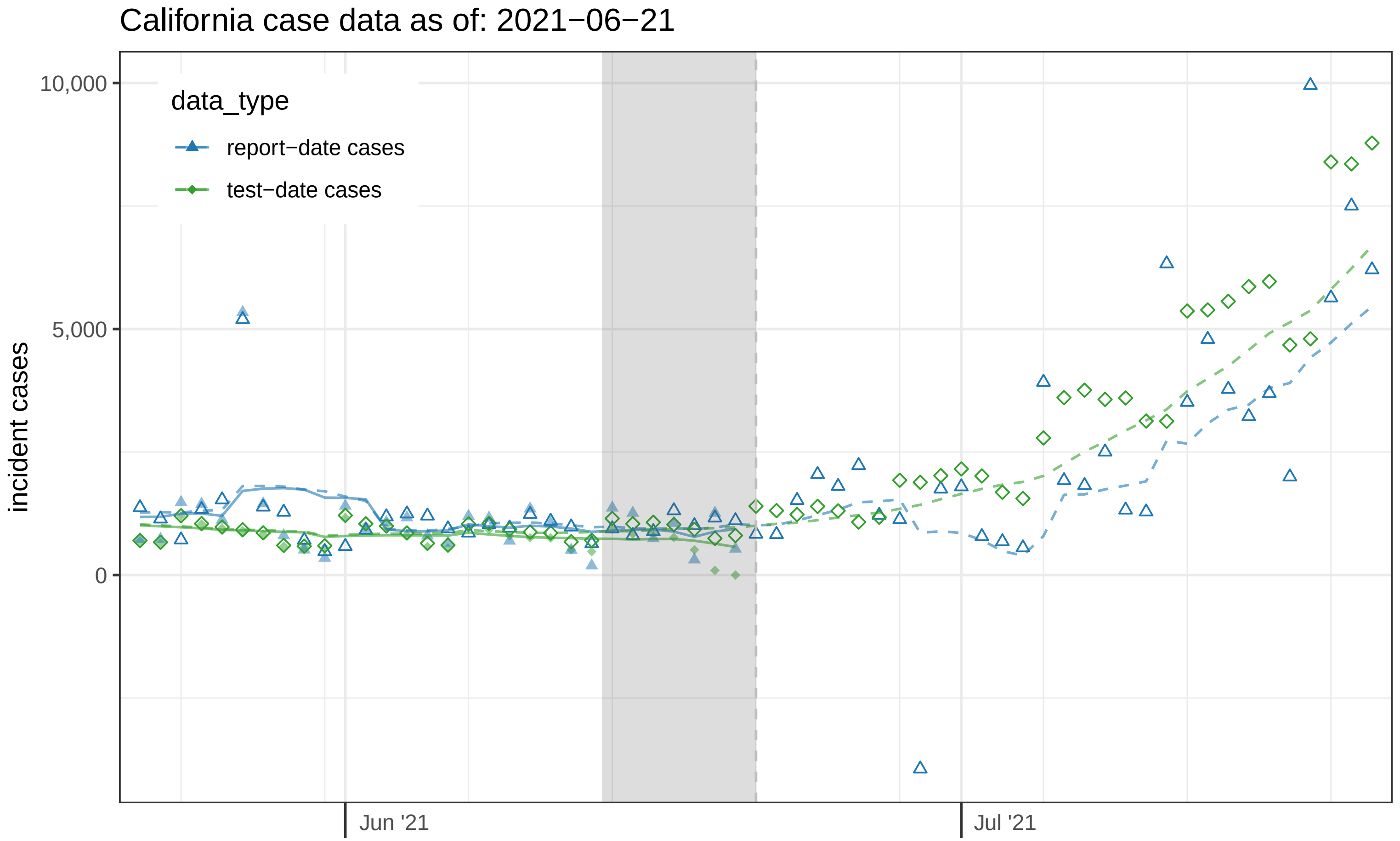
California case data as of: 2021−06−21
data_type
report−date cases
test−date cases
incident cases
10,000
5,000
0
Jun '21
Jul '21

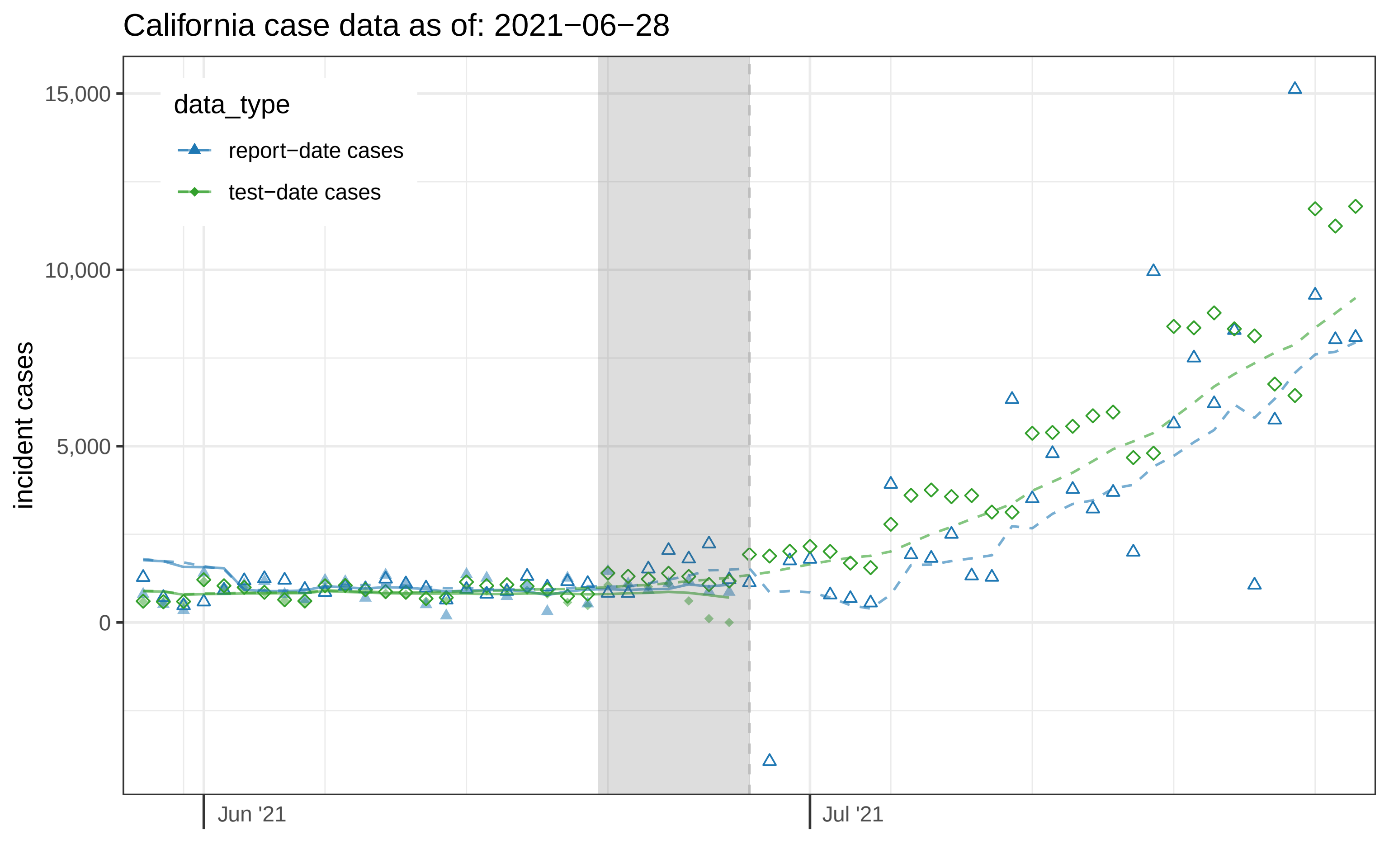
California case data as of: 2021−06−28
data_type
report−date cases
test−date cases
incident cases
15,000
10,000
5,000
0
Jun '21
Jul '21

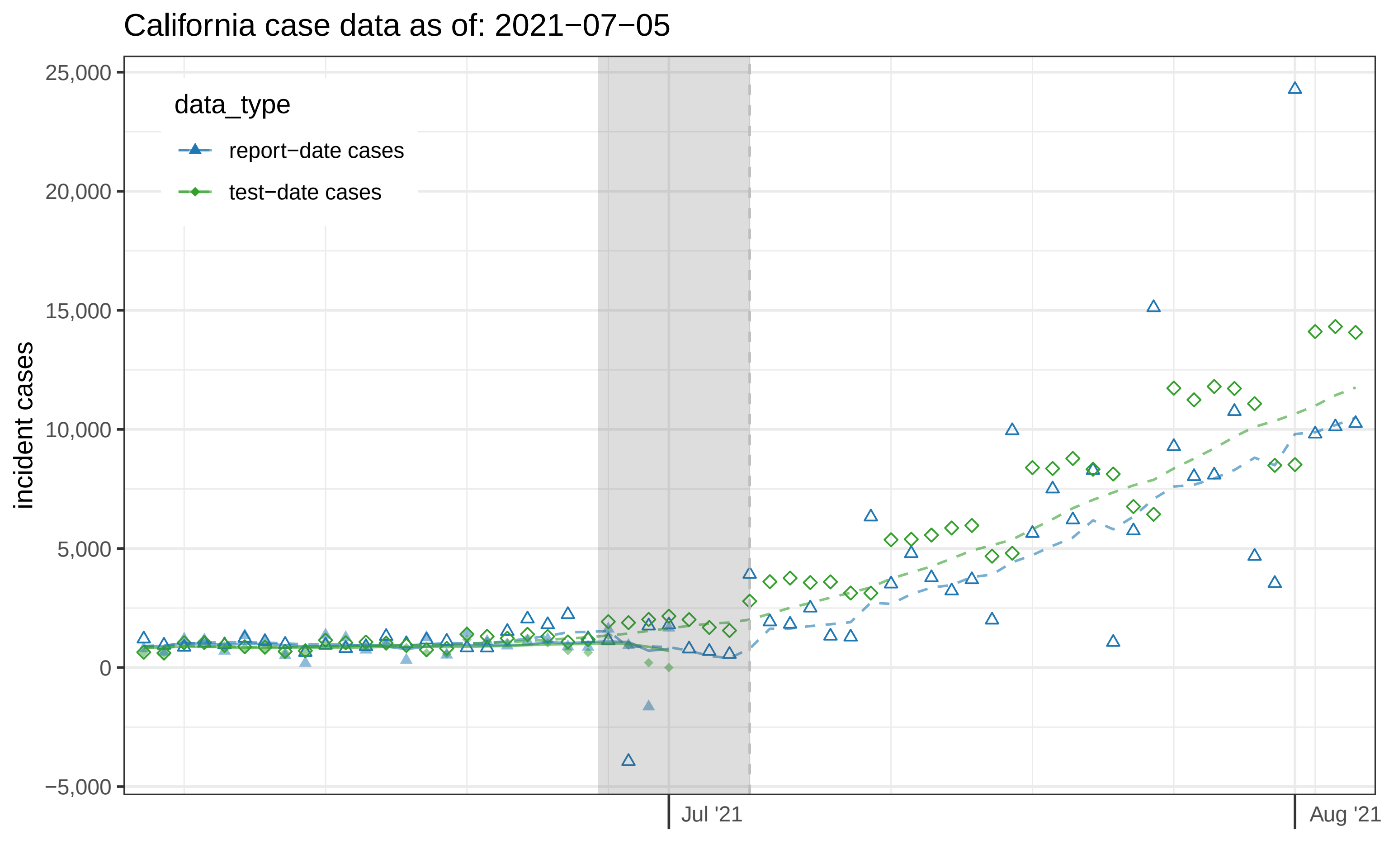
California case data as of: 2021−07−05
incident cases
25,000
20,000
15,000
10,000
5,000
0
−5,000
data_type
report−date cases
test−date cases
Jul '21
Aug '21

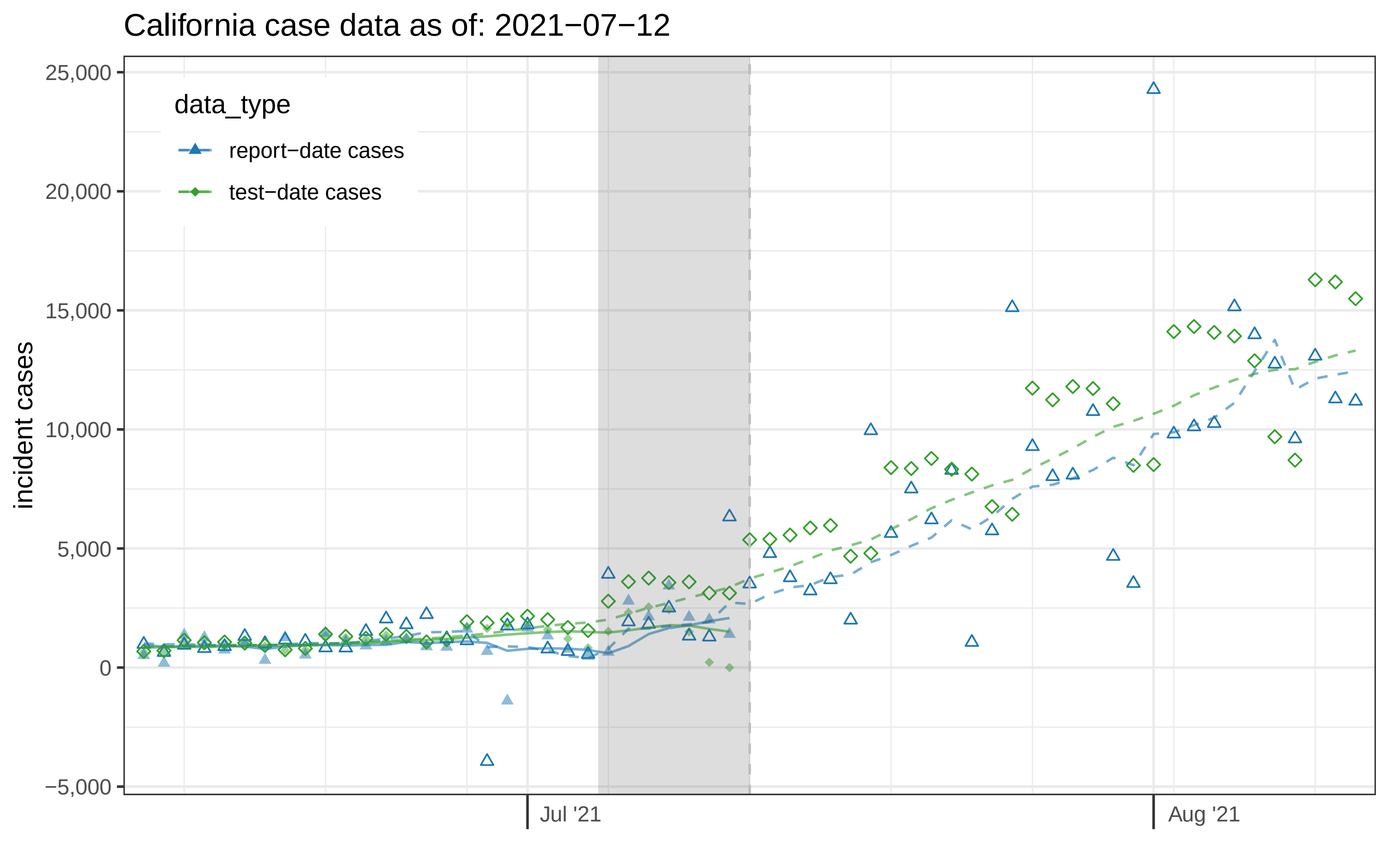

California case data as of: 2021−07−12
data_type
report−date cases
test−date cases
incident cases
25,000
20,000
15,000
10,000
5,000
0
−5,000
Jul '21
Aug '21

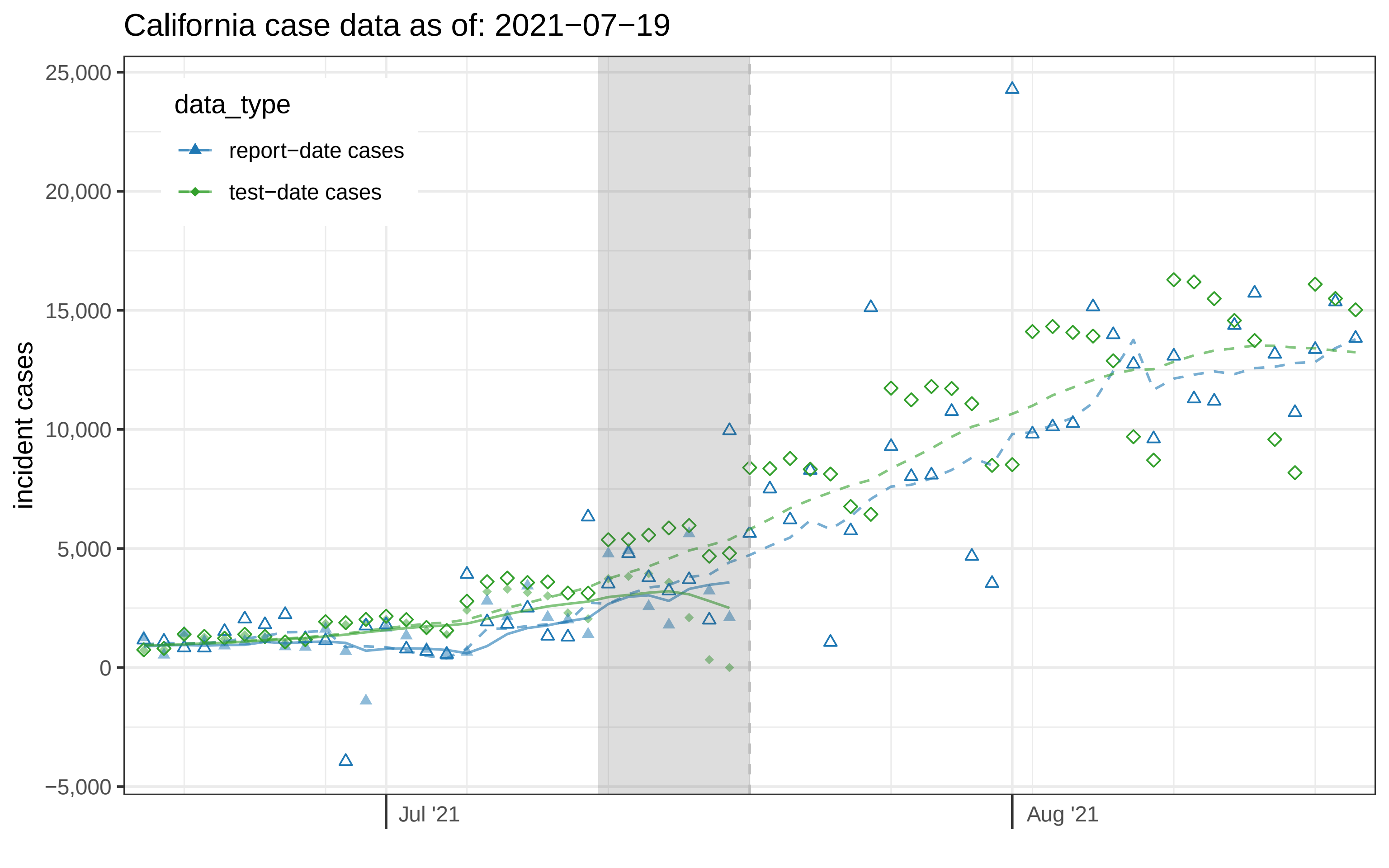
California case data as of: 2021−07−19
data_type
report−date cases
test−date cases
incident cases
25,000
20,000
15,000
10,000
5,000
0
−5,000
Jul '21
Aug '21

California case data as of: 2021−07−26

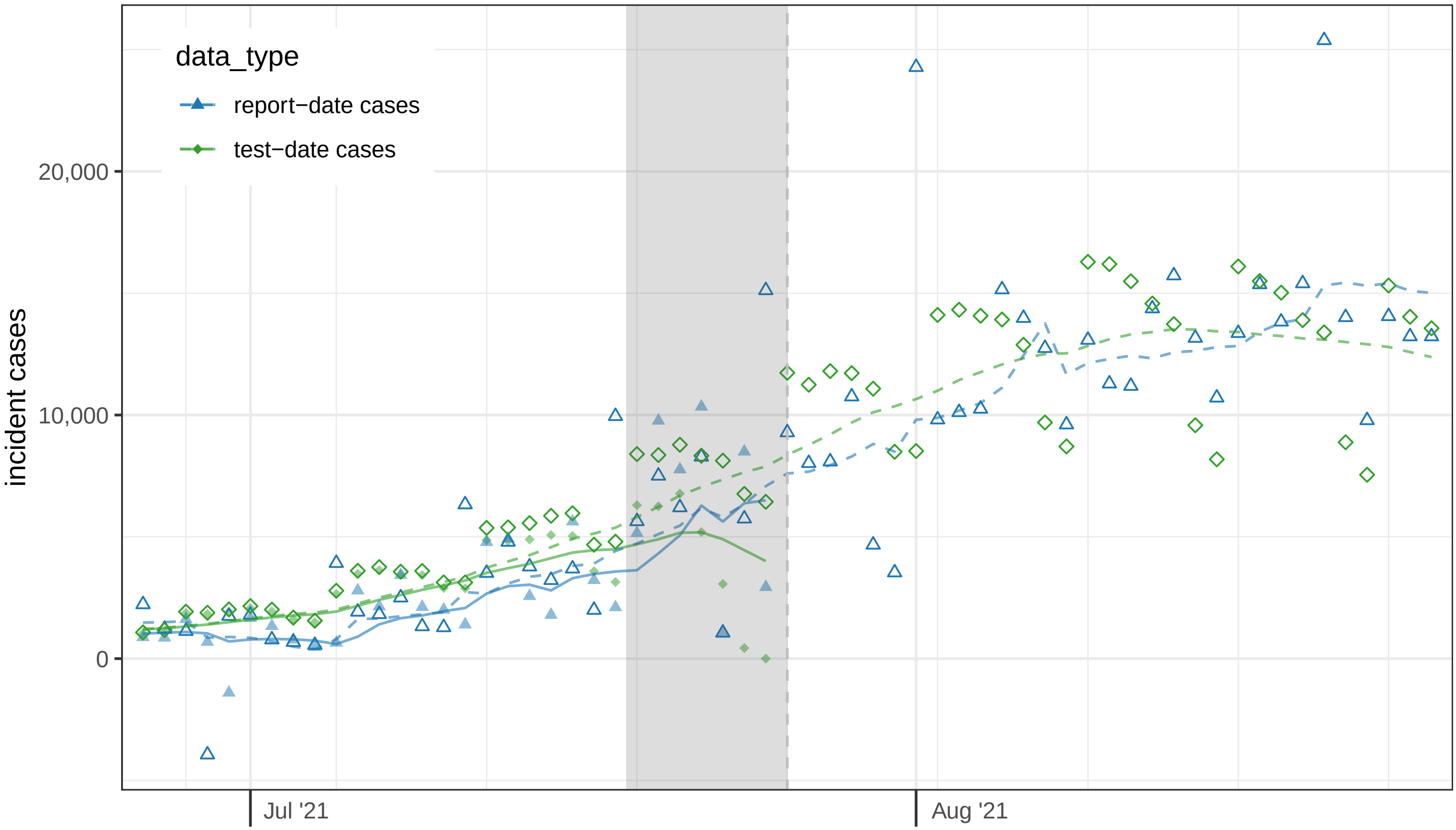

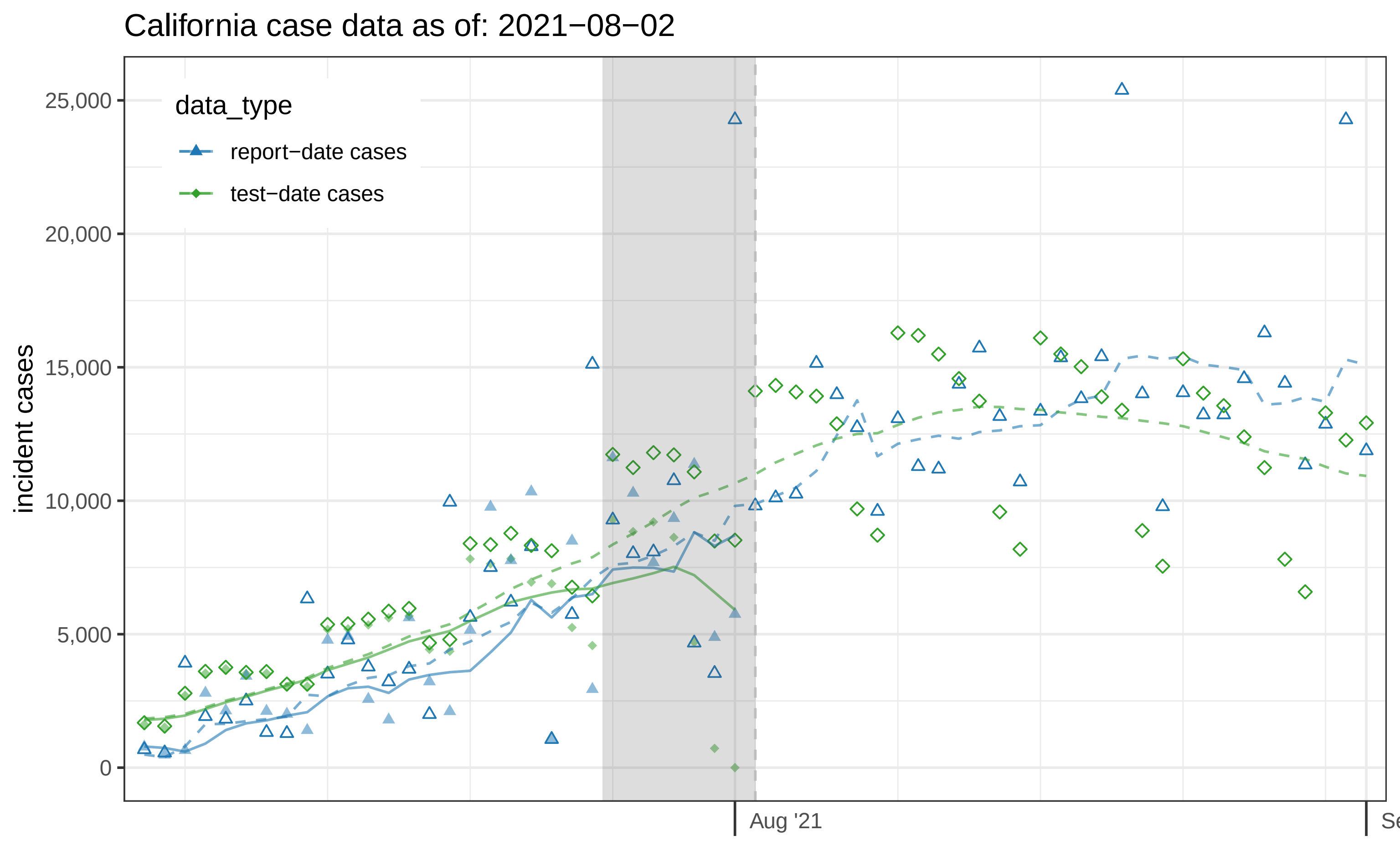

California case data as of: 2021−08−02
data_type
report−date cases
test−date cases
incident cases
25,000
20,000
15,000
10,000
5,000
0
Aug '21
Se

California case data as of: 2021−08−09

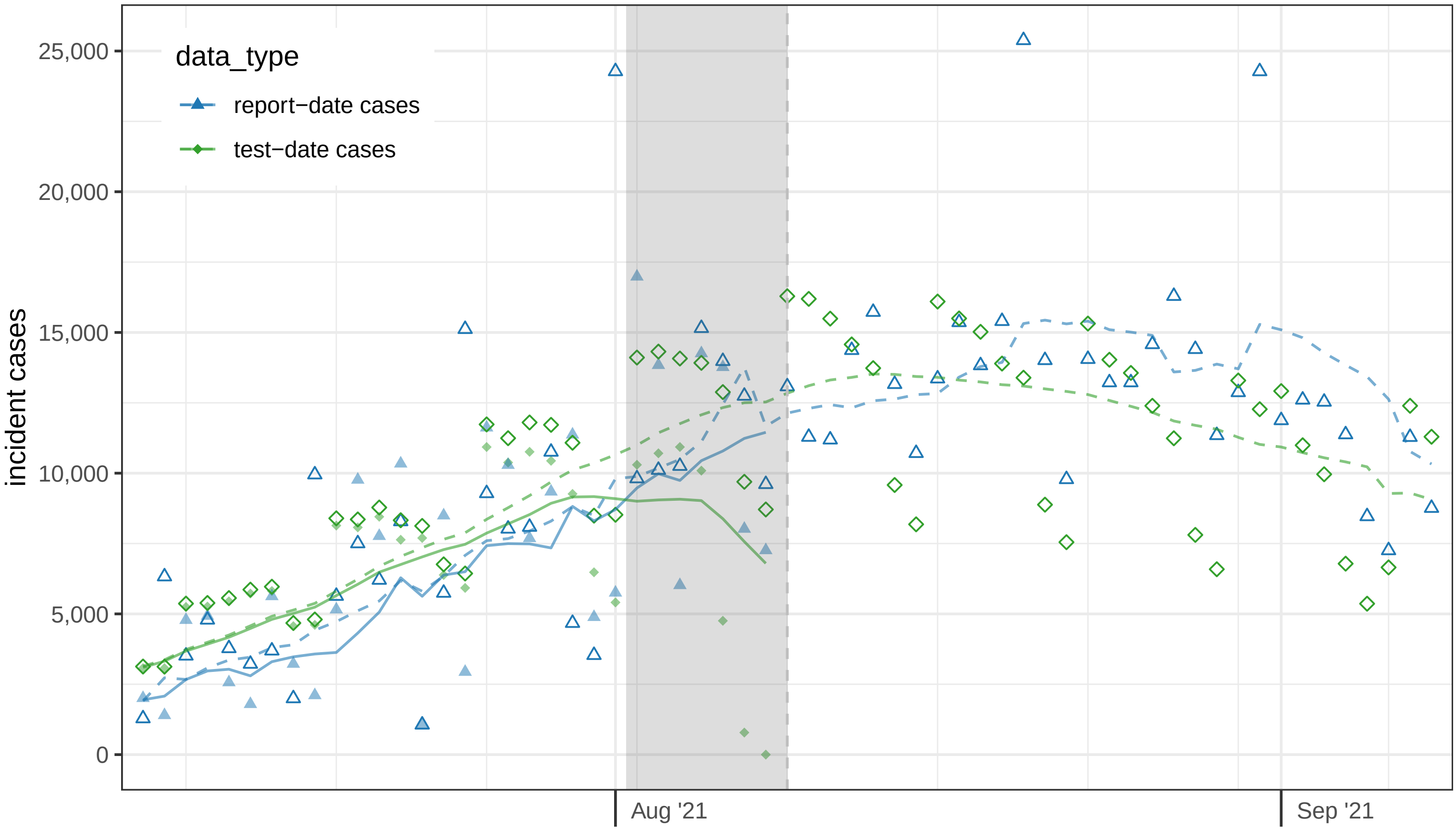

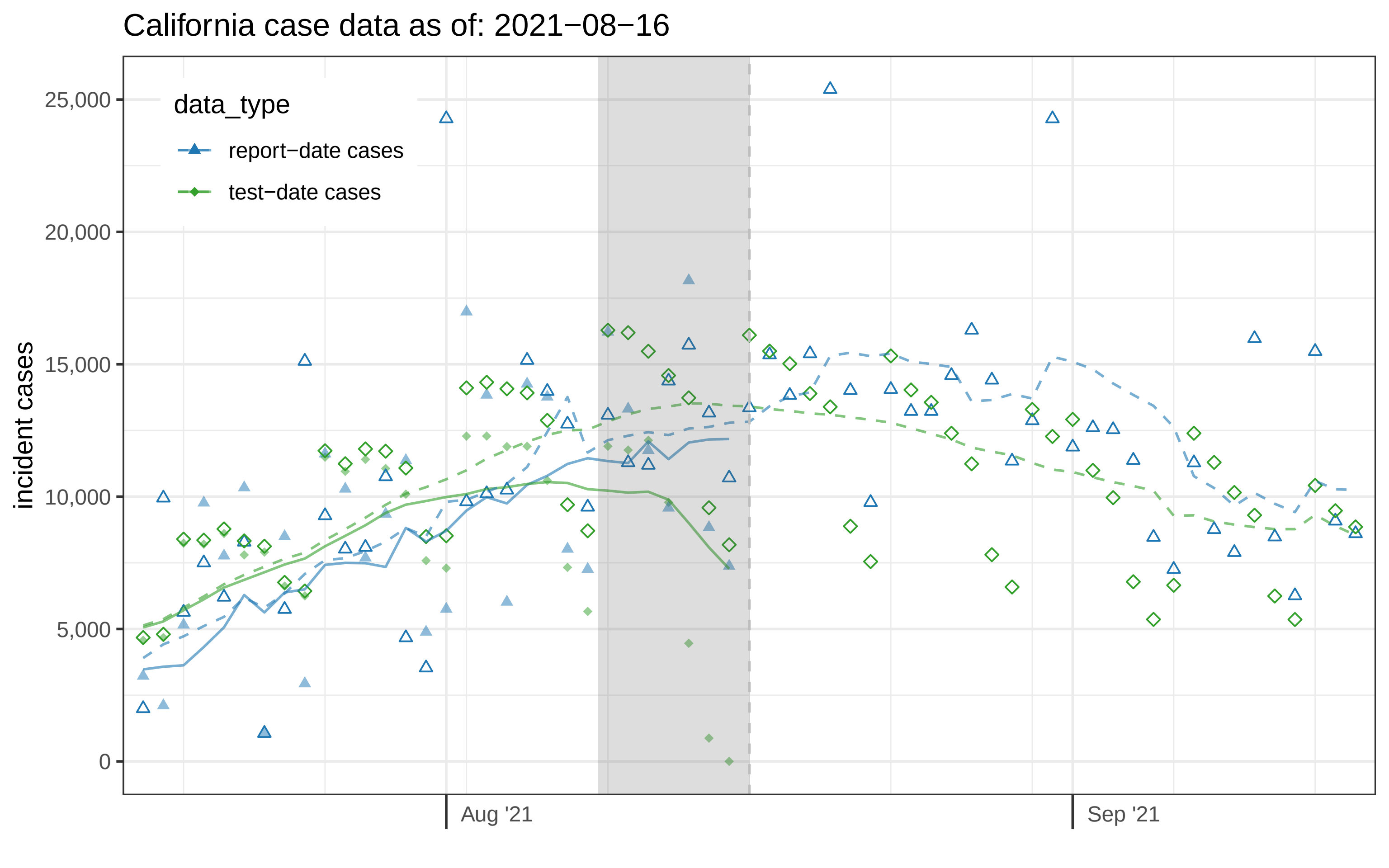

California case data as of: 2021−08−16
data_type
report−date cases
test−date cases
incident cases
25,000
20,000
15,000
10,000
5,000
0
Aug '21
Sep '21

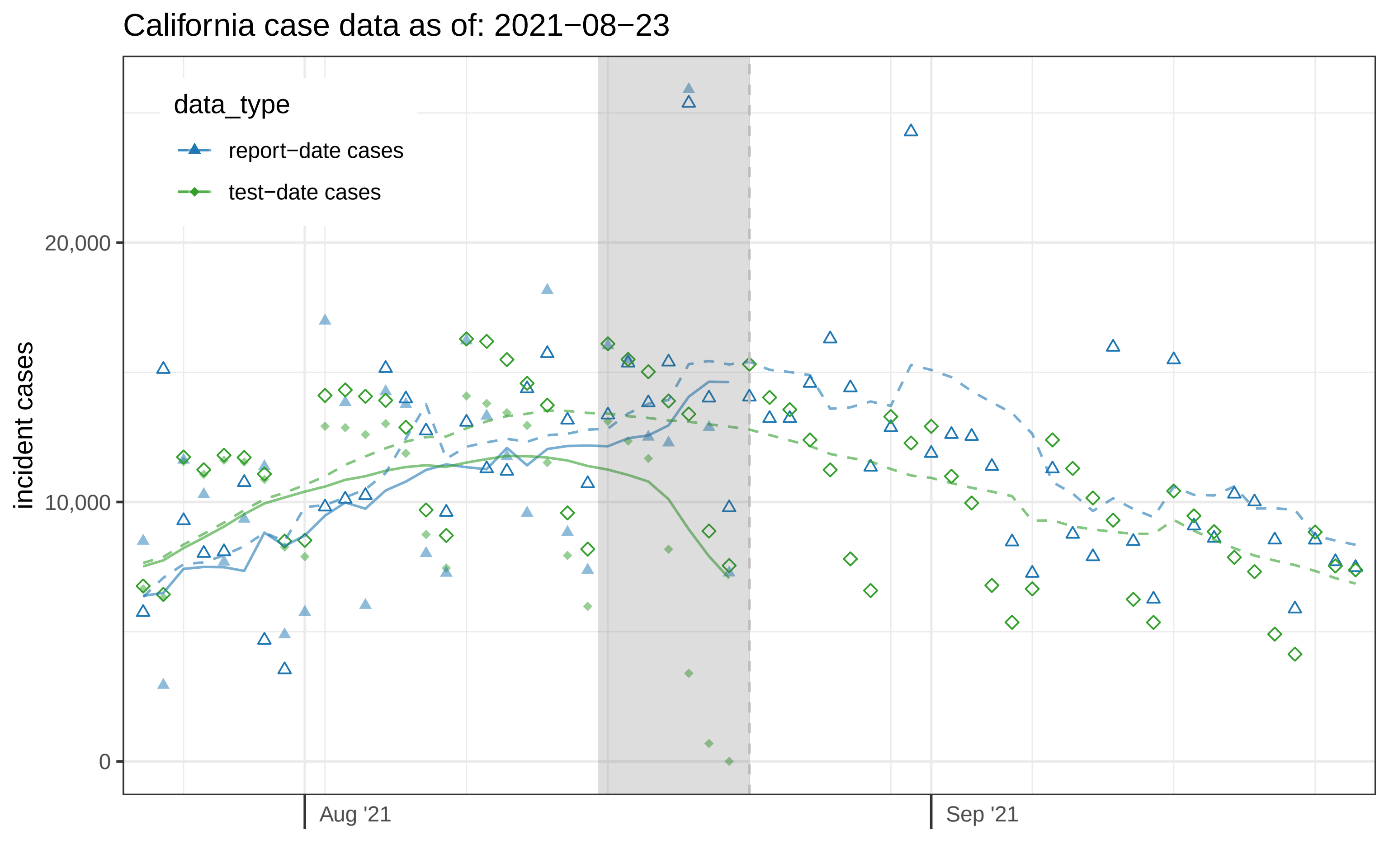
California case data as of: 2021−08−23
data_type
report−date cases
test−date cases
incident cases
20,000
10,000
0
Aug '21
Sep '21

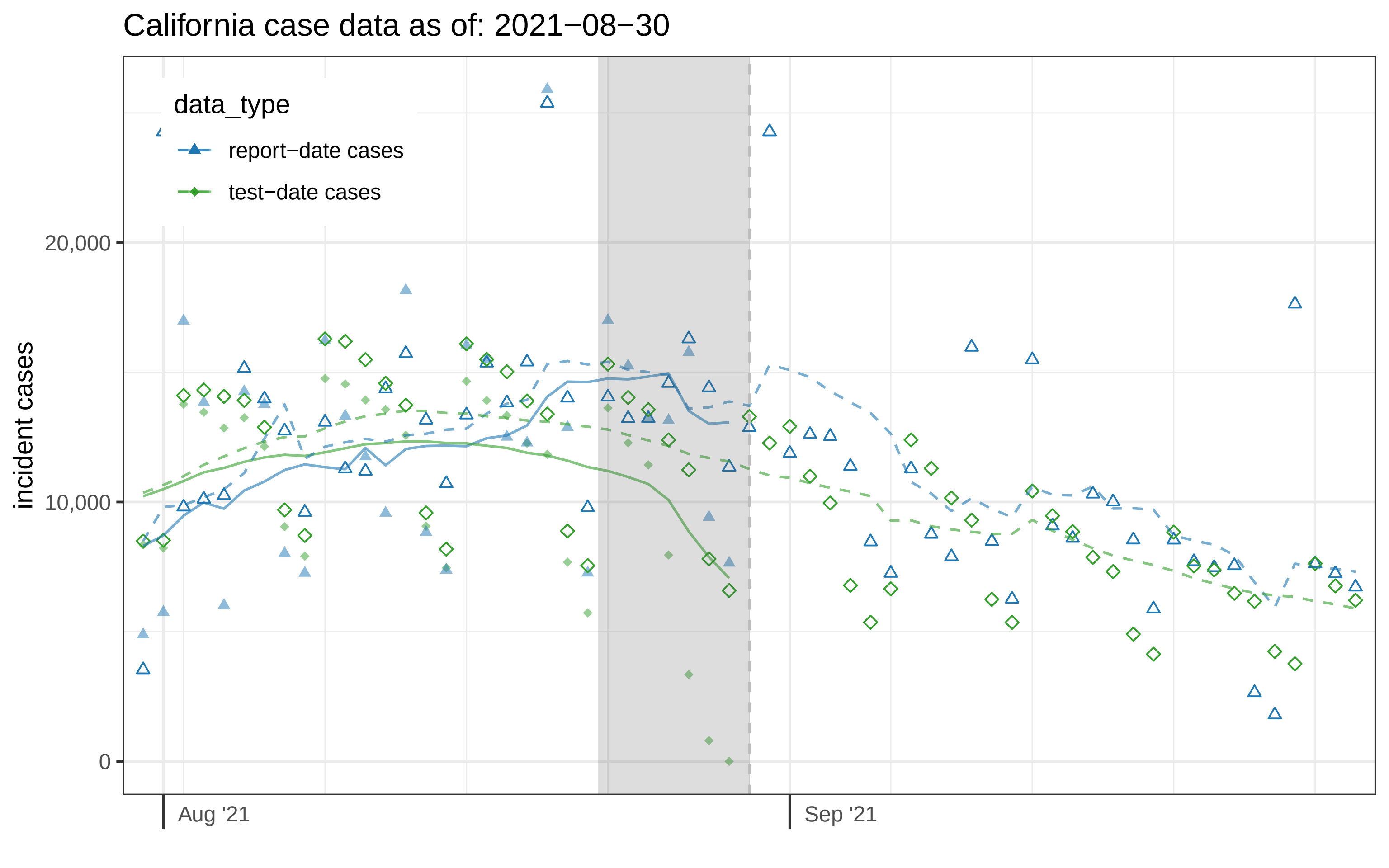
California case data as of: 2021−08−30
data_type
report−date cases
test−date cases
incident cases
20,000
10,000
0
Aug '21
Sep '21

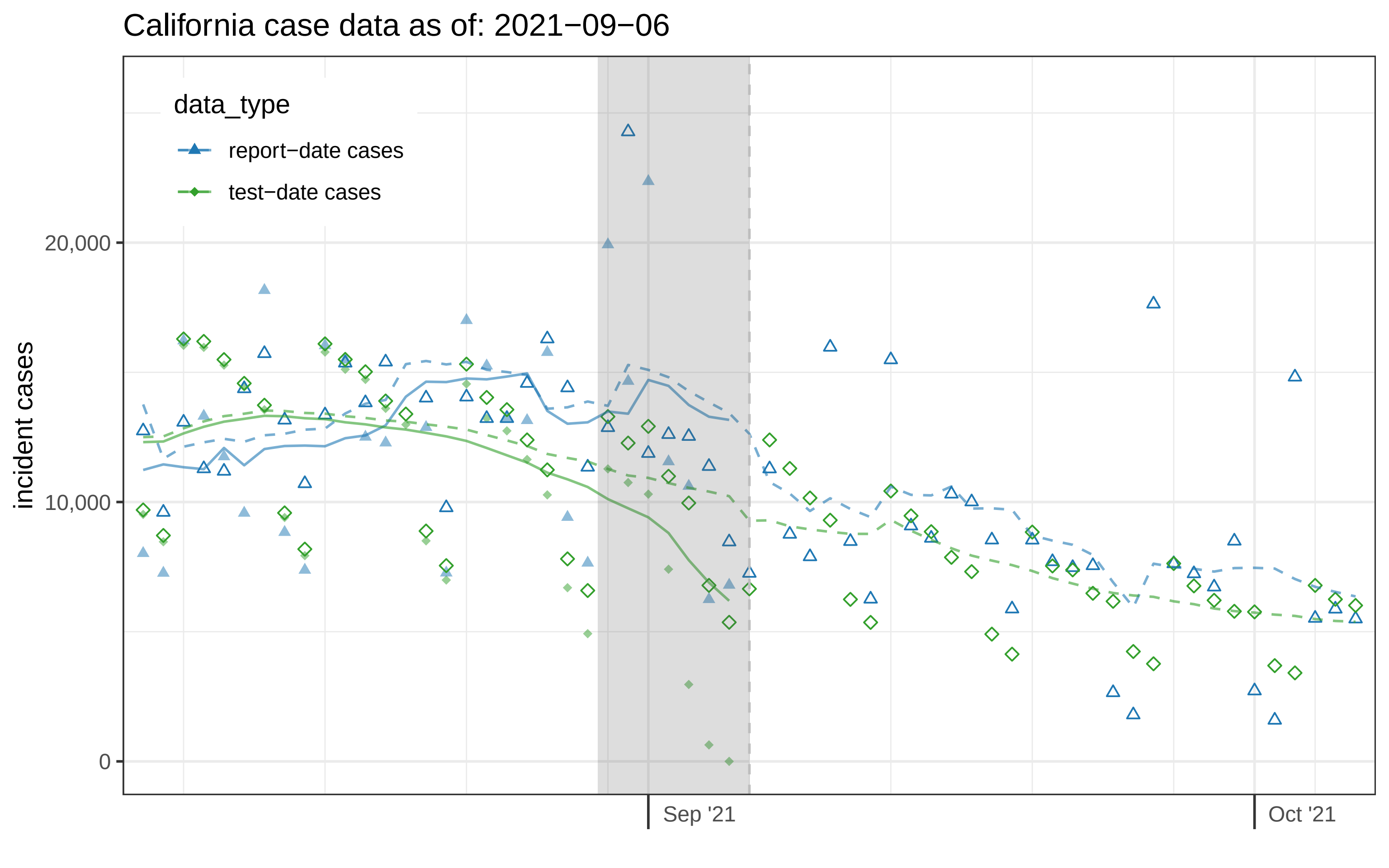
California case data as of: 2021−09−06
data_type
report−date cases
test−date cases
incident cases
20,000
10,000
0
Sep '21
Oct '21

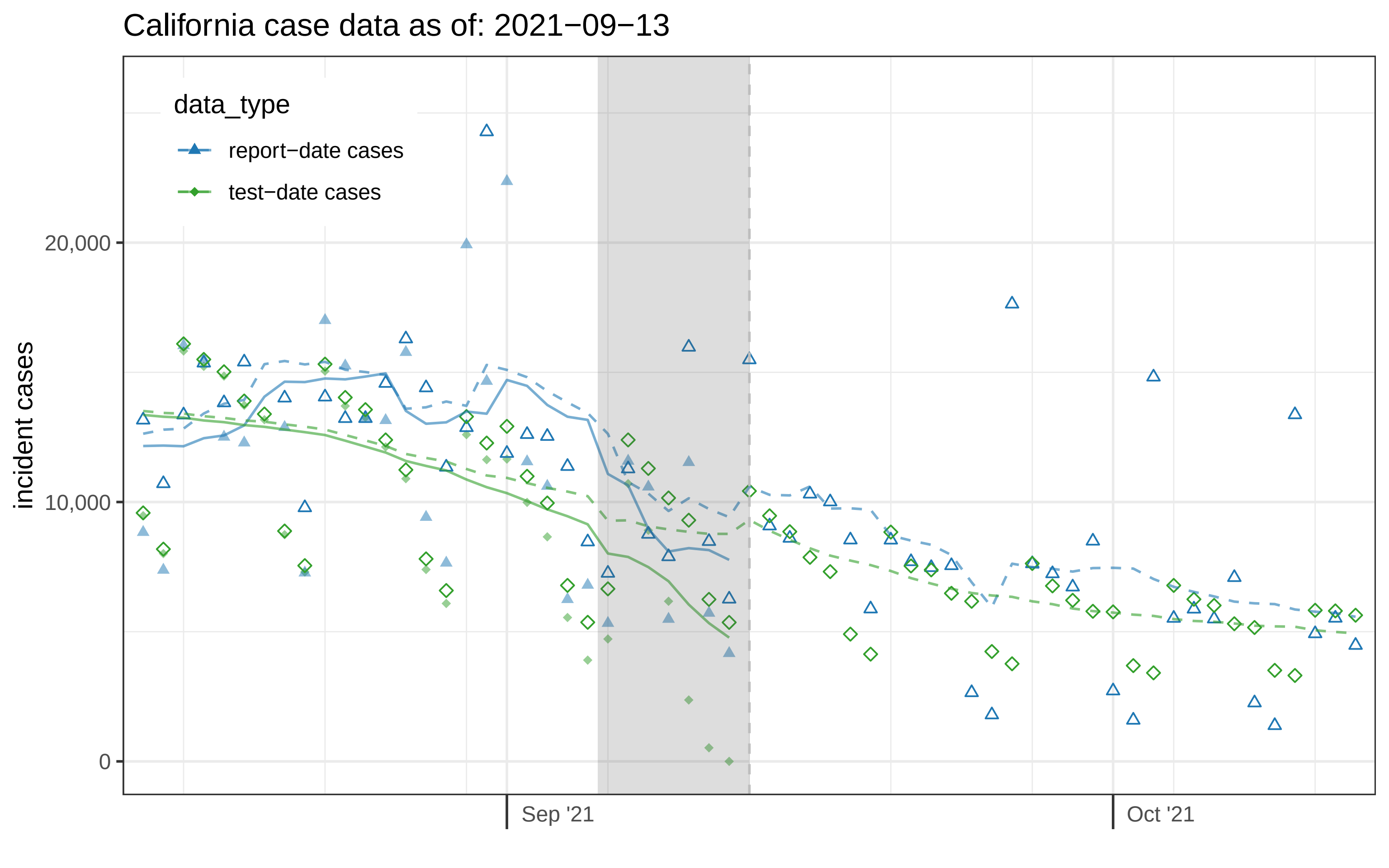
California case data as of: 2021−09−13
data_type
report−date cases
test−date cases
incident cases
20,000
10,000
0
Sep '21
Oct '21

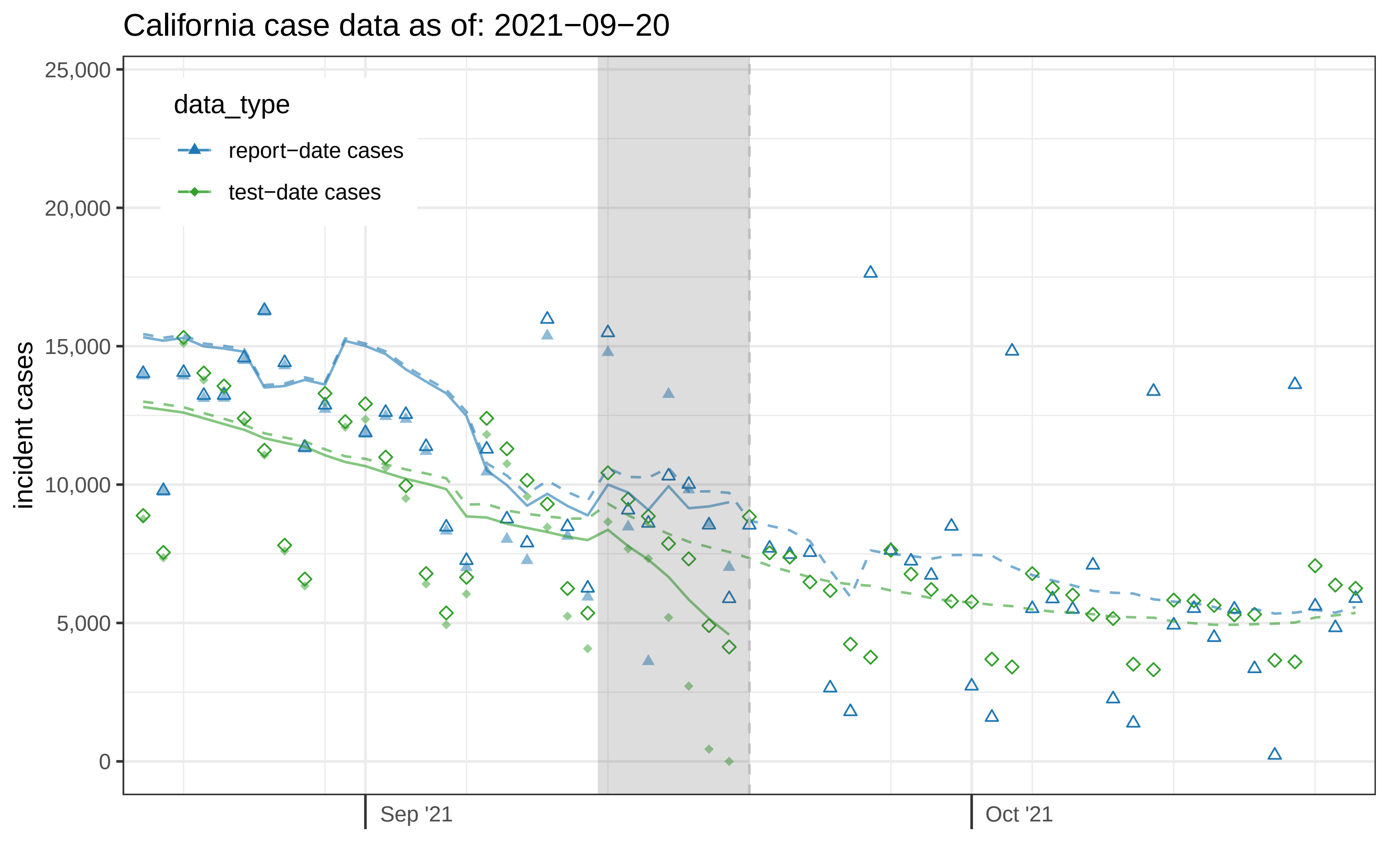
California case data as of: 2021−09−20
data_type
report−date cases
test−date cases
incident cases
25,000
20,000
15,000
10,000
5,000
0
Sep '21
Oct '21

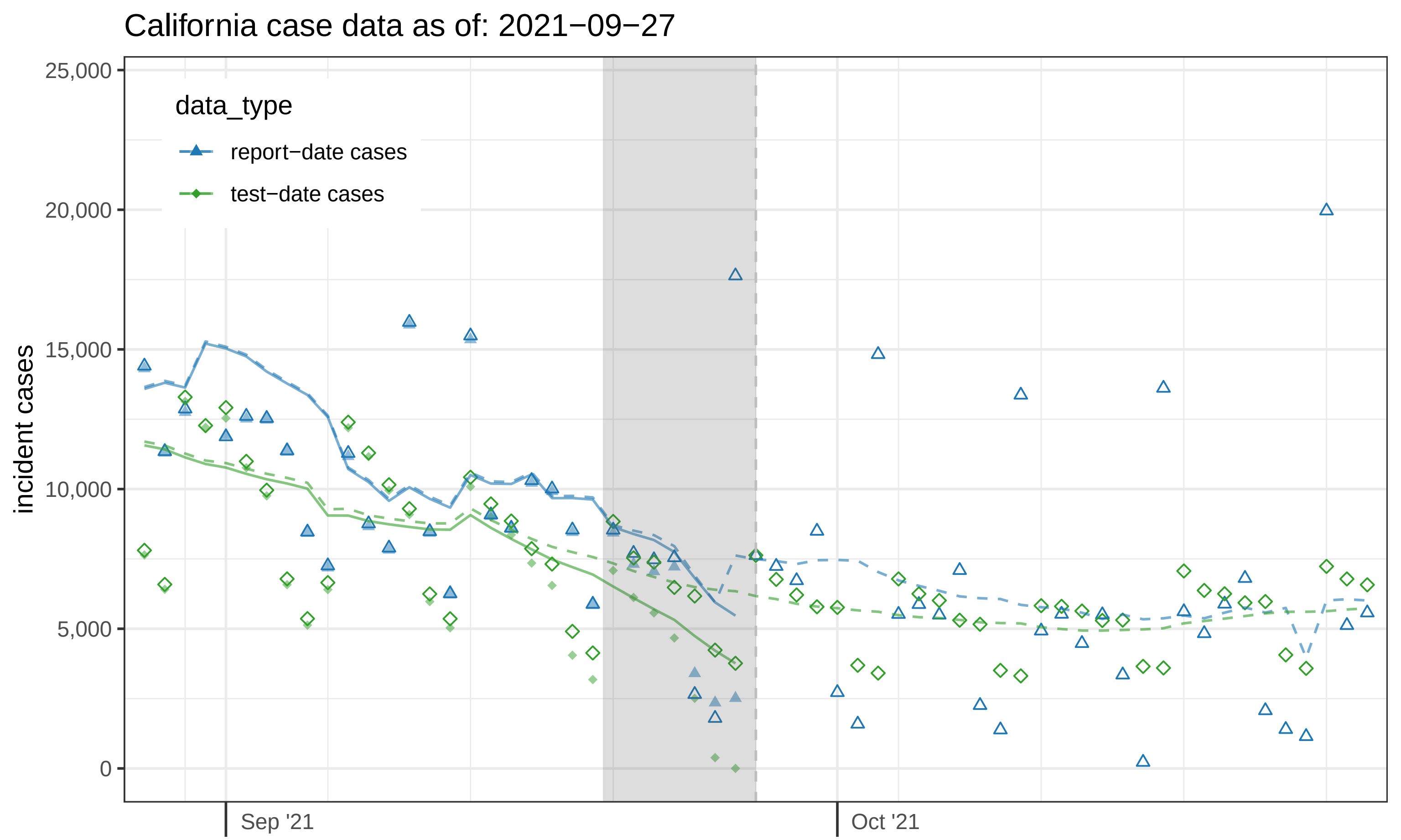
California case data as of: 2021−09−27
data_type
report−date cases
test−date cases
incident cases
25,000
20,000
15,000
10,000
5,000
0
Sep '21
Oct '21

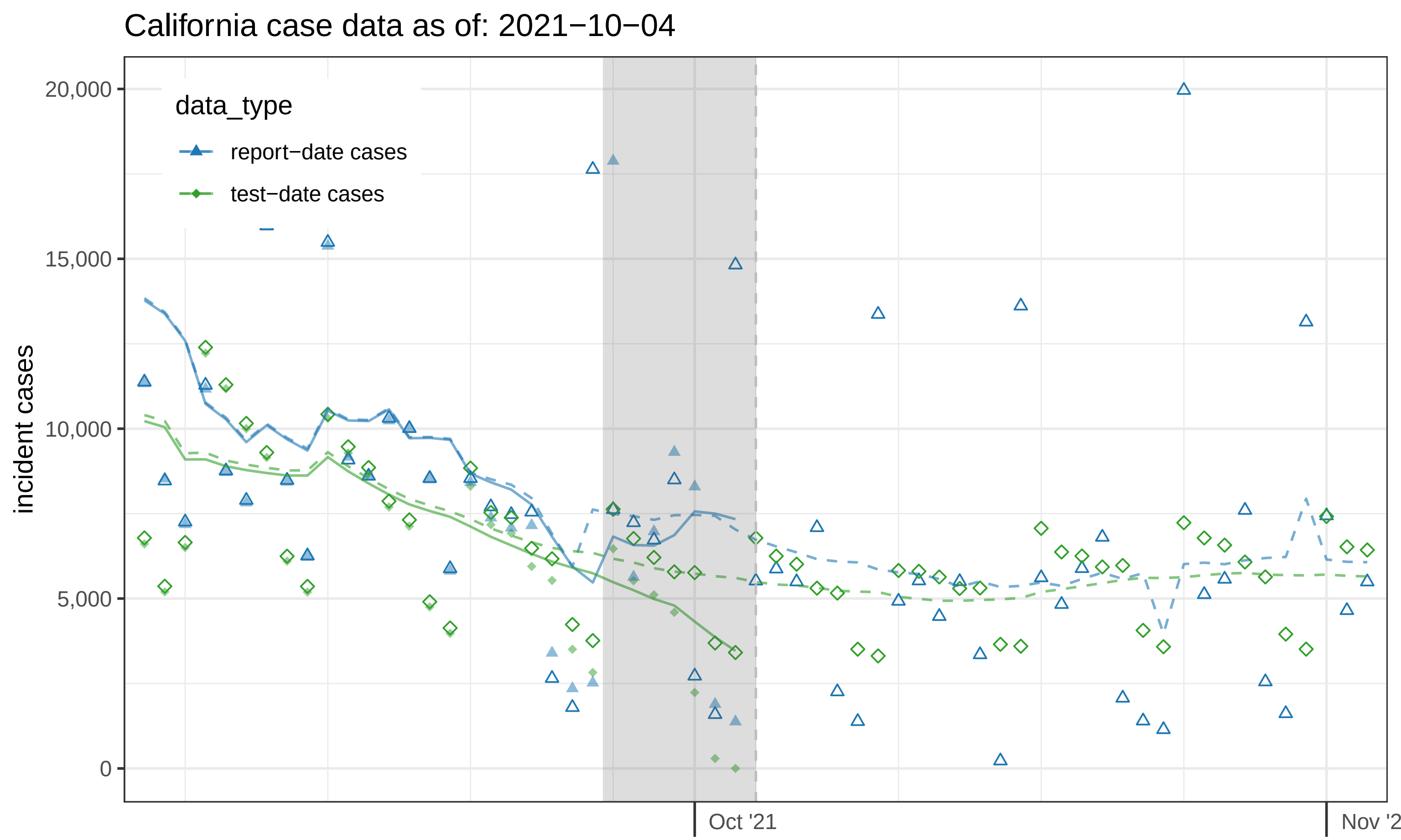

California case data as of: 2021−10−04
data_type
report−date cases
test−date cases
incident cases
20,000
15,000
10,000
5,000
0
Oct '21
Nov '2

California case data as of: 2021−10−11

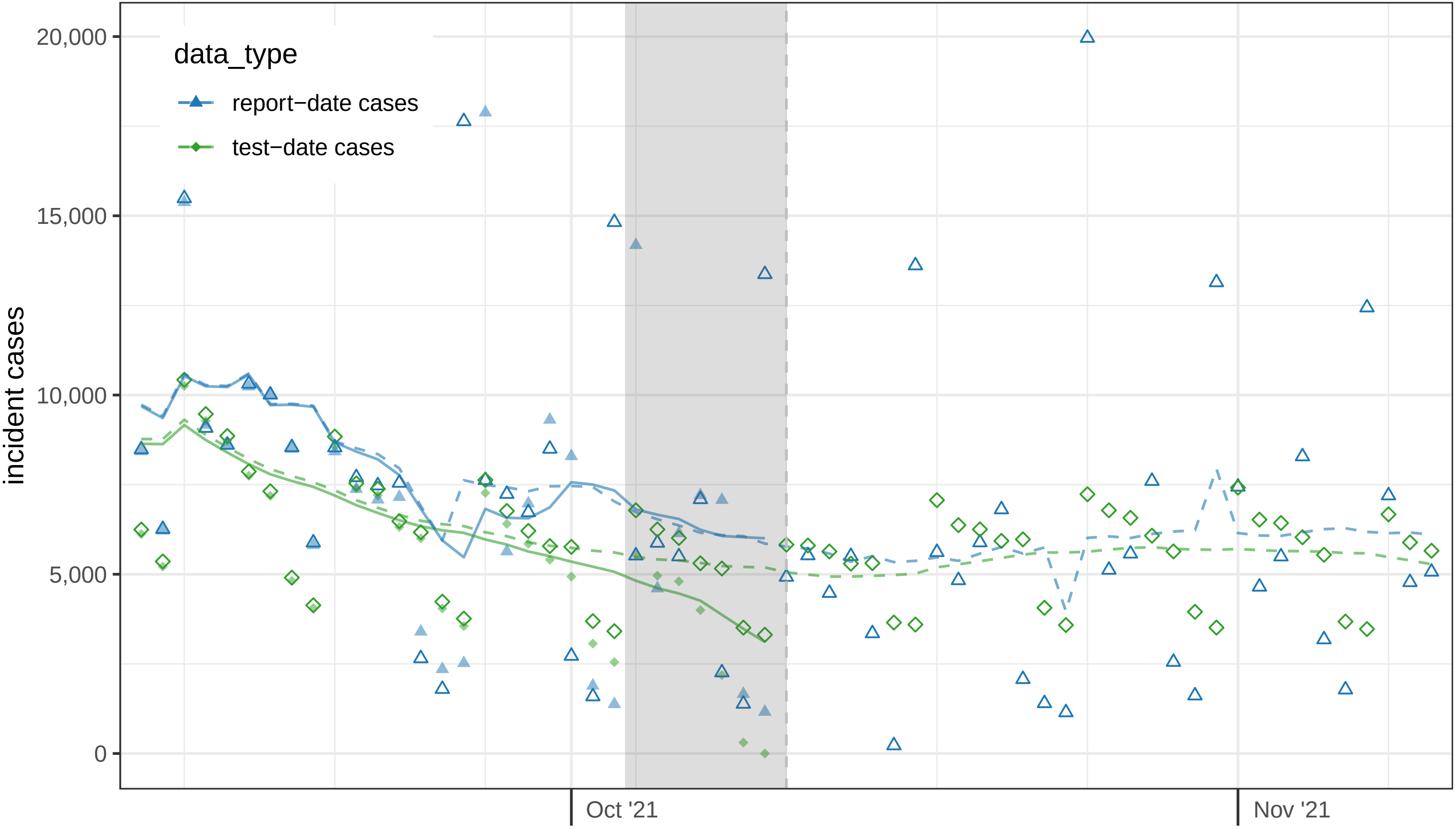

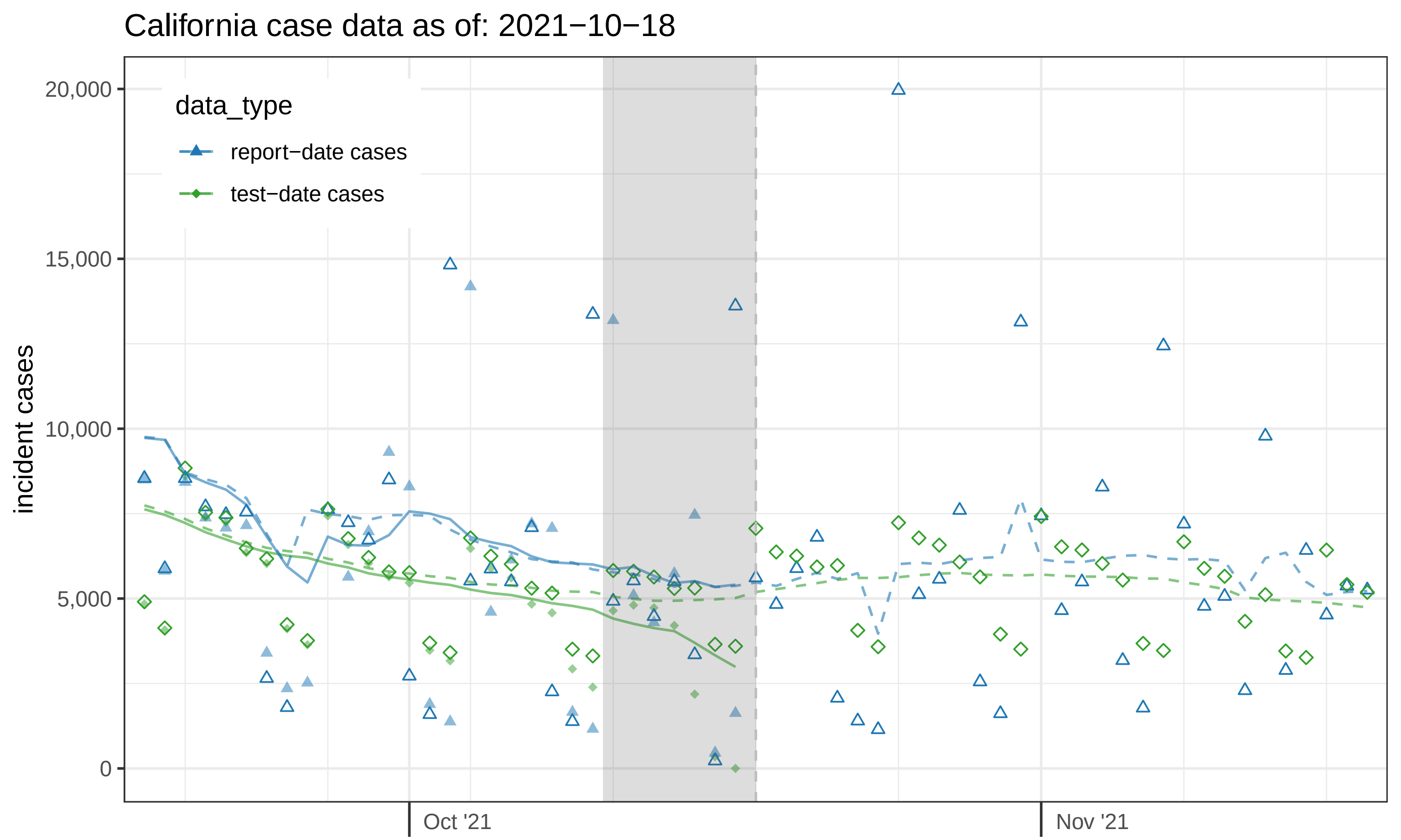

California case data as of: 2021−10−18
data_type
report−date cases
test−date cases
incident cases
20,000
15,000
10,000
5,000
0
Oct '21
Nov '21

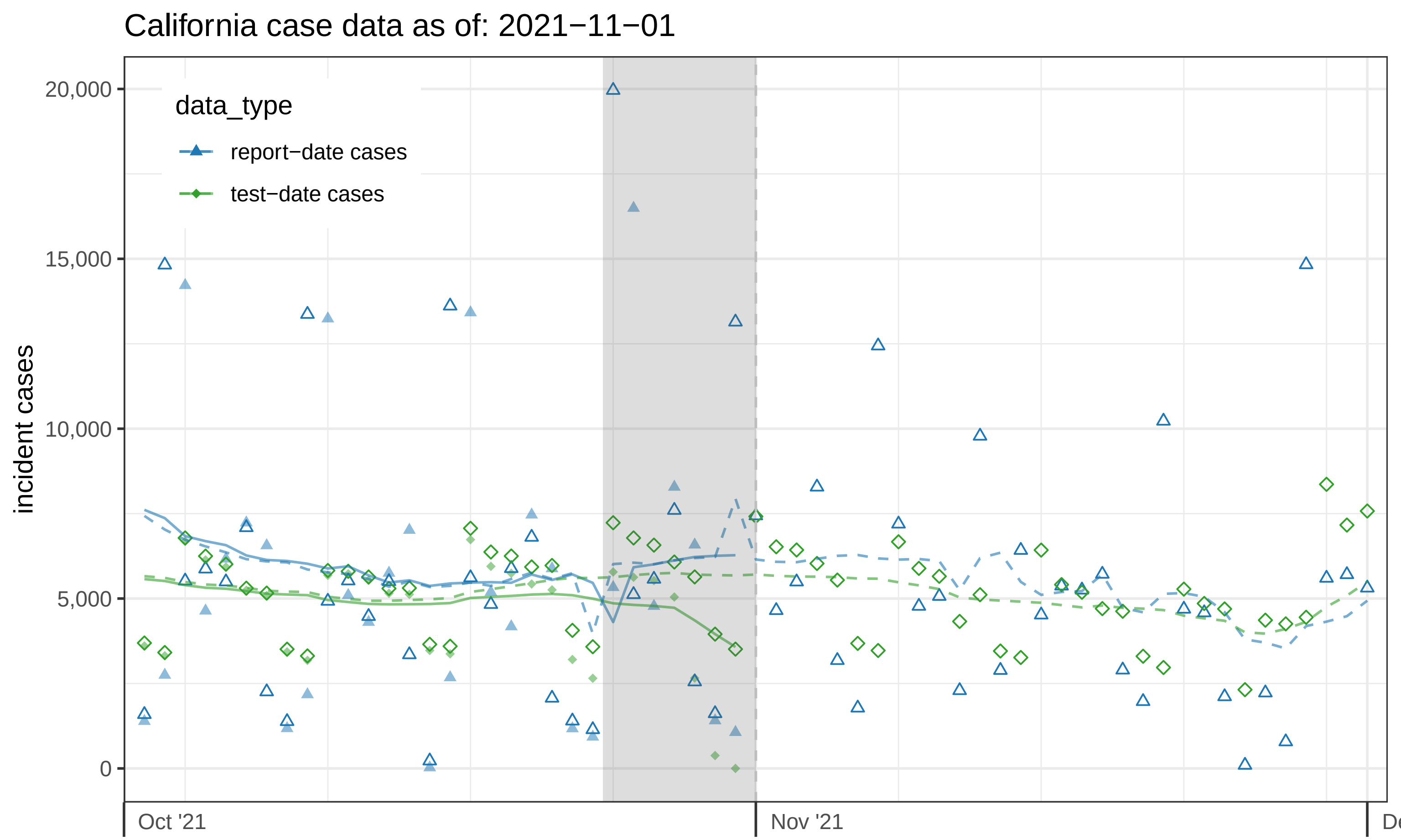

California case data as of: 2021−11−01
data_type
report−date cases
test−date cases
incident cases
20,000
15,000
10,000
5,000
0
Oct '21
Nov '21

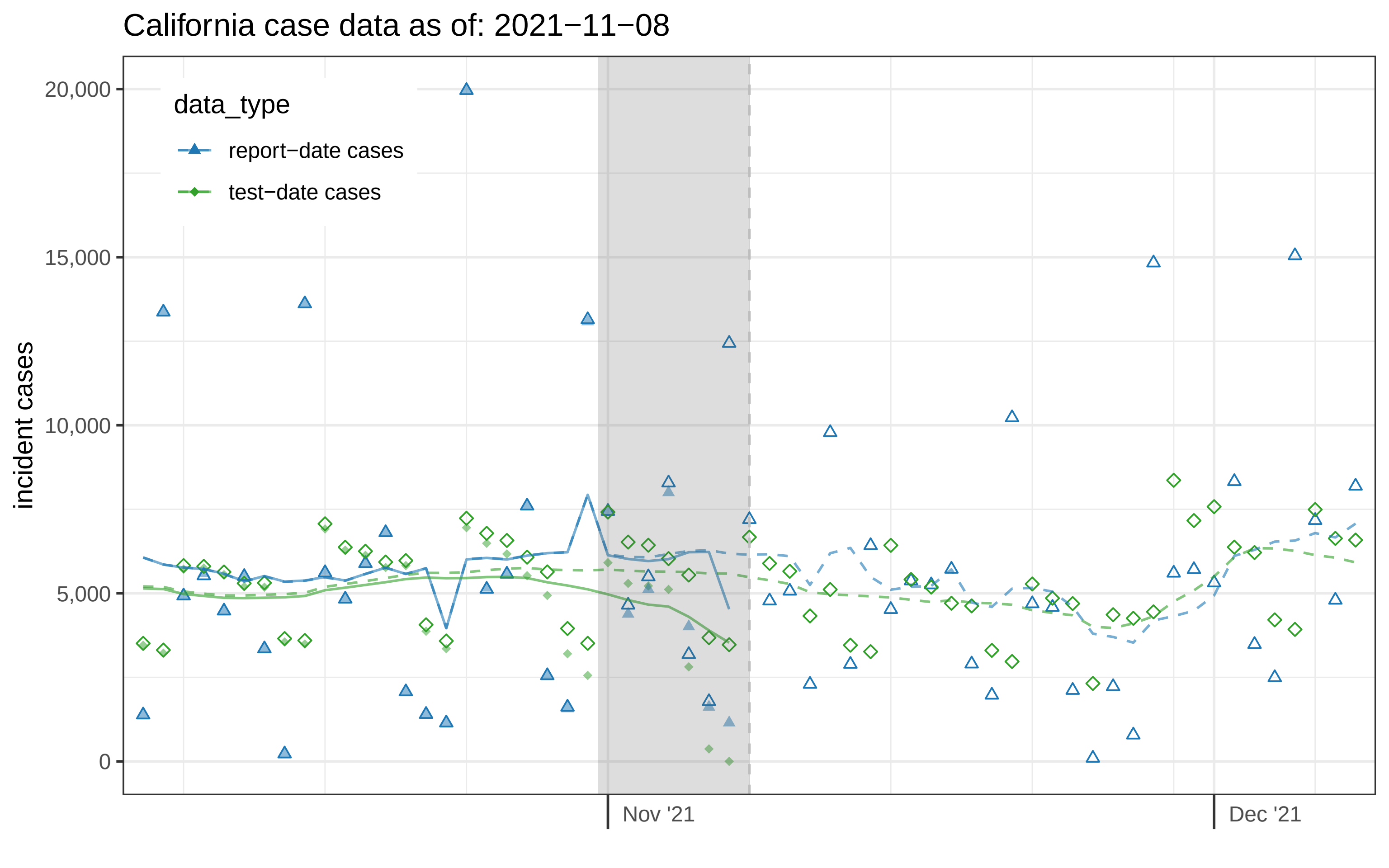

California case data as of: 2021−11−08
data_type
report−date cases
test−date cases
incident cases
20,000
15,000
10,000
5,000
0
Nov '21
Dec '21

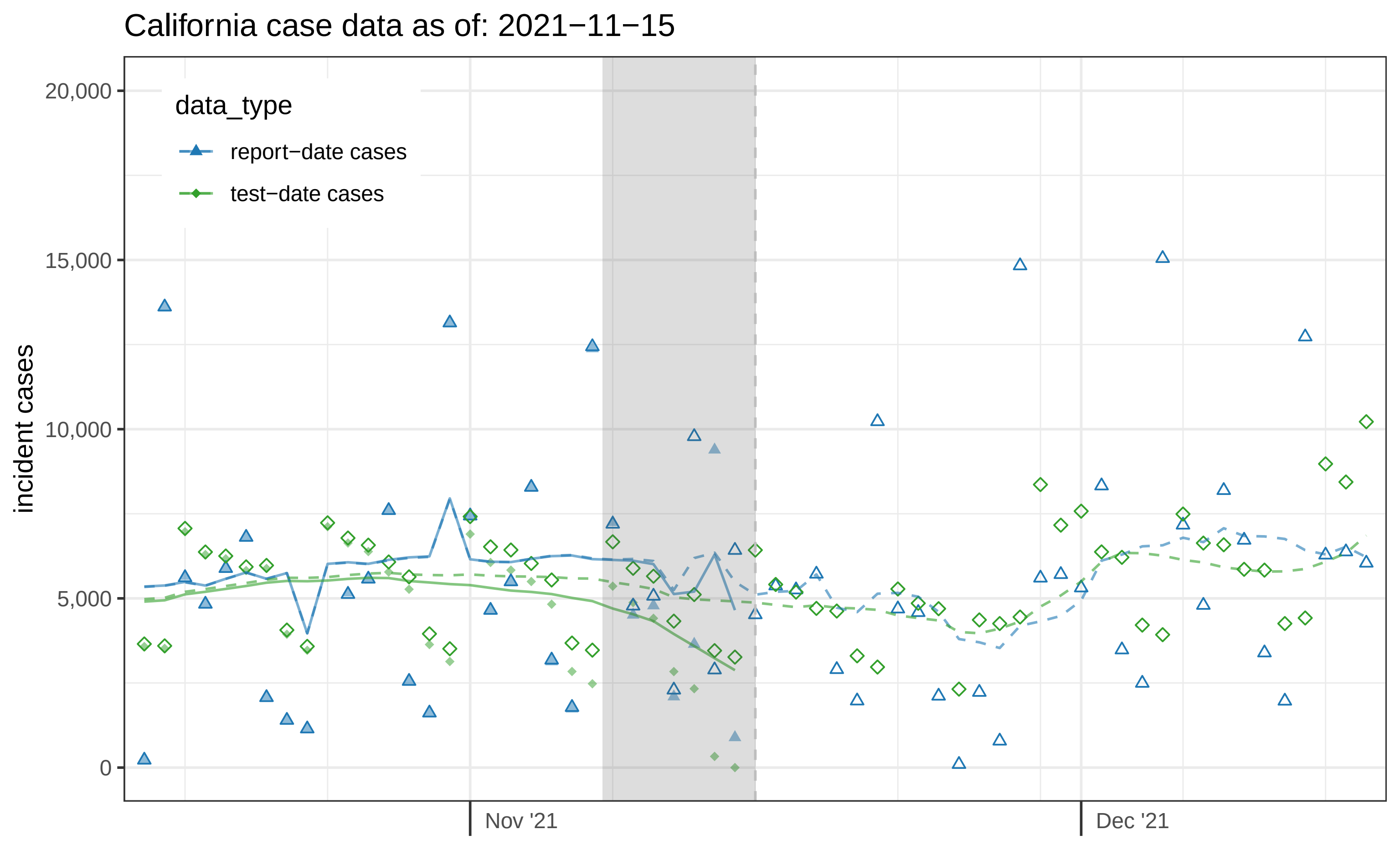
California case data as of: 2021−11−15
data_type
report−date cases
test−date cases
incident cases
20,000
15,000
10,000
5,000
0
Nov '21
Dec '21

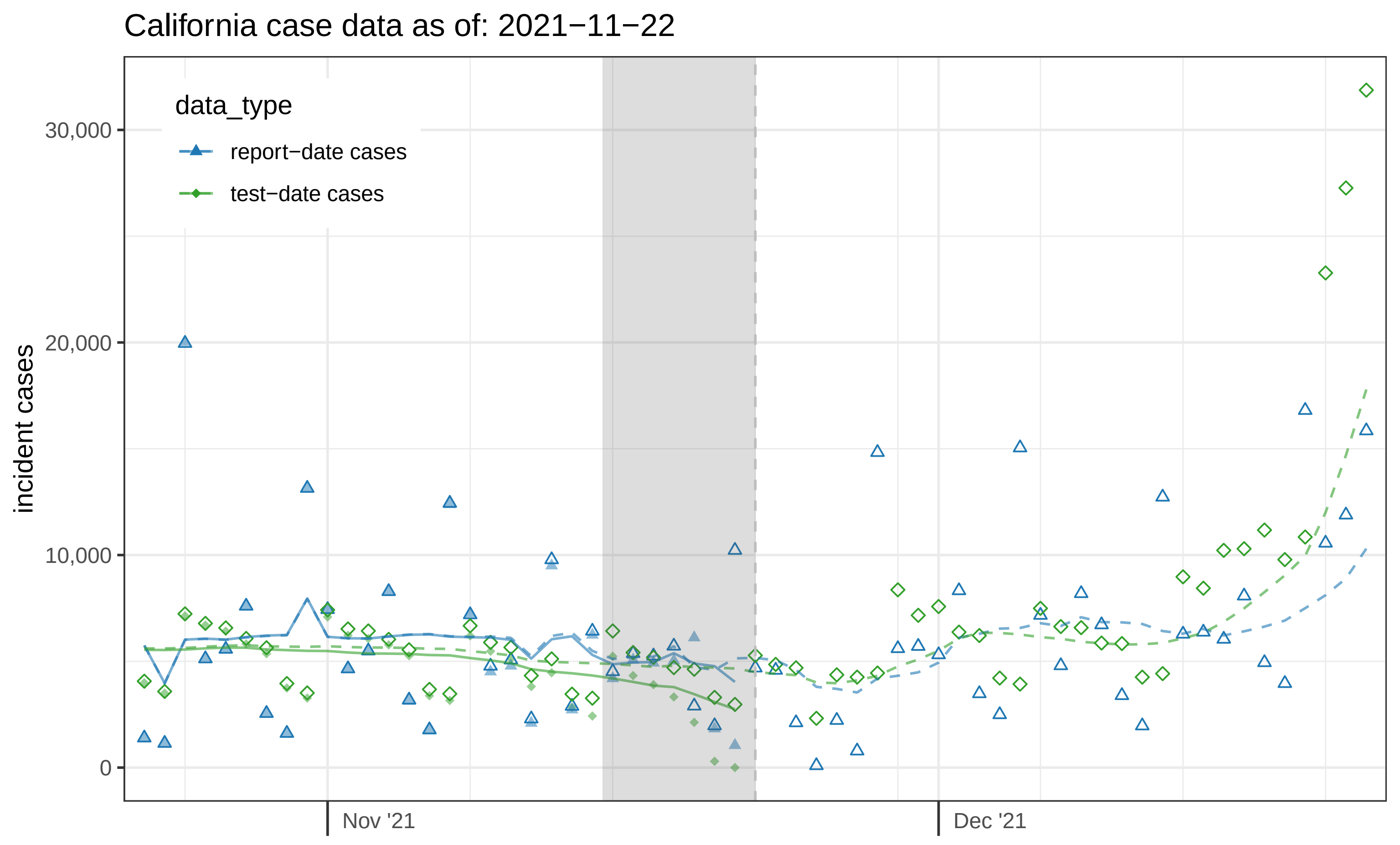

California case data as of: 2021−11−22
data_type
report−date cases
test−date cases
incident cases
30,000
20,000
10,000
0
Nov '21
Dec '21

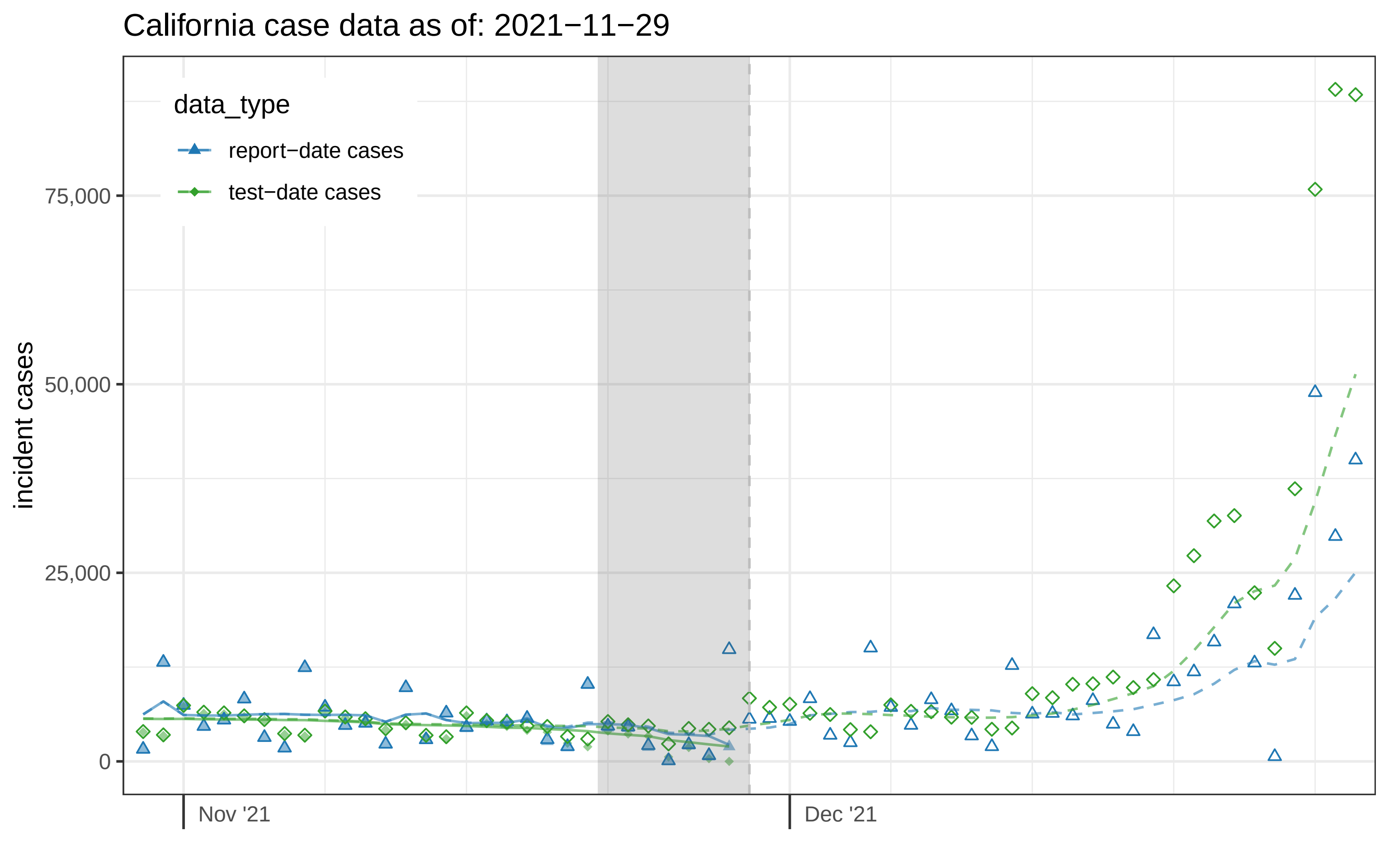
California case data as of: 2021−11−29
data_type
report−date cases
test−date cases
incident cases
75,000
50,000
25,000
0
Nov '21
Dec '21

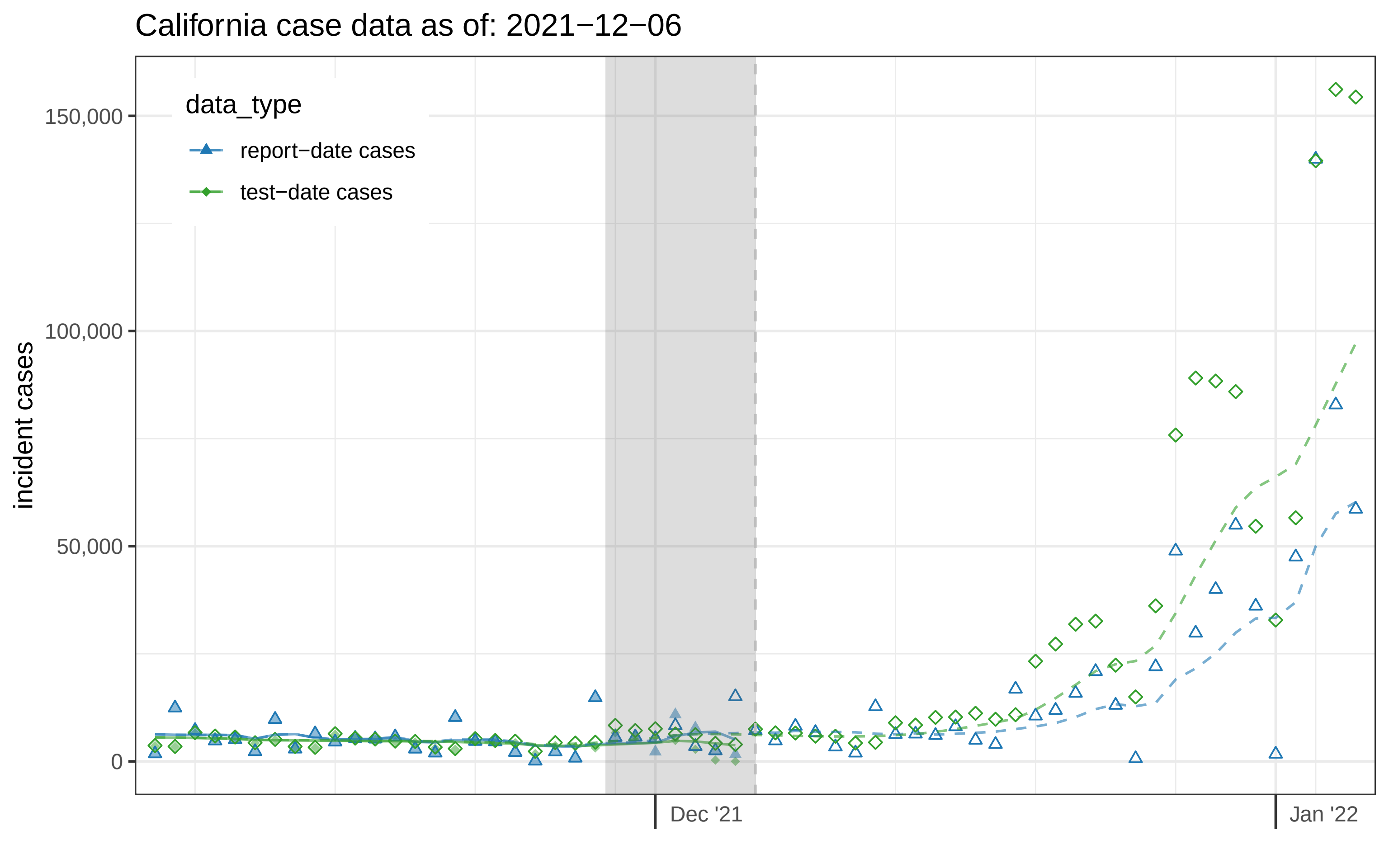

California case data as of: 2021−12−06
data_type
report−date cases
test−date cases
150,000
100,000
50,000
0
incident cases
Dec '21
Jan '22

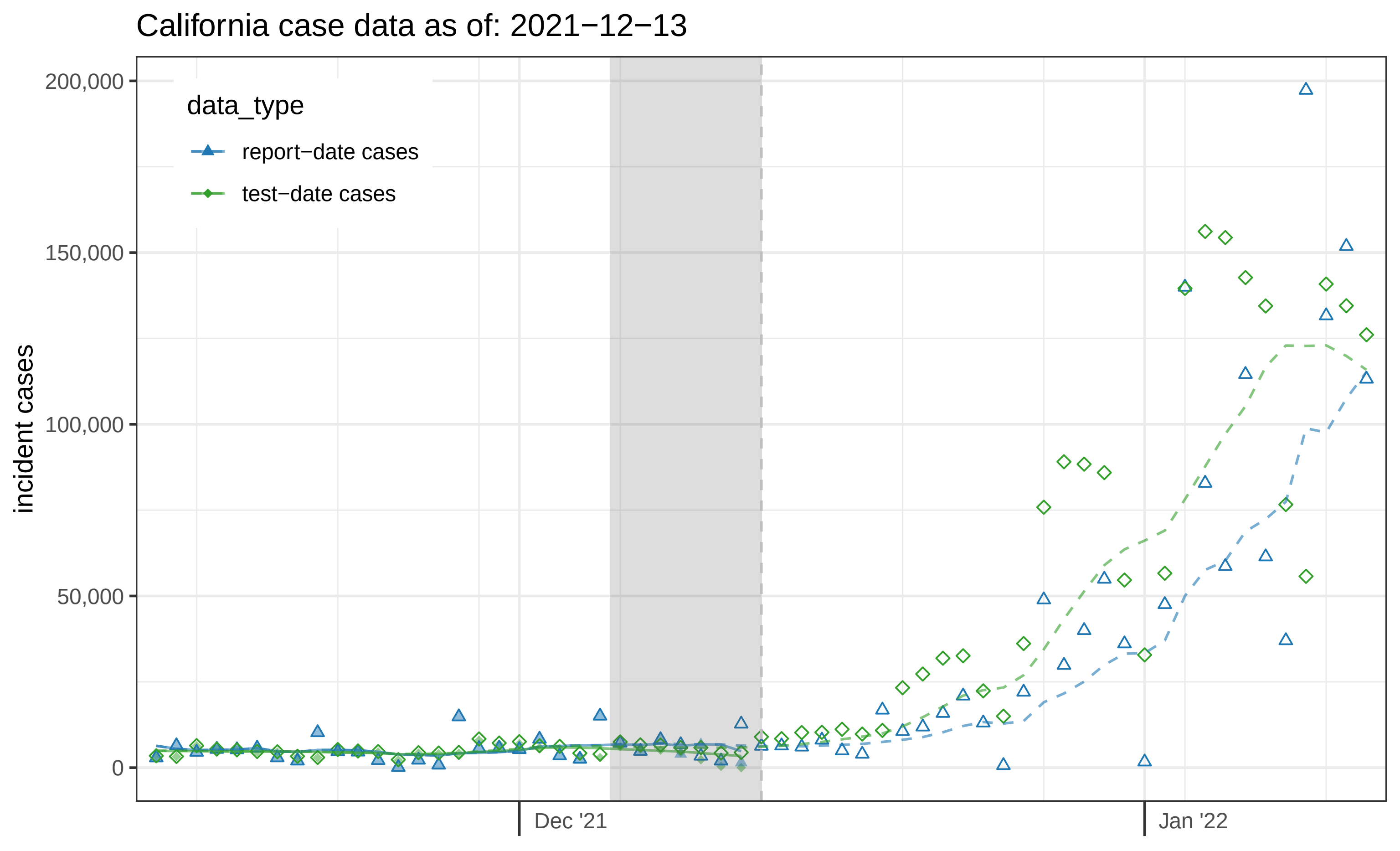

California case data as of: 2021−12−13
data_type
report−date cases
test−date cases
incident cases
200,000
150,000
100,000
50,000
0
Dec '21
Jan '22

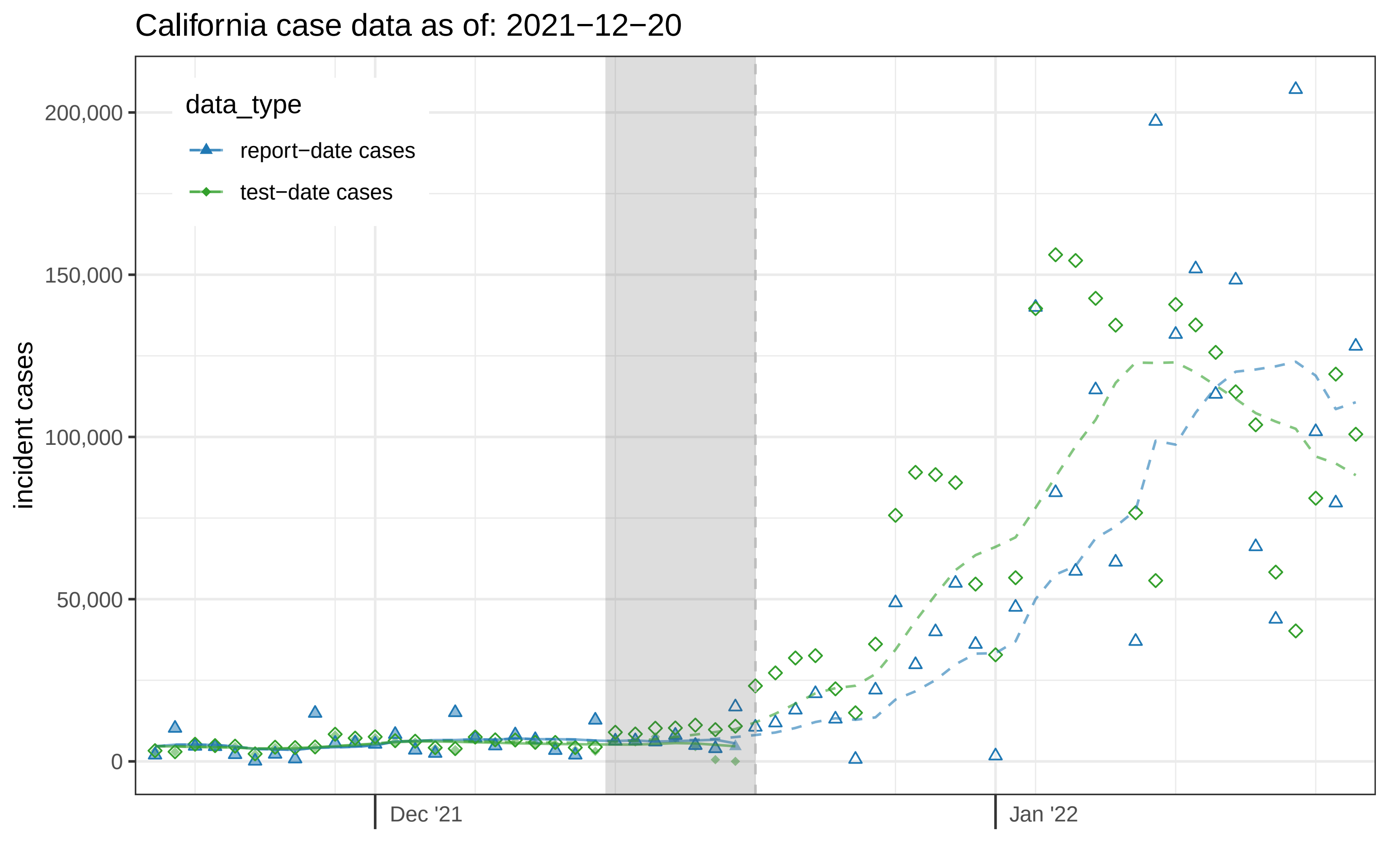
California case data as of: 2021−12−20
data_type
report−date cases
test−date cases
incident cases
200,000
150,000
100,000
50,000
0
Dec '21
Jan '22

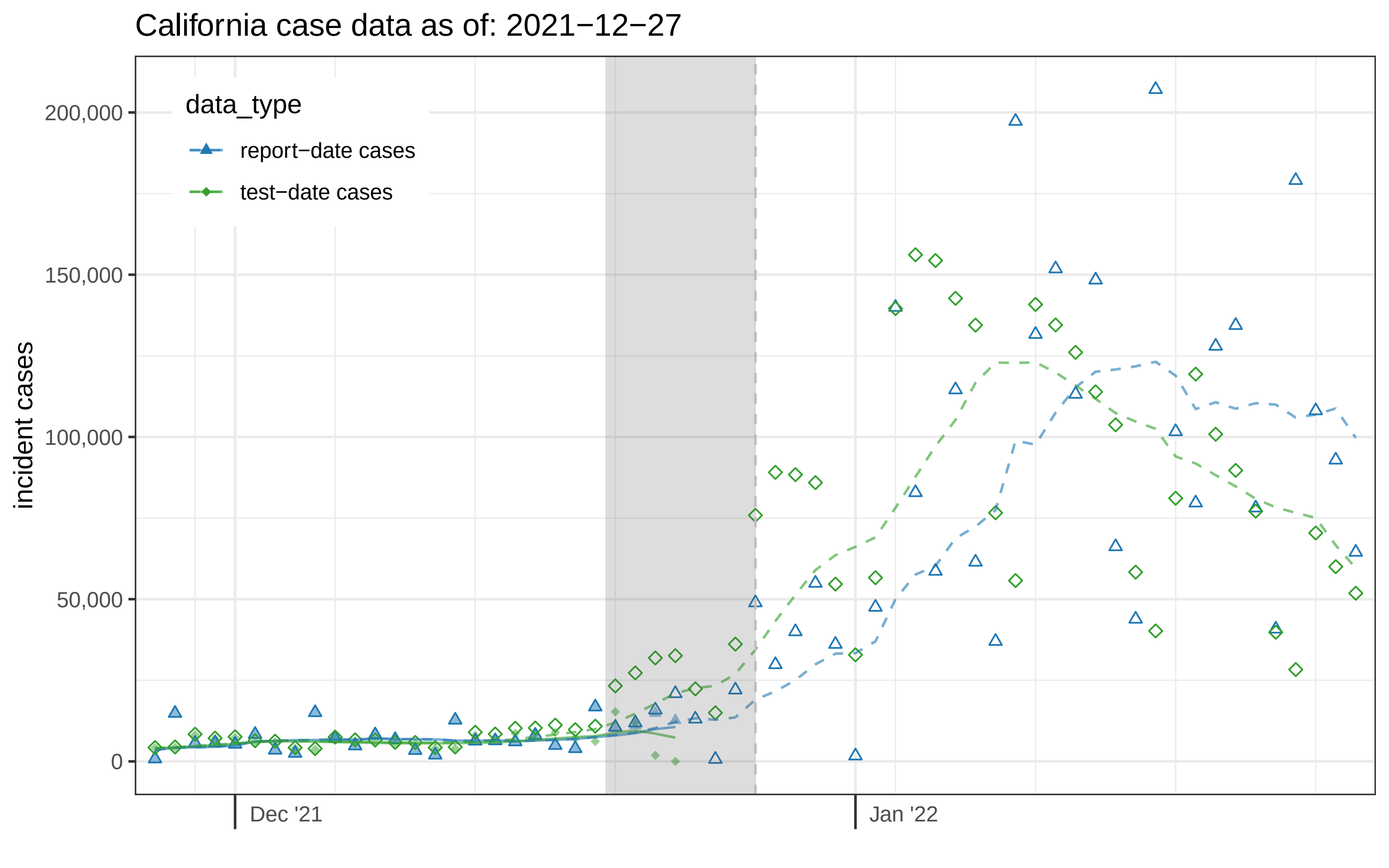
California case data as of: 2021−12−27
data_type
report−date cases
test−date cases
incident cases
200,000
150,000
100,000
50,000
0
Dec '21
Jan '22

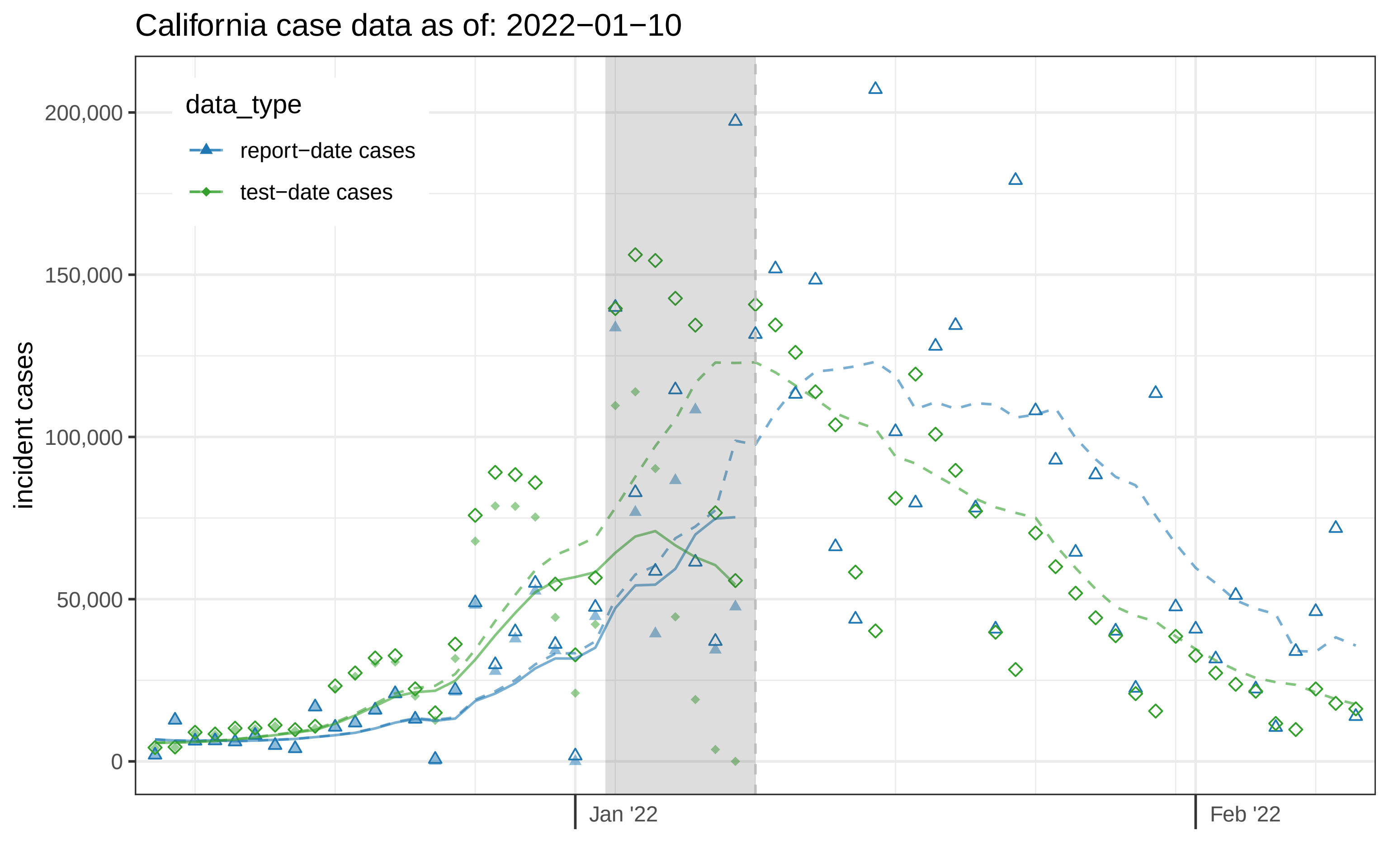
California case data as of: 2022−01−10
data_type
report−date cases
test−date cases
incident cases
200,000
150,000
100,000
50,000
0
Jan '22
Feb '22

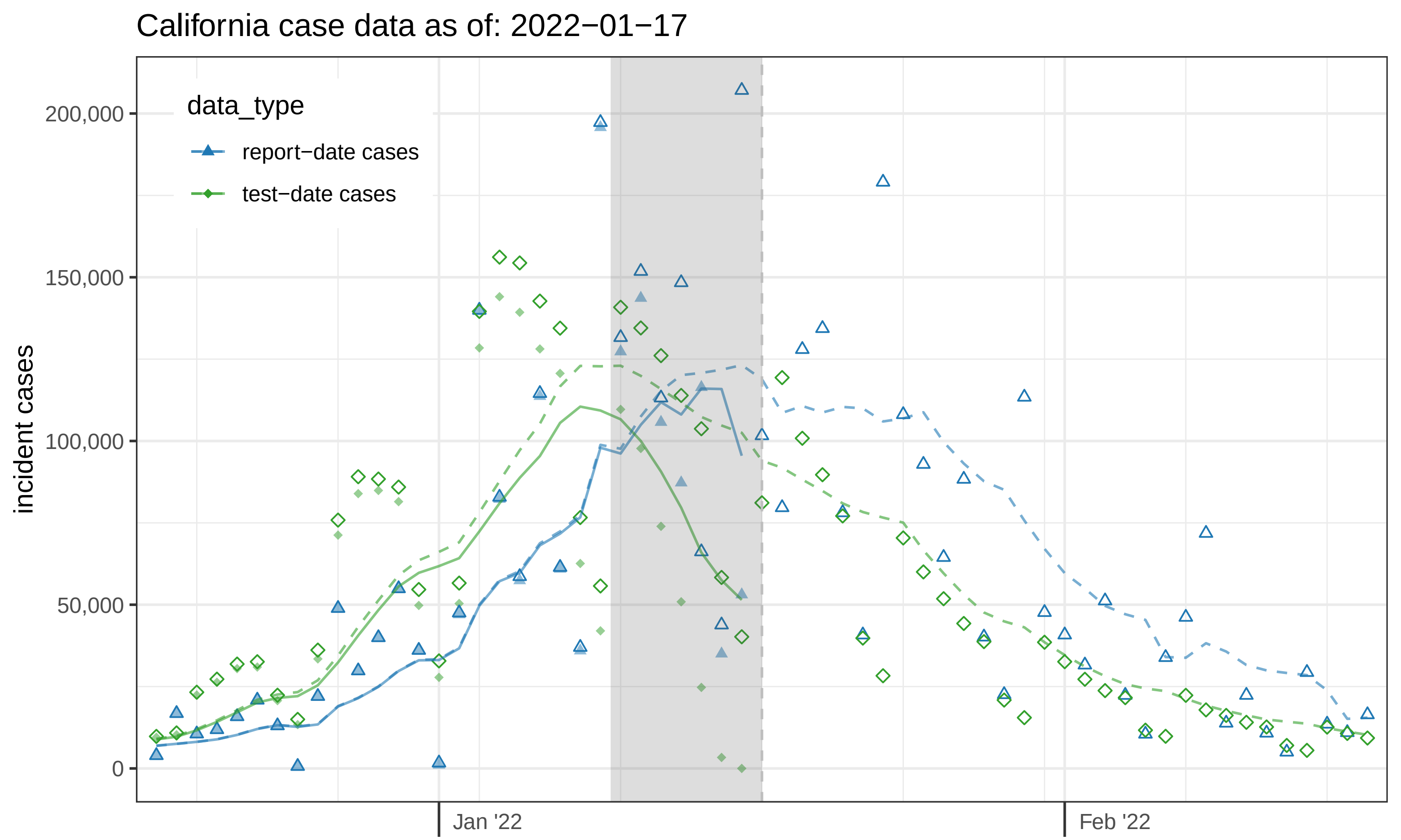
California case data as of: 2022−01−17
data_type
report−date cases
test−date cases
incident cases
200,000
150,000
100,000
50,000
0
Jan '22
Feb '22

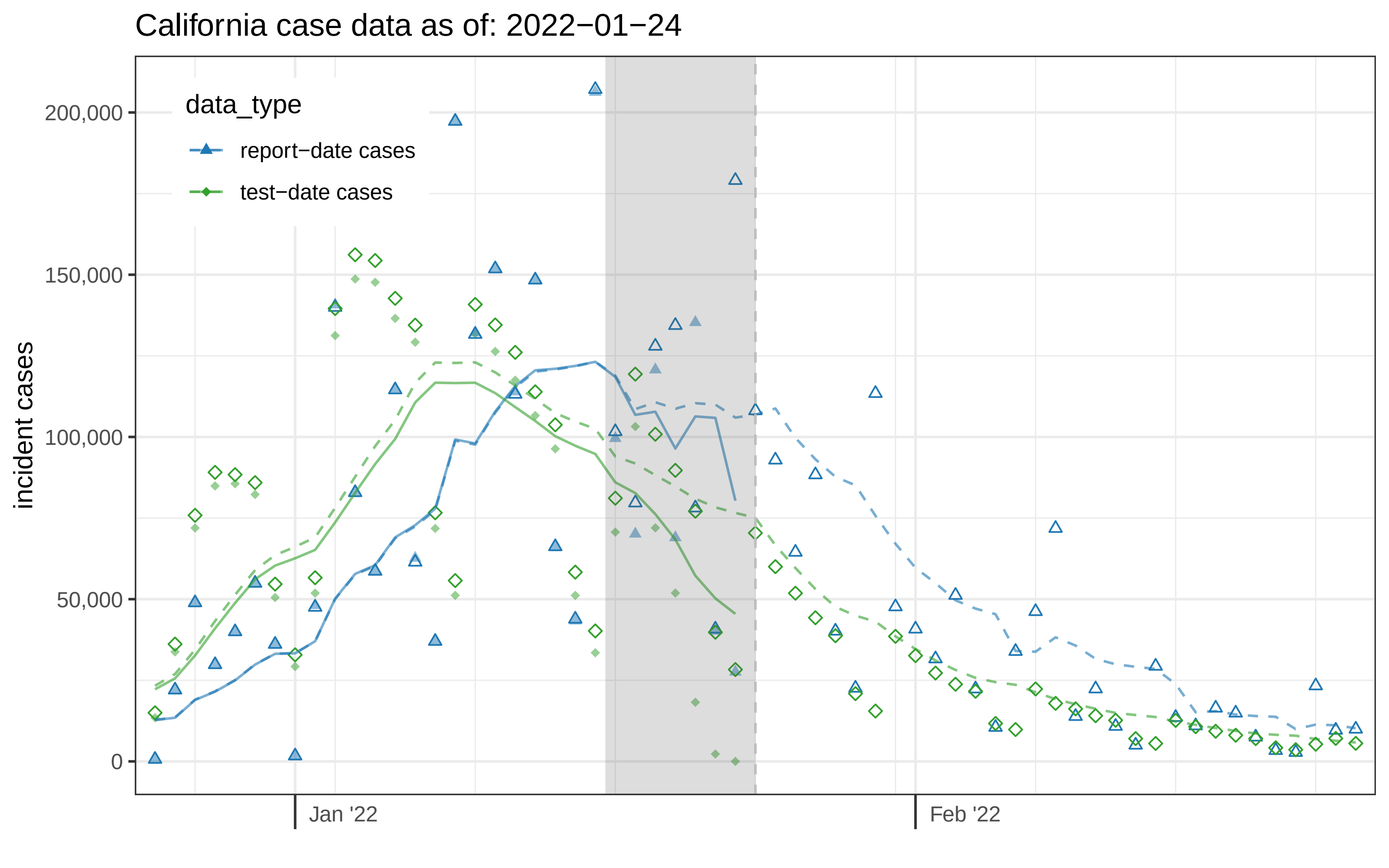

California case data as of: 2022−01−24
data_type
report−date cases
test−date cases
incident cases
200,000
150,000
100,000
50,000
0
Jan '22
Feb '22

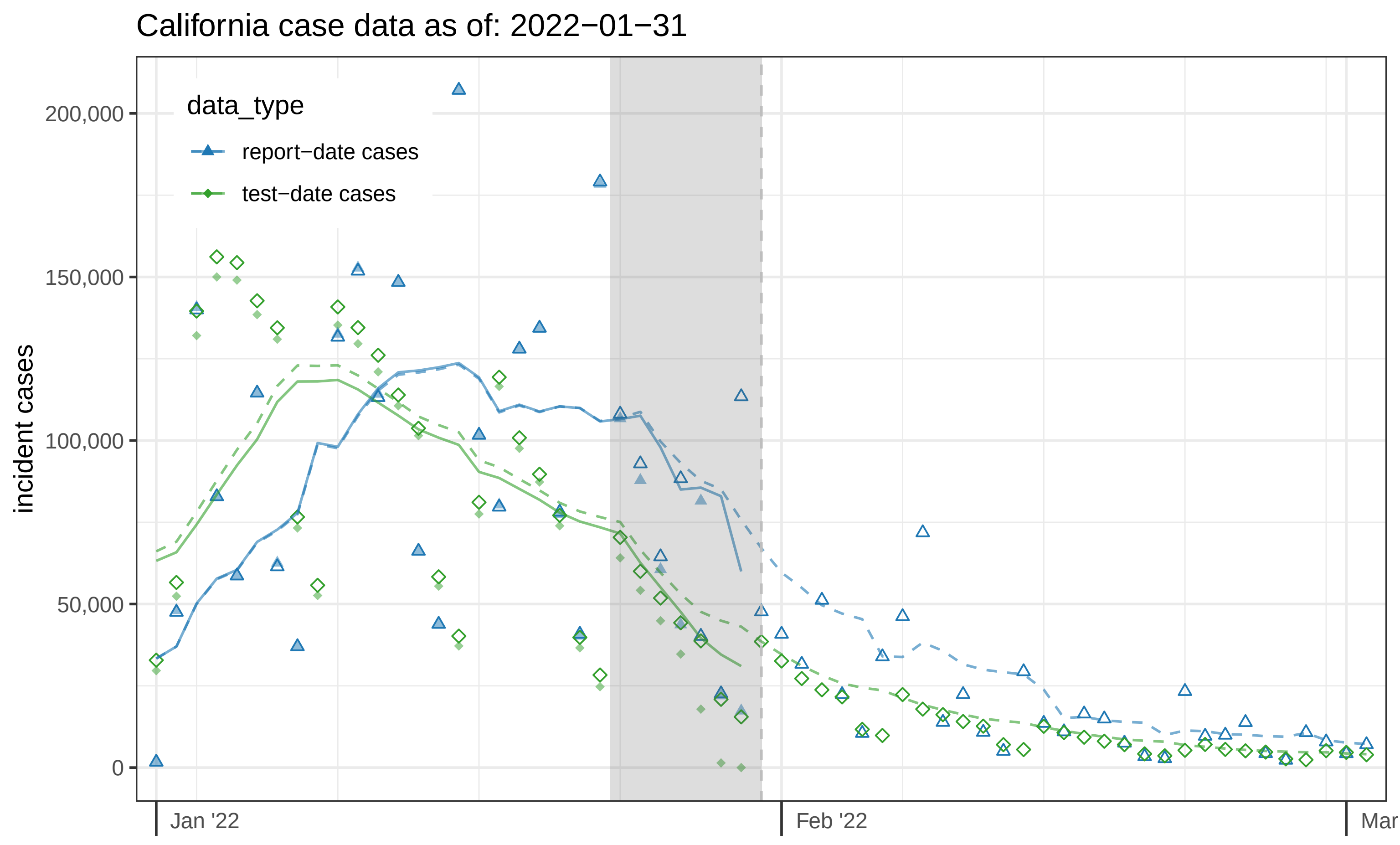
California case data as of: 2022−01−31
data_type
report−date cases
test−date cases
incident cases
200,000
150,000
100,000
50,000
0
Jan '22
Feb '22
Mar

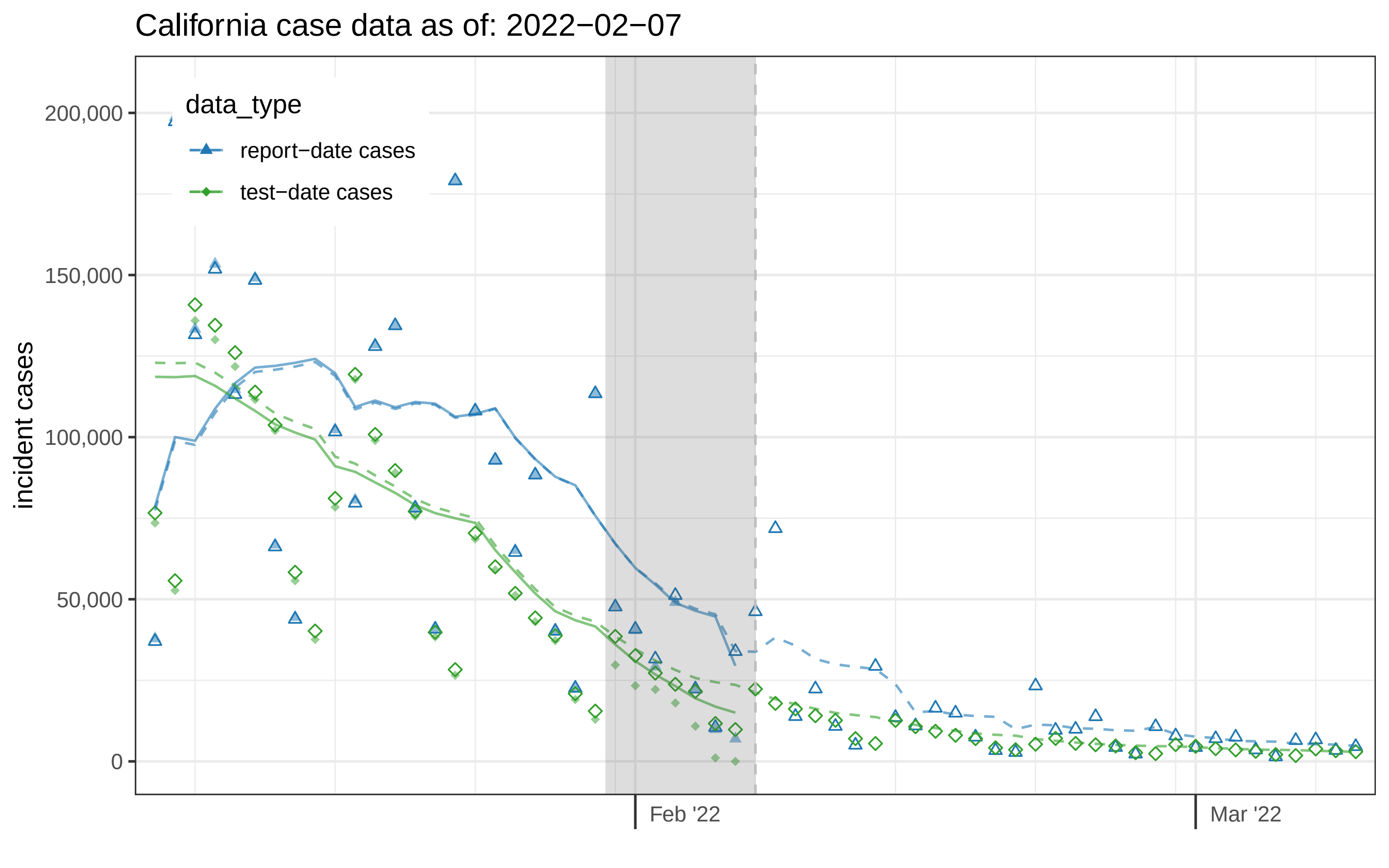
California case data as of: 2022−02−07
data_type
report−date cases
test−date cases
incident cases
200,000
150,000
100,000
50,000
0
Feb '22
Mar '22

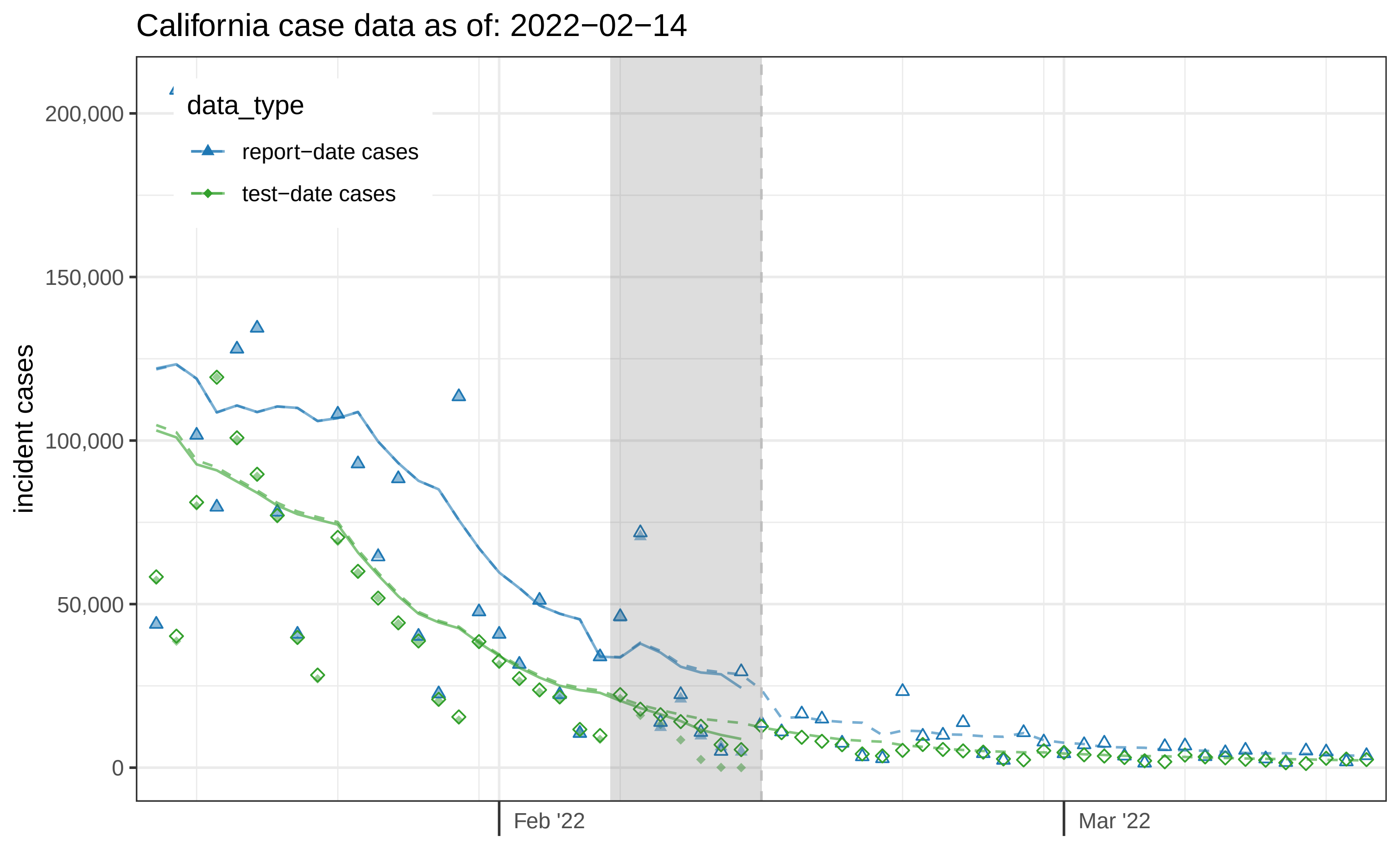
California case data as of: 2022−02−14
data_type
report−date cases
test−date cases
incident cases
200,000
150,000
100,000
50,000
0
Feb '22
Mar '22

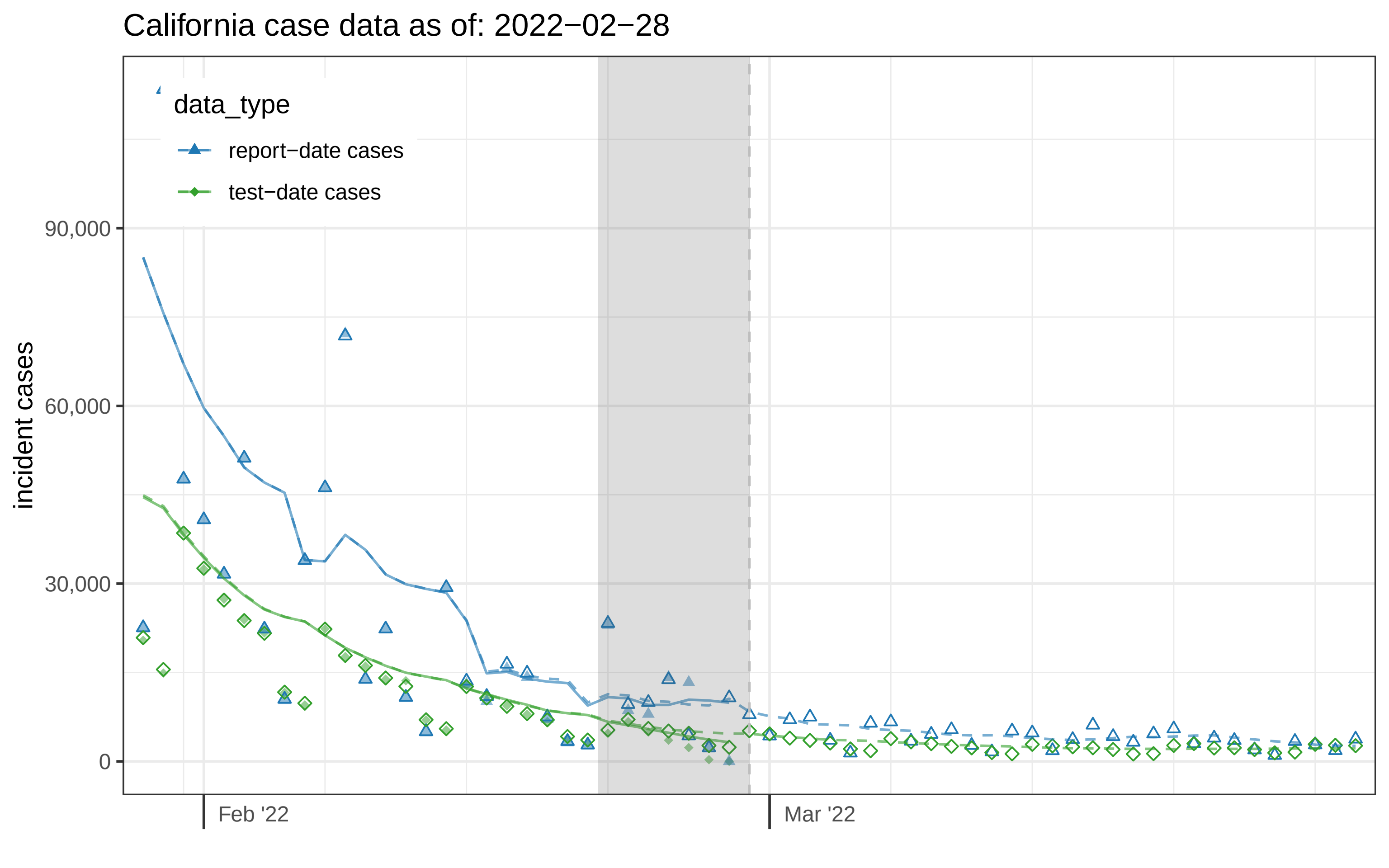

California case data as of: 2022−02−28
data_type
report−date cases
test−date cases
incident cases
90,000
60,000
30,000
0
Feb '22
Mar '22

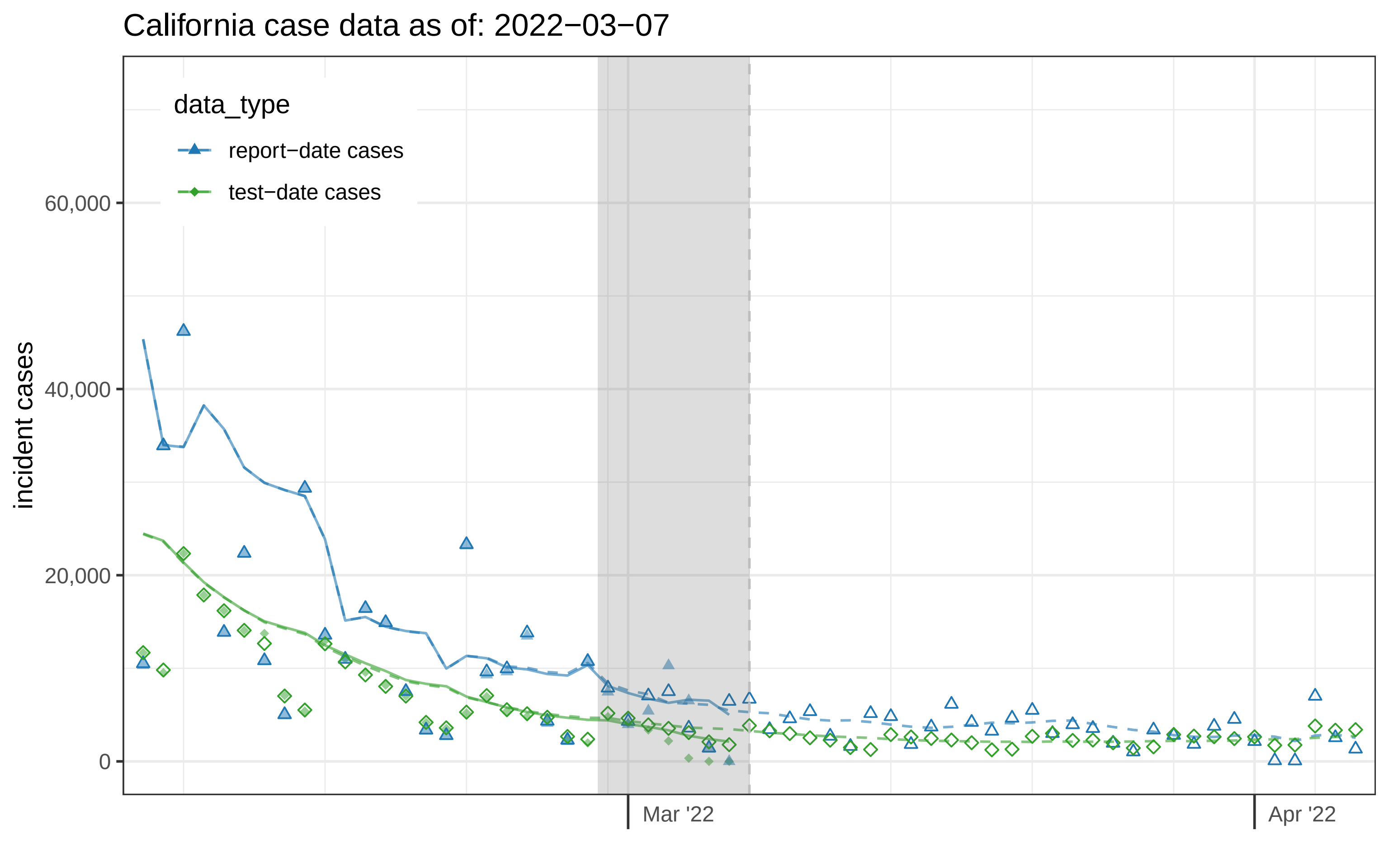

California case data as of: 2022−03−07
data_type
report−date cases
test−date cases
60,000
40,000
20,000
0
incident cases
Mar '22
Apr '22

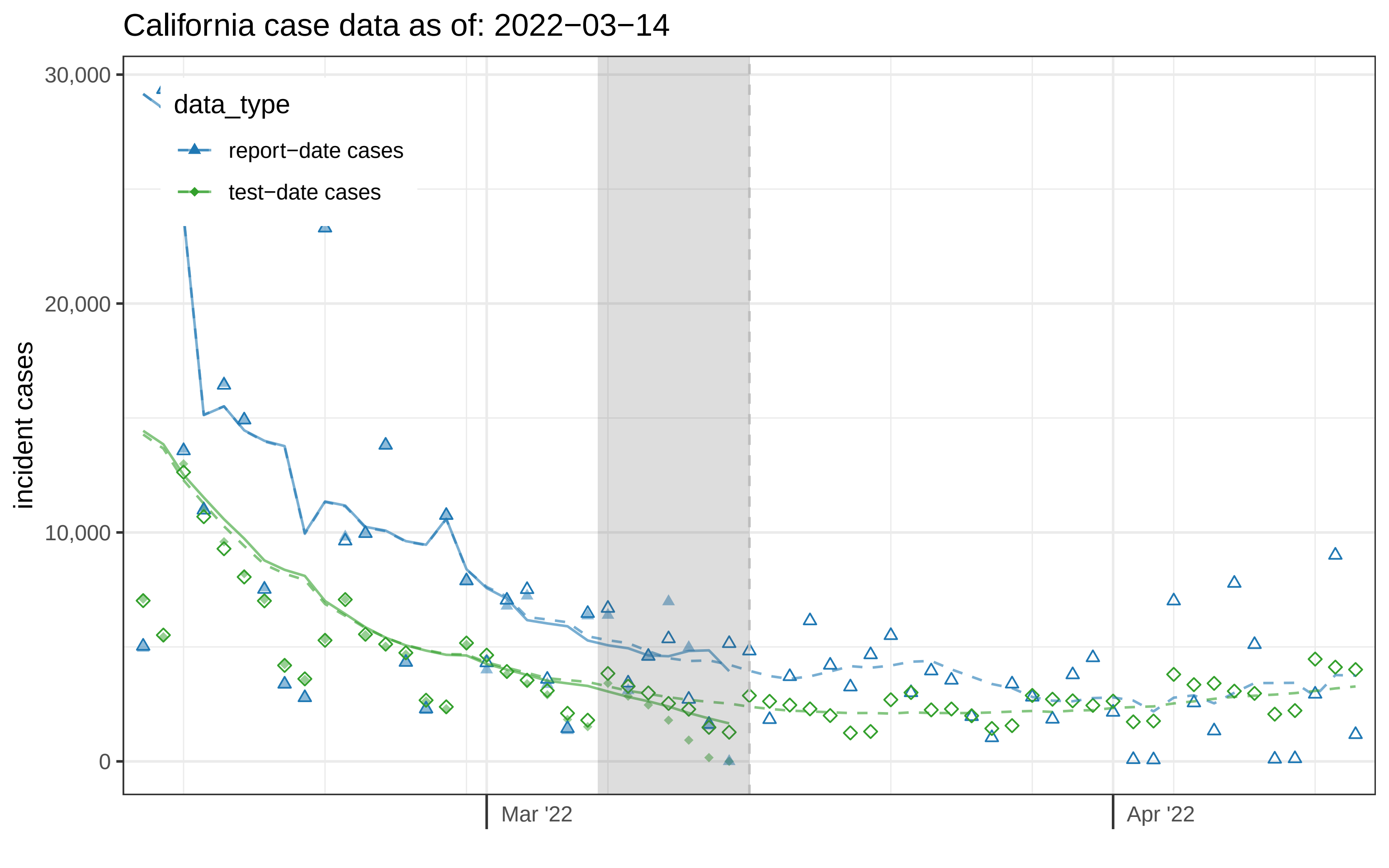

California case data as of: 2022−03−14
data_type
report−date cases
test−date cases
incident cases
30,000
20,000
10,000
0
Mar '22
Apr '22

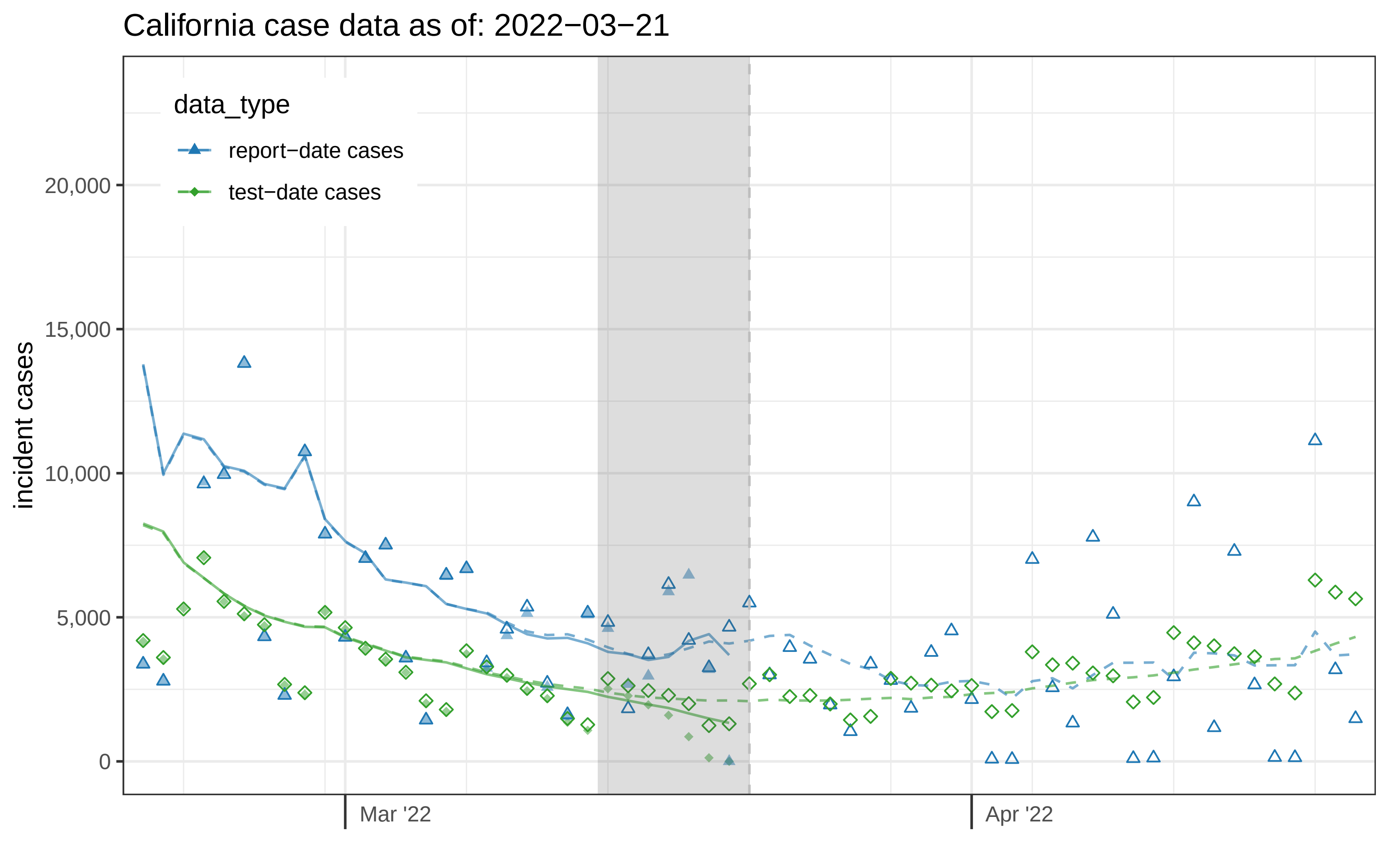

California case data as of: 2022−03−21
data_type
report−date cases
test−date cases
incident cases
20,000
15,000
10,000
5,000
0
Mar '22
Apr '22

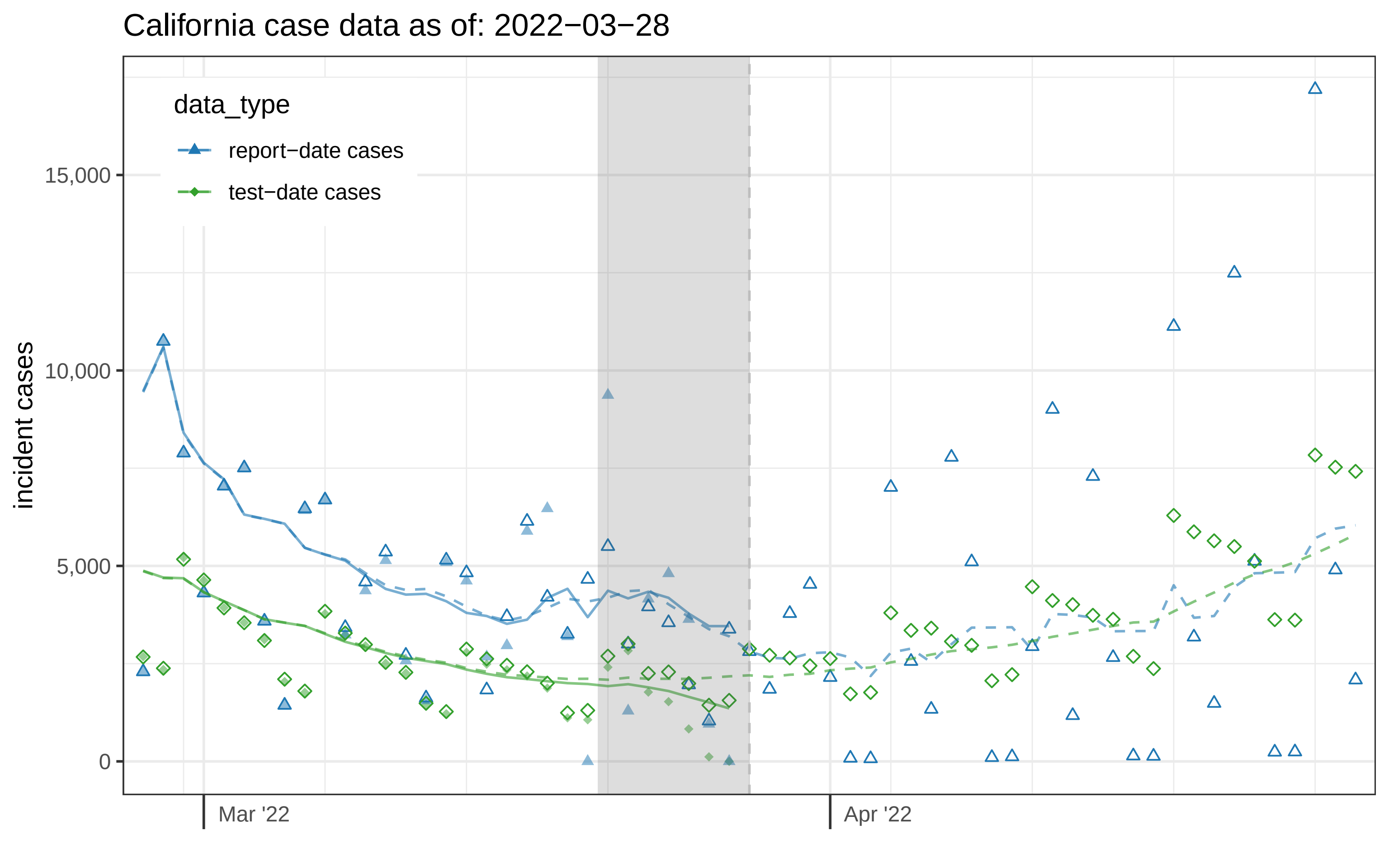
California case data as of: 2022−03−28
data_type
report−date cases
test−date cases
incident cases
15,000
10,000
5,000
0
Mar '22
Apr '22

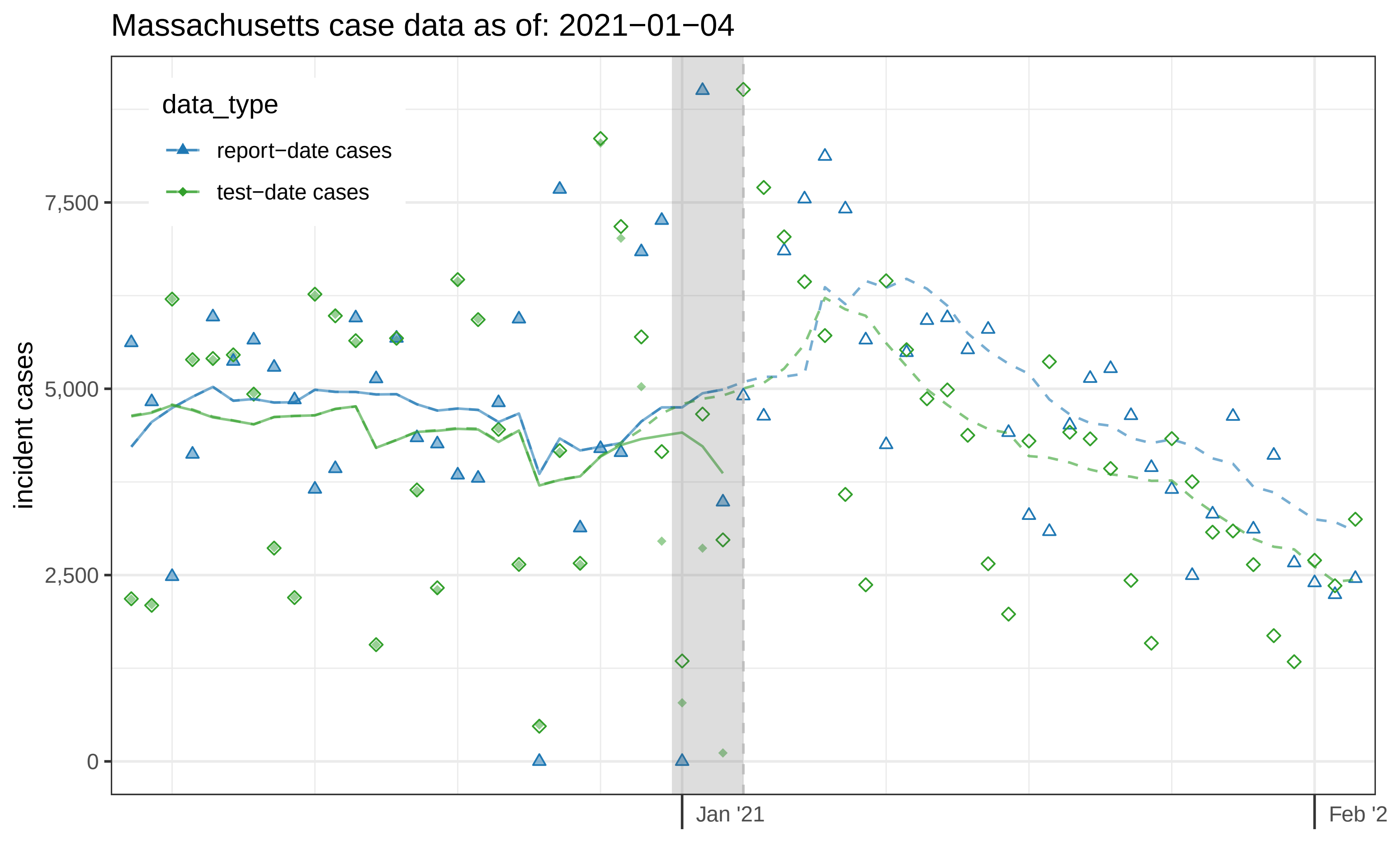

Massachusetts case data as of: 2021−01−04
data_type
report−date cases
test−date cases
incident cases
7,500
5,000
2,500
0
Jan '21
Feb '2

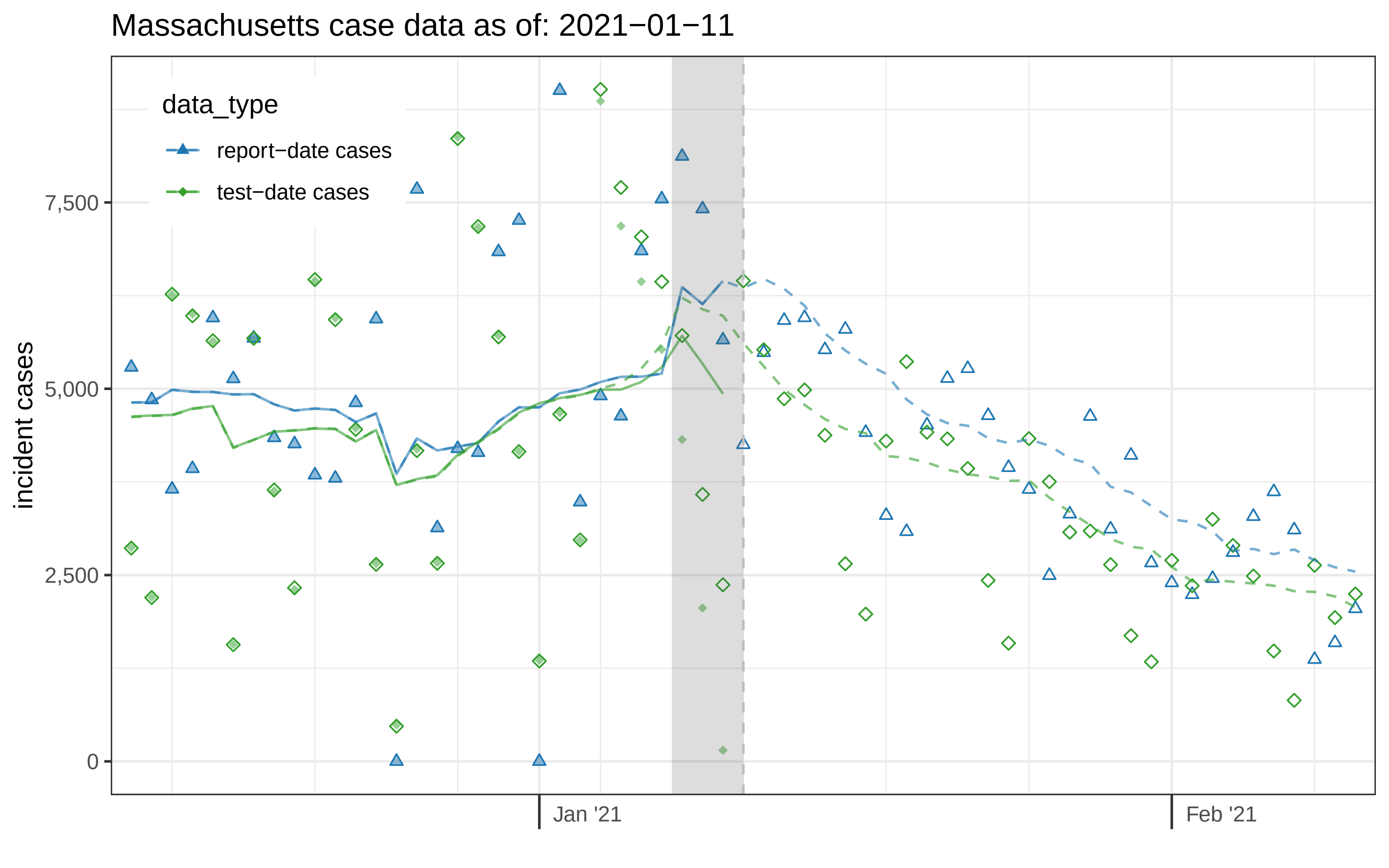

Massachusetts case data as of: 2021−01−11
data_type
report−date cases
test−date cases
incident cases
7,500
5,000
2,500
0
Jan '21
Feb '21

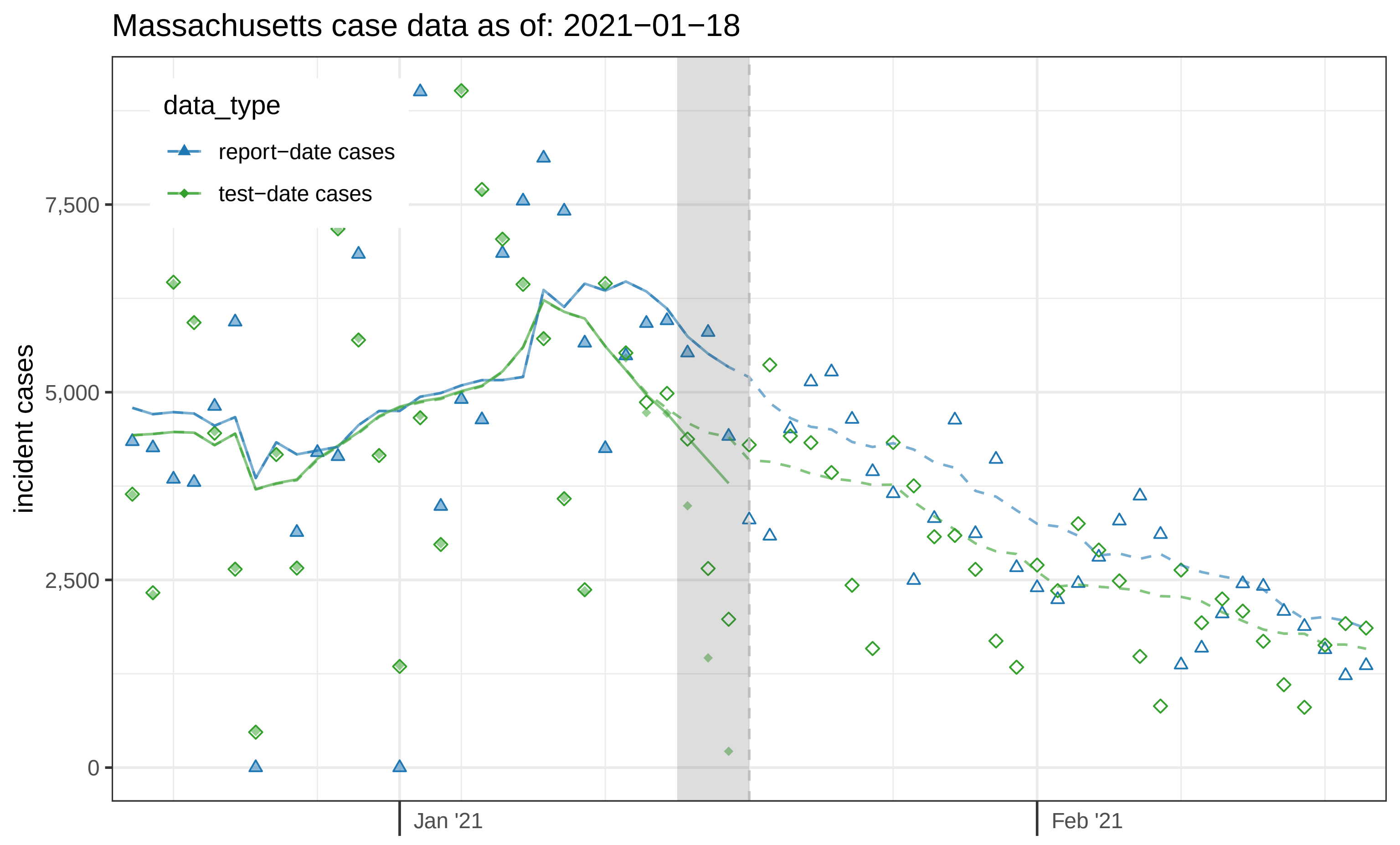

Massachusetts case data as of: 2021−01−18
data_type
report−date cases
test−date cases
incident cases
7,500
5,000
2,500
0
Jan '21
Feb '21

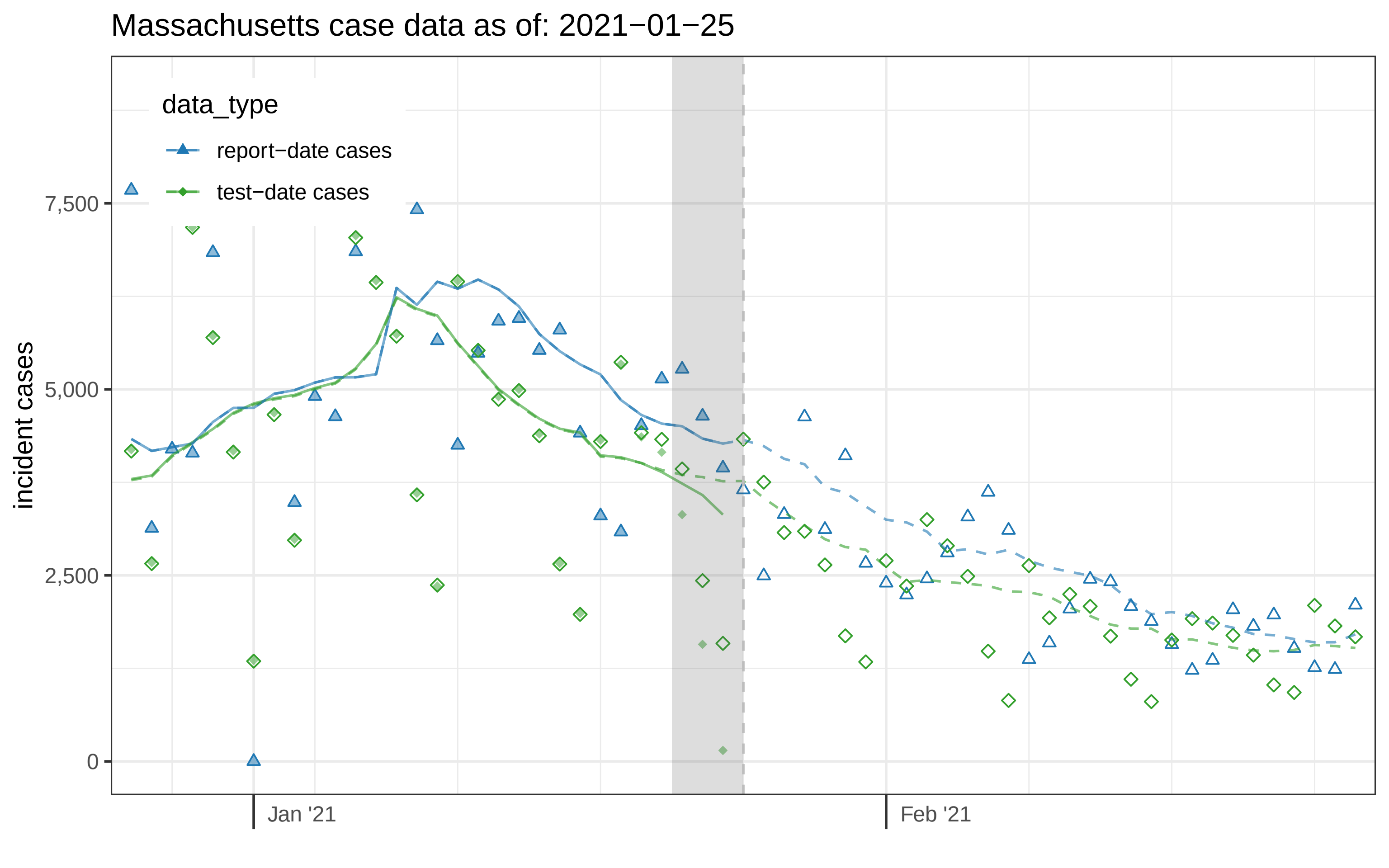

Massachusetts case data as of: 2021−01−25
data_type
report−date cases
test−date cases
7,500
5,000
2,500
0
incident cases
Jan '21
Feb '21

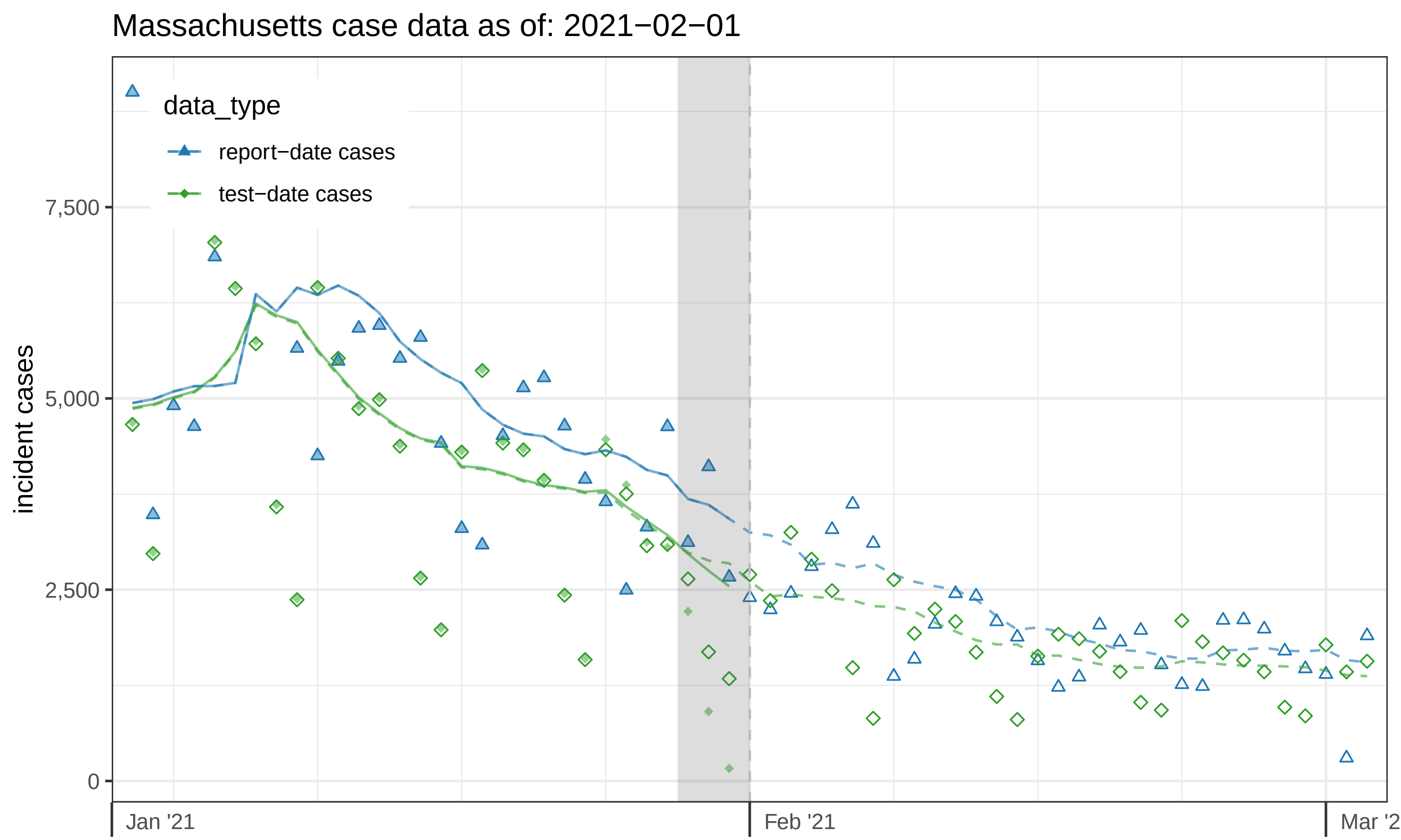

Massachusetts case data as of: 2021−02−01
data_type
report−date cases
test−date cases
incident cases
7,500
5,000
2,500
0
Jan '21
Feb '21
Mar '2

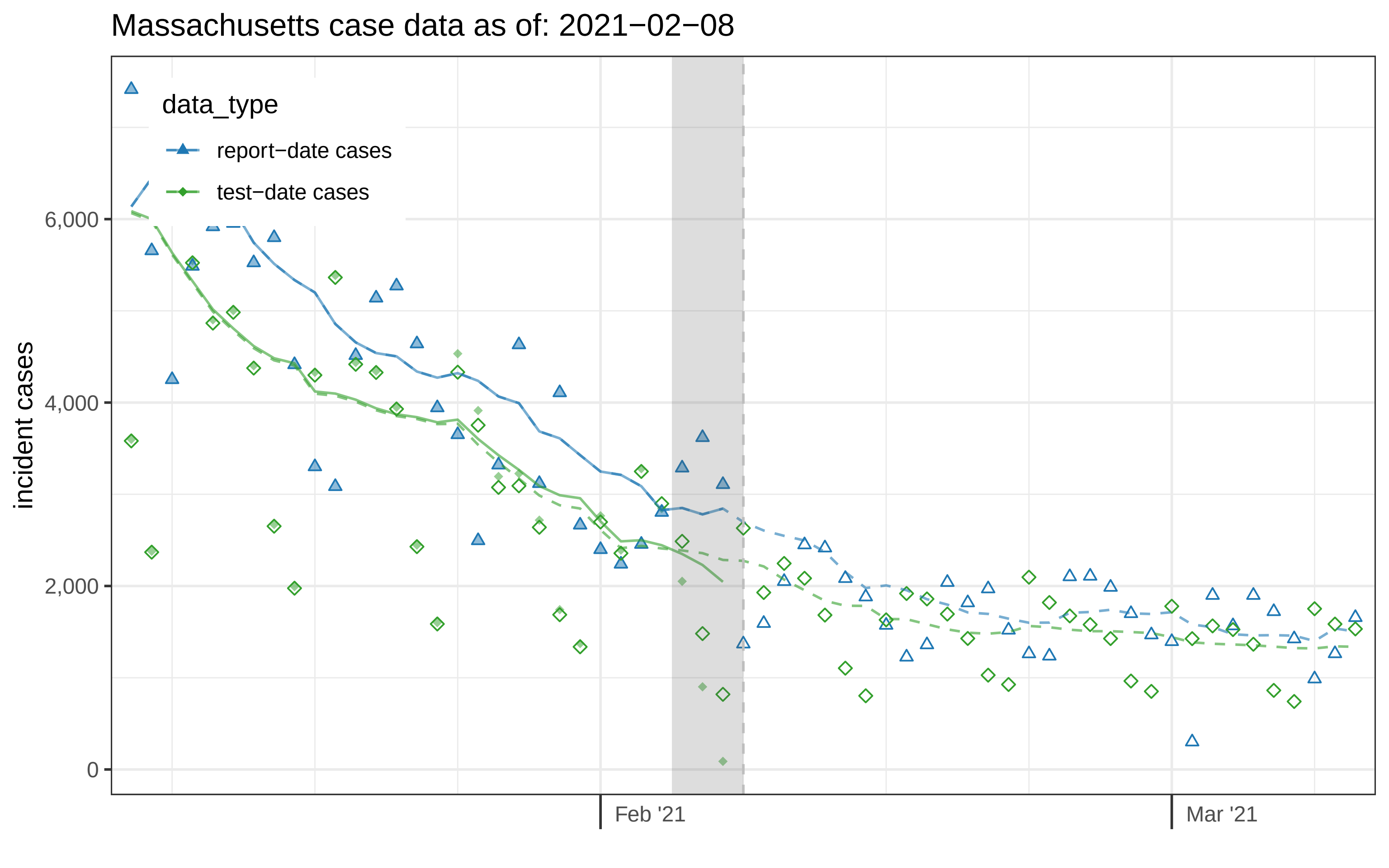
Massachusetts case data as of: 2021−02−08
data_type
report−date cases
test−date cases
incident cases
6,000
4,000
2,000
0
Feb '21
Mar '21

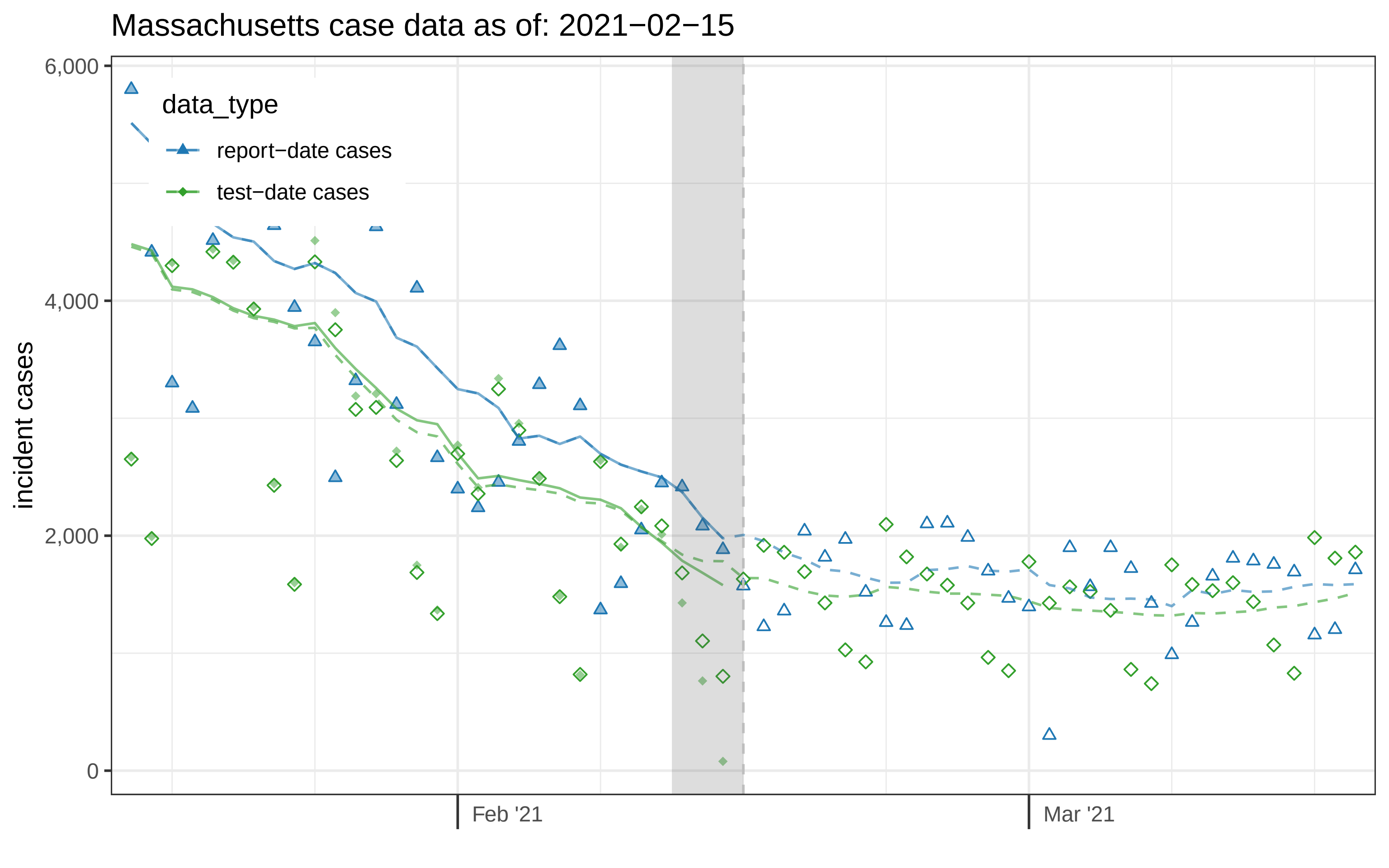

Massachusetts case data as of: 2021−02−15
6,000
4,000
2,000
0
incident cases
data_type
report−date cases
test−date cases
Feb '21
Mar '21

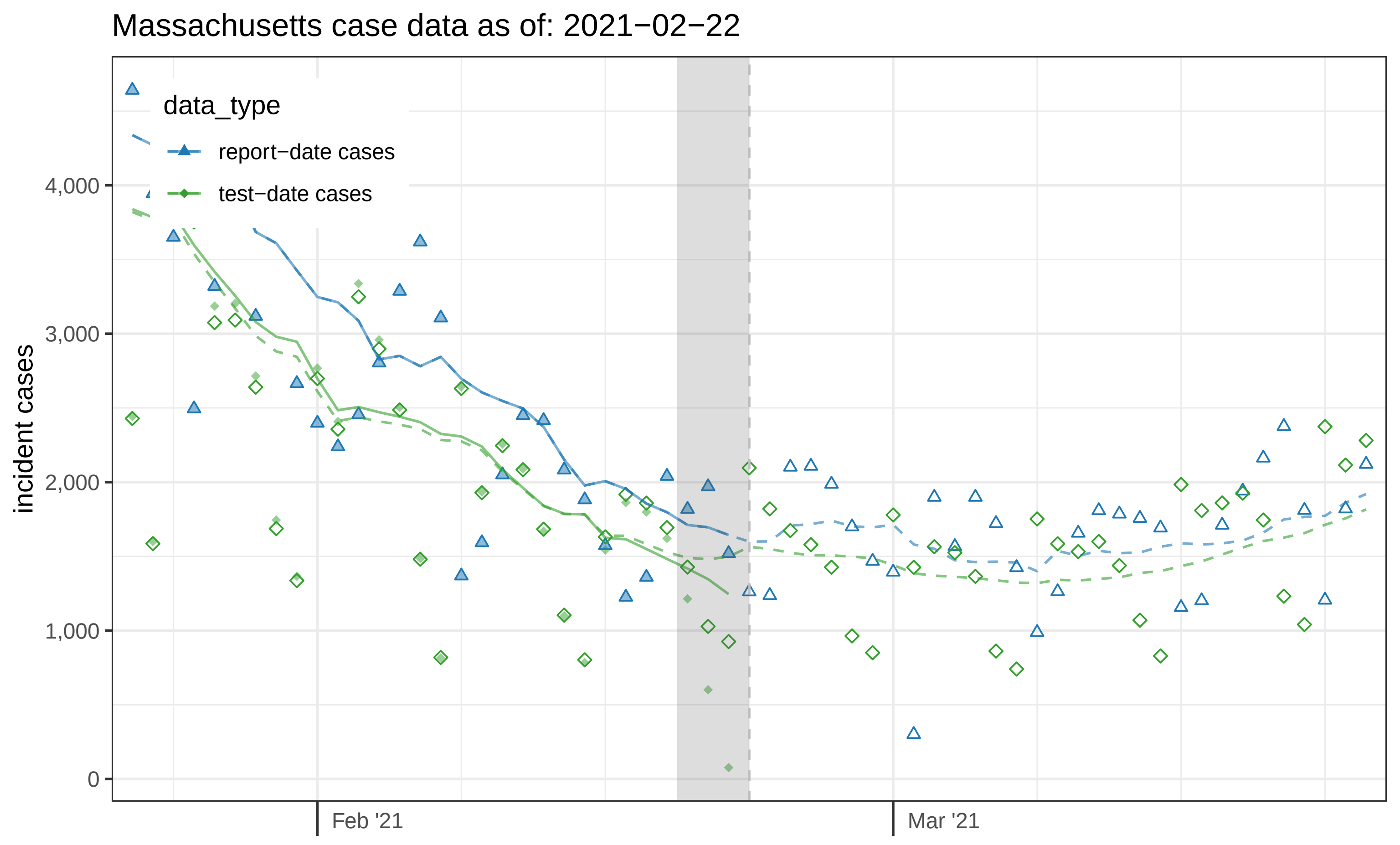

Massachusetts case data as of: 2021−02−22
data_type
report−date cases
test−date cases
incident cases
4,000
3,000
2,000
1,000
0
Feb '21
Mar '21

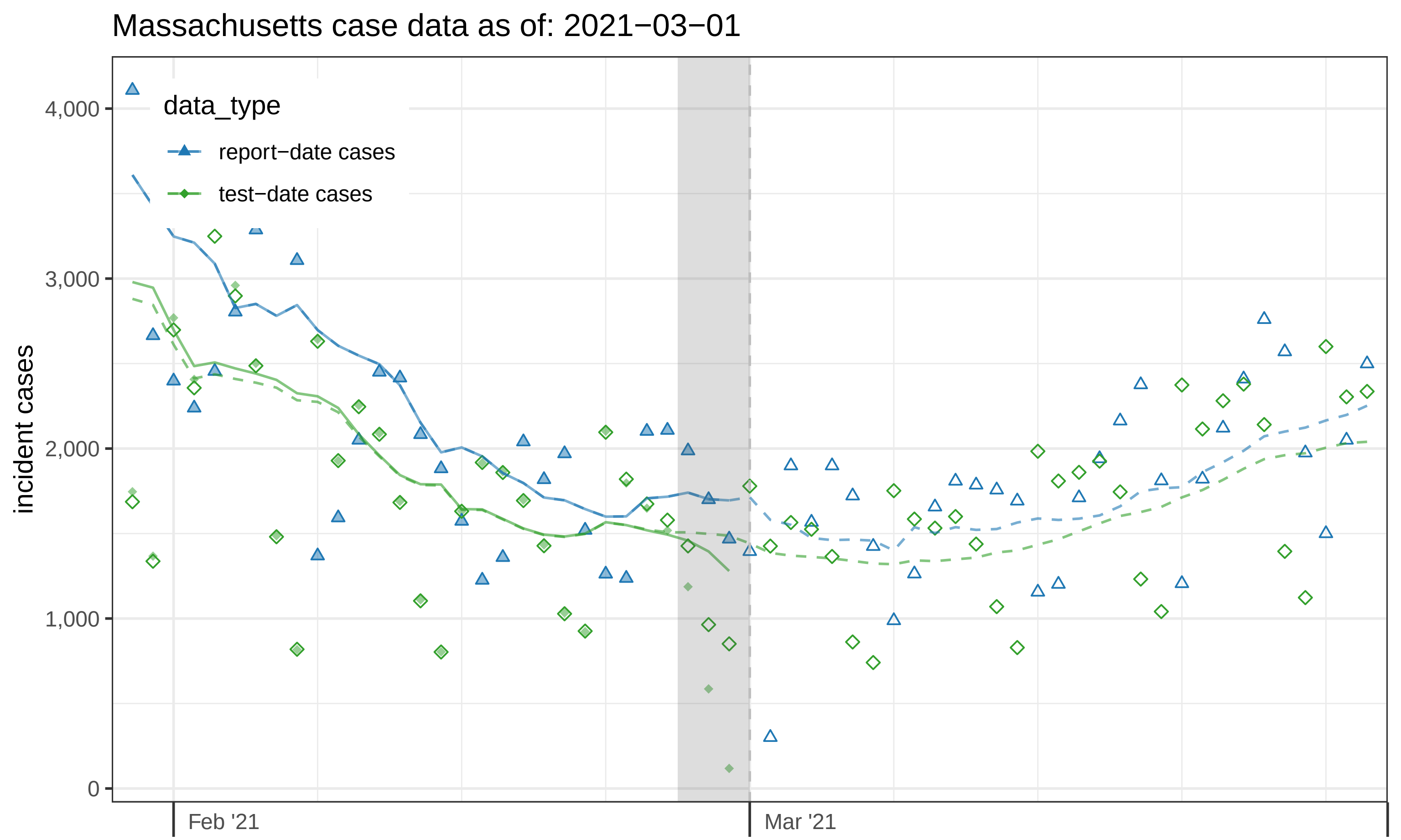

Massachusetts case data as of: 2021−03−01
data_type
report−date cases
test−date cases
incident cases
4,000
3,000
2,000
1,000
0
Feb '21
Mar '21

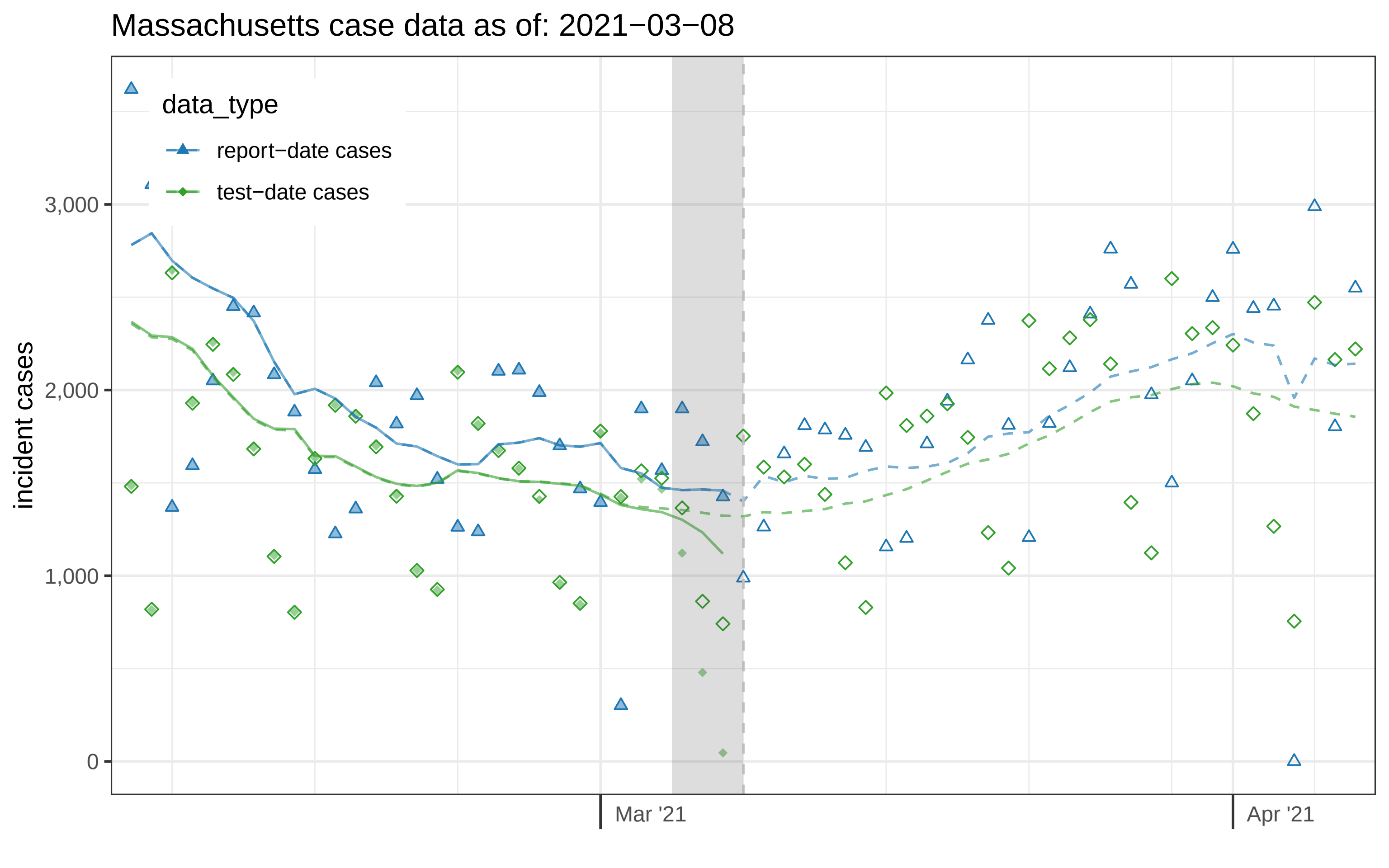

Massachusetts case data as of: 2021−03−08
data_type
report−date cases
test−date cases
incident cases
3,000
2,000
1,000
0
Mar '21
Apr '21

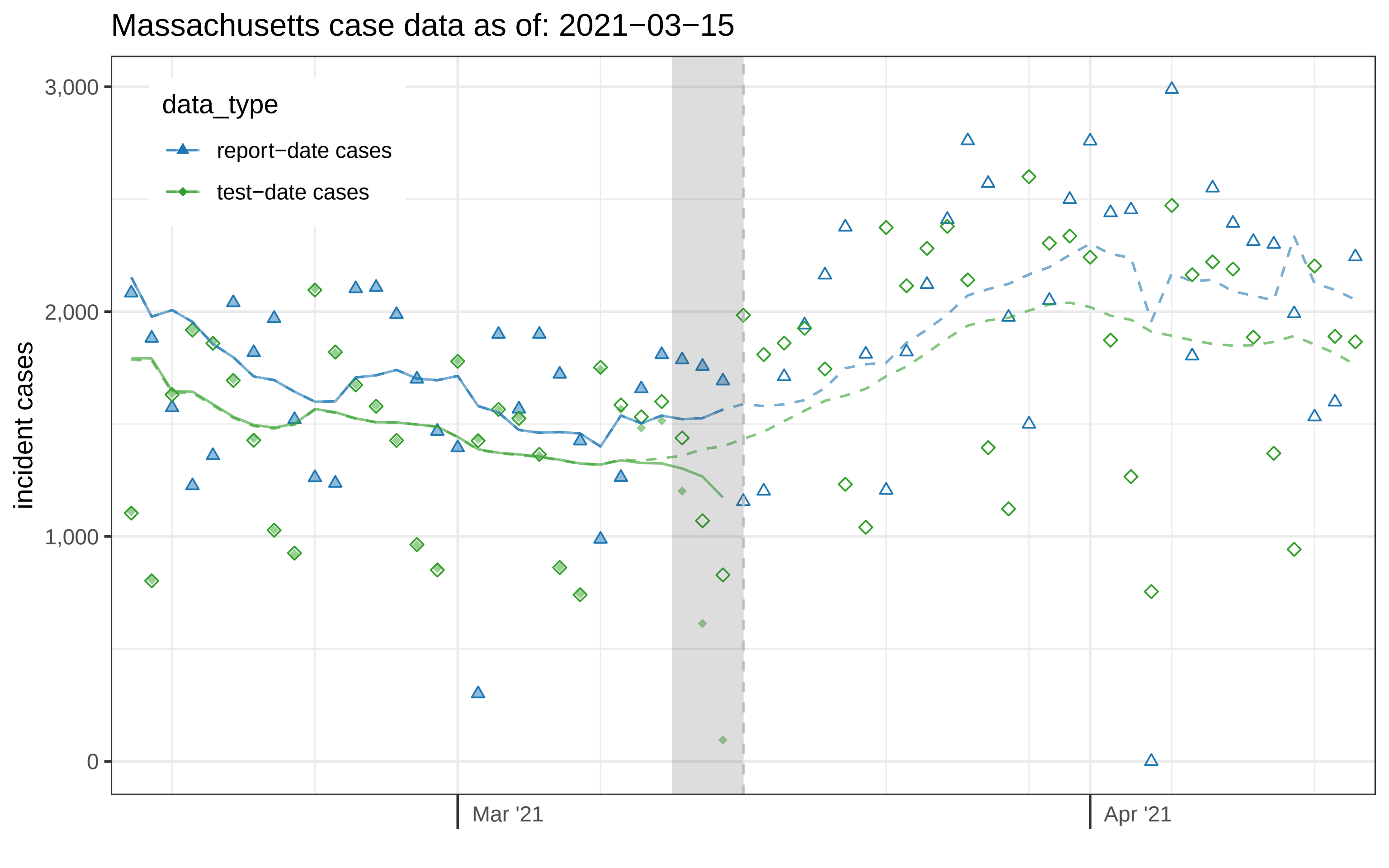
Massachusetts case data as of: 2021−03−15
data_type
report−date cases
test−date cases
incident cases
3,000
2,000
1,000
0
Mar '21
Apr '21

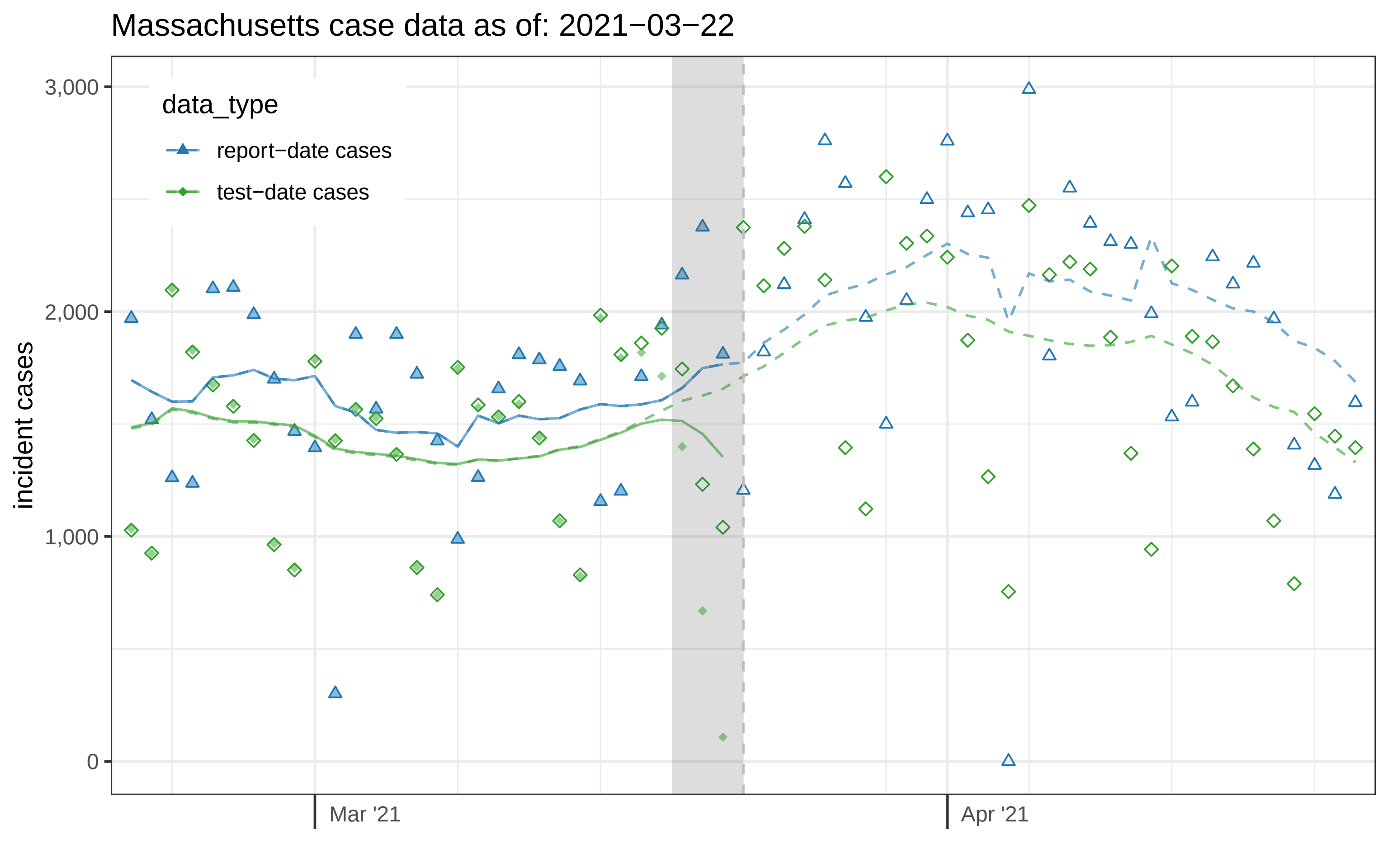

Massachusetts case data as of: 2021−03−22
data_type
report−date cases
test−date cases
incident cases
3,000
2,000
1,000
0
Mar '21
Apr '21

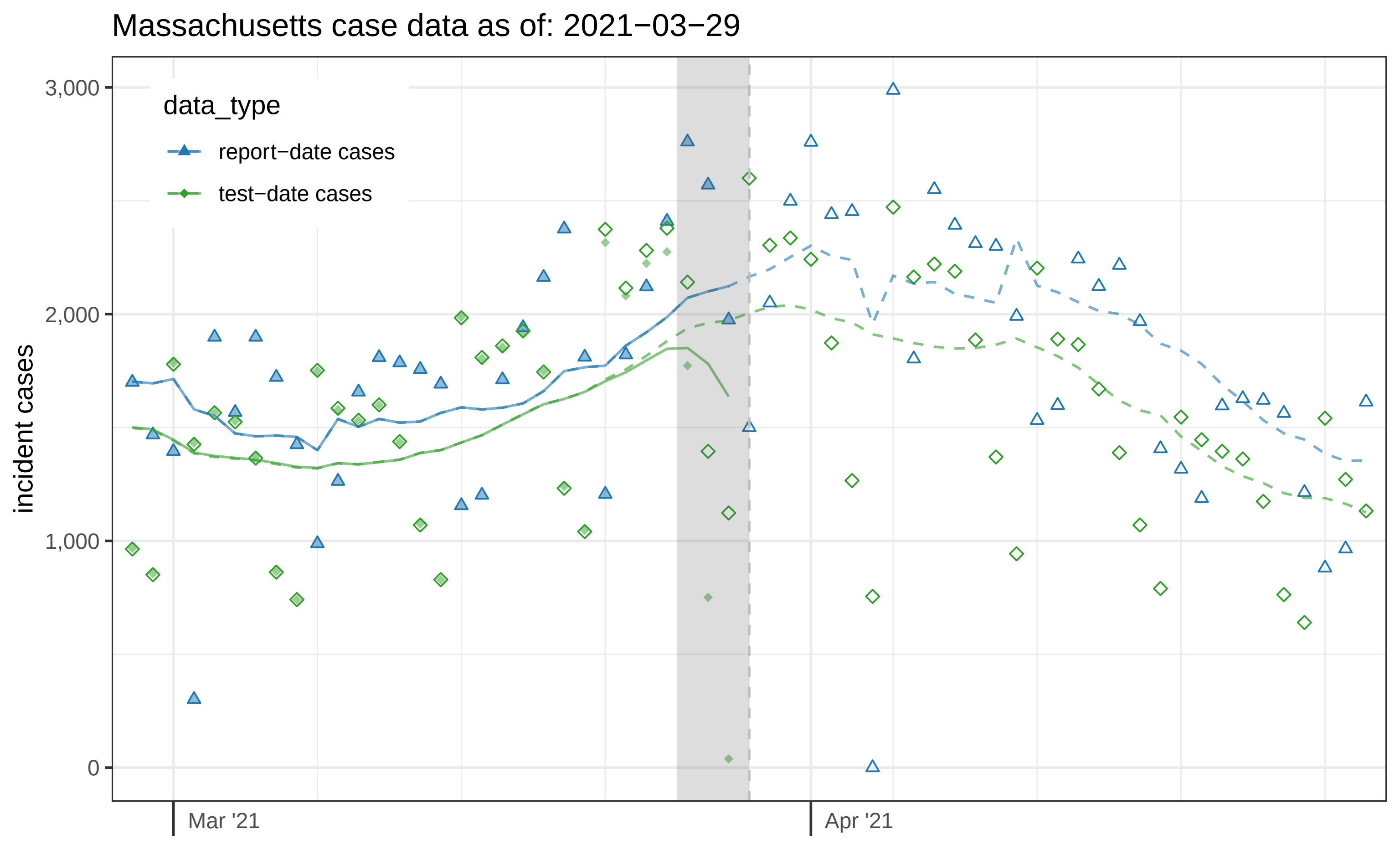

Massachusetts case data as of: 2021−03−29
data_type
report−date cases
test−date cases
incident cases
3,000
2,000
1,000
0
Mar '21
Apr '21

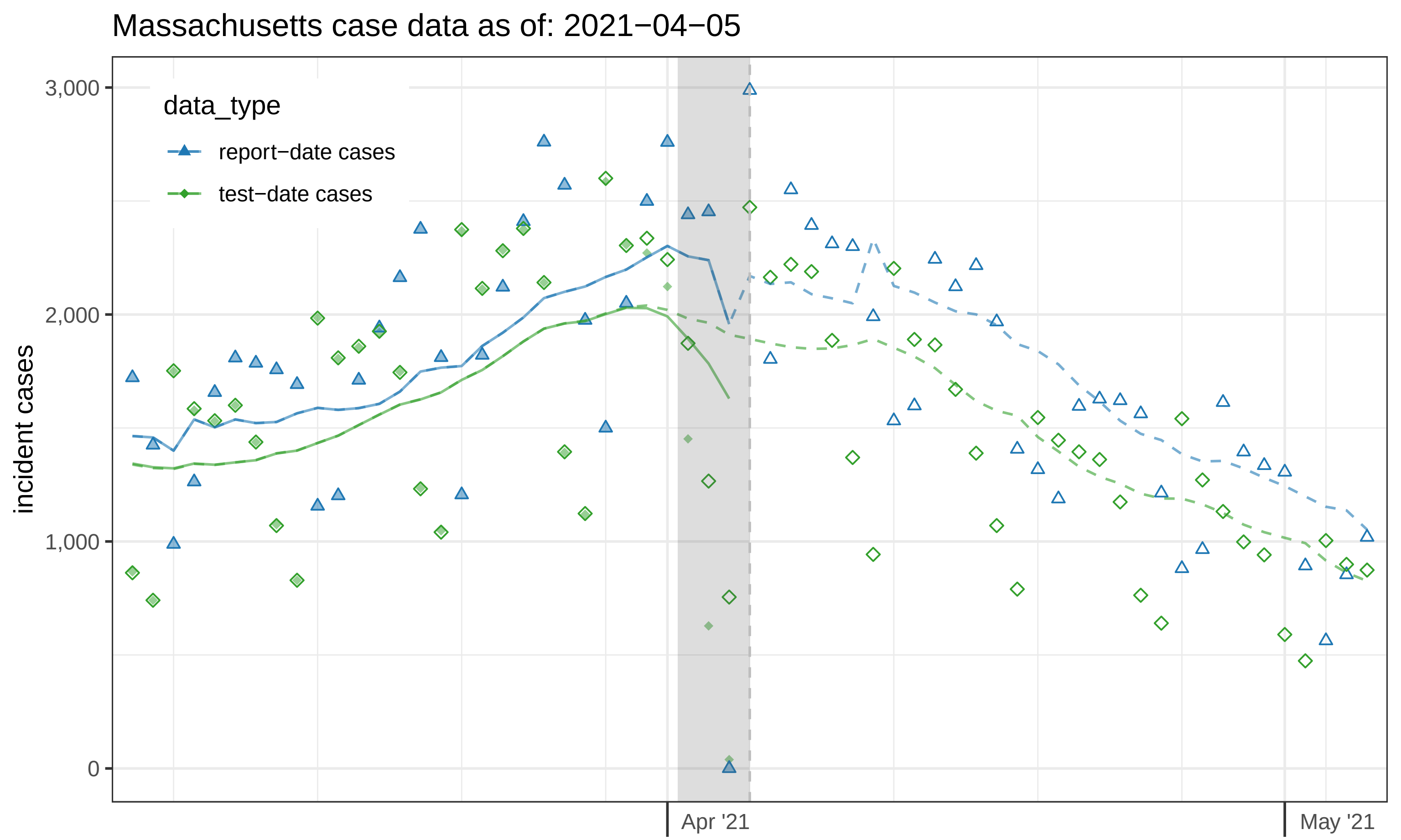
Massachusetts case data as of: 2021−04−05
data_type
report−date cases
test−date cases
incident cases
3,000
2,000
1,000
0
Apr '21
May '21

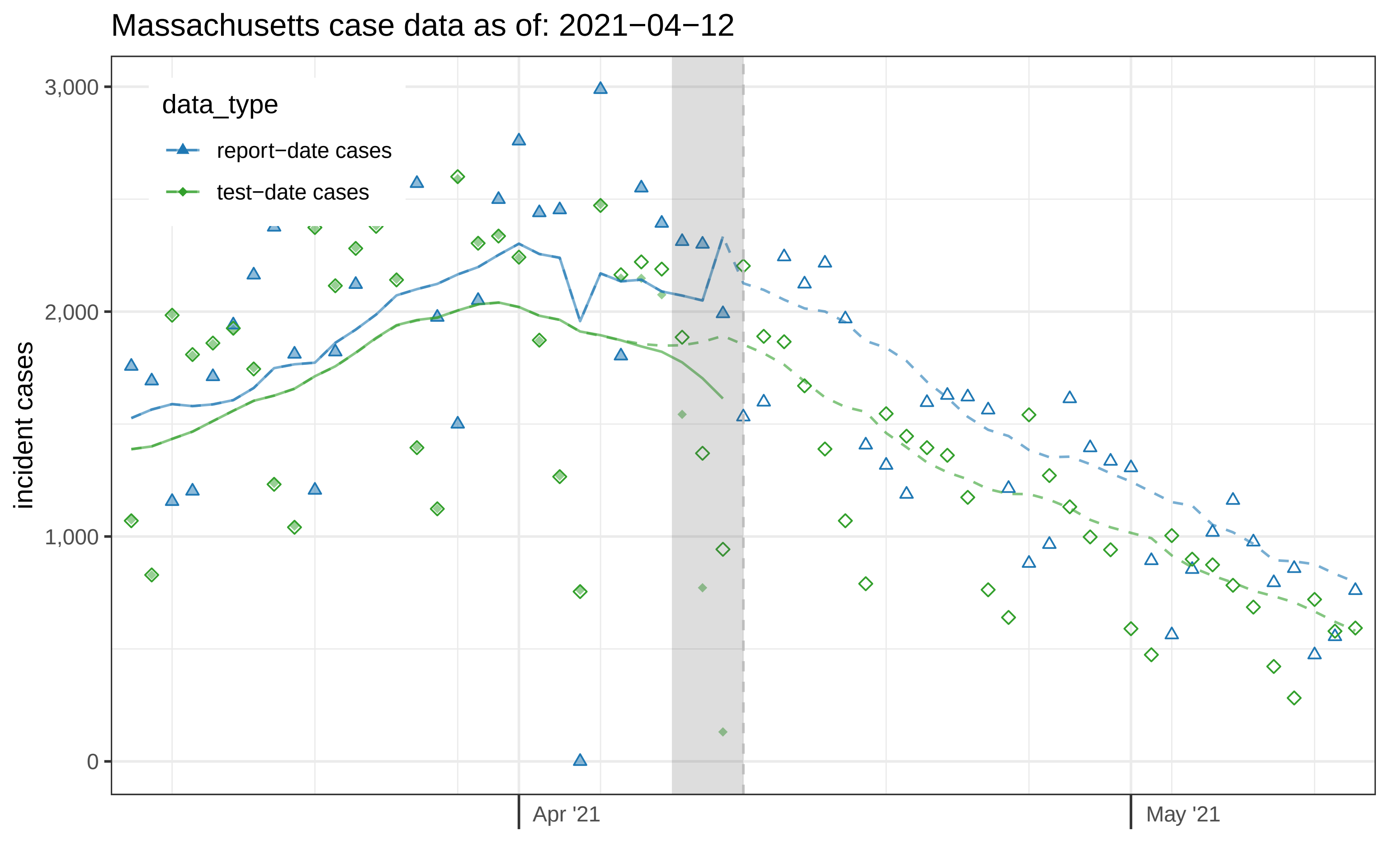

Massachusetts case data as of: 2021−04−12
data_type
report−date cases
test−date cases
incident cases
3,000
2,000
1,000
0
Apr '21
May '21

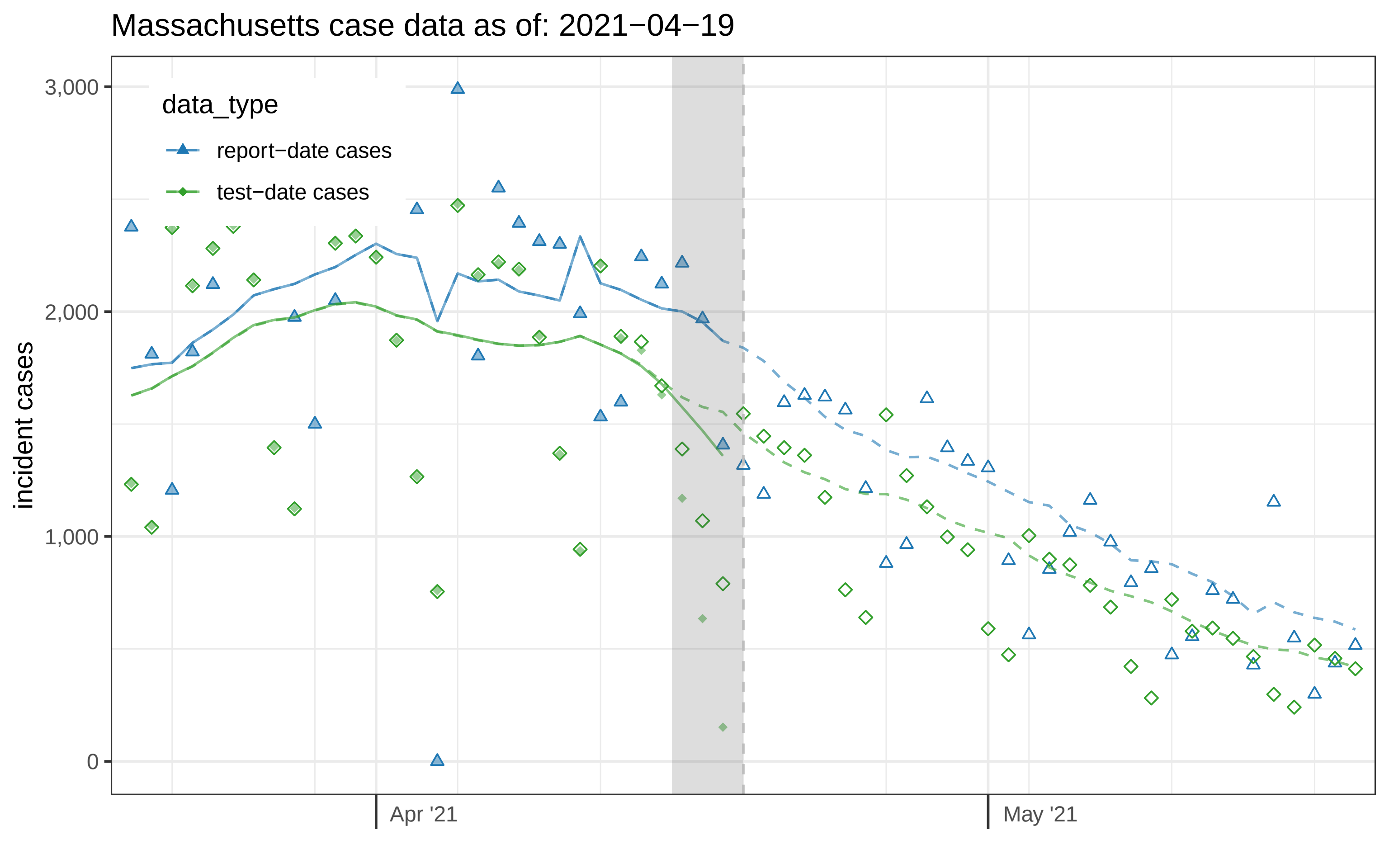

Massachusetts case data as of: 2021−04−19
data_type
report−date cases
test−date cases
incident cases
3,000
2,000
1,000
0
Apr '21
May '21

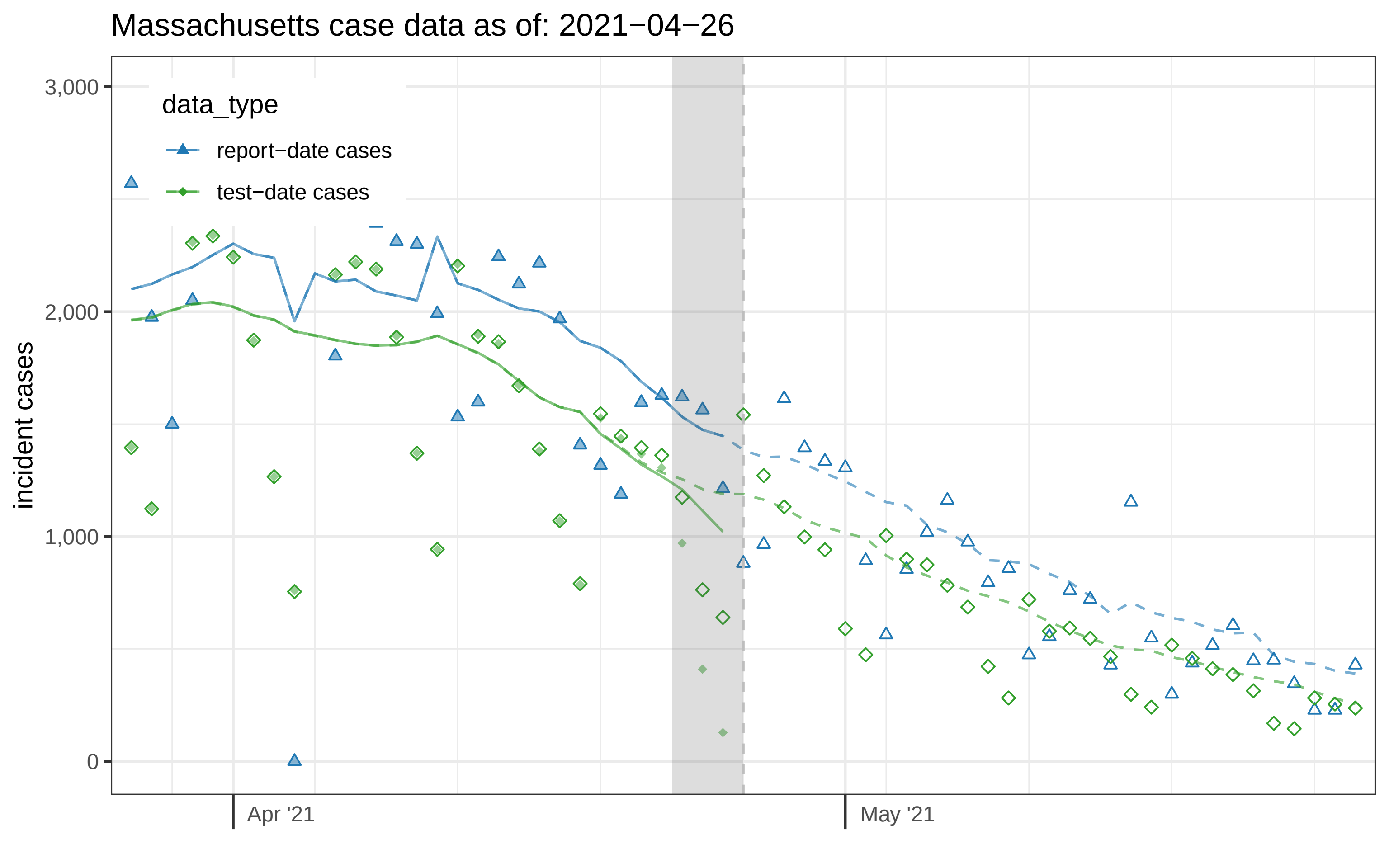

Massachusetts case data as of: 2021−04−26
data_type
report−date cases
test−date cases
incident cases
3,000
2,000
1,000
0
Apr '21
May '21

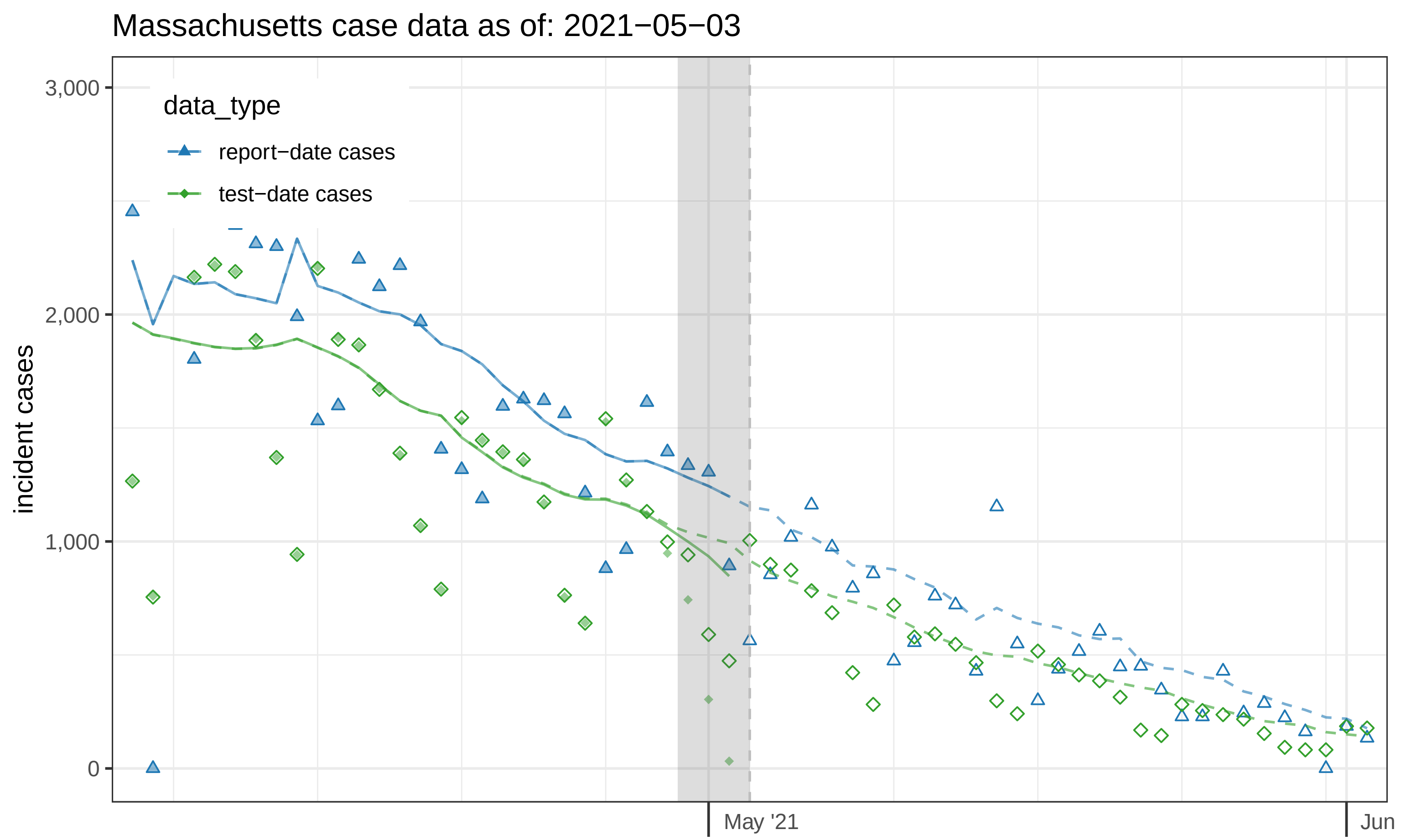

Massachusetts case data as of: 2021−05−03
data_type
report−date cases
test−date cases
incident cases
3,000
2,000
1,000
0
May '21
Jun

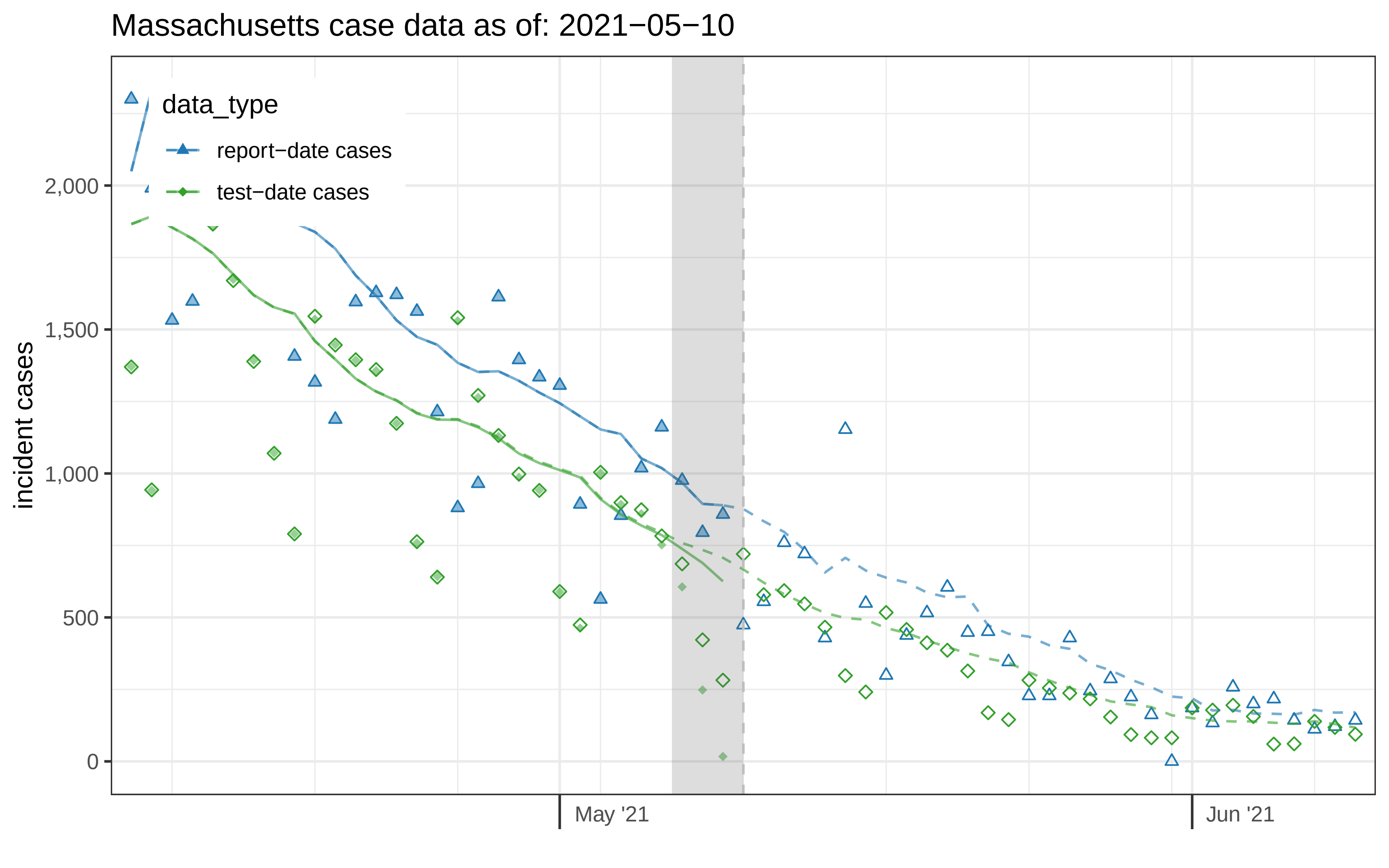
Massachusetts case data as of: 2021−05−10
data_type
report−date cases
test−date cases
incident cases
2,000
1,500
1,000
500
0
May '21
Jun '21

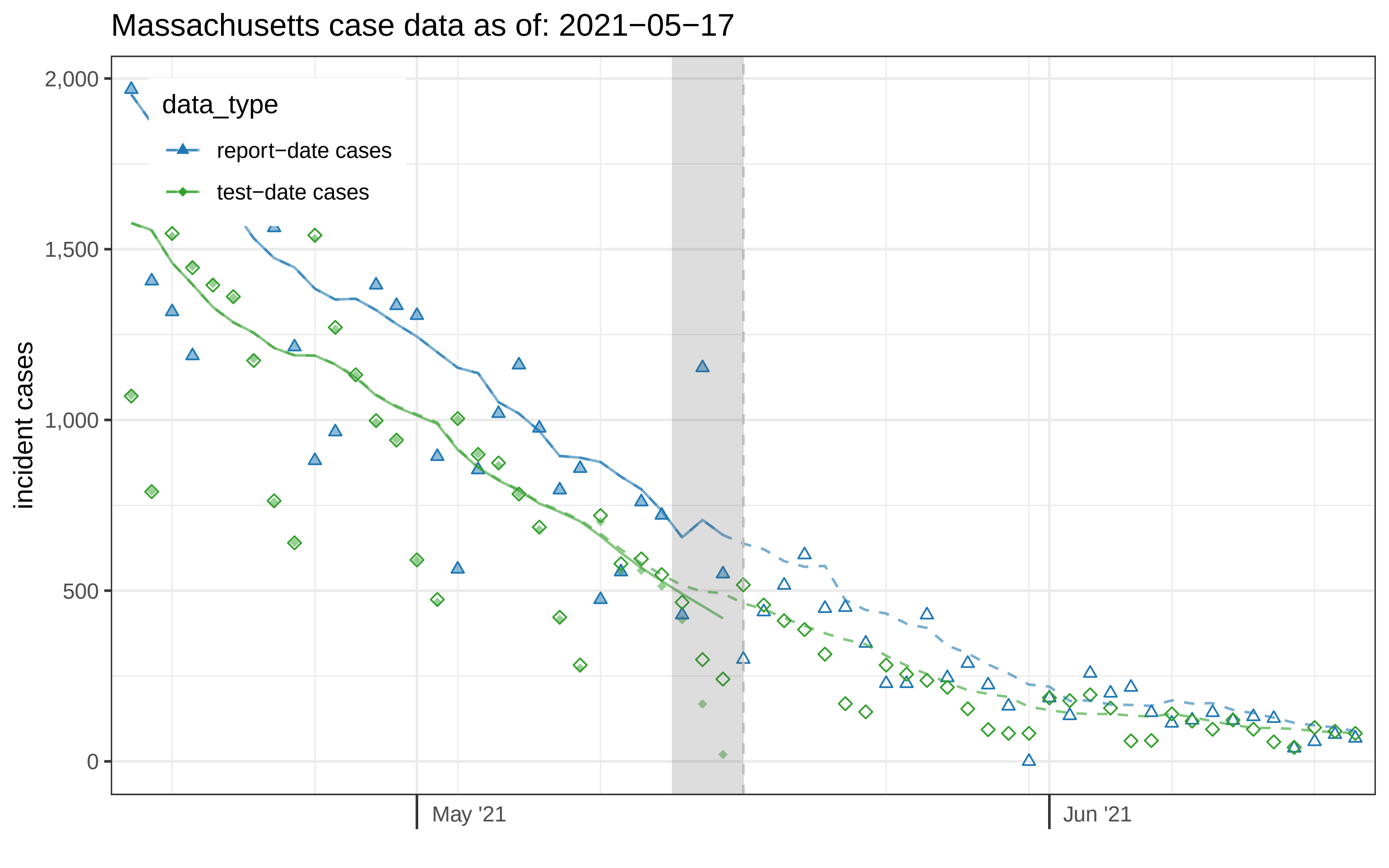

Massachusetts case data as of: 2021−05−17
incident cases
2,000
1,500
1,000
500
0
data_type
report−date cases
test−date cases
May '21
Jun '21

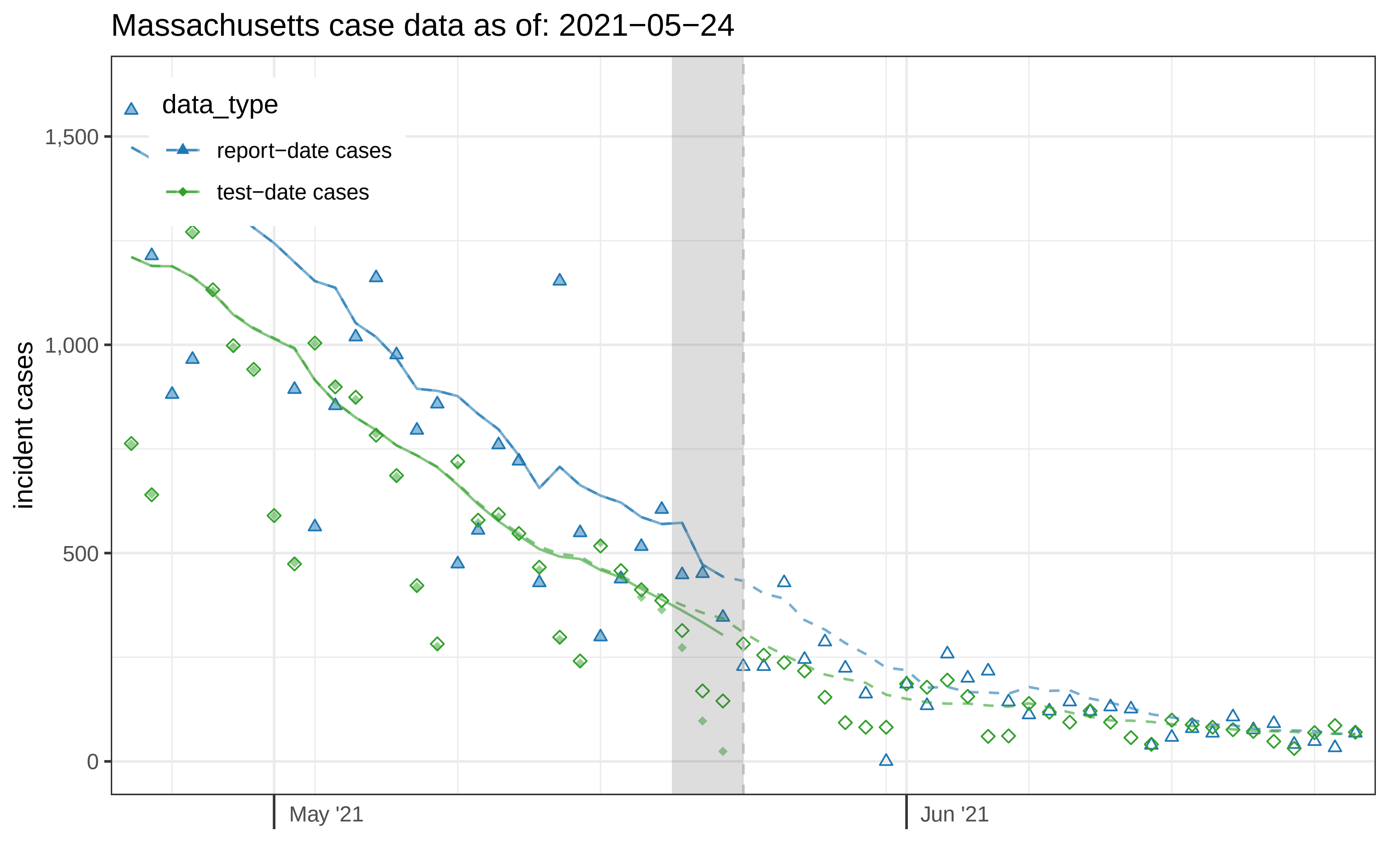
Massachusetts case data as of: 2021−05−24
data_type
report−date cases
test−date cases
incident cases
1,500
1,000
500
0
May '21
Jun '21

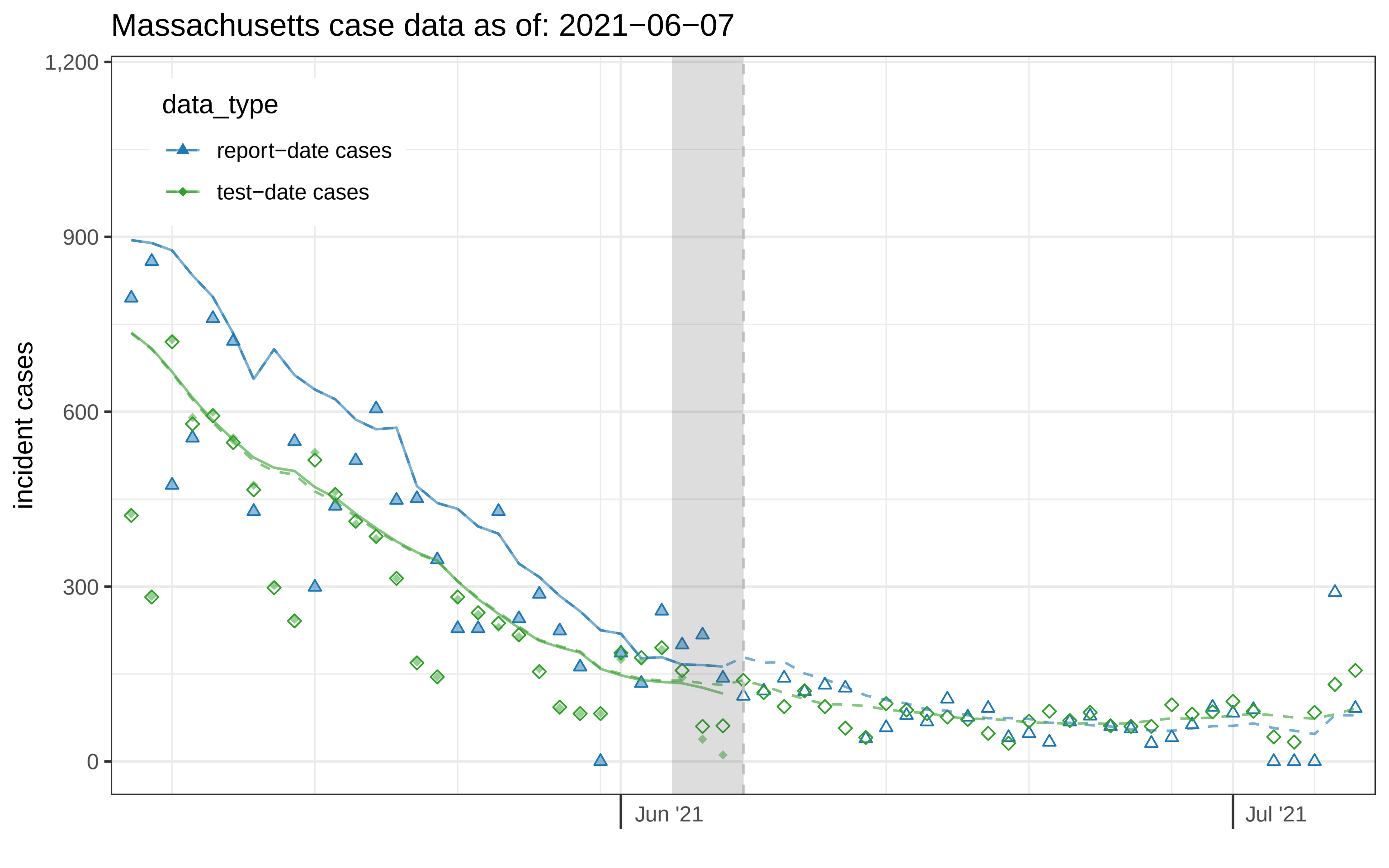
Massachusetts case data as of: 2021−06−07
incident cases
1,200
900
600
300
0
data_type
report−date cases
test−date cases
Jun '21
Jul '21

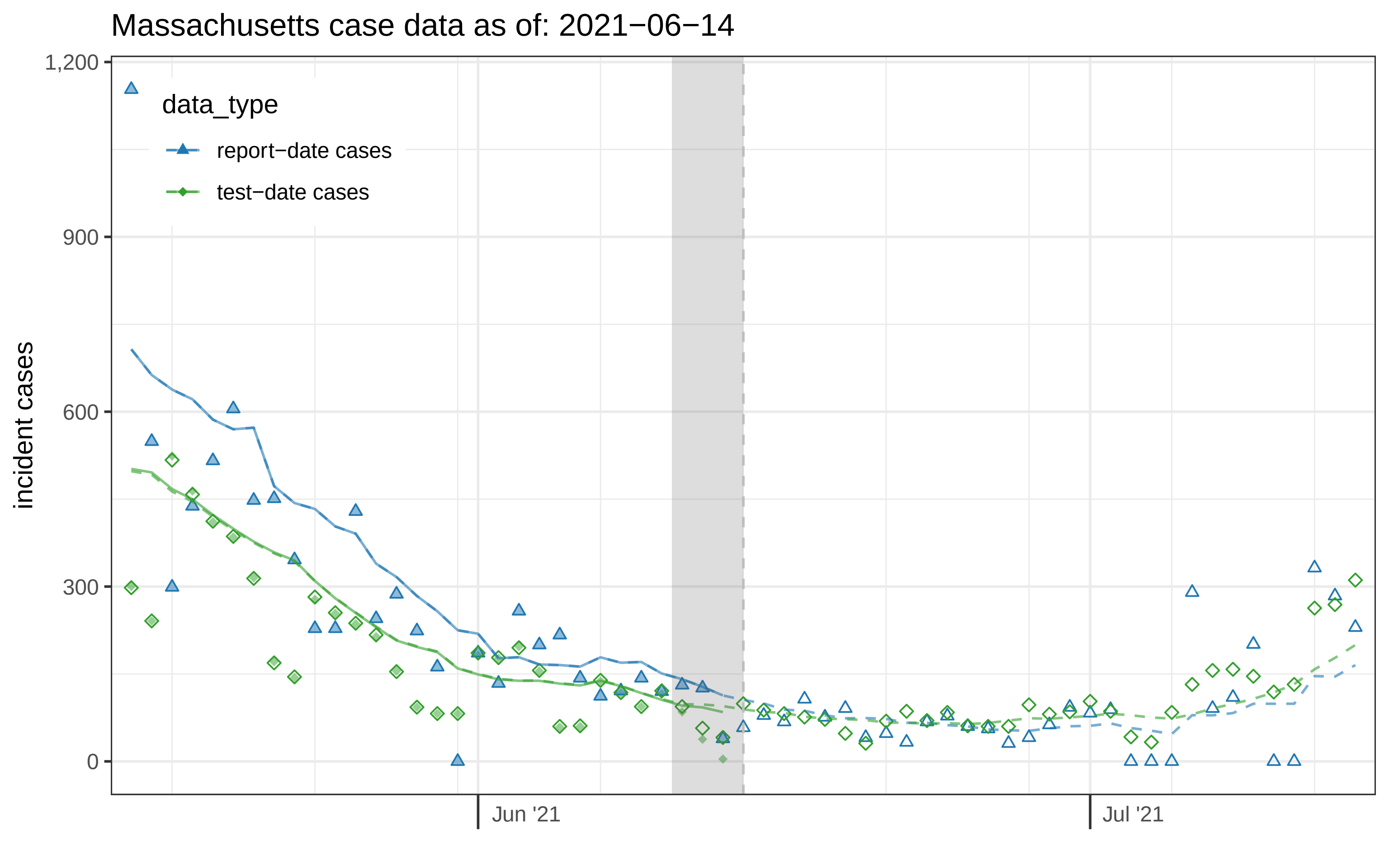

Massachusetts case data as of: 2021−06−14
1,200
900
600
300
0
incident cases
data_type
report−date cases
test−date cases
Jun '21
Jul '21

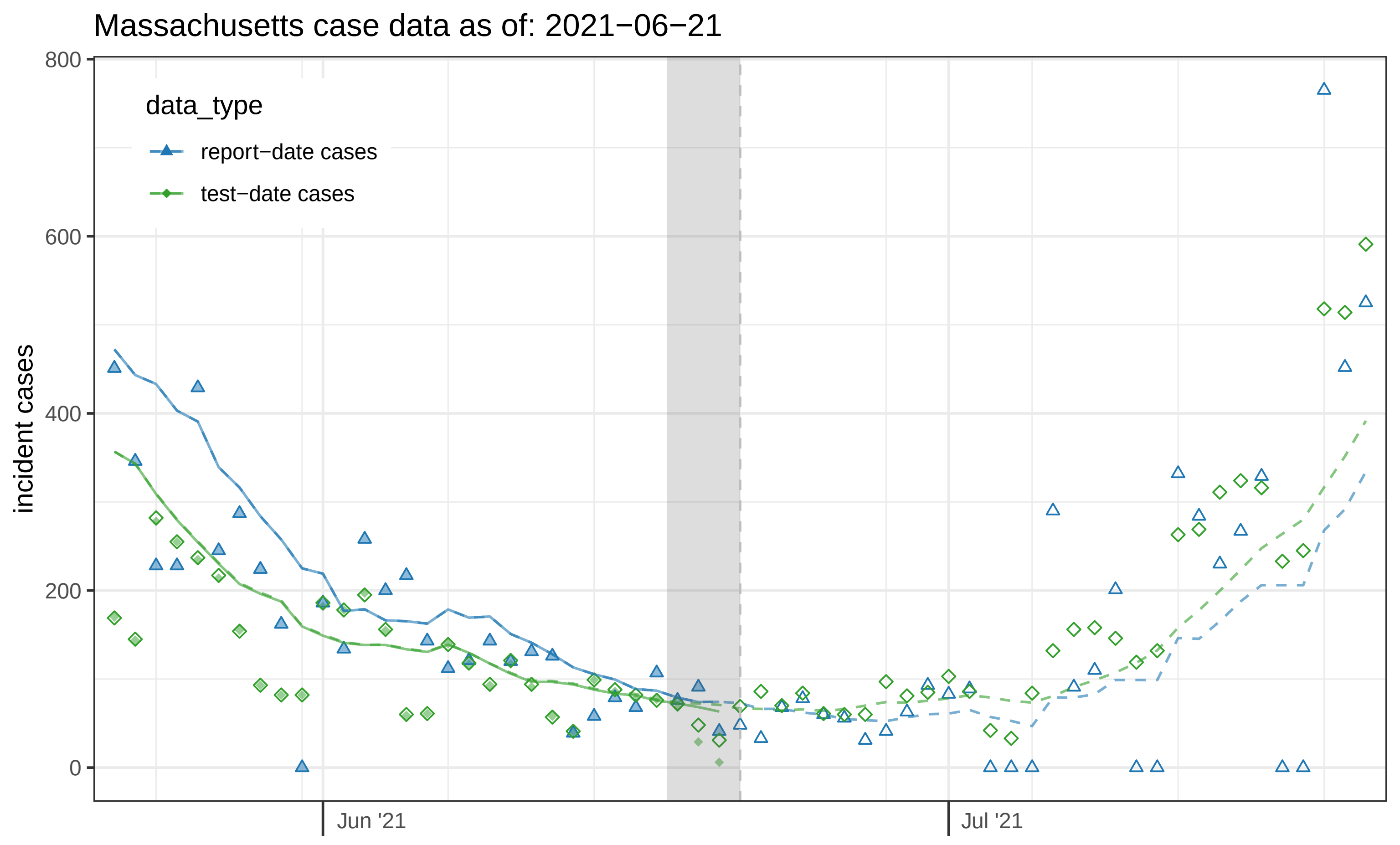
Massachusetts case data as of: 2021−06−21
data_type
report−date cases
test−date cases
incident cases
800
600
400
200
0
Jun '21
Jul '21

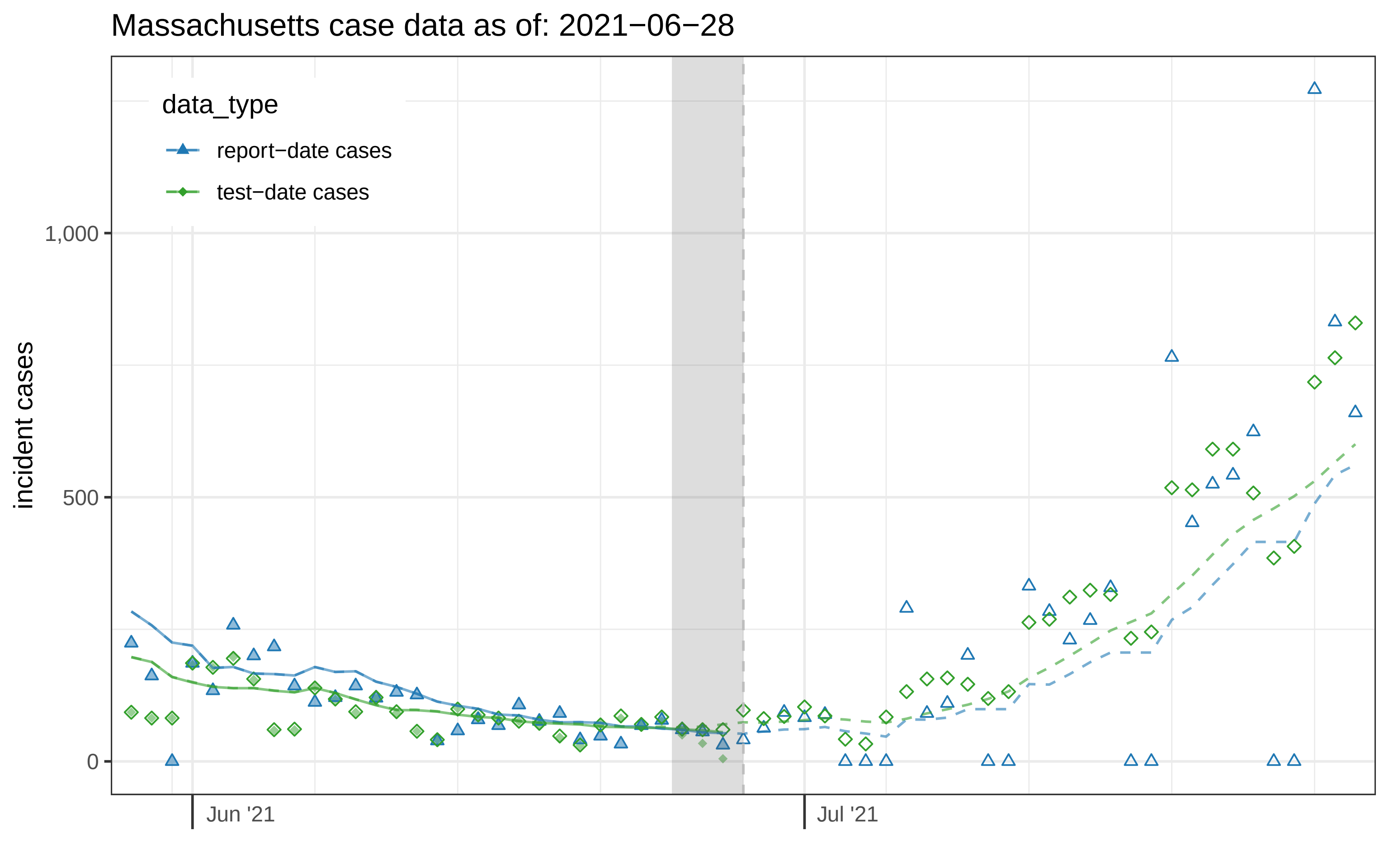

Massachusetts case data as of: 2021−06−28
data_type
report−date cases
test−date cases
incident cases
1,000
500
0
Jun '21
Jul '21

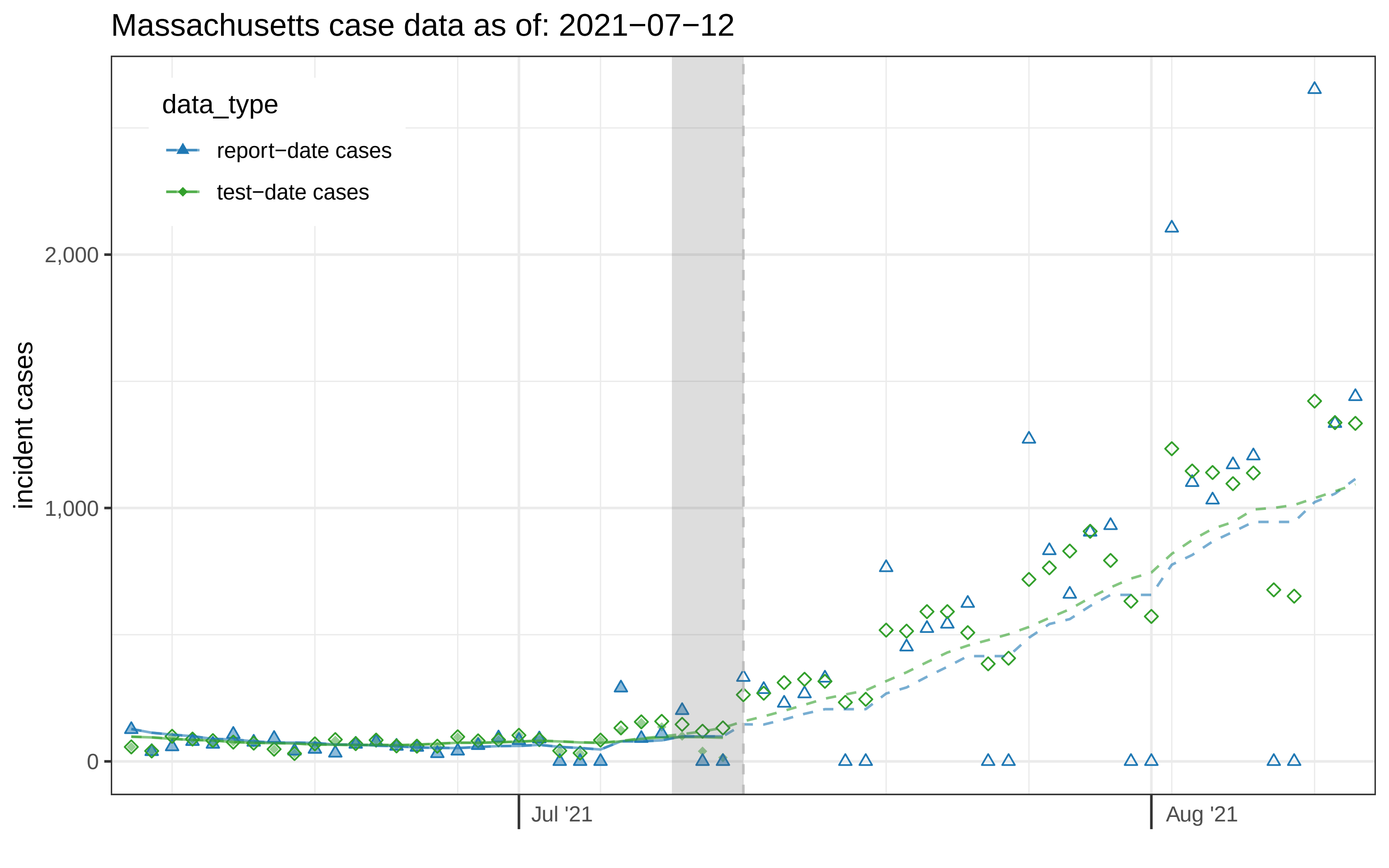

Massachusetts case data as of: 2021−07−12
data_type
report−date cases
test−date cases
incident cases
2,000
1,000
0
Jul '21
Aug '21

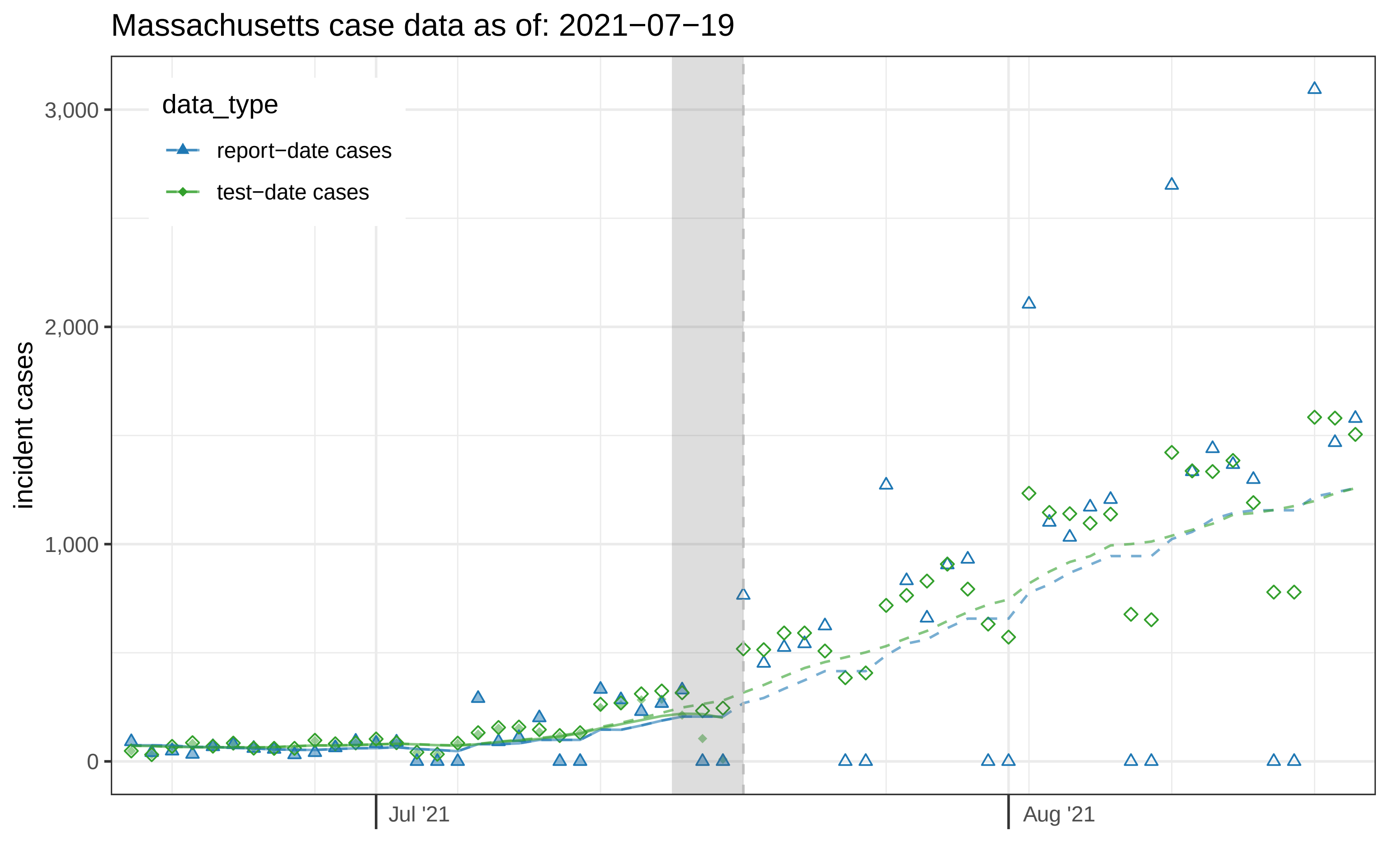

Massachusetts case data as of: 2021−07−19
data_type
report−date cases
test−date cases
incident cases
3,000
2,000
1,000
0
Jul '21
Aug '21

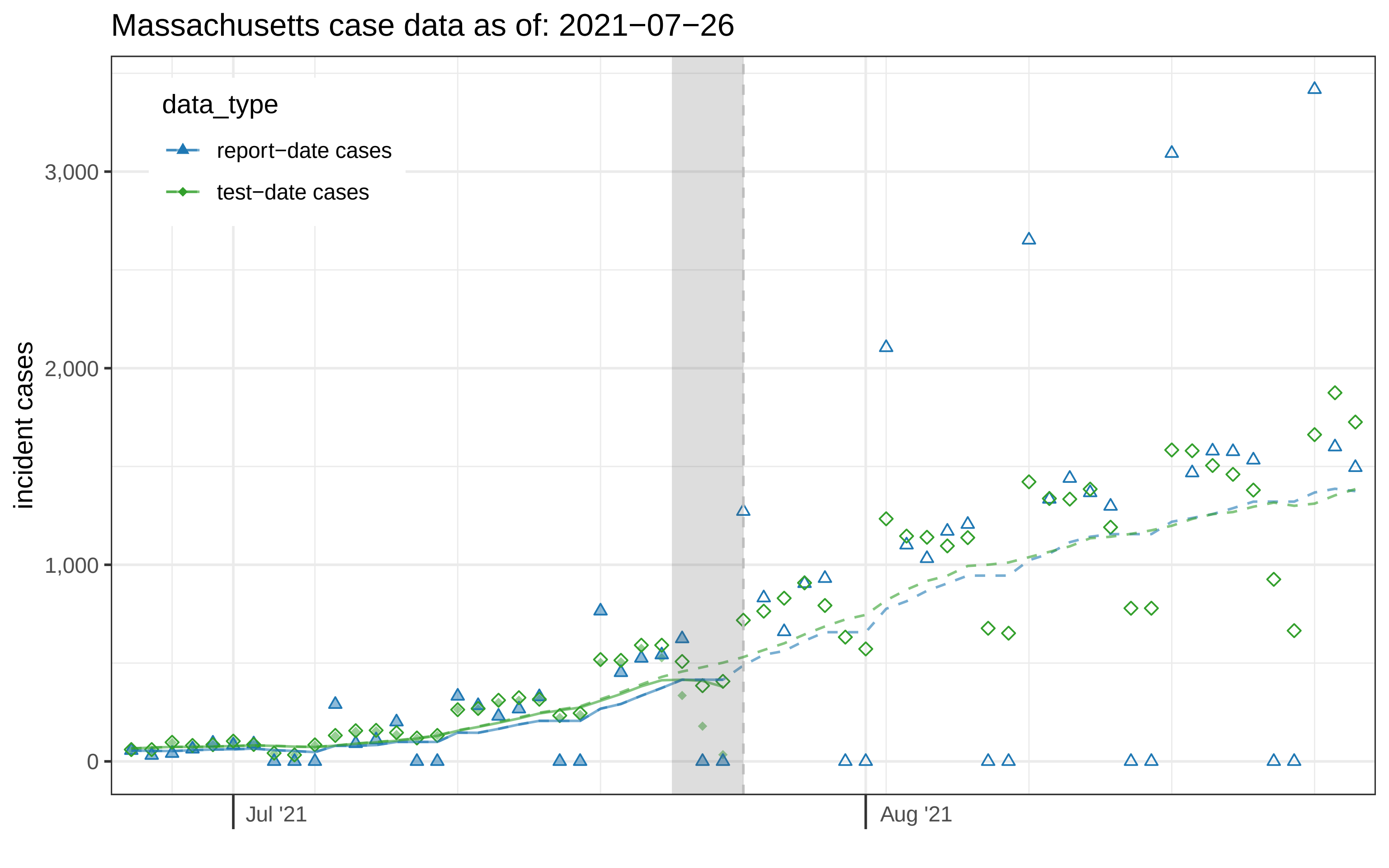
Massachusetts case data as of: 2021−07−26
data_type
report−date cases
test−date cases
incident cases
3,000
2,000
1,000
0
Jul '21
Aug '21

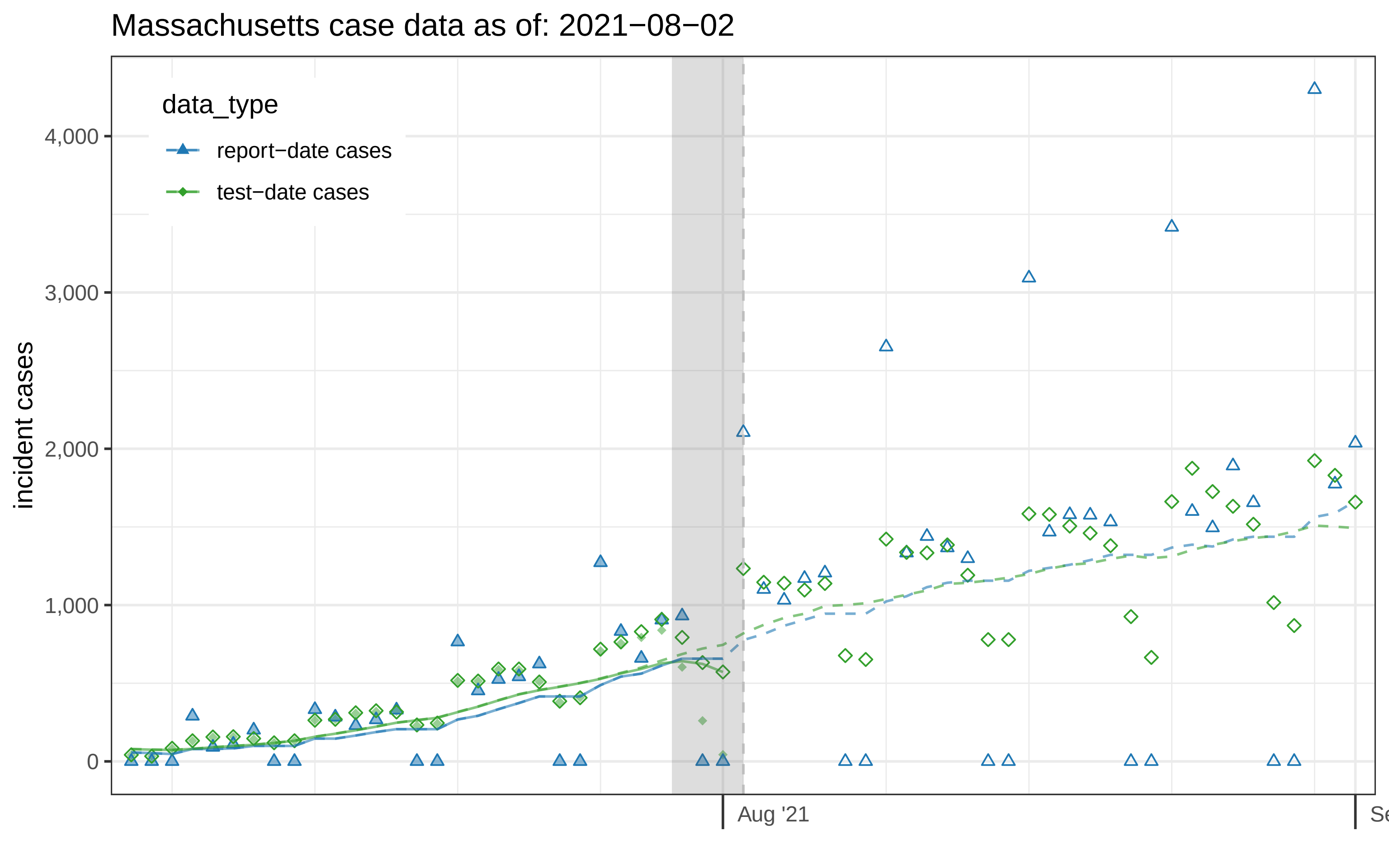

Massachusetts case data as of: 2021−08−02
data_type
report−date cases
test−date cases
incident cases
4,000
3,000
2,000
1,000
0
Aug '21

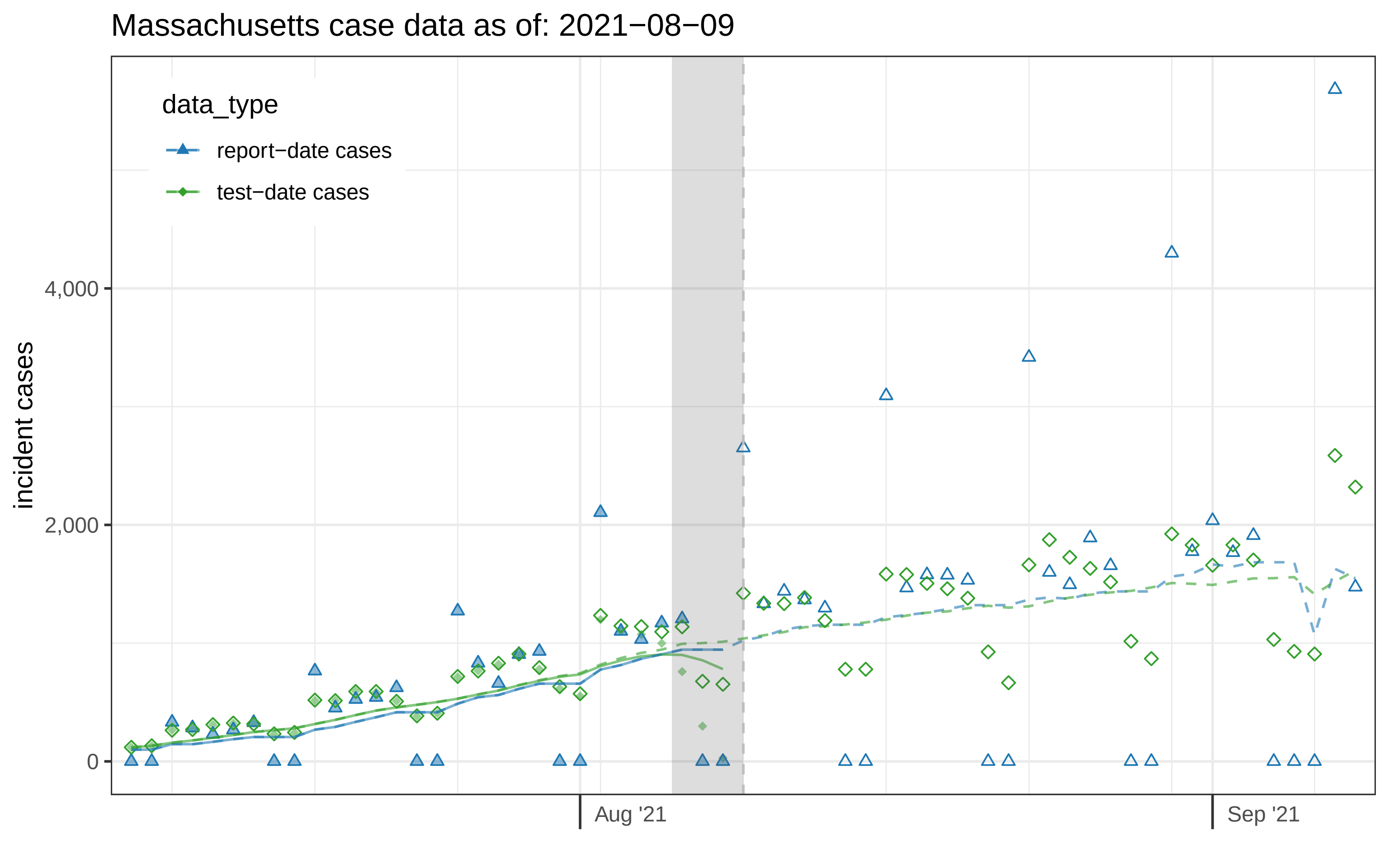

Massachusetts case data as of: 2021−08−09
data_type
report−date cases
test−date cases
incident cases
4,000
2,000
0
Aug '21
Sep '21

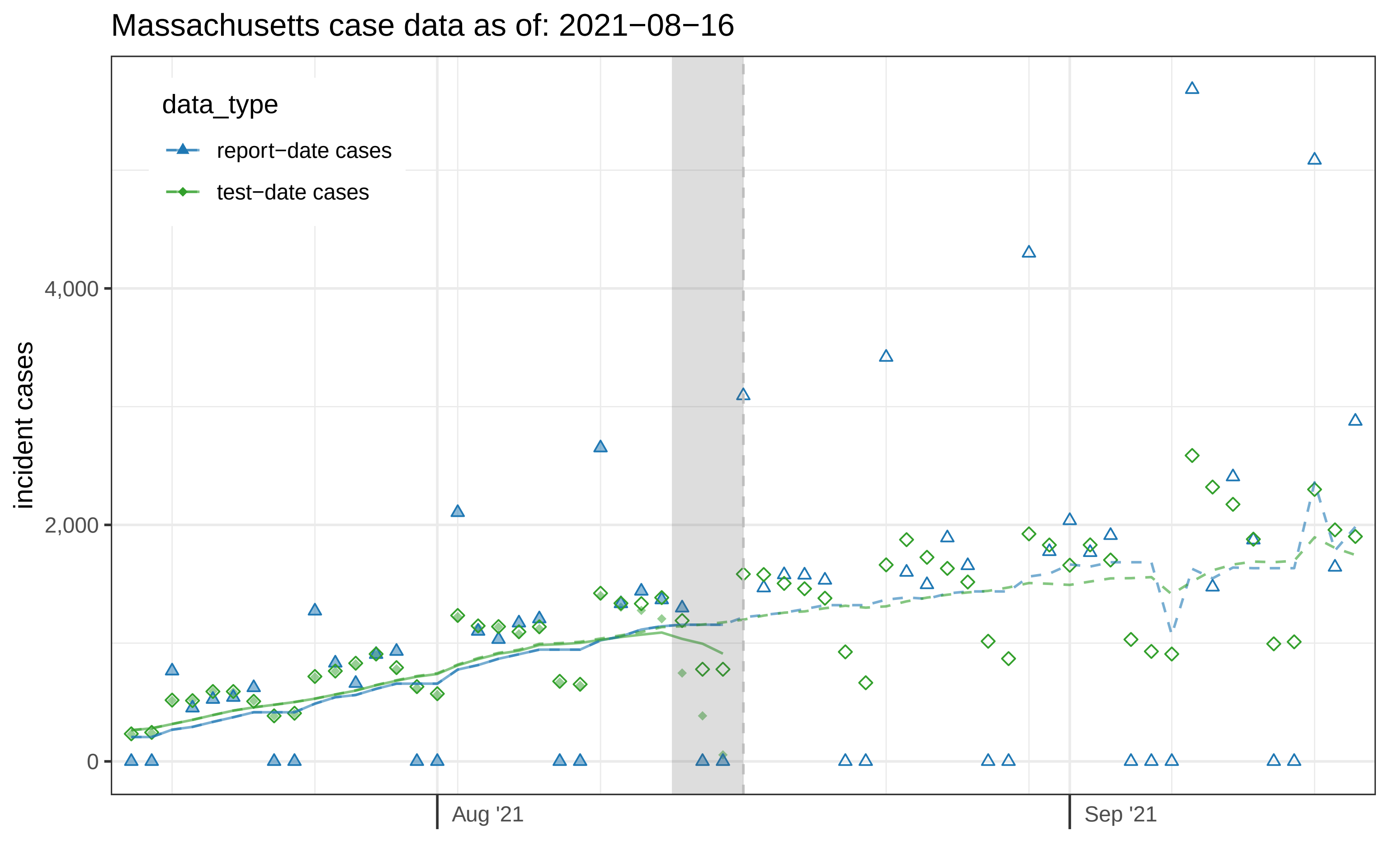

Massachusetts case data as of: 2021−08−16
data_type
report−date cases
test−date cases
incident cases
4,000
2,000
0
Aug '21
Sep '21

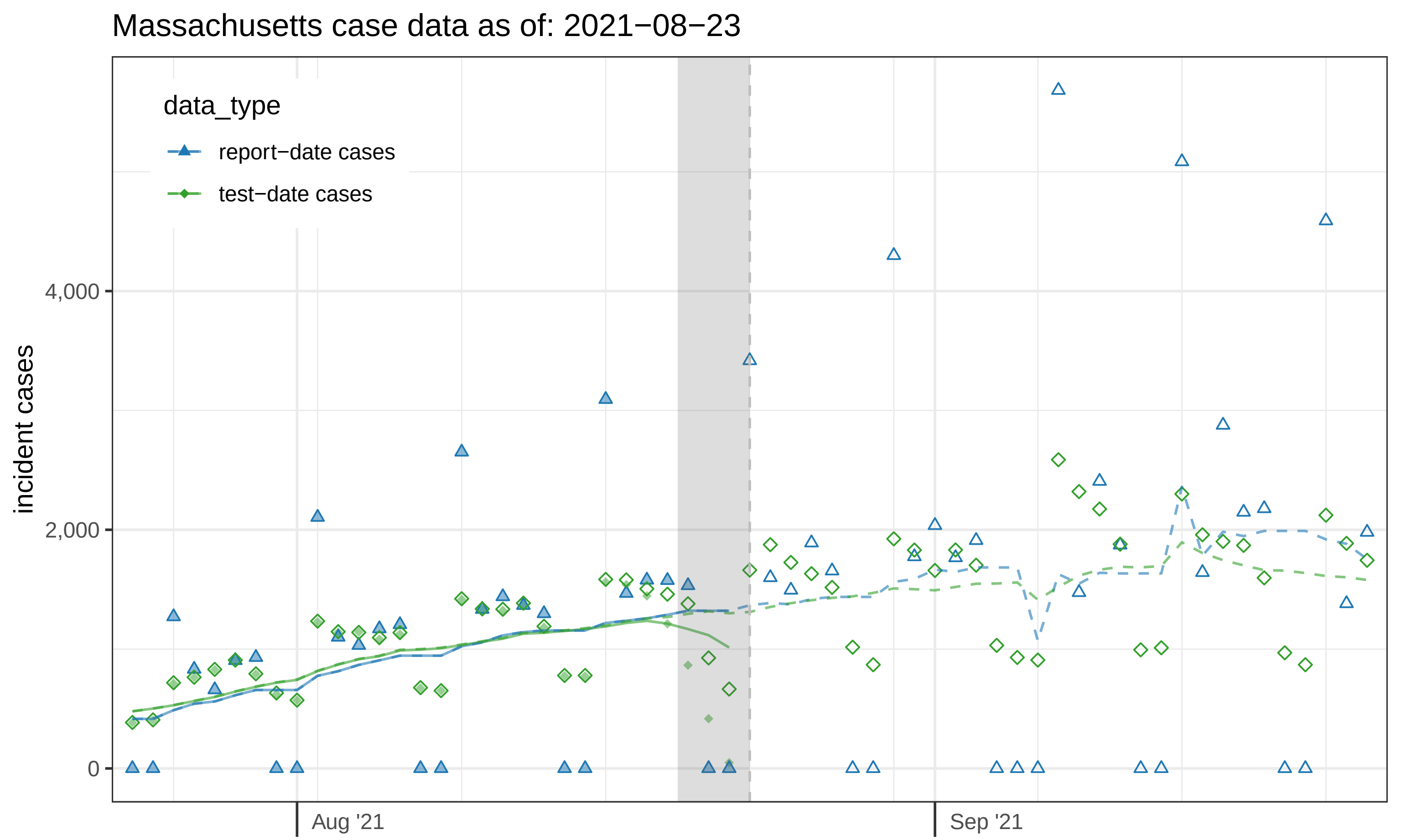
Massachusetts case data as of: 2021−08−23
data_type
report−date cases
test−date cases
incident cases
4,000
2,000
0
Aug '21
Sep '21

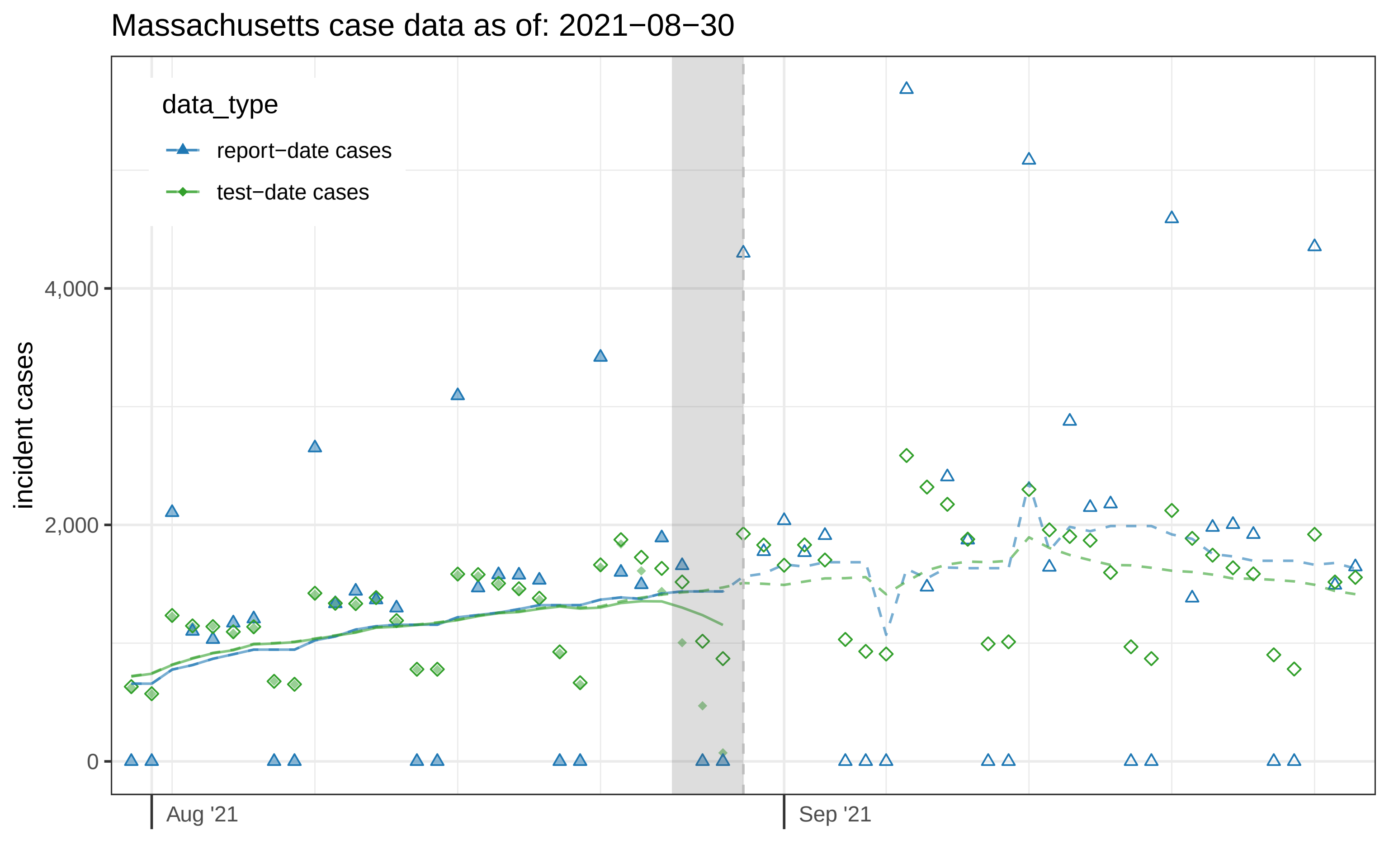
Massachusetts case data as of: 2021−08−30
data_type
report−date cases
test−date cases
incident cases
4,000
2,000
0
Aug '21
Sep '21

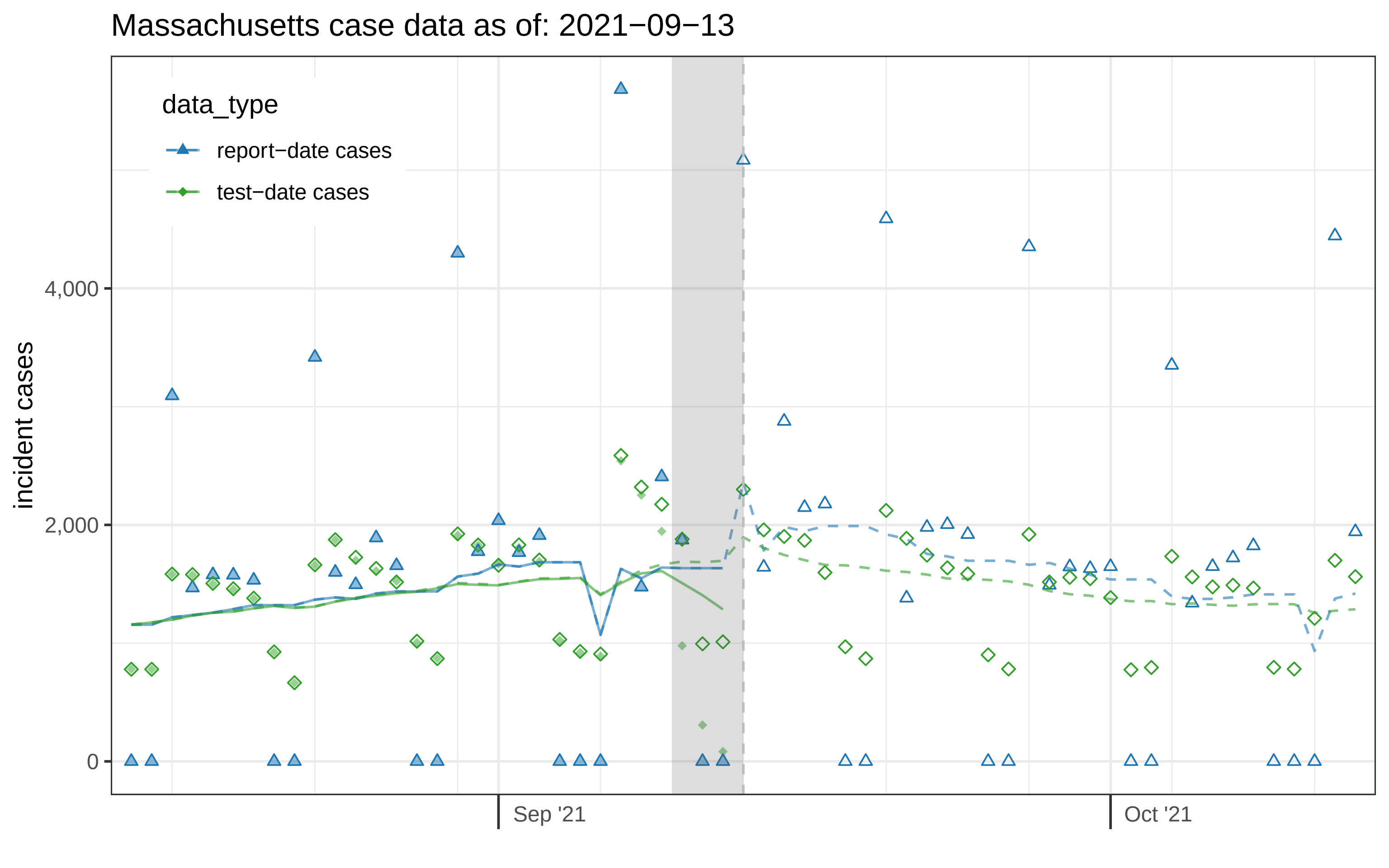
Massachusetts case data as of: 2021−09−13
data_type
report−date cases
test−date cases
incident cases
4,000
2,000
0
Sep '21
Oct '21

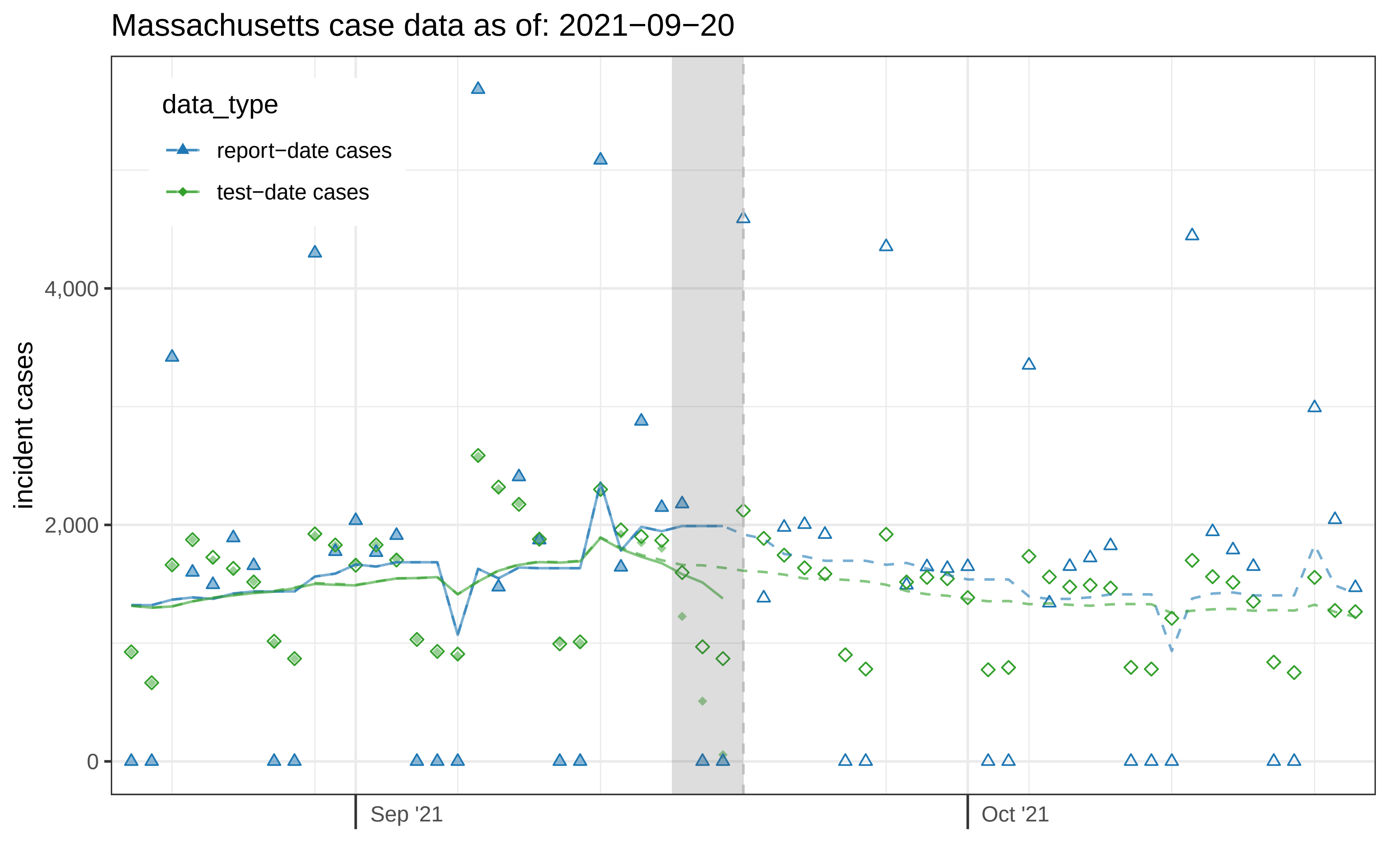
Massachusetts case data as of: 2021−09−20
data_type
report−date cases
test−date cases
incident cases
4,000
2,000
0
Sep '21
Oct '21

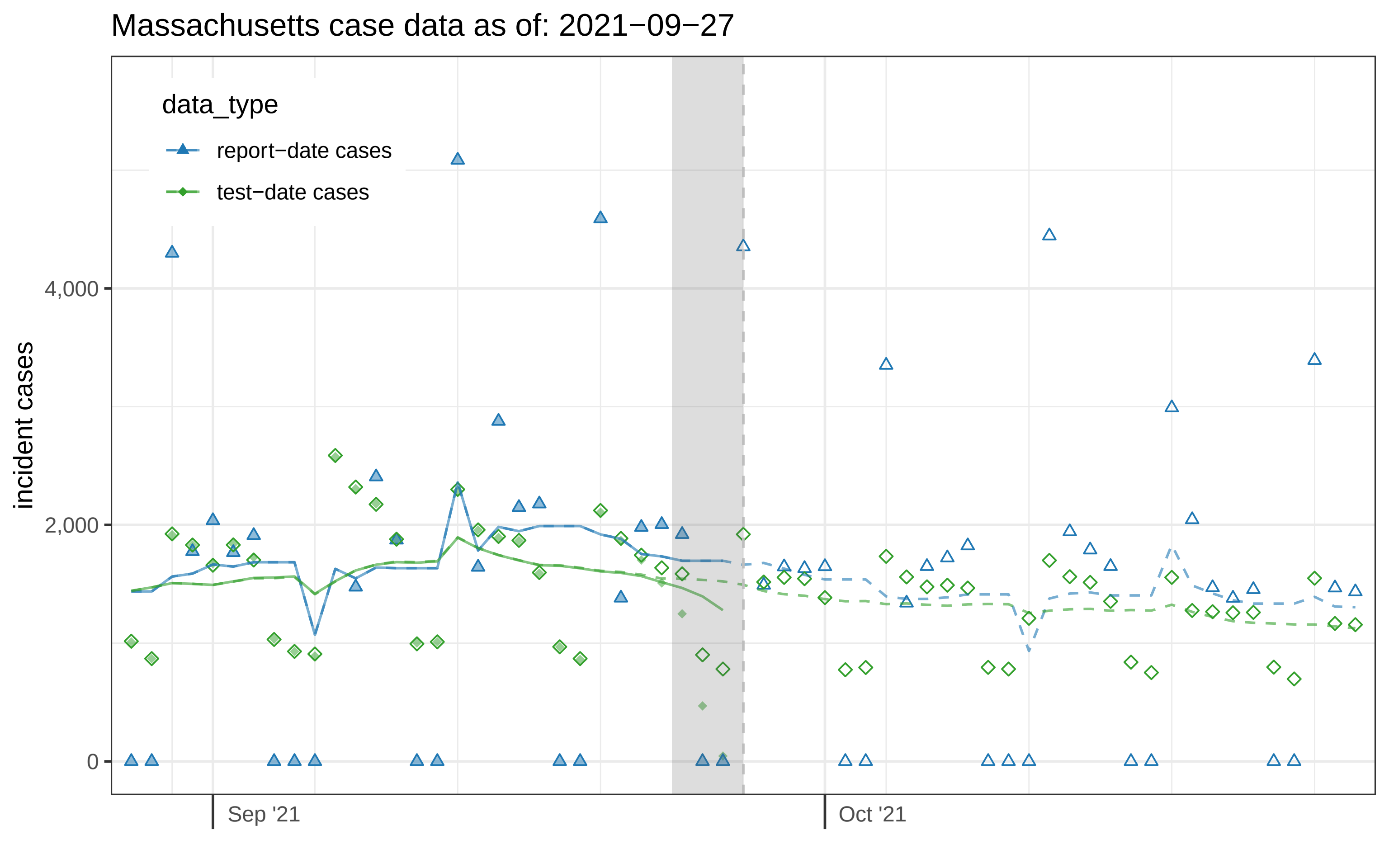

Massachusetts case data as of: 2021−09−27
data_type
report−date cases
test−date cases
incident cases
4,000
2,000
0
Sep '21
Oct '21

Massachusetts case data as of: 2021−10−04

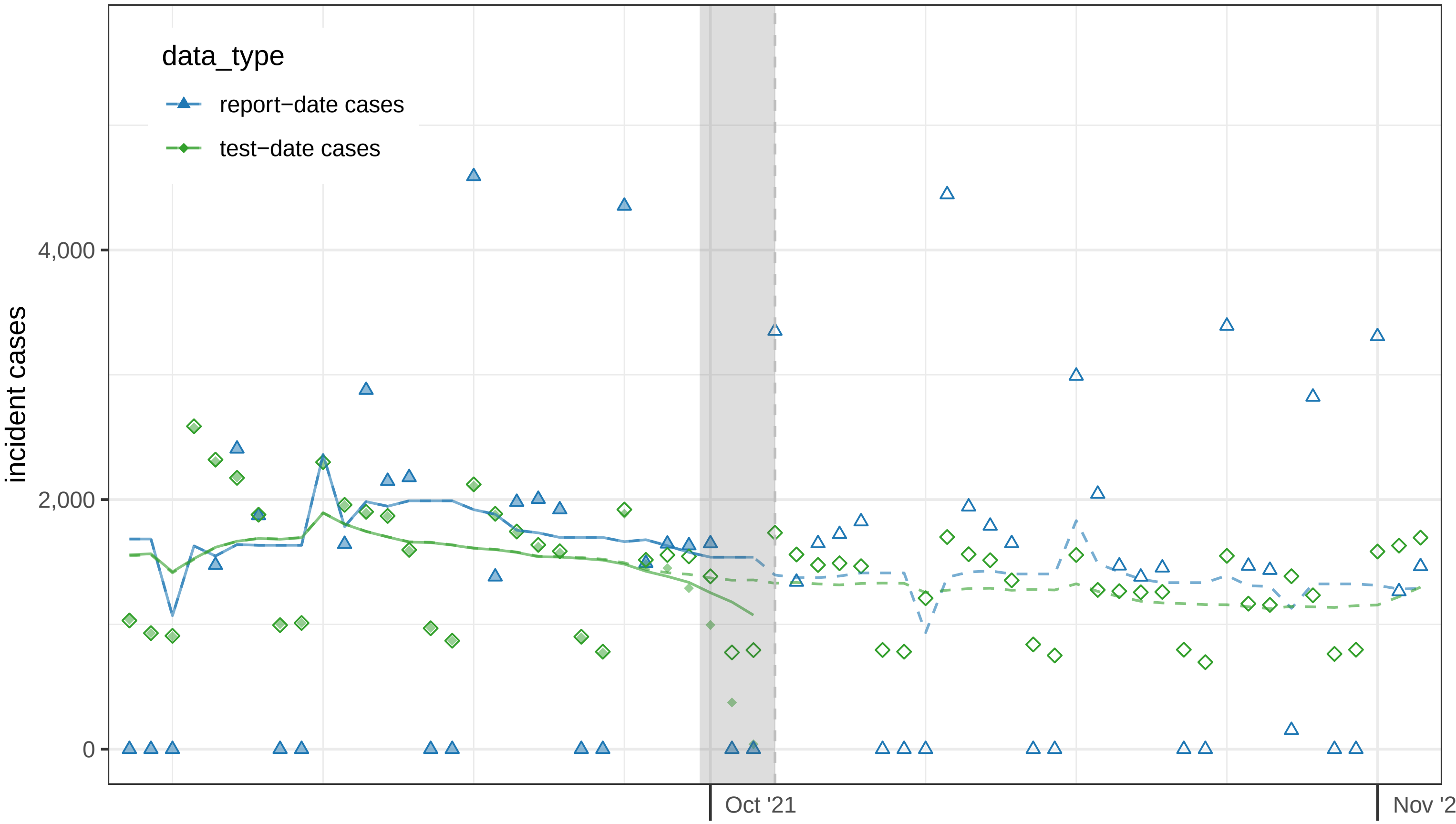

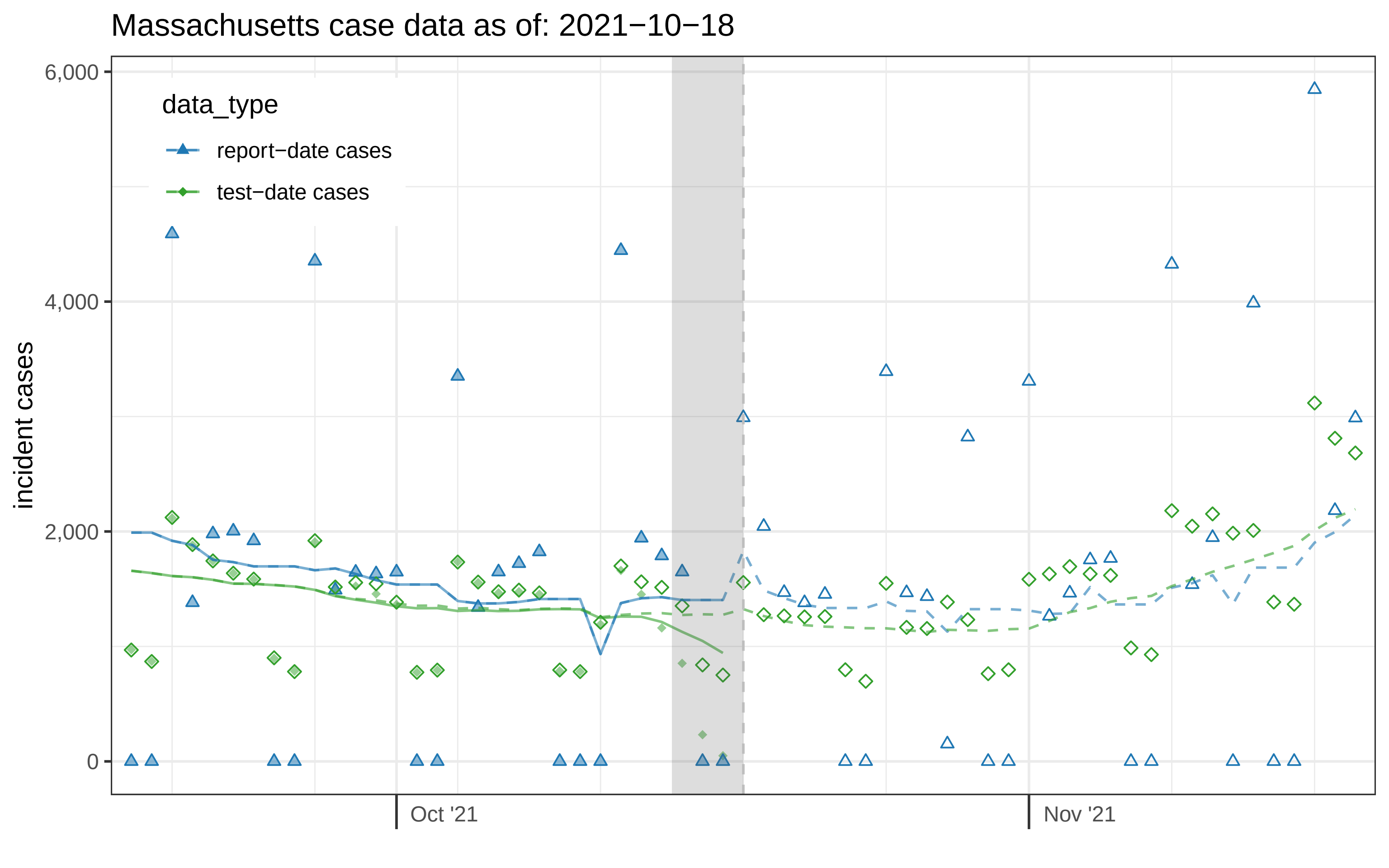

Massachusetts case data as of: 2021−10−18
data_type
report−date cases
test−date cases
incident cases
6,000
4,000
2,000
0
Oct '21
Nov '21

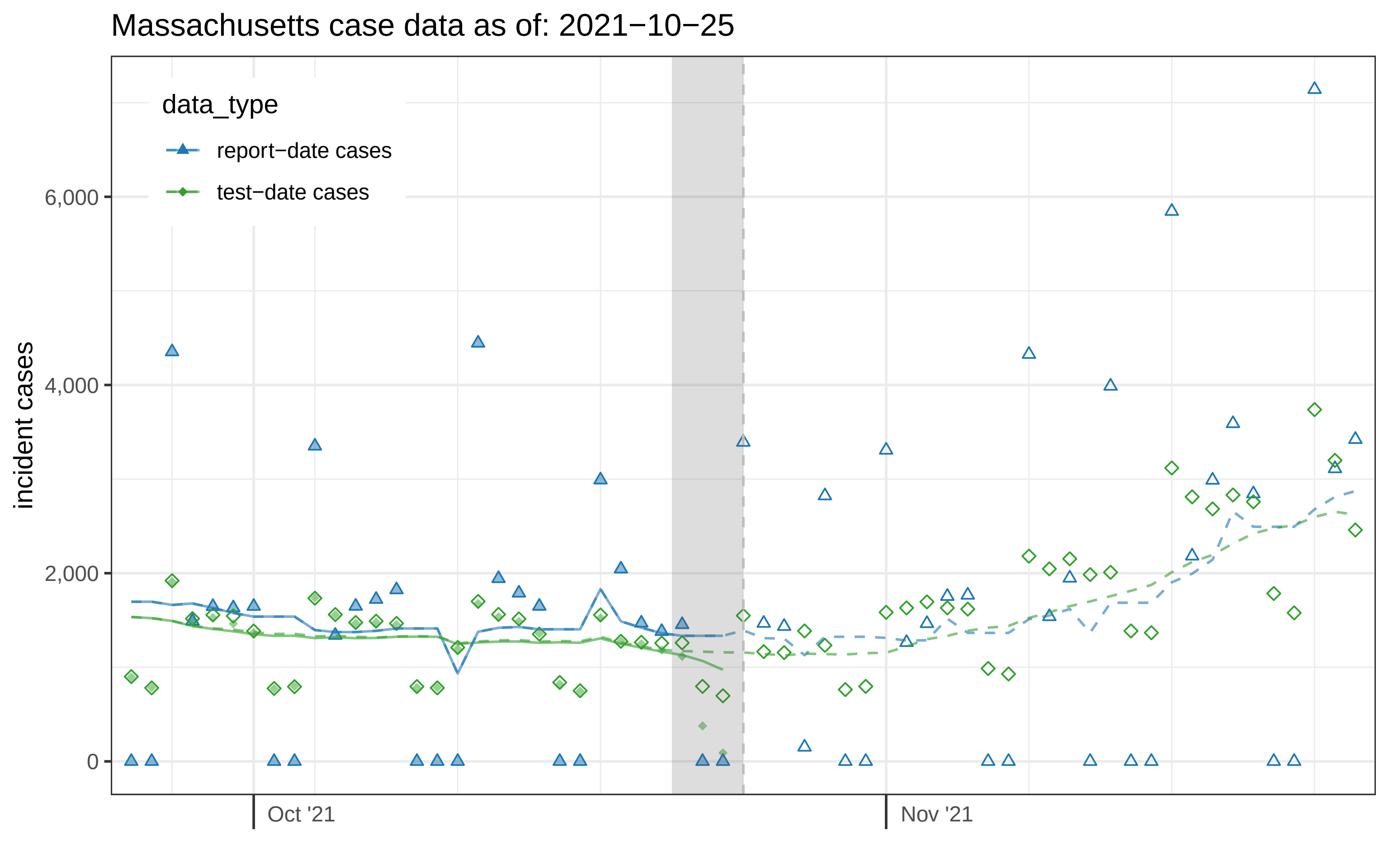

Massachusetts case data as of: 2021−10−25
data_type
report−date cases
test−date cases
incident cases
6,000
4,000
2,000
0
Oct '21
Nov '21

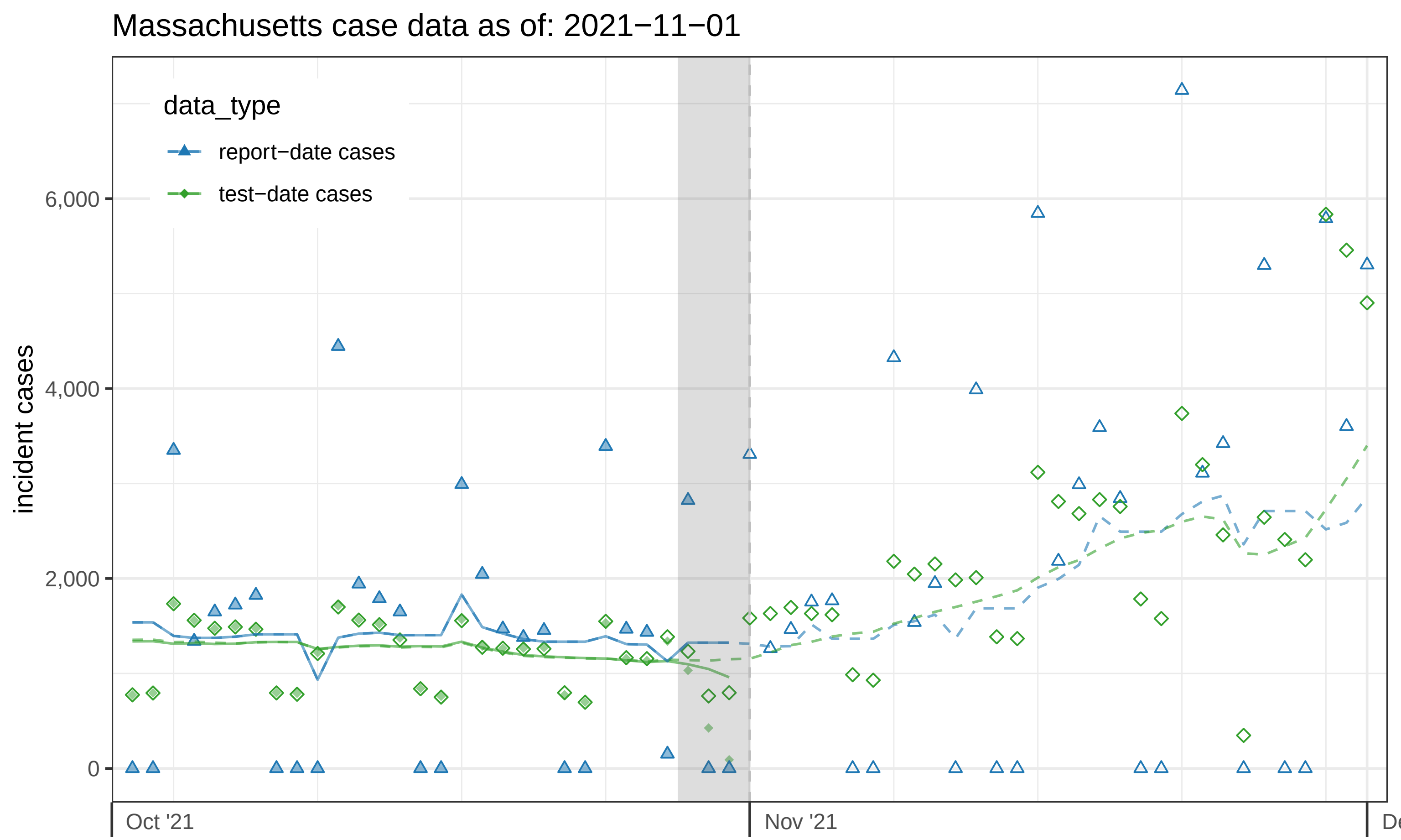
Massachusetts case data as of: 2021−11−01
data_type
report−date cases
test−date cases
incident cases
6,000
4,000
2,000
0
Oct '21
Nov '21

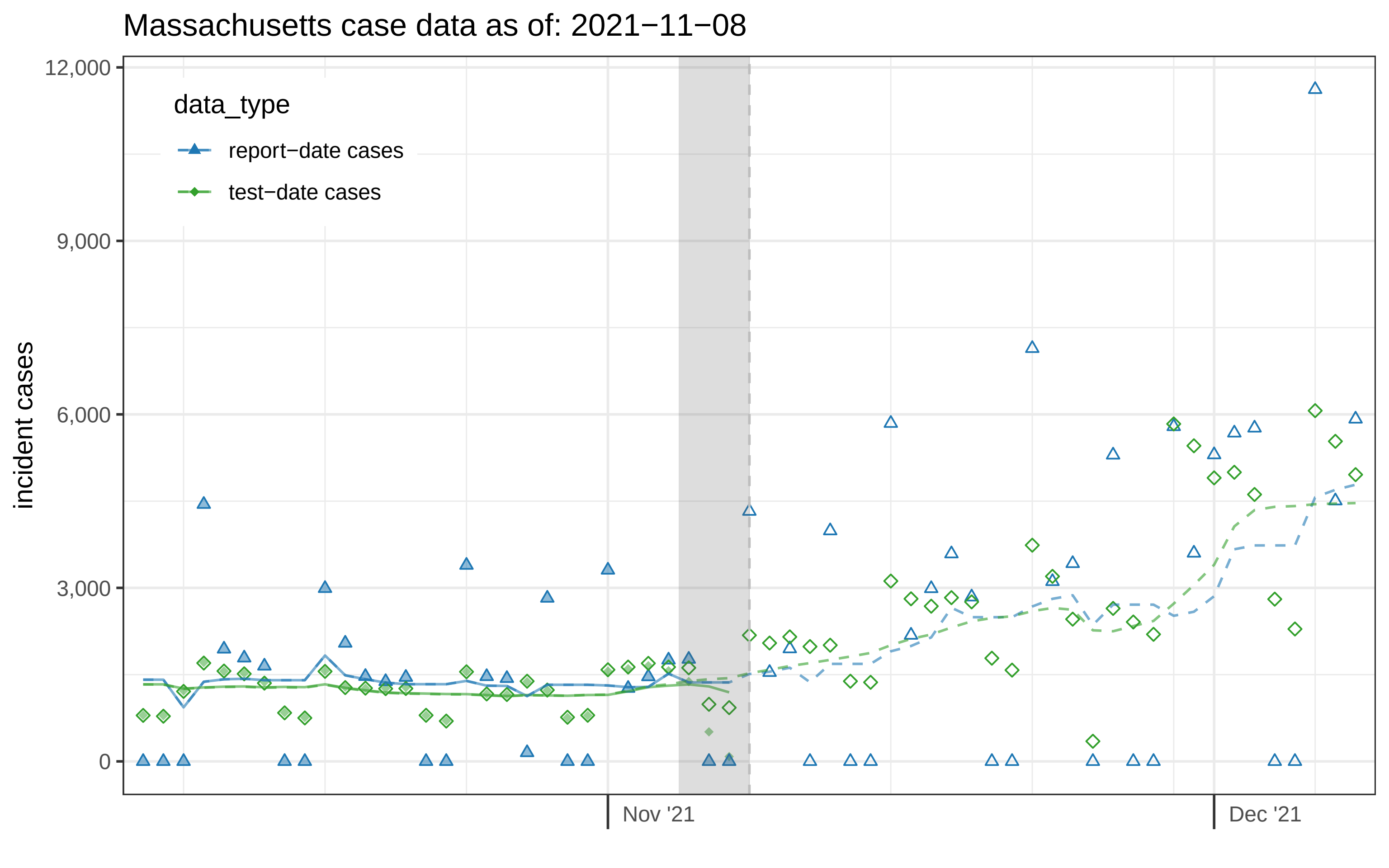

Massachusetts case data as of: 2021−11−08
data_type
report−date cases
test−date cases
incident cases
12,000
9,000
6,000
3,000
0
Nov '21
Dec '21

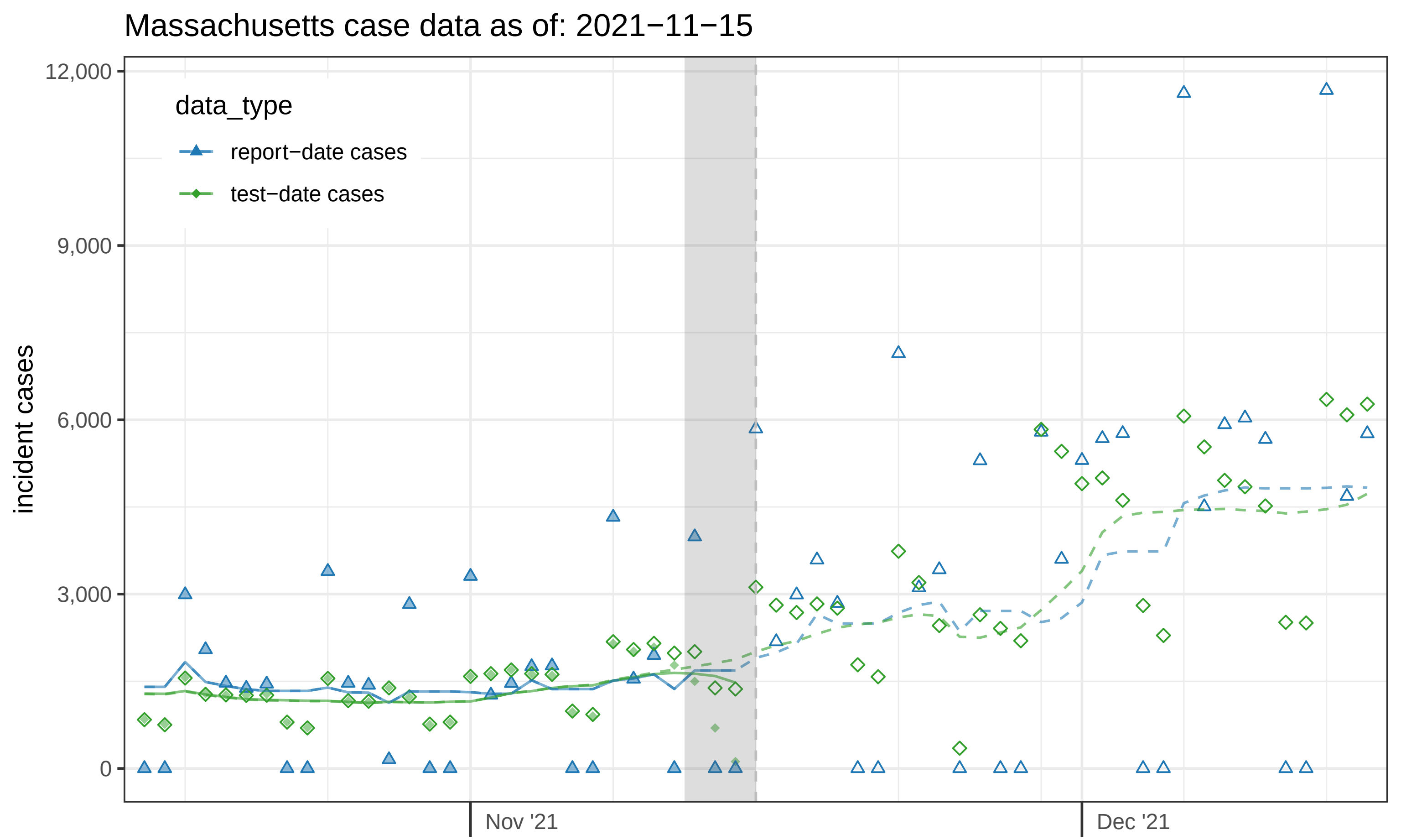
Massachusetts case data as of: 2021−11−15
data_type
report−date cases
test−date cases
incident cases
12,000
9,000
6,000
3,000
0
Nov '21
Dec '21

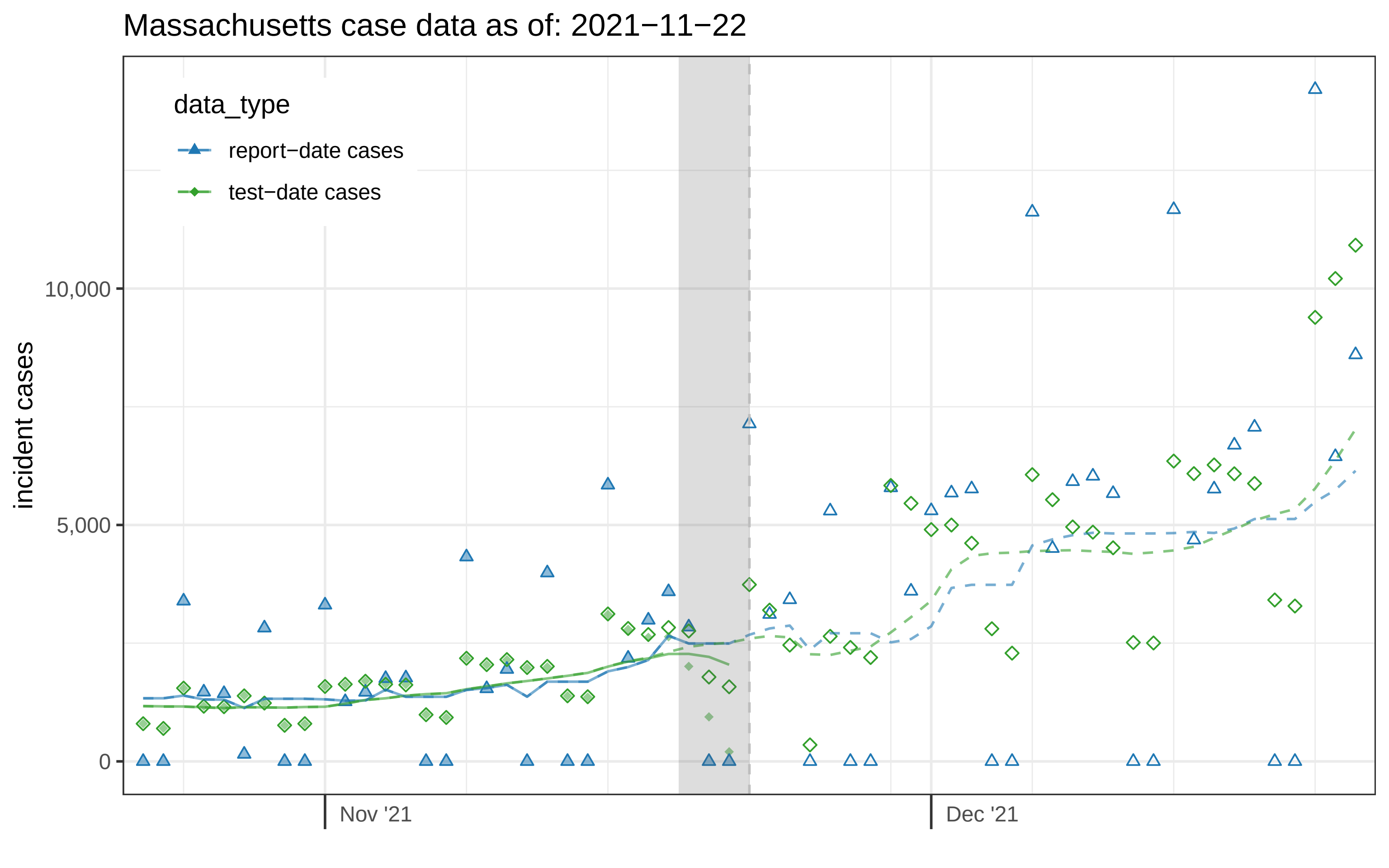

Massachusetts case data as of: 2021−11−22
data_type
report−date cases
test−date cases
incident cases
10,000
5,000
0
Nov '21
Dec '21

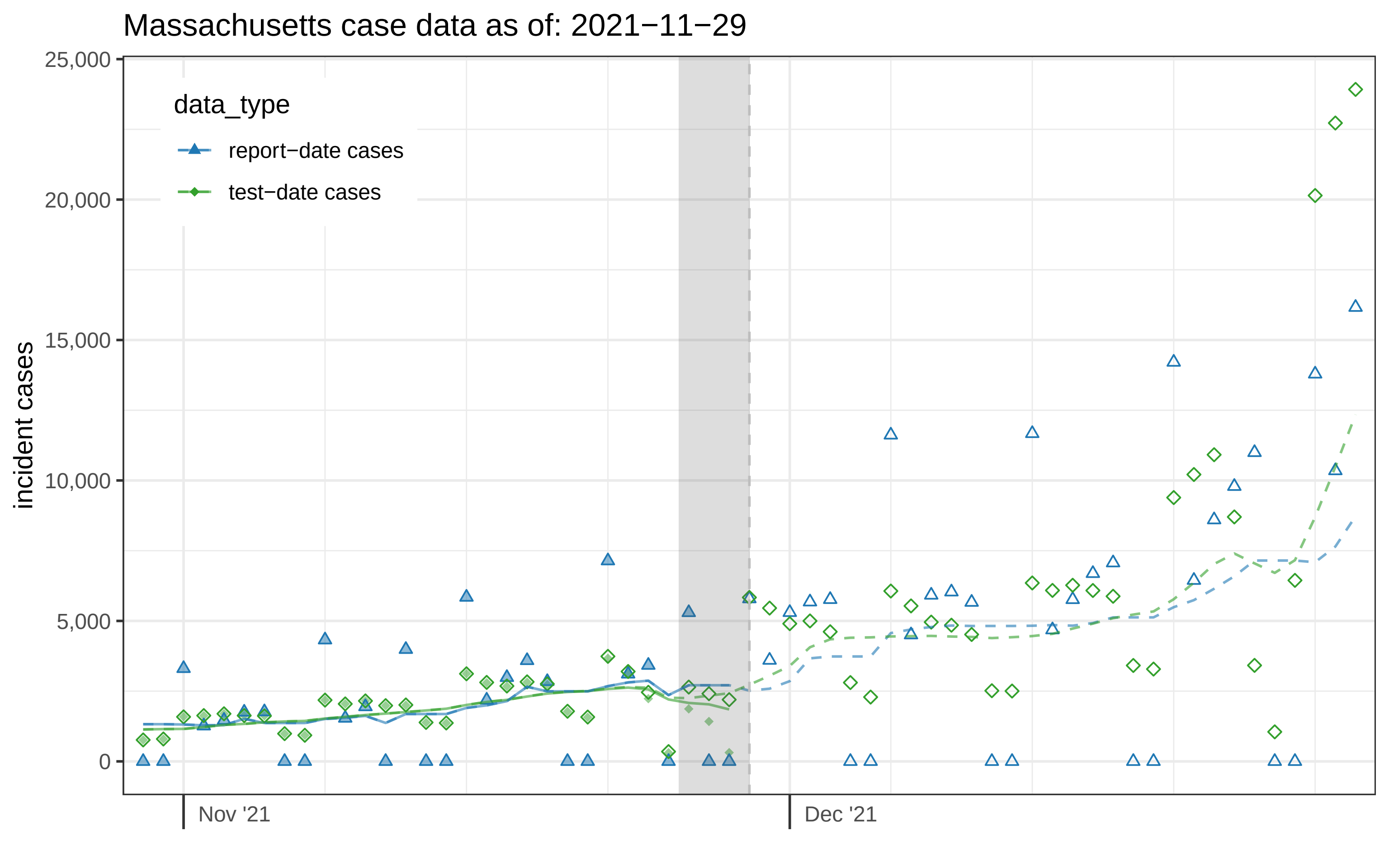
Massachusetts case data as of: 2021−11−29
data_type
report−date cases
test−date cases
incident cases
25,000
20,000
15,000
10,000
5,000
0
Nov '21
Dec '21

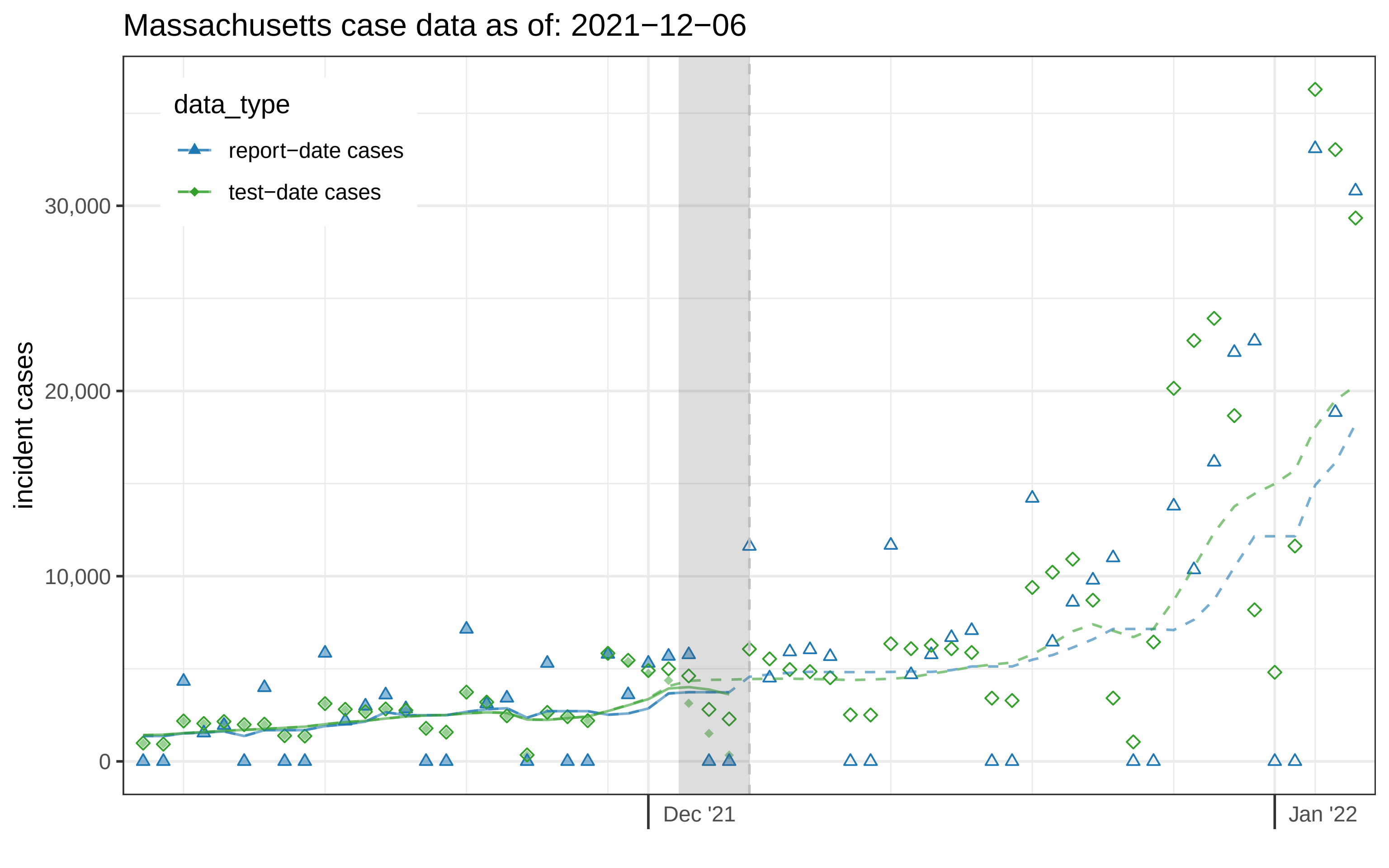

Massachusetts case data as of: 2021−12−06
data_type
report−date cases
test−date cases
incident cases
30,000
20,000
10,000
0
Dec '21
Jan '22

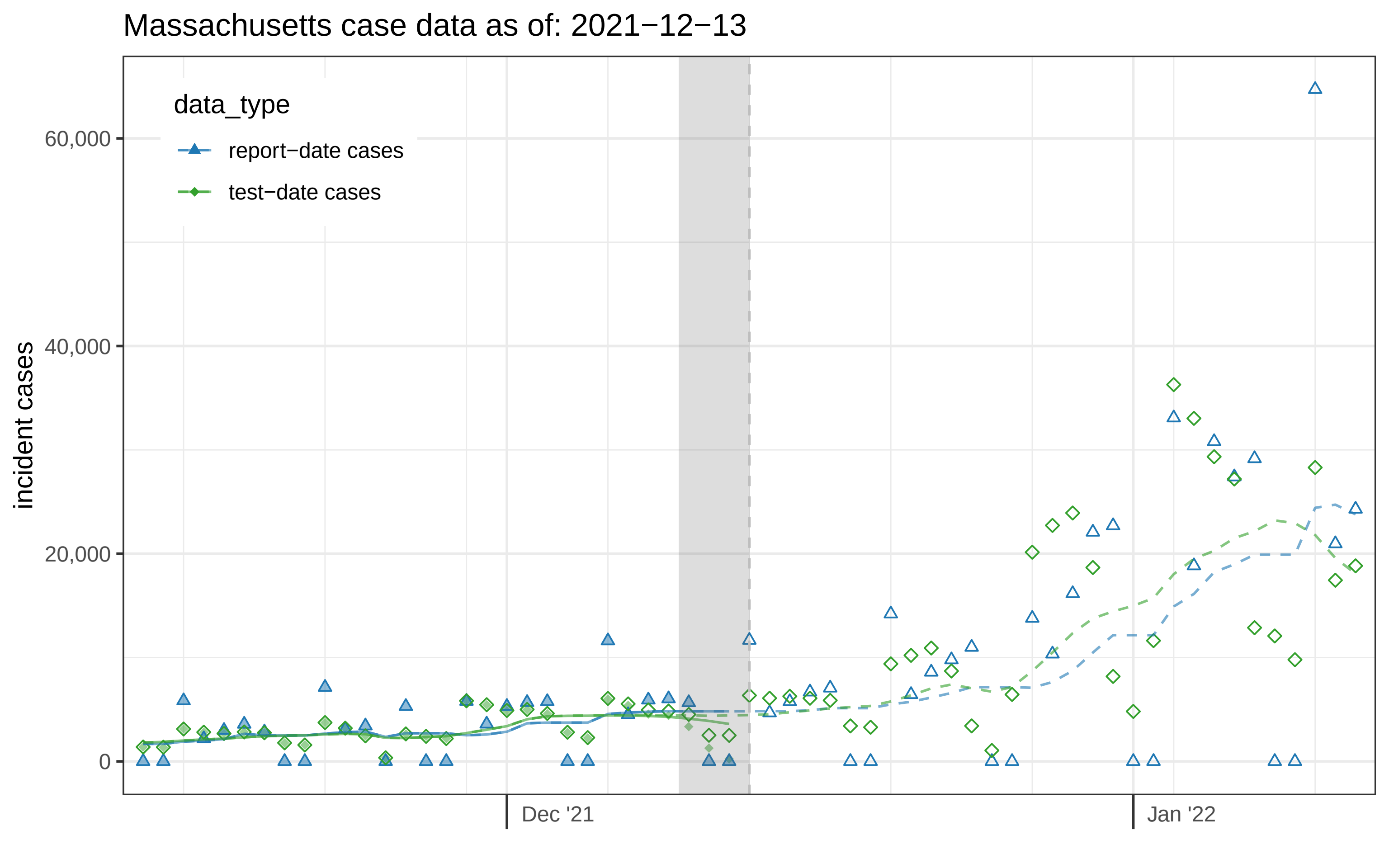
Massachusetts case data as of: 2021−12−13
data_type
report−date cases
test−date cases
60,000
40,000
20,000
0
incident cases
Dec '21
Jan '22

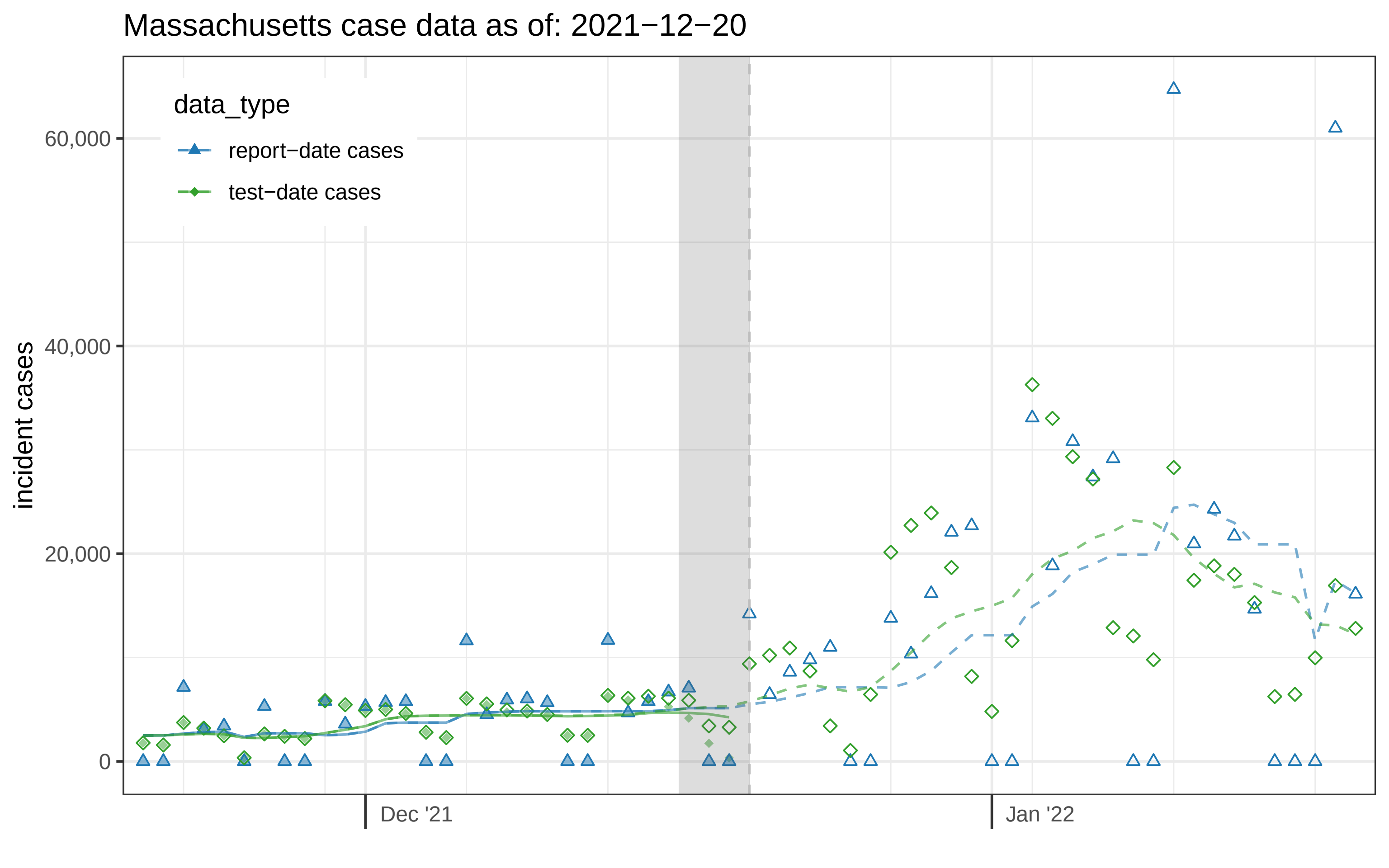

Massachusetts case data as of: 2021−12−20
data_type
report−date cases
test−date cases
incident cases
60,000
40,000
20,000
0
Dec '21
Jan '22

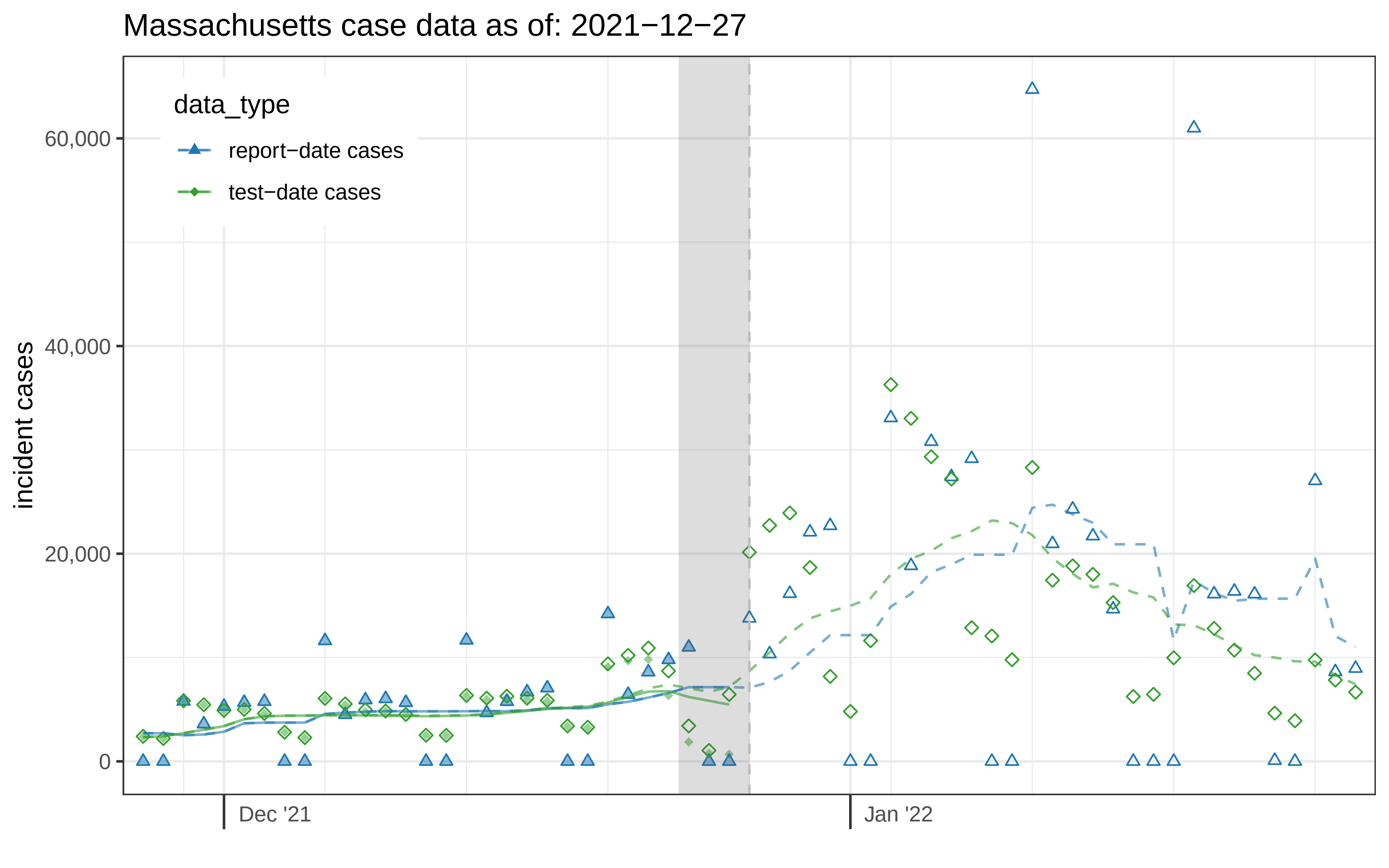

Massachusetts case data as of: 2021−12−27
data_type
report−date cases
test−date cases
60,000
40,000
20,000
0
incident cases
Dec '21
Jan '22

Massachusetts case data as of: 2022−01−03

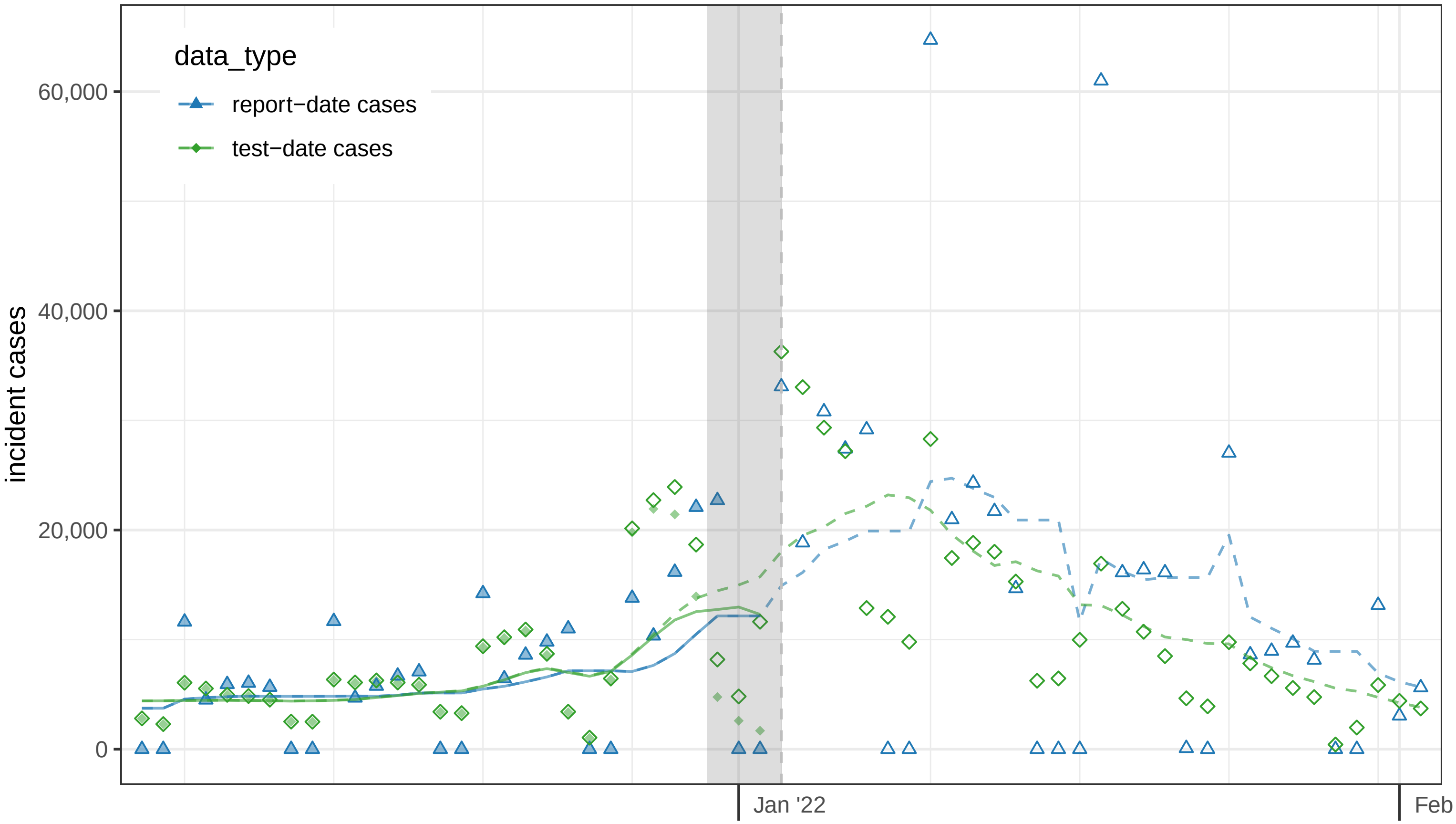

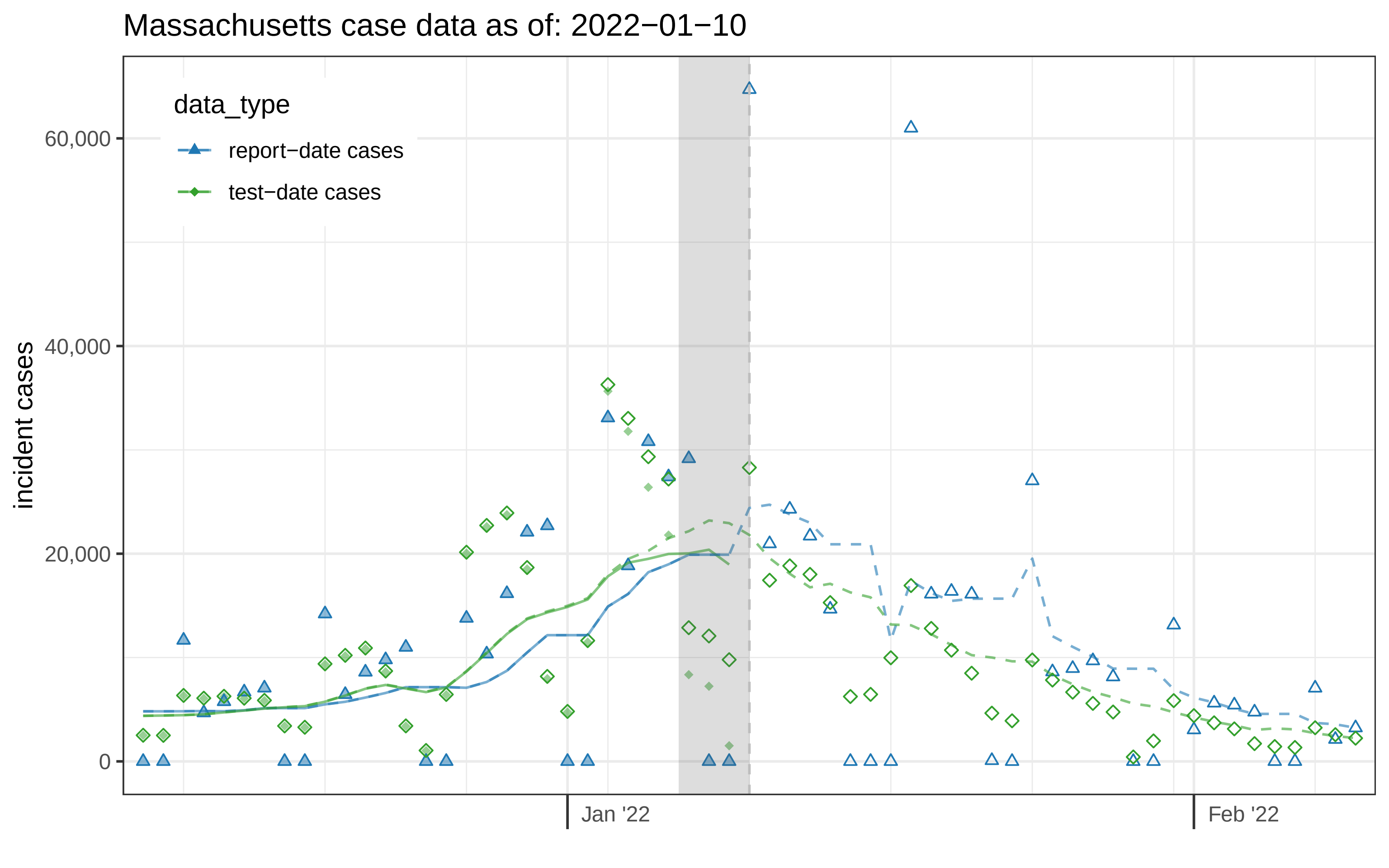

Massachusetts case data as of: 2022−01−10
data_type
report−date cases
test−date cases
incident cases
60,000
40,000
20,000
0
Jan '22
Feb '22

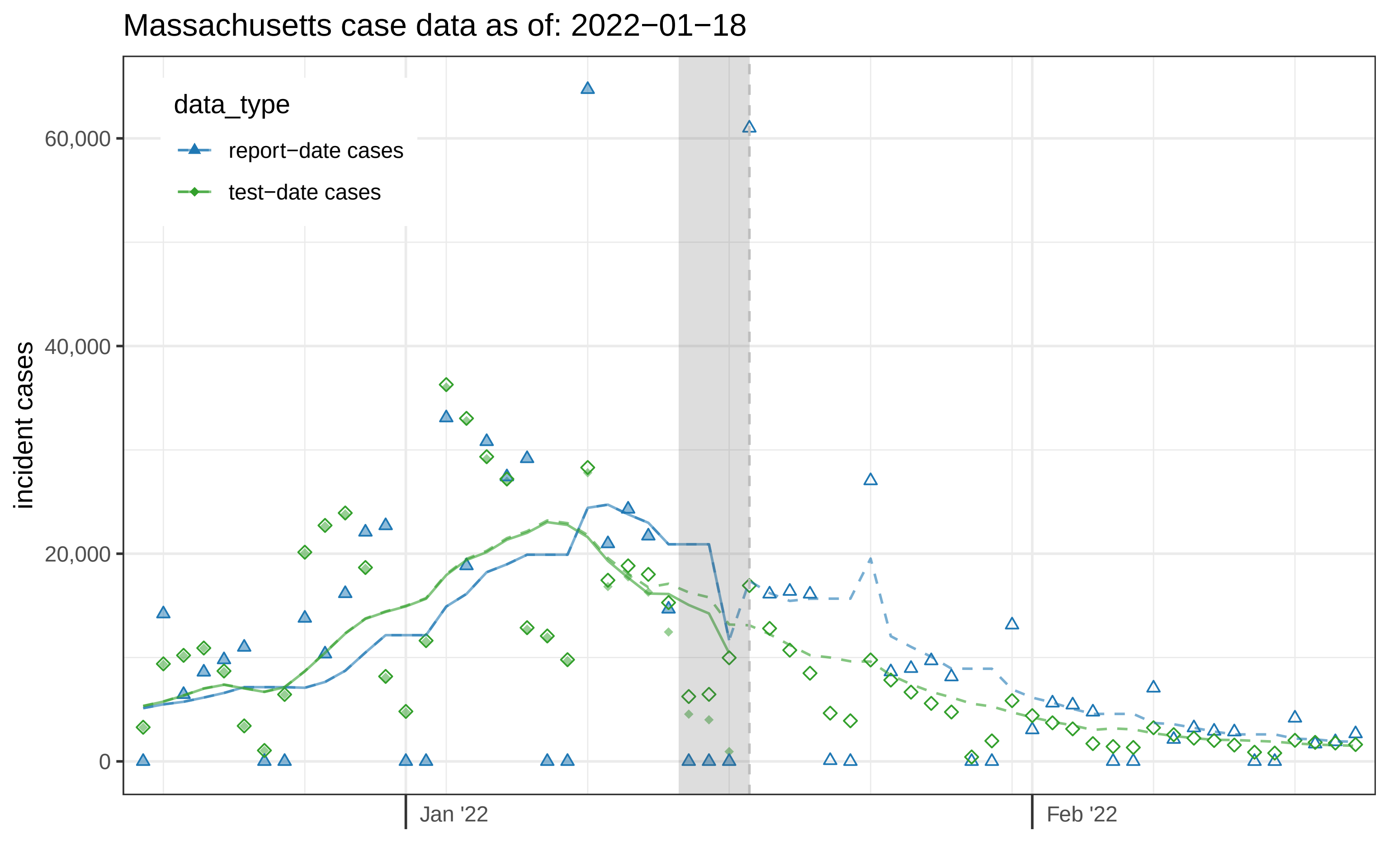

Massachusetts case data as of: 2022−01−18
data_type
report−date cases
test−date cases
incident cases
60,000
40,000
20,000
0
Jan '22
Feb '22

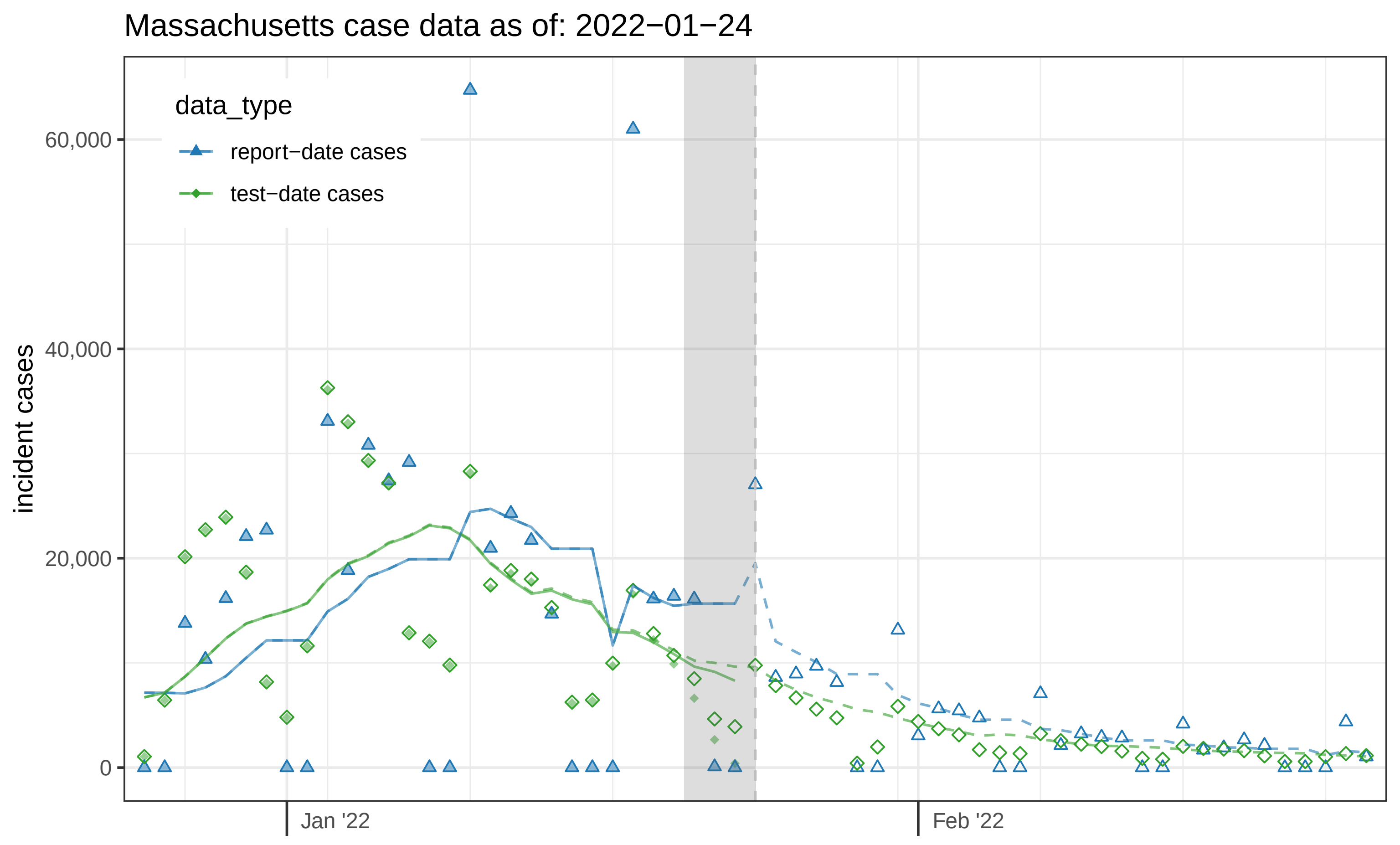
Massachusetts case data as of: 2022−01−24
data_type
report−date cases
test−date cases
incident cases
60,000
40,000
20,000
0
Jan '22
Feb '22

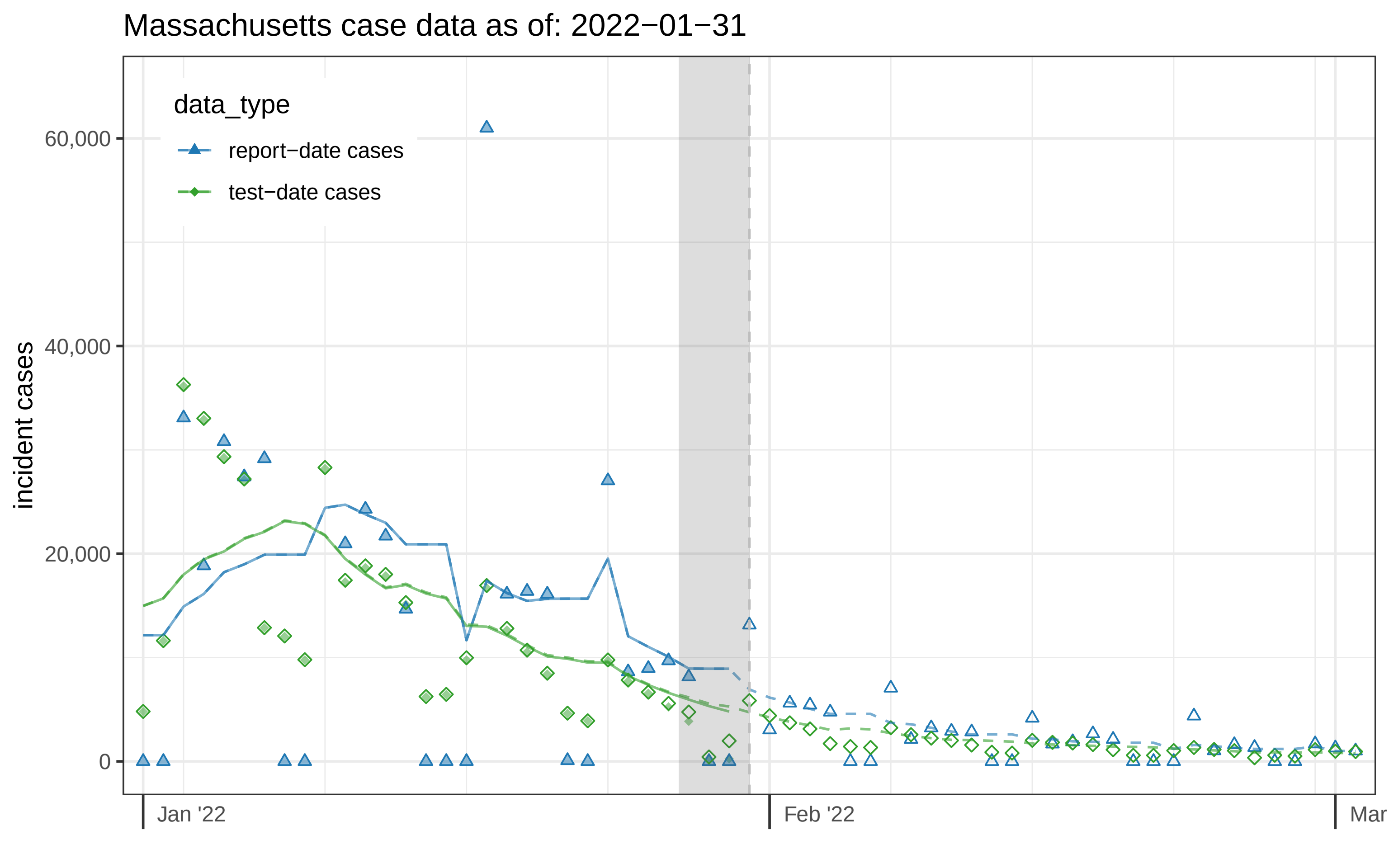

Massachusetts case data as of: 2022−01−31
data_type
report−date cases
test−date cases
60,000
40,000
20,000
0
incident cases
Jan '22
Feb '22
Mar

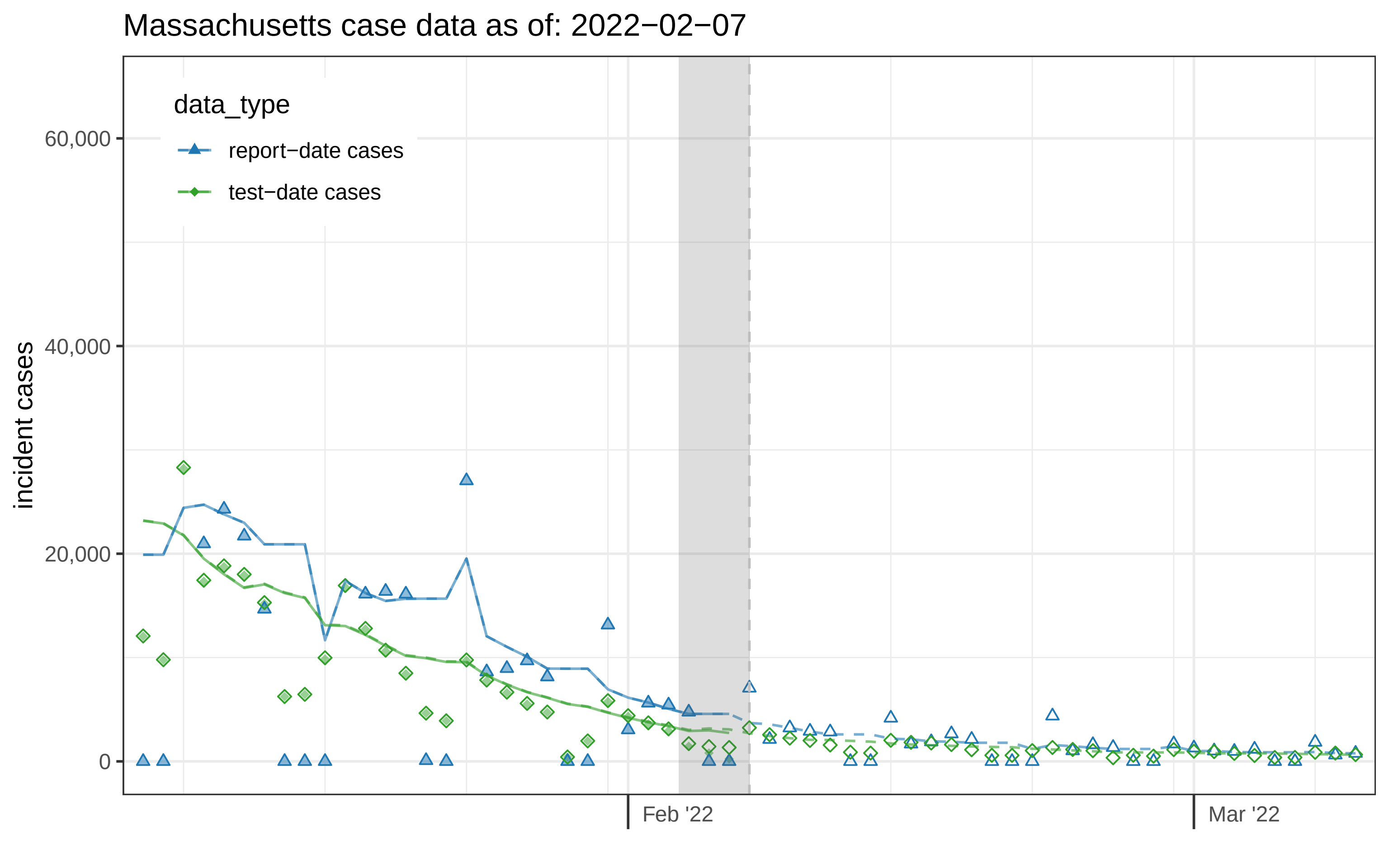

Massachusetts case data as of: 2022−02−07
data_type
report−date cases
test−date cases
incident cases
60,000
40,000
20,000
0
Feb '22
Mar '22

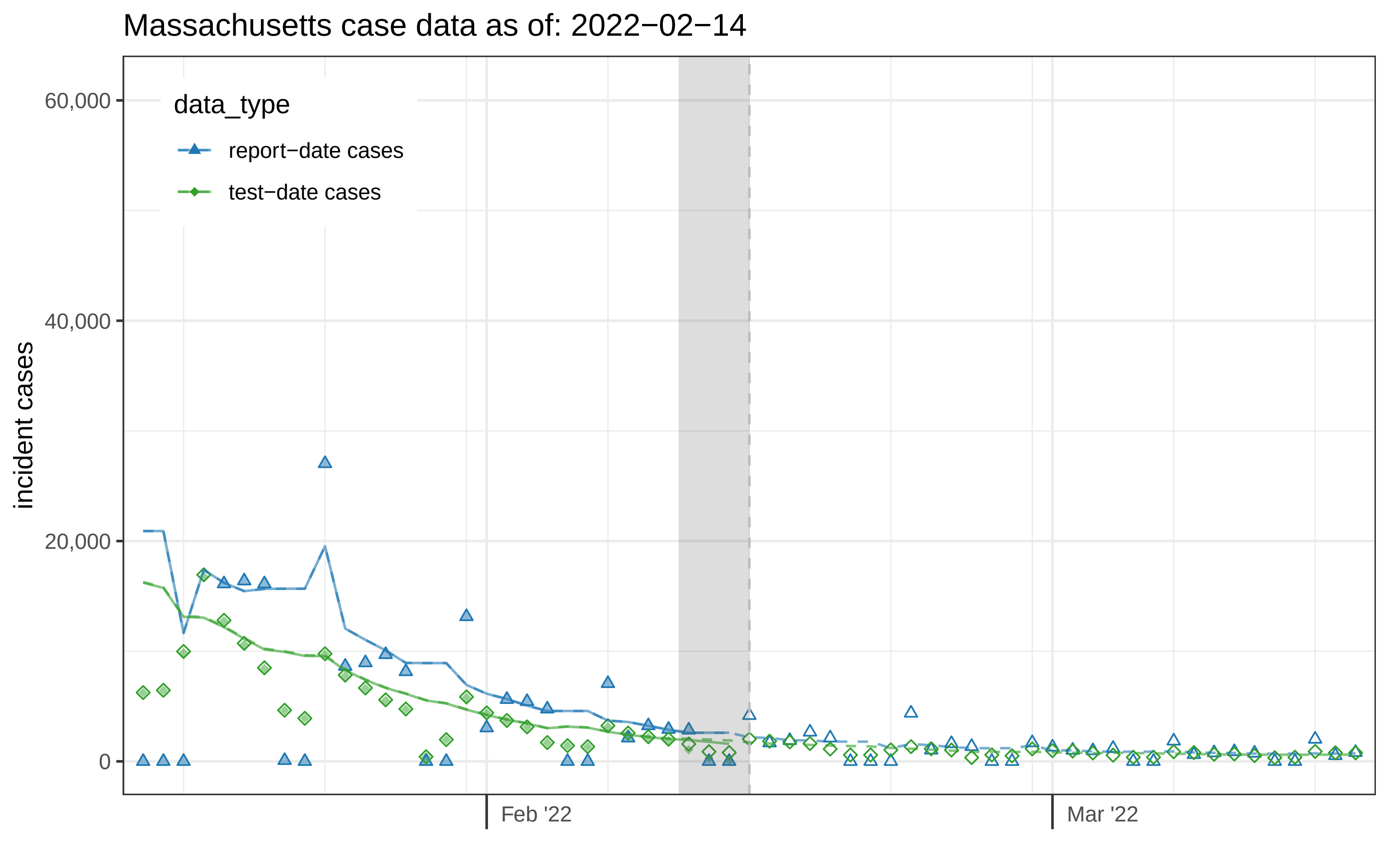

Massachusetts case data as of: 2022−02−14
data_type
report−date cases
test−date cases
60,000
40,000
20,000
0
incident cases
Feb '22
Mar '22

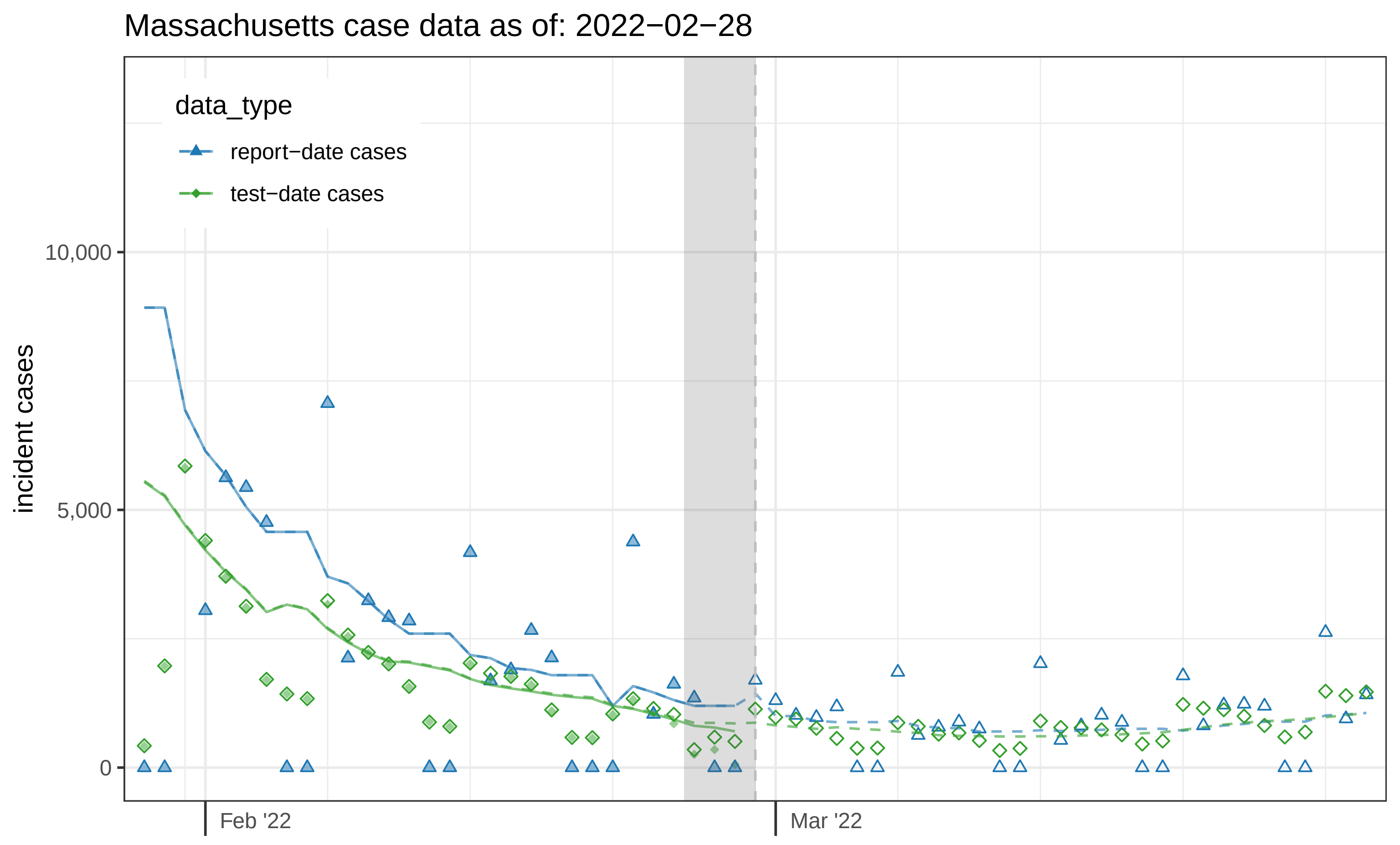
Massachusetts case data as of: 2022−02−28
data_type
report−date cases
test−date cases
incident cases
10,000
5,000
0
Feb '22
Mar '22

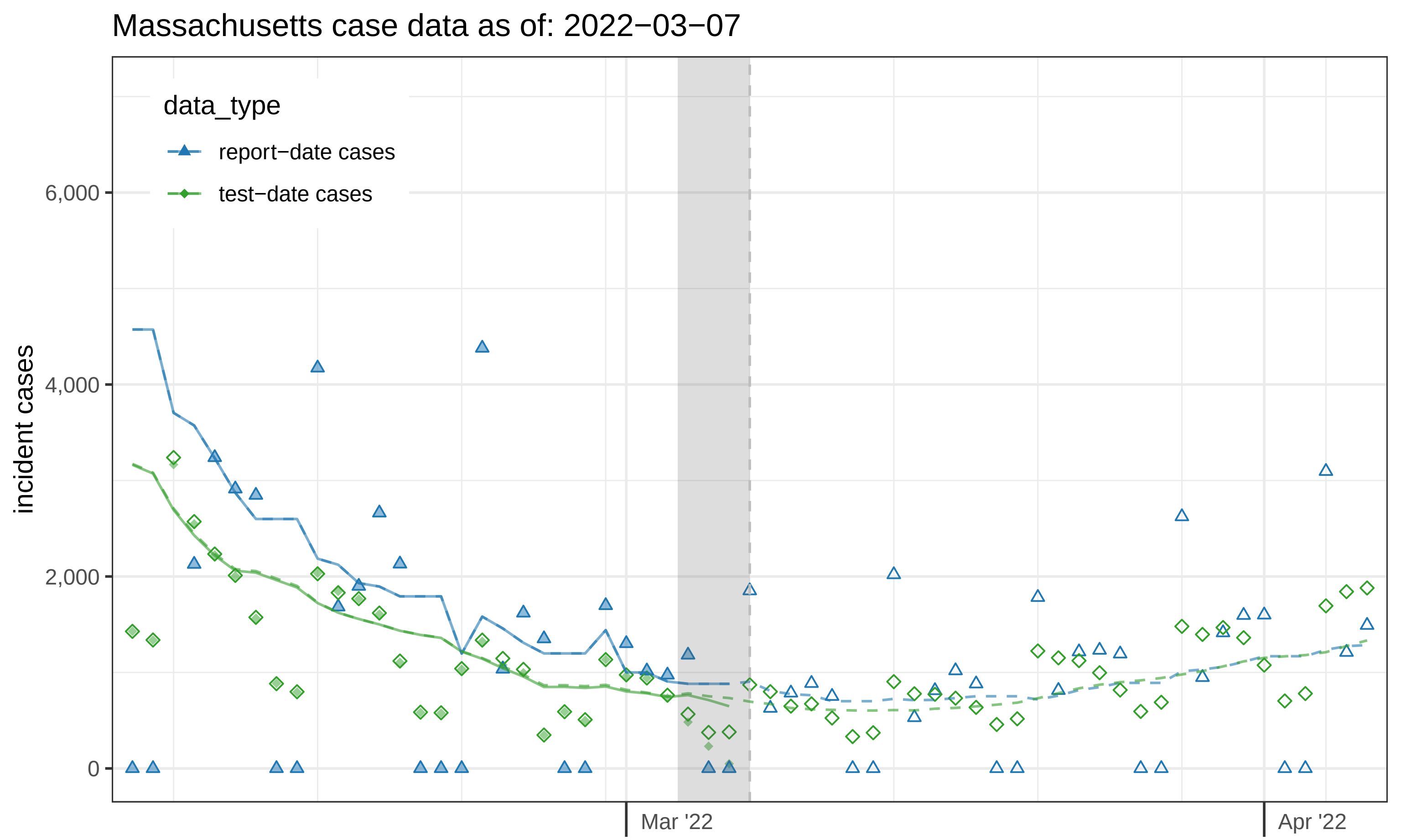
Massachusetts case data as of: 2022−03−07
data_type
report−date cases
test−date cases
incident cases
6,000
4,000
2,000
0
Mar '22
Apr '22

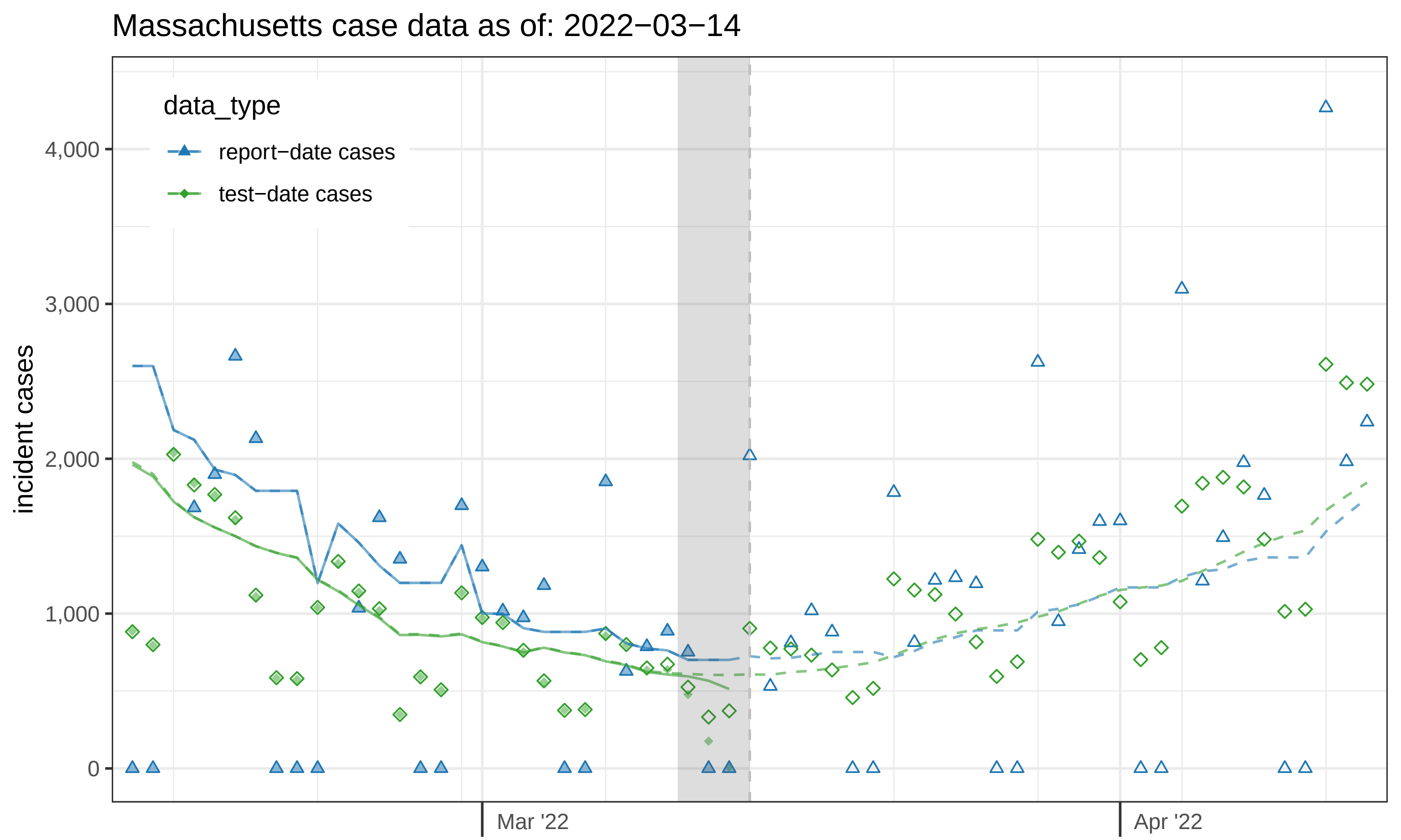

Massachusetts case data as of: 2022−03−14
data_type
report−date cases
test−date cases
incident cases
4,000
3,000
2,000
1,000
0
Mar '22
Apr '22

Massachusetts case data as of: 2022−03−21

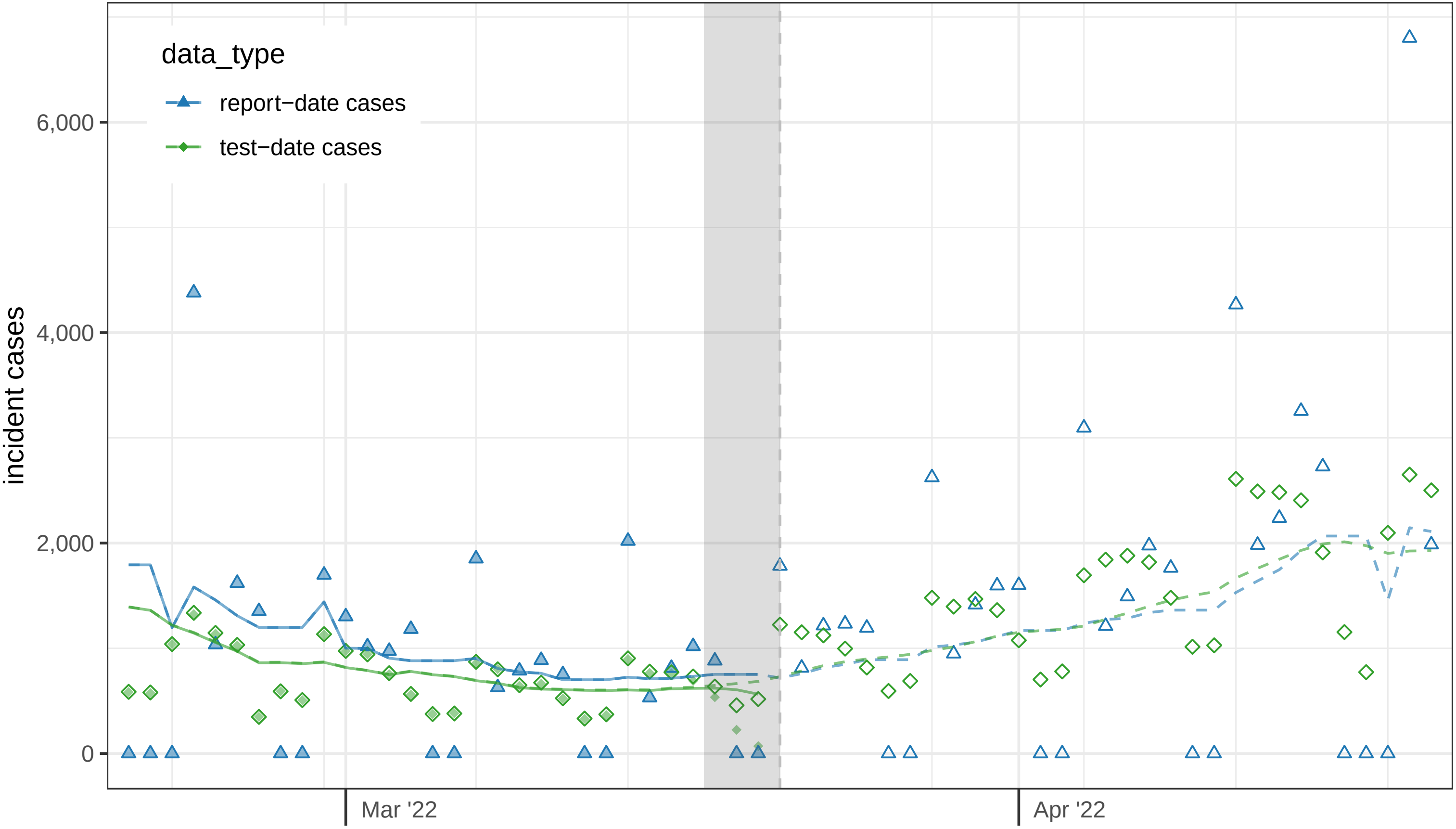

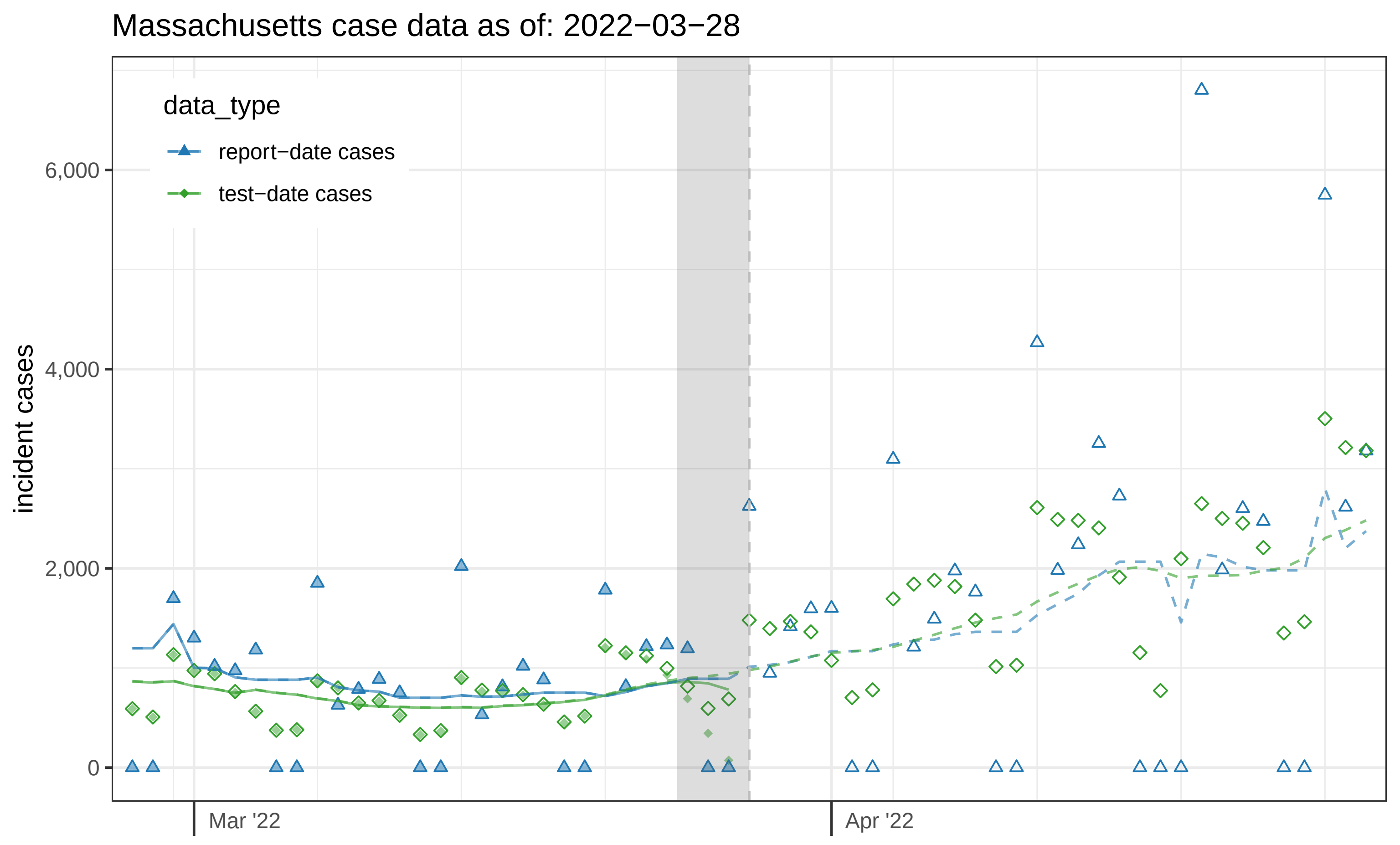

Massachusetts case data as of: 2022−03−28
data_type
report−date cases
test−date cases
incident cases
6,000
4,000
2,000
0
Mar '22
Apr '22

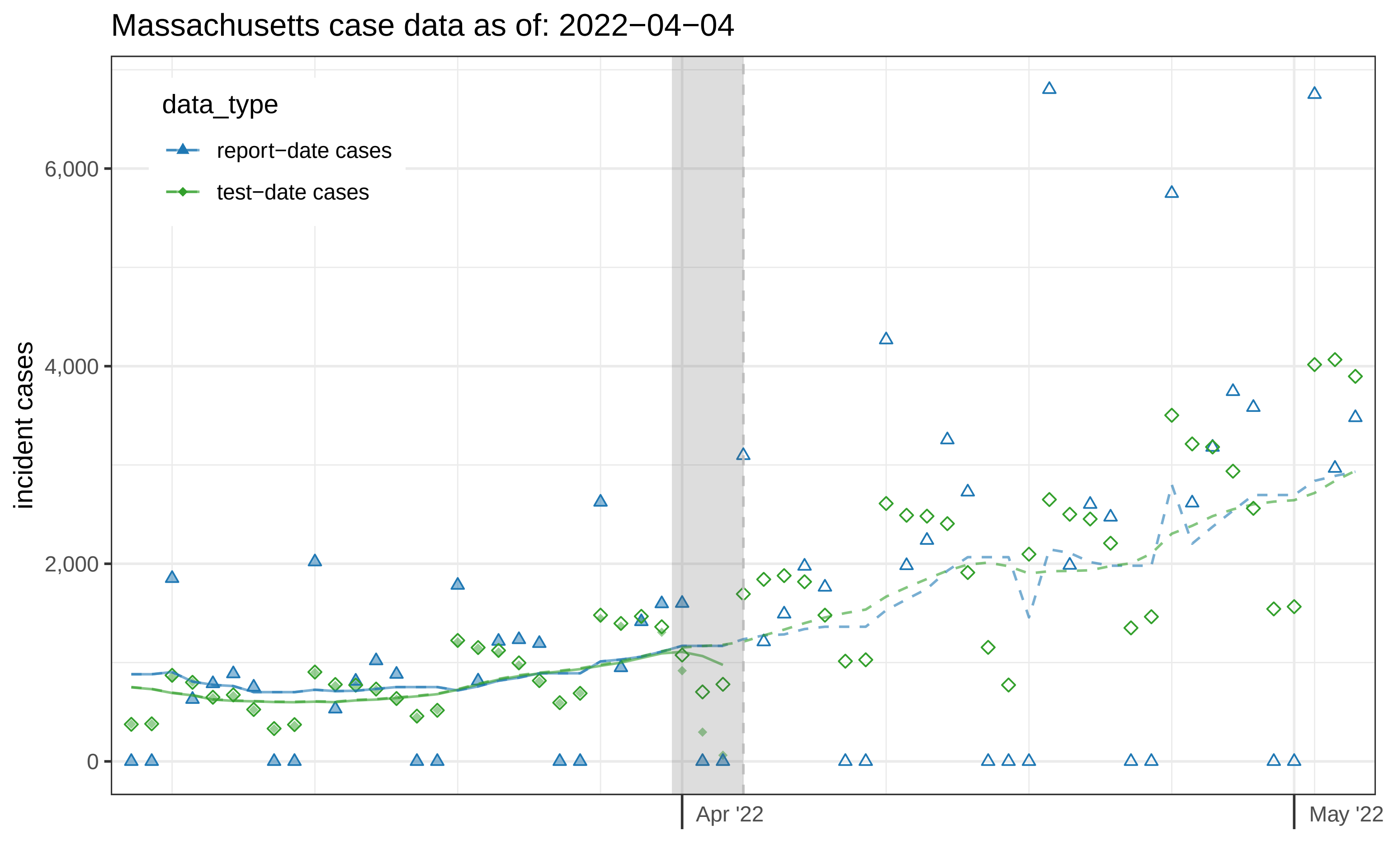

Massachusetts case data as of: 2022−04−04
data_type
report−date cases
test−date cases
incident cases
6,000
4,000
2,000
0
Apr '22
May '22

Massachusetts case data as of: 2022−04−11

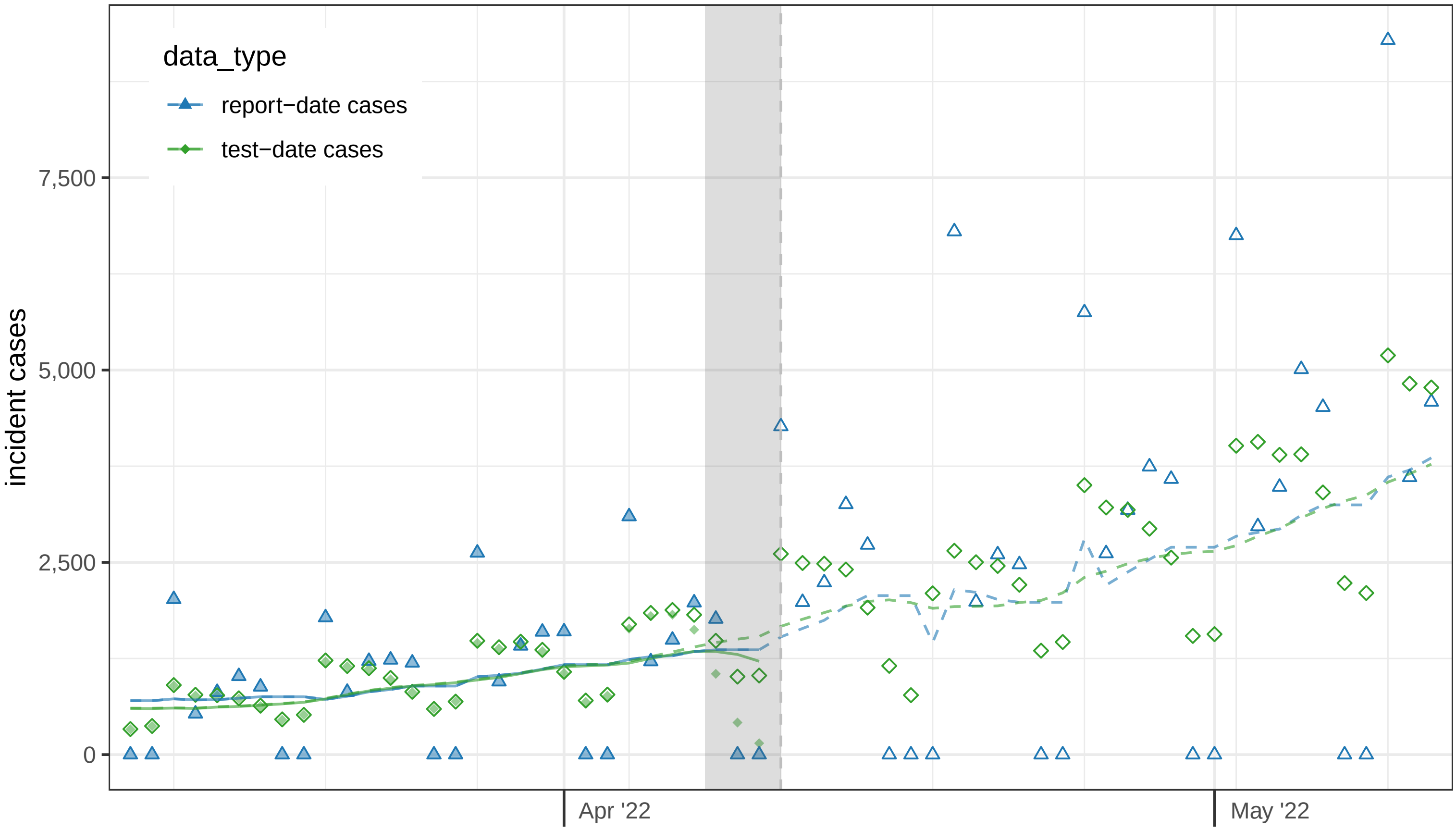

Massachusetts case data as of: 2022−04−25

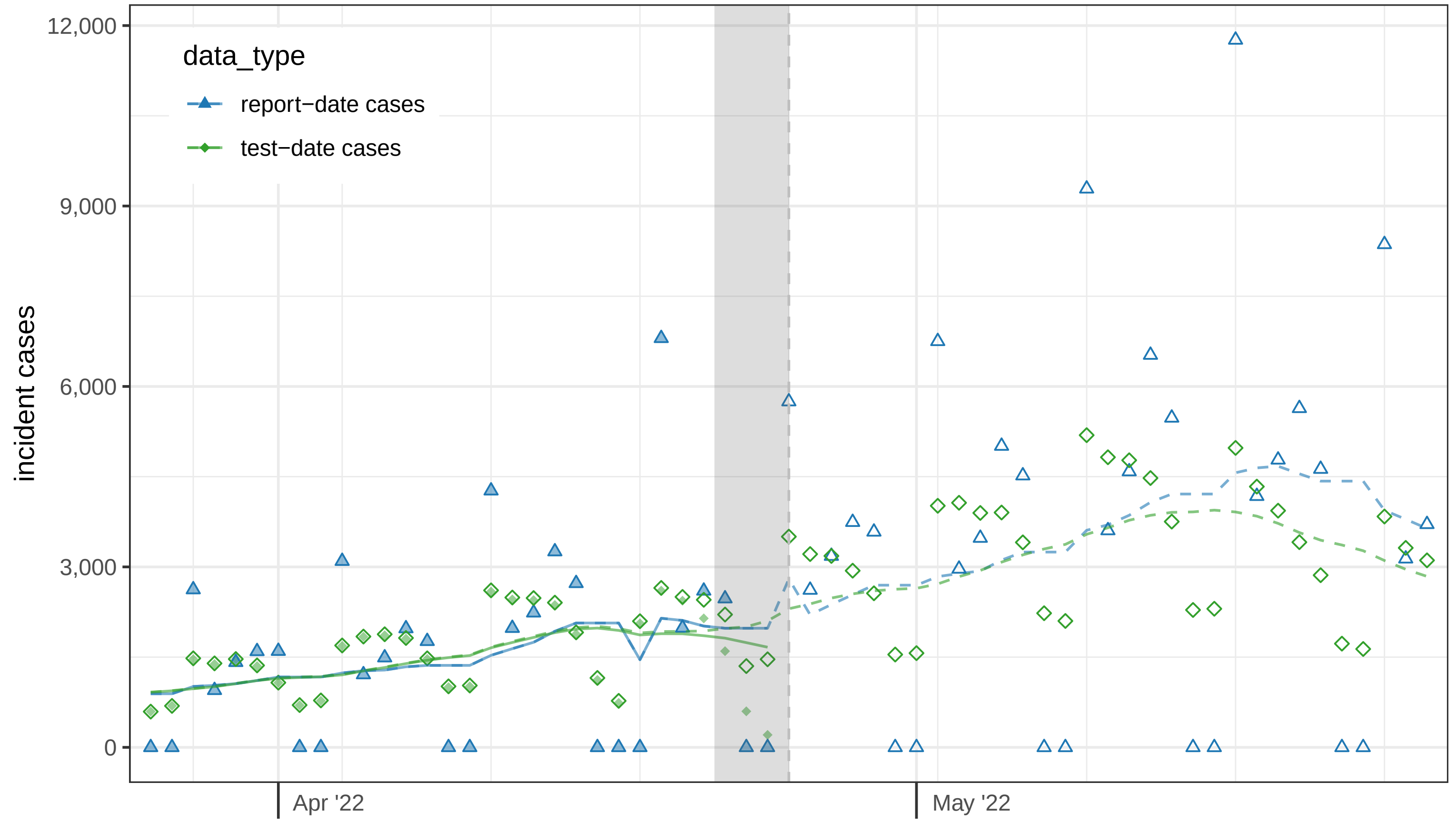

Massachusetts case data as of: 2022−05−02

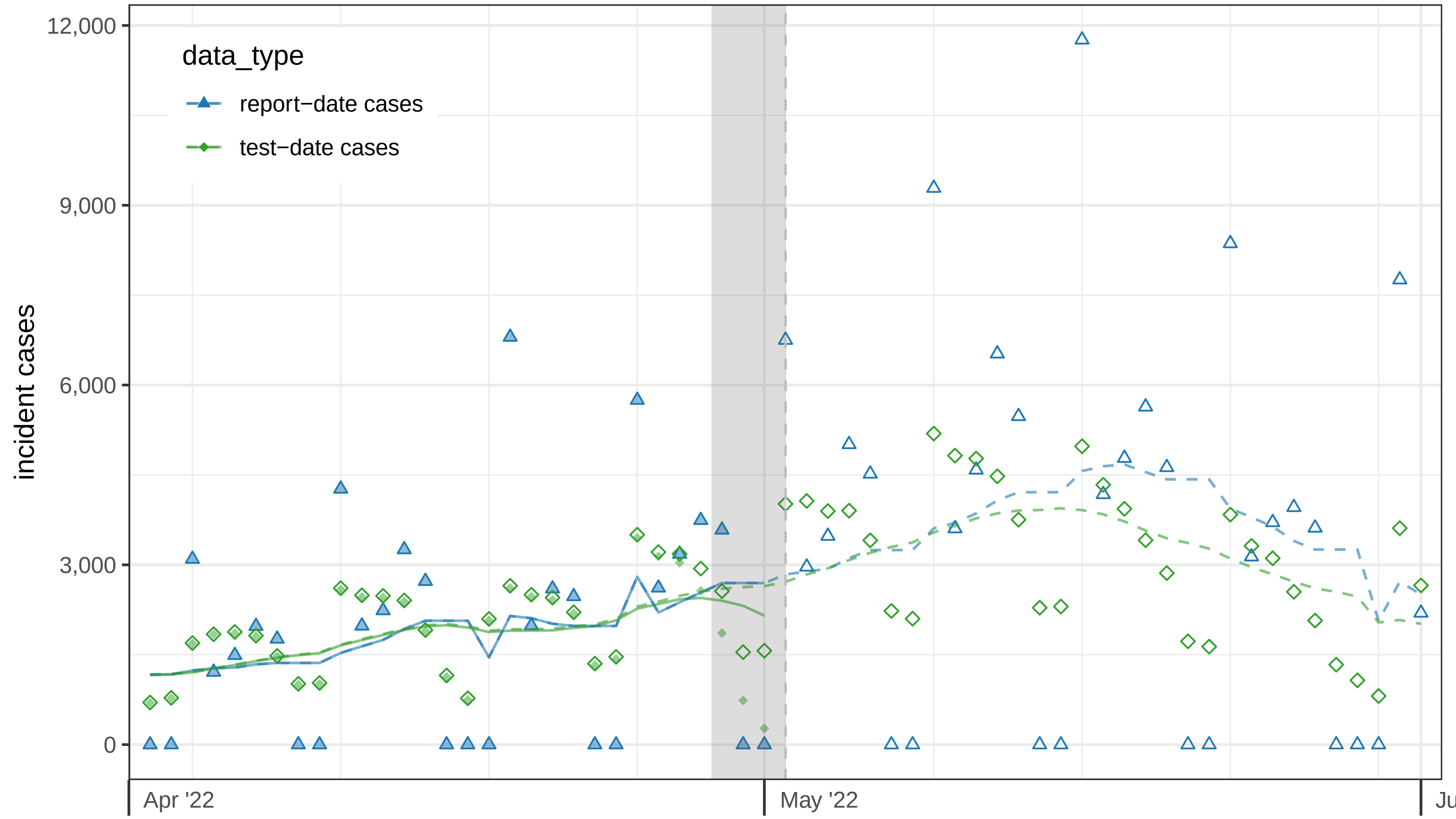

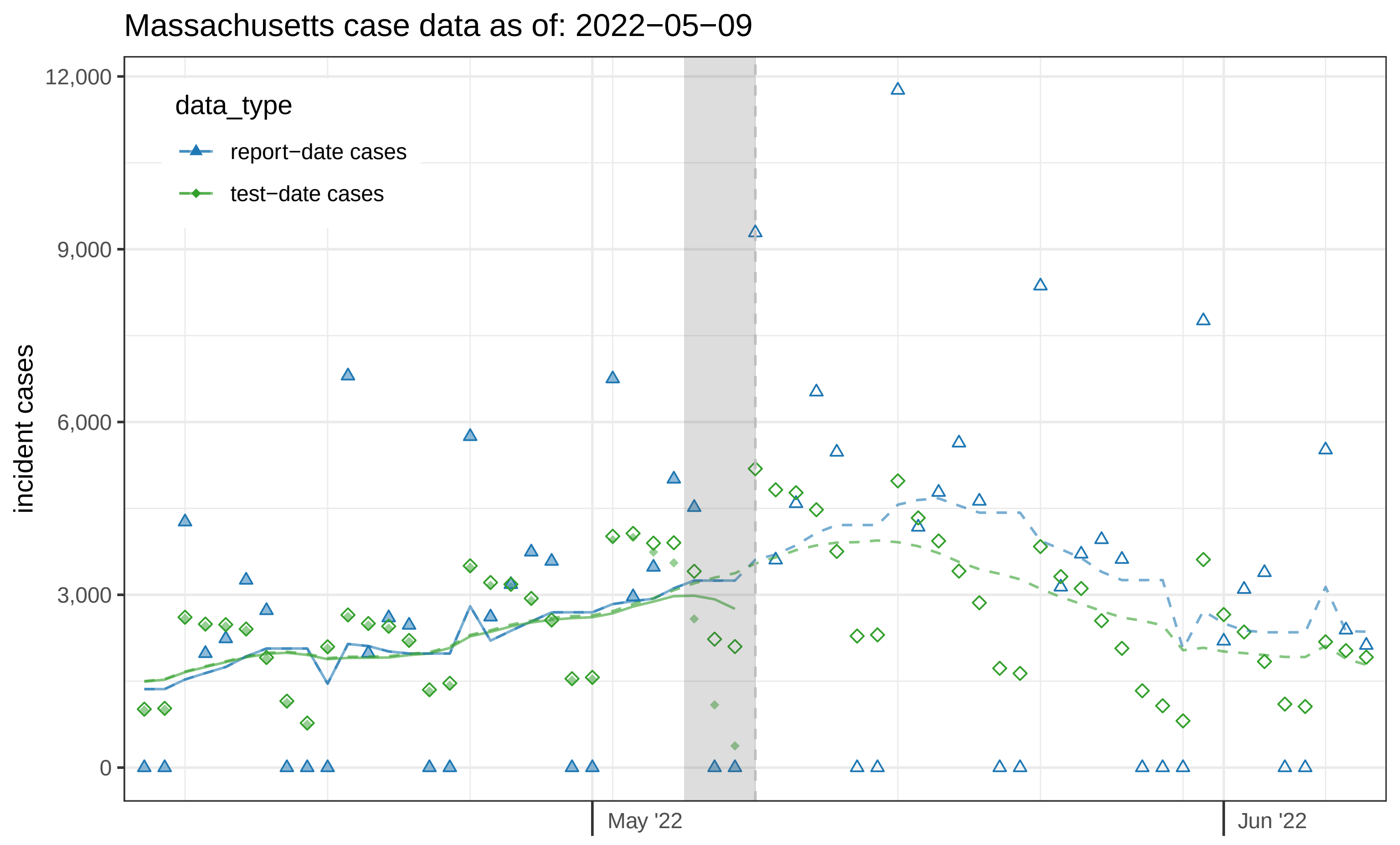
Massachusetts case data as of: 2022−05−09
data_type
report−date cases
test−date cases
incident cases
12,000
9,000
6,000
3,000
0
May '22
Jun '22

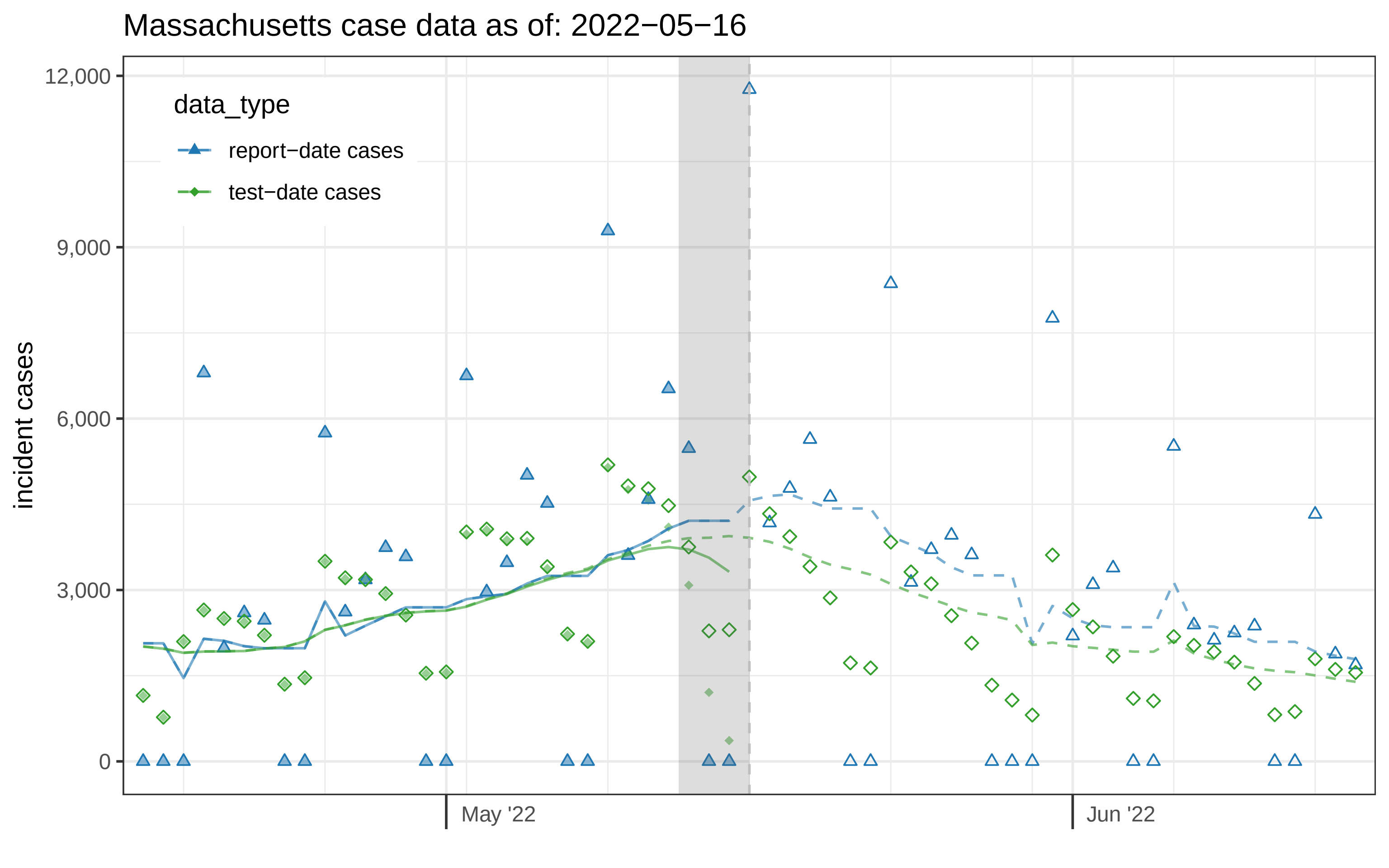

Massachusetts case data as of: 2022−05−16
incident cases
12,000
9,000
6,000
3,000
0
data_type
report−date cases
test−date cases
May '22
Jun '22

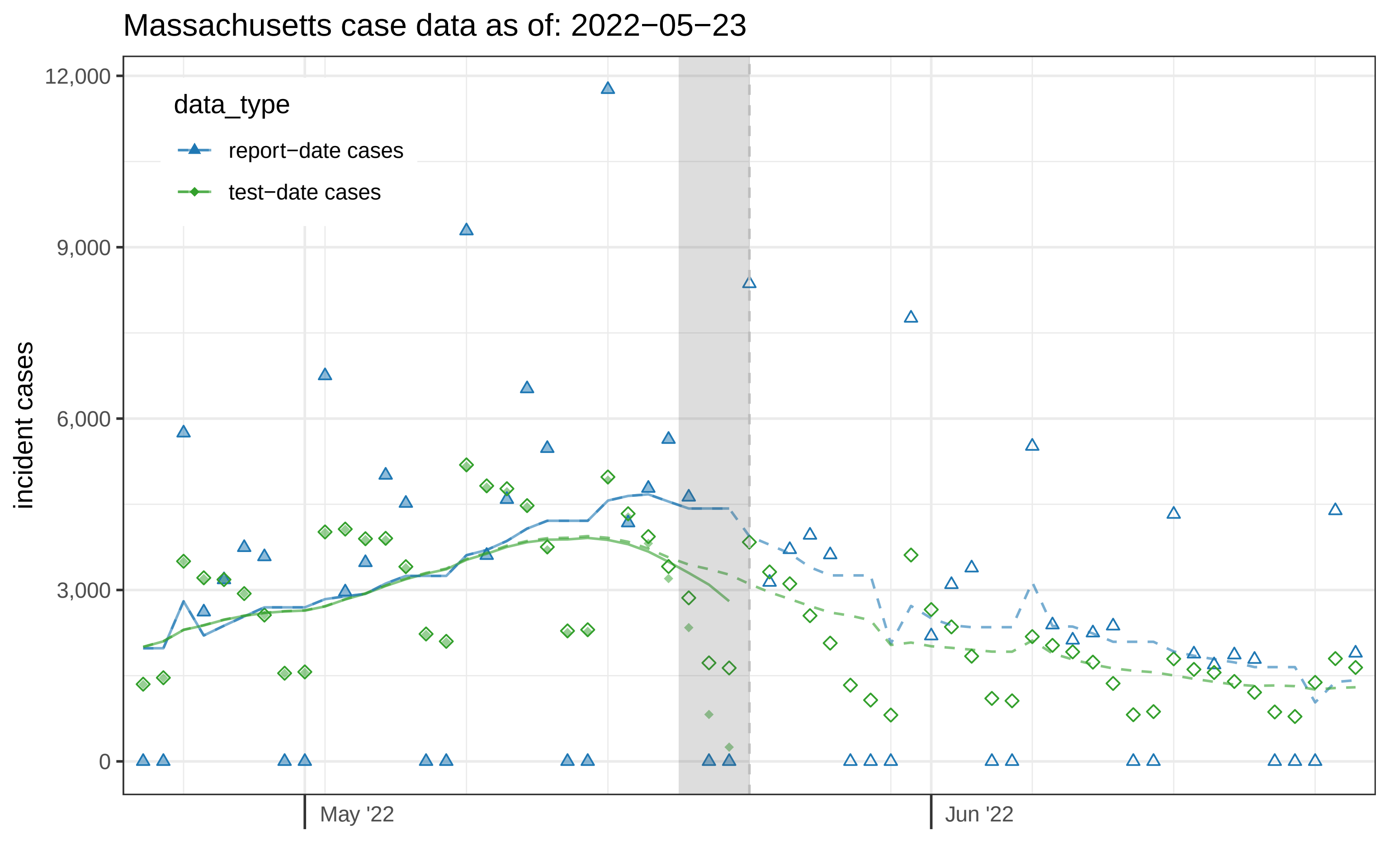
Massachusetts case data as of: 2022−05−23
incident cases
12,000
9,000
6,000
3,000
0
data_type
report−date cases
test−date cases
May '22
Jun '22

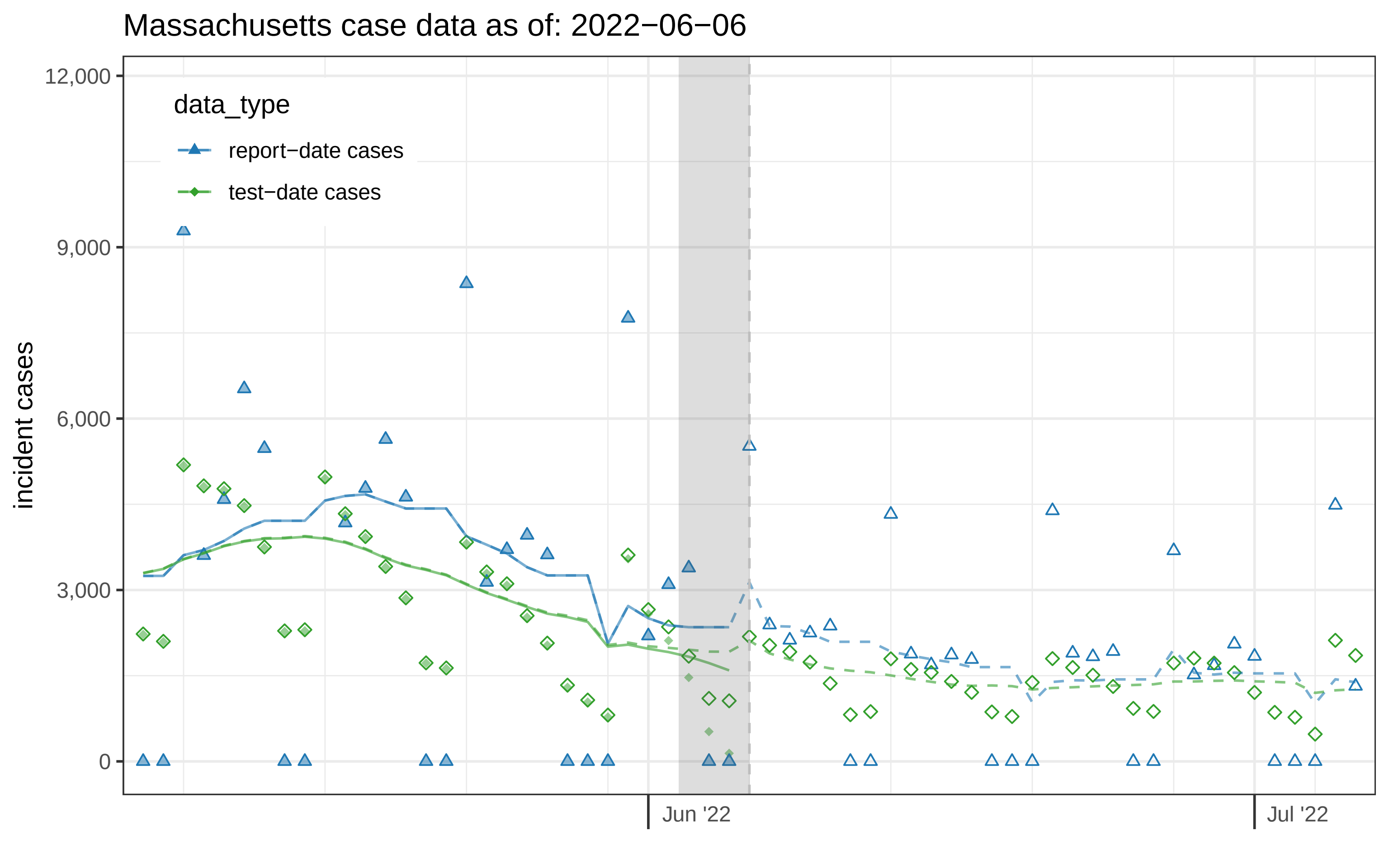
Massachusetts case data as of: 2022−06−06
data_type
report−date cases
test−date cases
incident cases
12,000
9,000
6,000
3,000
0
Jun '22
Jul '22

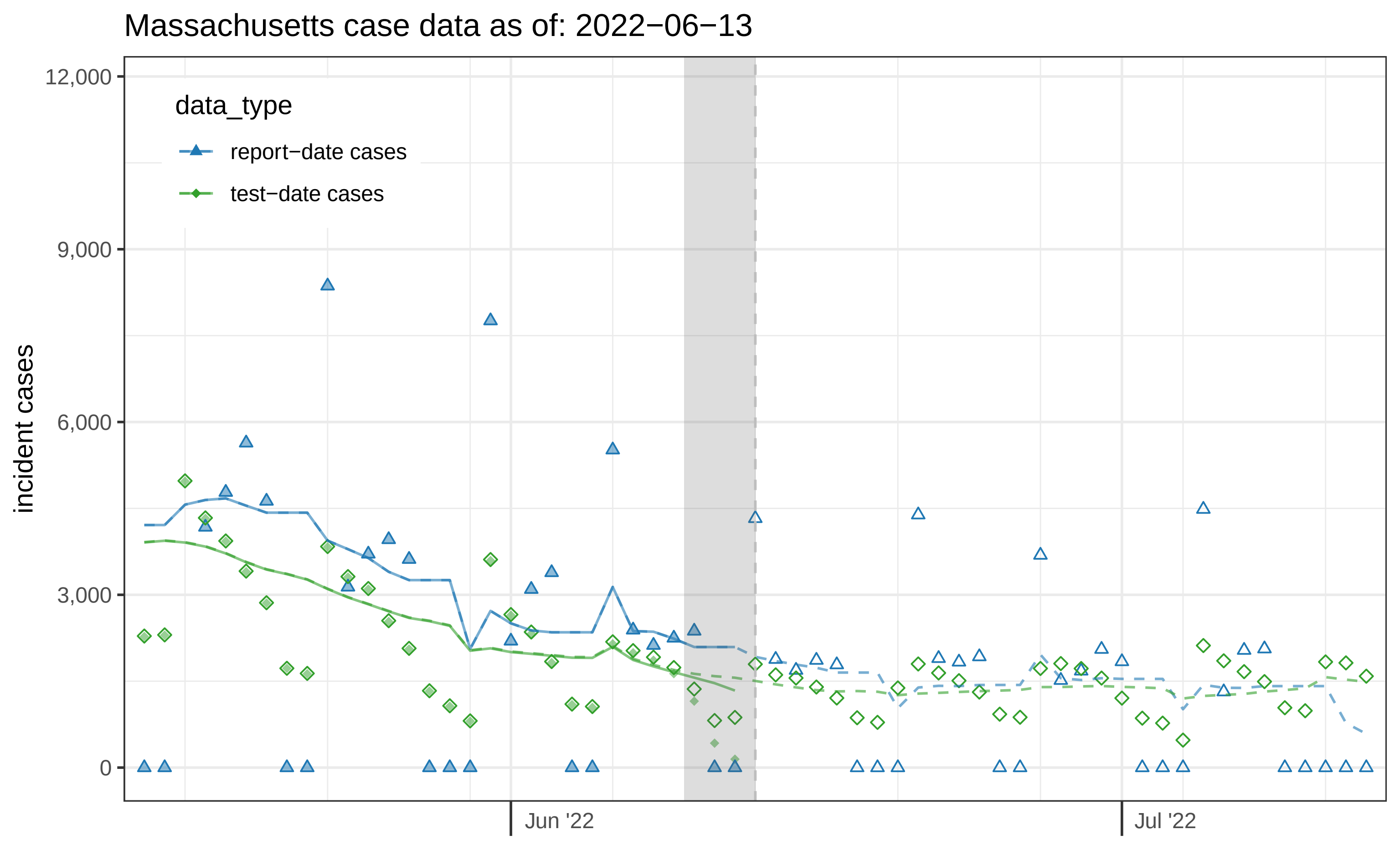
Massachusetts case data as of: 2022−06−13
data_type
report−date cases
test−date cases
incident cases
12,000
9,000
6,000
3,000
0
Jun '22
Jul '22

Massachusetts case data as of: 2022−06−27

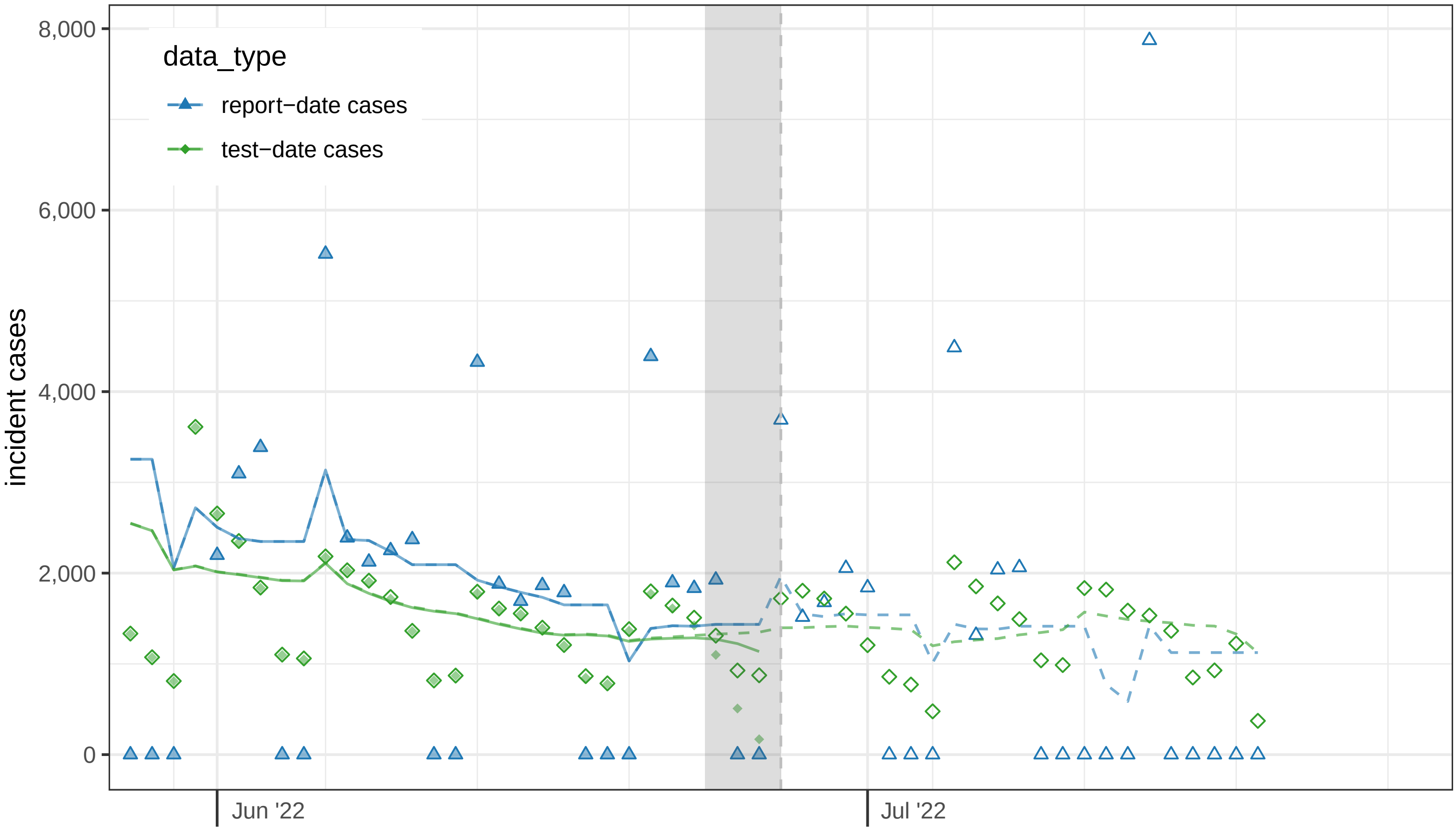

Supplement: Supplement 1 — Supplementary File 1: A booklet of figures showing report-date case signals from JHU CSSE (blue triangles) and test-date case signals from CA and MA DPH (green diamonds). Each page of the booklet shows data for one state and one date. The plots show open shapes for the “finalized” observations as of July 26, 2022 and solid shapes as vintages of data specific to every Monday date from January 4, 2021 through July 26, 2022. The solid and dashed lines shows a trailing 7-day average for the real-time vintages of data and the finalized data, respectively. The shaded region highlights how the last 3 (for MA) or 7 (for CA) days of data, especially for the test-date cases, tend to be under-reported. [file media-1.pdf]
